# The 15th Asia-Pacific Conferenceon Vision (APCV), 2019

**DOI:** 10.1177/2041669519877985

**Published:** 2019-10-15

The 15th Asia-Pacific Conference on Vision was held in Osaka, JAPAN, from 29th of July to 1st of August, 2019. The conference aimed to facilitate discussion on vision research in Asian-Pacific region, attended by 458 participants from all over the world. The program consisted of four keynote lectures, 13 symposia including 57 speakers, and 50 oral and 220 poster presentations. The organizing committee are grateful to all the contributions.

The Abstracts are provided below. Keynote talks are presented first, symposia second, and then the contributed talks and posters are listed by session.

APCV 2019 was funded partially by a grant from NICT International Exchange Program from the National Institute of Information and Communications Technology.

## Organizing committee of APCV 2019Chairs

**Table:** 

Izumi Ohzawa	CiNet, Osaka University
Takao Sato	Ritsumeikan University

## Executive Chair

**Table:** 

Kowa Koida	Toyohashi University of Technology

## Committee members (in an alphabetical order)

**Table:** 

Akiyoshi Kitaoka	Ritsumeikan University
Chieko Koike	Ritsumeikan University
Hidetoshi Kanaya	Ritsumeikan University
Hiroshi Ashida	Kyoto University
Ichiro Fujita	CiNet, Osaka University
Ichiro Kuriki	Tohoku University
Kaoru Amano	CiNet, NICT
Masataka Sawayama	NTT
Satoshi Shioiri	Tohoku University
Shin’ya Nishida	Kyoto University, NTT
Takahiro Kawabe	NTT
Takashi Murakami	Ritsumeikan University
Tatsuya Sato	Ritsumeikan University

## Abstract reviewing committee

**Table:** 

Akiyoshi Kitaoka	Ritsumeikan University
Atsushi Wada	CiNet, NICT
Chia-huei Tseng	Tohoku University
Chieko Koike	Ritsumeikan University
Hidetoshi Kanaya	Ritsumeikan University
Hiromasa Takemura	CiNet, NICT
Hiroshi Ashida	Kyoto University
Hiroshi Ban	CiNet, NICT
Hiroshi Tamura	CiNet, Osaka University
Ichiro Fujita	CiNet, Osaka University
Ichiro Kuriki	Tohoku University
Izumi Ohzawa	CiNet, Osaka University
Jun Saiki	Kyoto University
Kaoru Amano	CiNet, NICT
Katsunori Kitano	Ritsumeikan University
Kota Sasaki	CiNet, Osaka University
Kowa Koida	Toyohashi University of Technology
Masataka Sawayama	NTT
Mikio Inagaki	CiNet, Osaka University
Naokazu Goda	NIPS
Satoshi Shioiri	Tohoku University
Shin’ya Nishida	NTT
Takahiro Kawabe	NTT
Takahisa Sanada	Kansai Medical University
Tetsuya Yagi	Osaka University
Tomoyuki Naito	Osaka University
Yuji Wada	Ritsumeikan University
Yuki Hayashida	Osaka University

## Co-hosts

Vision Society of Japan

Vision Science Forum

Osaka University

Ritsumeikan University

## Council members of APCV

**Table:** 

David Crewther	Swinburne University of Technology
Mowei Shen	Zhejiang University
Hong Xu	Nanyang Technological University
Pi-Chun Huang	National Cheng Kung University
Kowa Koida	Toyohashi University of Technology
Sang-Hun Lee	Seoul National University

## History of APCV (ACV)

**Keiji Uchikawa**

Kanagawa Institute of Technology, Tokyo Institute of Technology

## 

APCV (formerly known by Asia Conference on Vision; ACV) was first organized by the Vision Society of Japan and the Vision Research Group in Korea in 2001. The purpose of having ACV was to stimulate, especially for young researchers, to discuss and communicate one another in the international conference, since in East Asia there had been no international vision specific conference, so vision researchers in East Asia had been suffering from difficulty to present their papers in an international conference. In 1999, Keiji Uchikawa (Chairman of the Vision Society of Japan) and Chan-Sup Chung (Chairman of the Vision Research Group in Korea) agreed with each other on having a new international vision conference held in Asia. After two-year preparing period ACV was, first, realized in Japan being supported by many people including Chinese vision researchers.

At the first conference held in Hayama, Japan, as ACV 2001, we had attendees mostly from two countries, Korea and Japan. After the 2nd and 3rd conferences were held in Gyeongju (2002) and Chongqing (2004), the 4th conference was held in Matsue (2006). At the 5th conference in Brisbane, Australia 2008, the conference name changed to Asia-Pacific Conference on Vision (APCV) to cover pacific regions. After the conference in Taipei (2010), APCV have been held annually.

## Keynote Lecture 1 (July 29, 2019)

## Reconstructing Visual and Subjective Experience from the Brain

**Yukiyasu Kamitani**

Kyoto University and ATR Computational Neuroscience Laboratories

## 

The internal visual world is thought to be encoded in hierarchical representations of the brain. However, previous attempts to visualize perceptual contents based on machine-learning analysis of fMRI patterns have been limited to reconstructions with low level image bases or to matchings to exemplars. While categorical decoding of imagery contents has been demonstrated, the reconstruction of internally generated images has been challenging. I introduce our recent study showing that visual cortical activity can be decoded (translated) into the hierarchical features of a pre-trained deep neural network (DNN) for the same input image, providing a way to make use of the information from hierarchical visual features. Next I present a novel image reconstruction method, in which the pixel values of an image are optimized to make its DNN features similar to those decoded from human brain activity at multiple layers. Our method was able to reliably produce reconstructions that resembled the viewed natural images. While our model was solely trained with natural images, it successfully generalized to artificial shapes, indicating that our model was not simply matching to exemplars. The same analysis applied to mental imagery demonstrated rudimentary reconstructions of the subjective content. Our method can effectively combine hierarchical neural representations to reconstruct perceptual and subjective images, providing a new window into the internal contents of the brain.

## Keynote Lecture 2 (July 30, 2019)

## Pluripotent Stem Cell Derived Photoreceptor Transplantation

**Masayo Takahashi**

Center for Biosystems Dynamics Research, RIKEN

## 

Photoreceptor transplantation is a possible treatment to restore visual function to photoreceptor only degenerated retinas such as retinitis pigmentosa. We have been reported that the sensory retinal sheet transplantation, which supplies photoreceptors and secondary retinal neurons, can reintroduce visual function in mice with end-stage retinal degeneration. For the photoreceptor transplantation, previously many groups used photoreceptor suspension. However, it was revealed in 2017 that in those suspension transplantation GFP positive cells were host cells that have material transfer from grafted cells. In fact, transplanted retinal sheets survive for a longer period than suspended cells. Furthermore, only retinal sheets could become mature in the subretinal space and obtain tissue structure with outer segments of photoreceptors after transplantation. To confirm the functional recovery by the grafted sheets, we developed new disease mice models and functional tests analysis. With those, synaptic contact between graft photoreceptors and host bipolar cells was confirmed by immunohistochemistry, electrical physiology, and behavior tests. MEA recording showed that grafted cells could elicit light responses in the host ganglion cells.

Now we are investigating how to increase the number of synapses and efficacy of transplantation. However, immunohistochemical characterization of synapse in the degenerated retina or of grafted retina is often challenging, as traits are not as clear as in the wild type retina. Therefore, using postnatal wild type mouse retina as a training data, we developed a new method to objectively count synapses. Using this method, we evaluated the synapses of iPSC-retina after transplantation to rd1 mice. The number of synapses increased on 30 days after transplantation, while we could not found any synapse formation in in vtro retinal organoids. The synapse numbers were more in the light/dark cycle environment than completely dark one.

I will talk about the future strategy of outer retinal transplantation.

## Keynote Lecture 3 (July 31, 2019)

## A Motor Theory Of Sleep-Wake Control

**Yang Dan**

University of California, Berkeley

## 

Sleep is a fundamental biological process observed throughout the animal kingdom, and its disruption has devastating health consequences. Using a combination of optogenetics, electrophysiology, imaging, and gene expression profiling, we identify key neurons in the sleep control circuits and map their synaptic connections. Sleep appears to be controlled by a highly distributed network spanning the forebrain, midbrain, and hindbrain, where REM and non-REM sleep neurons are part of the central somatic and autonomic motor circuits. The intimate association between the sleep and autonomic/somatic motor control circuits suggests that a primary function of sleep is to suppress movement and promote processes incompatible with motor activity.

## Keynote Lecture 4 (August 1, 2019)

## ISETBIO: Software for the Foundations Of Vision Science

**Brian Wandell**

Stanford University

## 

Over four centuries scientists have developed theories and made measurements that characterize how the visual system converts light into neural signals. This knowledge is contained in a diverse set of textbooks and research papers that can be hard for young students—or old professors—to integrate. We are developing an open-source Matlab toolbox, ISETBIO, that combines the historical knowledge into a set of accessible computations. Our hope is that many people can check the computations and then use them as a basis for further theorizing about visual system function. Our team has implemented software (Matlab, Docker) to provide scientists and engineers with (a) quantitative computer graphics methods to create a wide range of complex 3D scene spectral radiances, (b) optics simulations that transform these 3D radiances into retinal irradiance using physiological models of the eye, (c) quantitative specifications of the lens transmission, macular pigment and photopigments, (d) fixational eye movement models, (e) photoreceptor spatial sampling from fovea to periphery, (f) cone excitation calculations, and (g) cone photocurrent models. This talk will explain how the current computations, which characterize the initial stages of visual encoding, can be helpful to research scientists and engineers seeking to understand the information available in the nervous system and how this information impacts perceptual judgments, such as Vernier acuity, contrast sensitivity, and color and motion sensitivity. We hope some of you will check our work and extend it. I will discuss ways these tools might be helpful in developing theories of visual performance and perception. Collaborative work with David Brainard, Nicolas Cottaris, Trisha Lian, Zhenyi Liu, Joyce Farrell, Haomiao Jiang, Fred Rieke, and James Golden.

## Symposium 1-1 (July 29, 2019)

## Physiological, Psychological, and Computational Foundations of Scene Understanding

**Organizers: Yukako Yamane^1^ and Ko Sakai^2^**

^1^Osaka University, Okinawa Institute of Science and Technology

^2^University of Tsukuba

## 

Vision science has revealed the nature of human vision and the visual functions in cognition in various ways, although ‘how humans understand an entire scene?’ is still a challenging problem. How does the visual system segregate images into meaningful parts and then assemble those parts into informative representations of the outside world? How do those representations support our immediate, intuitive knowledge about where we are, what things are present, and how those things relate to each other and to the overall scene? About what just happened in the scene, and what might happen next and how we should react for? Recent rapid advancement of machine learning algorithms enabled the identification, description, and even generation of a scene, however, they are still incapable of providing clues for understanding a scene as we humans do. We invite world-leading scientists to discuss the physiological, psychological, and computational foundations of scene understanding.

## Objects, Scenes, and Gravity in Ventral Pathway Visual Cortex

**Alexandriya Emonds, Siavash Vaziri and Charles E. Connor**

Krieger Mind/Brain Institute, Johns Hopkins University

## 

It has long been thought that the ventral pathway is dedicated exclusively to visual object processing, while scene understanding is primarily a dorsal pathway function. However, we reported recently that many neurons in macaque monkey ventral pathway, including a majority in the TEd channel, are far more responsive to large-scale scene structure. These neurons are particularly tuned for tilt and slant of planar surfaces and edges, in ways that suggest they represent the direction of gravity. In new experiments, we have begun to examine how object information and scene information are integrated in the ventral pathway. Early results show that individual neurons can be congruently selective for ground tilt, object tilt, and object balance (distribution of mass with respect to points of ground contact). This is consistent with the theory that visual cortex serves as an intuitive physics engine for understanding the natural world, in particular the energetic potentials and constraints imposed on objects by the ubiquitous force of gravity.

## Human Scene-selective Areas Represent the Orientation of and Distance to Large Surfaces

**Mark Lescroart**

University of Nevada, Reno

## 

A network of areas in the human brain—including the Parahippocampal Place Area (PPA), the Occipital Place Area (OPA), and the Retrosplenial Complex (RSC)—respond to images of visual scenes. Long-standing theories suggest that these areas represent the 3D structure of the local visual environment. However, most experiments that study representation of scene structure have relied on operational or categorical definitions of scene structure—for example, comparing responses to open vs closed scenes. It is not clear, based on such studies, how these areas might respond to scenes that do not fall into one of the investigated categories. Furthermore, it has been hypothesized that sceneselective areas represent 2D cues for 3D structure rather than 3D structure per se. To evaluate the degree to which each of these hypotheses explain variance in scene-selective areas, we develop an encoding model of 3D scene structure and test it against a model of low-level 2D features. Our 3D structural model uses continuous parameters based on 3D data (surface normals and depth maps) rather than human-assigned categorical labels such as “open” or “closed”. We fit the models to fMRI data recorded while subjects viewed visual scenes. Variance partitioning on the fit models reveals that scene-selective areas represent the distance to and orientation of large surfaces, at least partly independent of low-level features. Individual voxels appear to be tuned for combinations of the orientation of and distance to large surfaces. Principal components analysis of the model weights reveals that the most important dimensions of 3D structure are distance and openness. Finally, reconstructions of the stimuli based on our model demonstrate that the model captures unprecedented detail about the 3D structure of local visual environment from BOLD responses in scene-selective areas.

## Perceptual Organization and Attention to Objects

**Ernst Niebur^1,2^**

^1^Solomon Snyder Department of Neuroscience and Zanvyl Krieger Mind

^2^Brain Institute, Johns Hopkins University

## 

One of the most important strategies of dealing with the extremely high complexity presented by the visual signal from a cluttered scene is to organize the input into perceptual objects. The task of scene understanding is then transformed from interpreting ∼10^6^ rapidly changing input signals in terms of a much smaller number of spatio-temporal patterns that mostly correspond to structures in the real world, and are constrained by physical laws. This task is, however, highly non-trivial and requires to group those elements of the raw input that correspond to the same object, and segregate them from those corresponding to other objects and the background. We propose that primates solve this perceptual organization task using small populations of dedicated neurons that represent different objects. We note that this segregation process does not require the formation of fully-defined recognizable objects: computational models show that this can be accomplished on perceptual pre-cursors of objects with very simple properties that we call proto-objects. Key features of the models are “grouping” neurons integrating local features into coherent proto-objects and excitatory feedback to the same local feature neurons which caused the associated proto-object’s grouping neuron’s activation. Organization of the scene into proto-objects thus transforms the seemingly impossible task of scene understanding into manageable sub-tasks. For instance, object recognition can then proceed in a sequential fashion, by operating on one proto-object at a time. A more general mid-level task is attention to objects and the model explains how attention can be directed (top-down) to objects even though the central structures that control top-down attention do not have a representation of the detailed features of these objects.

## How Interactions Between Shading and Color Inform Object and Scene Understanding

**Steven W. Zucker**

Department of Computer Science, Yale University

## 

What is the shape of an apple, and what color is it? These are instances of classical questions about object perception: (i) how is it possible that we can infer the three-dimensional shape of an object (e.g., an apple) from its shading, and (ii) how is it possible that we can separate the intensity changes due to material effects (the apple’s pigmentation) from intensity changes due to shading. Clearly mistakes in solving (ii) would impact (i). Importantly, these two problems arise in scene perception as well. Objects are described by surfaces and their parts; scenes are described by surface arrangements and their interactions. For example, when walking along a path through the woods, where is the ground surface and why do shadows not effect its perceived shape? Again, mistakes would impact performance if shadows were interpreted as holes.

We introduce a computational approach to these questions grounded in basic neurophysiology and computational theory. Regarding problem (i), we outline a novel approach to shape-from-shading inferences that is based on visual flows, a mathematical abstraction of how information is represented in visual cortex. Regarding (ii), we introduce a model of color representation (hue flows) also based on visual cortex, that is analogous to the shading flows. Then, given both flows, we can determine when hue co-varies generically with shading, thereby addressing problem (ii) and implying a material effect. We demonstrate psychophysically how hue flows can be designed to alter shape percepts, and we demonstrate computationally how hue flows pass through cast shadows. Taken together, key aspects of our abilities to wander through scenes and to describe and manipulate objects are both supported by the foundational interactions of shading and hue flows.

## Symposium 1-2 (July 29, 2019)

## Unpacking Cognitive and Neural Mechanisms Underpinning The Recognition and Representations of Unfamiliar and Familiar Faces and Facial Expressions: Behavioral, Eye Movement and ERP Studies

**Organizer: Kazuyo Nakabayashi**

University of Hull

## 

This symposium addresses some of the key debates in studies of face recognition and expressions. Here, we provide novel evidence demonstrating perceptual, cognitive and neural mechanisms underlying the representations and recognition of familiar and unfamiliar faces, and facial expressions across a range of paradigms. One study concerns the role of facial features in detecting facial expressions through manipulation of spatial frequencies. Two studies report perceptual matching of unfamiliar faces, with one study concerning effects of culture (Japanese vs. British) on eye movements, inversion effects and Thatcherization. The other study sheds light on the relative contribution of featual and holistic processing to same- and different-identity matching. The two remaining studies investigate cognitive and neural representations of familiar faces, with one study manipulating appearance of famous faces and their popularity in order to elucidate the representations of familiar faces in the cognitive system. The other study measures event-related brain potentials (N250) to reveal how semantic and visual information may interact, giving a rise to the recognition of familiar faces. The symposium will provide a comprehensive view towards how faces and expressions are processed and stored in memory.

## Three Dimensional Configuration and Spatial Frequency Properties of Facial Expression

**Sakura Torii**

Kobe Shoin Women’s University

## 

The features of happy, angry, sad and surprised faces were studied. In the happy face, it was found that distinct characteristic changes occurred around the cheeks by the three dimensional analysis, and that the deviation range of gray level was wider than that measured in other faces by gray level analysis. By face recognition experiment using frequency-filtered face images, it was found that only happy face was easily recognized than other faces even under low-pass condition. These results suggested that emphasizing the contrast in a wide region of the facial surface increased the three-dimensional features, which possibly resulted in the selective enhancement of the “happy face features”. I foresee new make-up foundations that can only emphasize and develop the “happy face feature”.

## The Role of Inversion and Thatcherization in Matching Own- and Other-Race Faces

**Kazuyo Nakabayashi**

University of Hull

## 

In the Thatcher illusion a face in which the eyes and mouth are inverted looks grotesque when shown upright but not when inverted. Two experiments examined the contribution of feature-based and configural processing to matching normal and Thatcherized pairs of isolated face parts (i.e., the eyes and mouth), and how perceptual matching would be influenced by the race of face and inversion (better performance for upright than inverted faces). White British and native Japanese groups made same/different judgements to isolated face parts. Experiment 1 had the same identity pairs, encouraging feature-based processing while Experiment 2 having different identity pairs, which induced configural. Across experiments, effects of inversion and Thatchierzation were found, but these effects varied depending on the race of observer and the race of stimulus face. In addition, eye movements demonstrated increased sampling of inverted compared with upright faces and for Thatcherized compared with normal faces. The findings demonstrate that perceptual biases, shaped by culture-specific strategies and task-based attentional demands, can determine sensitivity to feature-based and local configural information during perceptual encoding.

## Matching Faces: The Facial Features are Important, But So DO the Whole Face. Change ‘DOES’ to ‘DO’

**Alejandro J. Estudillo and Nate Frida**

University of Nottingham Malaysia

## 

Matching two unfamiliar faces is of paramount importance in forensic scenarios but the cognitive mechanisms behind this task are poorly understood. In fact, in contrast to the notion that faces are processed at holistic level, it has been suggested that face matching is driven by featural processing. The present study looks to shed light on this issue by exploring the role of holistic and featural processing for match (i.e., both faces depict the same identity) and mismatch (i.e., both faces depict different identities). Across two experiments, observers were asked to decide whether a pair of faces depicted the same or two different identities, while their eyes were being tracked. In Experiment 1, a gaze-contingent paradigm was used to manipulate holistic/featural processing. In Experiment 2, in addition to a standard face matching task, observers also performed a part/whole task, which provides an index of holistic processing. Results showed that both holistic and featural processing are important for face matching and that neither of them individually suffices for face matching.

## When Brad Pitt Is More Refined than George Clooney: The Role of Stability in Developing Parsimonious Facial Representations

**Christel Devue**

Victoria University of Wellington

## 

Most people can recognise large numbers of faces, but the facial information we rely on is unknown despite decades of experimentation. We developed a theory that assumes representations are parsimonious and that different information is more or less diagnostic in individual faces, regardless of familiarity. Diagnostic features are those that remain stable over encounters and so receive more representational weight. Importantly, coarse information is privileged over fine details. This creates cost-effective facial representations that may refine over time if appearance changes. The theory predicts that representations of people with a consistent appearance (e.g., George Clooney) should include stable coarse extra-facial features, and so their internal features need not be encoded with the same high resolution as those of equally famous people who change appearance frequently (e.g., Brad Pitt). In three preregistered experiments, participants performed a recognition task in which we controlled appearance of actors (variable, consistent) and their popularity (higher, lower). Consistent with our theory, in less popular actors, stable extra-facial features helped remember consistent faces compared to variable ones. However, in popular actors, representations of variable actors had become more refined than those of consistent actors. We will discuss broader implications of our theory for the field.

## The Sustained Familiarity Effect: A Robust Neural Correlate of Familiar Face Recognition

**Holger Wiese^1^, Simone C. Tüttenberg^1^, Mike Burton^2^ and Andrew Young^2^**

^1^Durham University

^2^University of York

## 

Humans are remarkably accurate at recognizing familiar faces independent of a particular picture. However, cognitive neuroscience has largely failed to show a robust neural correlate of image-independent familiar face recognition. Here, we examined event-related brain potentials elicited by highly personally familiar (close friends, relatives) and unfamiliar faces. We presented multiple “ambient” images per identity, varying naturally in lighting, viewing angles, expressions etc. Familiar faces elicited more negative amplitudes in the N250 (200–400 ms), reflecting the activation of stored face representations. Importantly, an increased familiarity effect was observed in the subsequent 400–600 ms time range. This Sustained Familiarity Effect (SFE) was reliably detected in 84% of individual participants. Additional experiments revealed that the SFE is smaller for personally, but less familiar faces (e.g., university lecturers) and absent for celebrities. Moreover, while the N250 familiarity effect does not strongly depend on attentional resources, the SFE is reduced when attention is directed away from the faces. We interpret the SFE as reflecting the integration of visual with additional person-related (e.g., semantic, affective) information needed to guide potential interactions. We propose that this integrative process is at the core of identifying a highly familiar person.

## Symposium 1-3 (July 29, 2019)

## Recent Studies on the Mechanisms of Color Vision and its Role in the Society

**Organizers: Ichiro Kuriki^1^ and Kowa Koida^2^**

^1^Tohoku University

^2^Toyohashi University of Technology

## 

The main topic of this symposium is to introduce the recent progress in the studies on color representation in primate (including human) visual cortex, especially *after* the level of cone-opponent stage, to the vision researchers in physiology, psychophysics, art, and computational field of study in Japan and other countries. Color vision is one of the fundamental features of primate vision. It is used not just in object search and social interactions during the survival of individual, but sometimes give a soul-steering impression in fine arts. The contribution of color on our life is tremendous. On the other hand, descriptions on color-vision mechanisms in most textbooks is stopping at the level of cone-opponent system, while it has been pointed out since early ‘90s that the outputs of cone opponent system do not directly correspond to pure red, blue, green, and yellow sensations (i.e., unique hues). Indeed, there has not been a clear-cut explanation about how our color perception is processed in our brain. However, various possibilities have been proposed on the structure of color-vision and related visual-processing mechanisms in the cortex, which may be related to our color perception (i.e., subjective experience of color appearance). Three leading studies on this topic will be introduced in this symposium.

Firstly, Dr. Conway will introduce recent studies on cortical structure and functionality for the processing of color and related high-level visual features in primate cortex. Secondly, Dr. Tanigawa will introduce their recent study on the neural structure of color-vision processing mechanisms in early visual cortex. Finally, Dr. Hiramatsu will introduce the recent studies on the polymorphism of color vision in primates using genetical and behavioral approach.

## On the Role of Color in High-level Vision

**Bevil Conway**

National Institutes of Health

## 

What is color for and how are color operations implemented in the brain? I will take up this question, drawing upon neurophysiological recordings in macaque monkeys, fMRI in humans and monkeys, psychophysics, and color-naming in a non-industrialized Amazonian culture. My talk will have three parts. First, I will discuss results showing that the neural implementation of color depends on a multi-stage network that gives rise to a uniform representation of color space within a mid-level stage in visual processing. Second, I will describe work suggesting that color is decoded by a series of stages within higher-order cortex, including inferior temporal cortex and prefrontal cortex (PFC). In a surprising twist, these discoveries reveal a general principle for the organization and operation of inferior temporal cortex and provide evidence for a stimulus-driven functional organization of PFC. Finally, I will describe two recent discoveries prompted by our neurobiological discoveries: a new interaction of color and face perception, which suggests that color evolved to play an important role in non-verbal social communication; and a universal pattern in color naming that reflects the color statistics of those parts of the world that we especially care about (objects). Together, the work supports the provocative idea that basic color categories are an emergent property arising from the needs we place on the brain (including object recognition and the assignment of object valence), rather than a constraint determined by color encoding.

## Hue Maps of DKL Space at Columnar Resolution in Macaque Early Visual Cortex

**Hisashi Tanigawa**

Zhejiang University

## 

It is a fundamental question about color vision how cone signals are transformed into perceptual colors in the cortex. Previous studies revealed functional structures for color processing in the early visual cortex, such as CO blobs in V1, CO thin stripes in V2, and color-sensitive domains in V4, and these structures are thought to play an important role in the transformation of cone signals. However, it is not known how hue selectivities based on cone opponency is organized as spatial arrangement in early visual cortex. Here we performed optical imaging in macaque V1, V2, and V4 to examine distribution of domains selective to individual hues of Derrington-Krauskopf-Lennie (DKL) color space that is based on the cone-opponent axes of the lateral geniculate nucleus (LGN). We presented visual stimuli with isoluminant color/gray or color/color stripes: the hue of color stripes was chosen from eight evenly spaced directions in an isoluminant plane of the DKL color space, in which four of them were along the L-M and S-(L-M) cardinal axes. In this talk, I will first introduce our past results of optical imaging in V4 and then show recent results of imaging in early visual cortex using the DKL color space.

## Polymorphic Nature of Color Vision in Primates

**Chihiro Hiramatsu**

Kyushu University

## 

Although mammals generally have dichromatic color vision, primates have uniquely evolved trichromatic vision. However, not all primates possess uniform trichromatic vision, and diverse color vision is ubiquitous in many Neotropical primates and human populations owing to polymorphisms of red-green visual pigment genes. The biological significance of polymorphic color vision and how it influences differences beyond perception are not fully understood. In this talk, I will present how color vision is polymorphic at genetic and perceptual levels, and how these traits affect behavior and even aesthetic impression. Then, I would like to discuss the potential influences of experience and social interaction, which may modify conscious perception of colors.

## Symposium 1-4 (July 29, 2019)

## Modeling Approaches to Visual Circuit Function, Pathology, and Regeneration

**Organizer: Katsunori Kitano**

Ritsumeikan University

## 

The main topic of this symposium is to show how a combination of highly quantitative measurements and mathematical modeling can lead to insights into higher order visual processing in the retina, retinal rhythmogenesis, and the mechanisms that underlie the re-establishment of retinal connections. In the first talk, Dr Tachibana will describe a novel mechanism through which eye movements can dramatically change the group signaling properties of the ganglion cell network, altering its informational content. Second, Dr. Kitano will present a mathematical model for an unexpected oscillatory activity in degenerating retina. The model suggests a source for the abnormal oscillations, and may allow us to devise therapeutic interventions. Finally, Dr Sher will present a quantitative analysis of how connectivity is reestablished in the adult outer retina follow the removal of a subset of photoreceptor (rod and cone) targets.

## Rapid and Coordinated Processing of Global Motion Images by Local Clusters of Retinal Ganglion Cells

**Masao Tachibana**

Ritsumeikan University

## 

Our visually perceived world is stable, irrespective of incessant motion of the retinal image due to the movements of eyes, head, and body. Accumulating evidence indicates that the central nervous system may play a key role for stabilization of the visual world. Fixational and saccadic eye movements cause jitter and rapid motion of the whole retinal image, respectively. However, it is not yet evident how the retina processes visual information during eye movements. Furthermore, it is not clear whether multiple subtypes of retinal ganglion cells (GCs) send visual information independently or cooperatively. We performed multi-electrode recordings and whole-cell clamp recordings from ganglion cells (GCs) of the retina isolated from goldfish. GCs were physiologically classified into six subtypes (Fast/Medium/Slow, transient/sustained) based on the temporal profile of the receptive field (RF) estimated by reverse correlation method. We found that the jitter motion of a global random-dot background induced elongation and sensitization of the spatiotemporal RF of the Fast-transient GC (Ft GC). The following rapid global motion induced synchronous firing among local Ft GCs and cooperative firing with precise latencies among adjacent specific GC subtypes. Thus, global motion images that simulated fixational and saccadic eye movements were processed in a coordinated manner by local clusters of specific GCs. Stimulus conditions (duration, area, velocity, and direction of motion) that altered the properties of the receptive field (RF) were consistent with the characteristics of *in vivo* goldfish eye movements. The wide-range lateral interaction, possibly mediated by electrical and GABAergic synapses, contributed to the RF alterations. These results indicate that the RF properties of retinal GCs in a natural environment are substantially different from those under simplified experimental conditions. Processing of global motion images is already started in the retina and may facilitate visual information processing in the brain.

## Normal and Pathological States Generated by Dynamical Properties of the Retinal Circuit

**Katsunori Kitano**

Ritsumeikan University

## 

The retina numerous subtypes of neurons each of which, when embedded in a circuit, exhibits different dynamical properties. These dynamical properties can influence the output of the retina under normal conditions, and may also play a role in establishing aberrant rhythms under pathological conditions. Indeed, an understanding the dynamical properties of pathological states may help us to understand dynamic neural mechanisms under more normal conditions. Compared to normal adult retina which lacks oscillatory activity, the retina of the rd1 (retinal degeneration 1) mouse is known to exhibit spontaneous, low frequency (<10 Hz), oscillations. Two potential mechanisms for the spontaneous oscillation have been proposed. One mechanism involves the properties of a gap junction network between cone bipolar cells (BCs) and AII amacrine cells (AII ACs) and between AII-ACs (Trenholm et al., 2012), whereas in the other, the oscillations arise from the intrinsic properties of AII-ACs (Choi et al., 2014). We studied the mechanism of spontaneous oscillation using a computational model of the AII-AC, BC, and GC network. In particular, to solve the paradoxical phenomenon mentioned above, we incorporated a ribbon synapse model at the BC-GC synapse. Even when bipolar cells are held in the depolarized state, neurotransmitter release was not always enhanced because of short term depression (Tsodyks and Markram, 1997). If we assume upregulation of the synapses in the inner plexiform layer of the rd1 retina (Dagar et al., 2014), our model could reproduce both the normal and abnormal neural states in the absence of light stimuli: no response in the normal retina and spontaneous rhythmic activity in the abnormal retina.

## Restoration of Selective Connectivity in Adult Mammalian Retina

**Alexander Sher**

University of California, Santa Cruz

## 

Specificity of synapses between neurons of different types is essential for the proper function of the central nervous system. While we have learned much about formation of these synapses during development, the degree to which adult CNS can reestablish specific connections following injury or disease remains largely unknown. I will show that specific synaptic connections within the adult mammalian retina can be reestablished after neural injury. We used selective laser photocoagulation to ablate small patches of photoreceptors in-vivo in adult rabbits, ground squirrels, and mice. Functional and structural changes in the retina at different time points after the ablation were probed via electrophysiology and immunostaining accompanied by confocal imaging. We found that deafferented rod bipolar cells located within the region where photoreceptors were ablated restructure their dendrites. New dendritic processes extend towards surrounding healthy photoreceptors and establish new functional synapses with them. To test if specific connectivity can be reestablished, we observed restructuring of deafferented S-cone bipolar cells that synapse exclusively with S-cone photoreceptors in the healthy retina. We discovered that deafferented S cone bipolar cells extend their dendrites in random directions within the outer plexiform layer. If the extended dendrite encounters a healthy S-cone, it forms a synapse with it. At the same time, it passes M-cone photoreceptors without making synapses. Finally, we used transgenic mice to investigate molecular mechanisms behind the observed restructuring. Our results indicate that the adult mammalian retina retains the ability to make new specific synapses leading to reestablishment of correct neural connectivity.

## Symposium 2-1 (July 30, 2019)

## On the Border of Implicit and Explicit Processing

**Organizer: Shao-Min (Sean) Hung**

California Institute of Technology

## 

Implicit processing plays an important role in maintaining visual functions. After all, at a given moment, our phenomenal experience is inherently limited by various factors, including attention, working memory, etc. In the current proposal, we will tackle major questions in the field and challenge intuitions on implicit/unconscious processing. These questions include the fundamental relation between attention and consciousness, using the level of visual processing as a delineation of explicit and implicit processing, and how implicit decision making perturbs the explicit sense of agency.

Naotsugu Tsuchiya will show recent findings on how attention tracks suppressed stimulus under binocular rivalry. Shao-Min Hung will provide evidence from unconscious language processing, substantiating high-level implicit processing. Daw-An Wu further discusses how TMS alters our attribution of motor decision making.

These topics will be integrated by Shinsuke Shimojo, providing an overall view of the current challenges and advances in the field, including “postdiction.” Some of these challenges can be better dealt with once we are equipped with more suitable views on implicit processing, such as a dynamic interaction among visual items across time, utilizing both predictive and postdictive factors.

## Implicit Processes are Dynamic and Interactive

**Shinsuke Shimojo**

California Institute of Technology

## 

Can the implicit level of mind execute only simple sensory/cognitive functions? And is the bottleneck to consciousness single, or multi-gated? These questions are elusive, especially considering examples such as implicit semantic priming, and implicit stroop effect (Hung talk in this symposium). I will aim for taxonomy and integration of related findings including my own, to have a clearer overview. First, there are multiple definitions of implicit processing on top of “subliminal”, as exemplified in causal misattribution in action (Wu talk), and attention vs. consciousness (Tsuchiya talk). Second, the implicit/ explicit distinction will NOT map onto the lower-/higher-levels of cognitive function (Hung talk). Rather, there are multiple gates to consciousness as indicated in the binocular rivalry debate (80s, up to now), and also quick interplays between implicit and explicit processes. Third, the implicit process may be dynamic spreading over time, operating predictively and postdictively. Auditory-visual “rabbit” effect would be a great example where implicit postdictive process leads to a conscious percept (Shimojo talk). The implicit process is also based on separate dynamic sampling frequencies. Some evidence comes from interpersonal bodily and neural synchrony (Shimojo talk), and dependence of perceptual changes upon allocation of attention relying on different temporal frequencies (Tsuchiya talk). Thus all together, we may need to abandon several simplistic ideas of implicit processes, and rather take a more dynamic and interactive view.

## Attention Periodically Samples Competing Stimuli during Binocular Rivalry

**Naotsugu Tsuchiya**

Monash University

## 

The attentional sampling hypothesis suggests that attention rhythmically enhances sensory processing when attending to a single (∼8 Hz), or multiple (∼4 Hz) objects. Here, we investigated whether attention samples sensory representations that are not part of the conscious percept during binocular rivalry. When crossmodally cued toward a conscious image, subsequent changes in consciousness occurred at ∼8 Hz, consistent with the rates of undivided attentional sampling. However, when attention was cued toward the suppressed image, changes in consciousness slowed to ∼3.5 Hz, indicating the division of attention away from the conscious visual image. In the electroencephalogram, we found that at attentional sampling frequencies, the strength of inter-trial phase-coherence over fronto-temporal and parieto-occipital regions correlated with changes in perception. When cues were not task-relevant, these effects disappeared, confirming that perceptual changes were dependent upon the allocation of attention, and that attention can flexibly sample away from a conscious image in a task-dependent manner.

## Language Processing outside the Realm of Consciousness

**Shao-Min (Sean) Hung^1,2^**

^1^California Institute of Technology

^2^Huntington Medical Research Institutes

## 

The concept “Out of sight, out of mind” has been repeatedly challenged by findings that show visual information biases behavior even without reaching consciousness. However, the depth and complexity of unconscious processing remains elusive. To tackle this issue, we examined whether high-level linguistic information, including syntax and semantics, can be processed without consciousness.

Using binocular suppression, we showed that after a visible sentential context, a subsequent syntactically incongruent word broke suppression and reached consciousness earlier. Critically, when the sentential context was suppressed while participants made a lexical decision to the subsequent visible word, faster responses to syntactically incongruent words were obtained. Further control experiments show that (1) the effect could not be explained by simple word-word associations since the effect disappeared when the subliminal words were flipped and (2) the effect occurred independent of accurate localization of the subliminal text.

In another study we utilized a “double Stroop” paradigm where a suppressed colored word served as a prime while participants responded to a subsequent visible Stroop word. In the word-naming task, we showed that word but not color inconsistency slowed down the response time to the target, suggesting that semantic retrieval was prioritized. However, when asked to name the color, the same effect was obtained only after a significant practice effect on the color naming (i.e. reduction of response time) occurred, suggesting a competition of attentional resources between the current conscious task and unconscious stimulus. These findings were later replicated in separate experiments.

Across multiple studies we showed that high-level linguistic information can be processed unconsciously and exert an effect. These findings push the limit of unconscious processing and further show that an interplay between conscious and unconscious processing is crucial for such unconscious effect to occur.

## The Feeling of Volition as a Retrospective Observational Inference

**Daw-An Wu**

California Institute of Technology

## 

We generally assume that intentions and decisions cause our voluntary acts: We form a conscious intention to do something, and then this mental act leads to a bodily act. Neuroscientific research into the timeline of volition faces the challenge of measuring and reconciling events along many unstably related timelines - external, neural and mental. We use motor TMS stimulation to create a reference event, allowing for single-trial temporal order judgements to be meaningful across all the timelines.

We use electromyography (EMG) to monitor the participant’s (e.g.) thumb. 2) TMS is targeted to motor cortex so as to elicit an involuntary thumb movement. 3) The participant is asked to relax, and at a time of their own choosing, to flex their thumb (a voluntary movement). When the EMG detect the initiation of this movement, it triggers the TMS to activate.

In many cases, the participants report that the TMS click and its resulting thumb movement happened prior to their own volition. Some describe it as if the machine was reading their mind, and just as they were about to decide to act, the TMS beat them to it. The way we have set up the system, however, the TMS cannot be triggered until the voluntary muscle movement has physically begun.

The initiation of a voluntary act is not a discrete, early event to which we have direct mental access. Instead, it is a process that continues to consolidate after the initiation of movement. Our perception of our intentions depends not only on neural signals generated at initiation onset, but also on the integration of information gathered later. This may be analogous to the role of re-entrant feedback to visual cortex in visual consciousness. Contrary to the Cartesian assumption that our introspective awareness is direct, our sense of agency is inferred based on predictive and postdictive inferences about its most likely cause.

## Symposium 2-2 (July 30, 2019)

## The Early Development of Face and Body Perception

**Organizers: Jiale Yang^1^ and Yumiko Otsuka^2^**

^1^Department of Life Sciences, University of Tokyo

^2^Department of Humanities and Social Sciences, Ehime University

## 

Human possess remarkable capacities to process face- and body-related signals. Prior studies consistently reported visual sensitivities to face and body at birth (e.g., Filippetti et al., 2013; Johnson et al., 1991). Moreover, culture specific experience shapes the development of the visual system to develop expertise for specific types of faces and bodies (e.g., own-race faces and communicative body gestures). Furthermore, it is well known that the development of face and body perception is at the foundation of more complex perceptual and cognitive abilities, such as learning and social skills. In this symposium, we will present 5 talks focusing on the early development of face and body perception from infancy to childhood by using a broad range of research methods: skin conductance, electroencephalogram (EEG), eye-tracking, and psychophysics measurements.

Xiao will show how experience of face-race determines early development of infants’ social perception, social learning, and stereotype formation. Chien will show that the pervasive own-race face experience shapes the development of fine-grained and efficient face perception across childhood, which further links to biased social development in childhood. Nava examines the development of multisensory integration from early infancy to childhood and its contribution to the development of body representation. Yang will show tactile information facilitates visual processing in infants, and how body representation modulates this multisensory enhancement. Hirai will talk about infants’ perception of body movements and bodily gestures, and its role in social learning.

In sum, this symposium brings together the latest findings regarding face and body perception across various stages of life and in different culture settings. These studies shed insights into the current advances and future directions of the field of early development of face and body perception.

## Biased Early Social Development by Perceptual Experience: Evidence from Mono-Racial Asian Countries

**Naiqi G. Xiao**

Princeton University

## 

Convergent evidence shows that experience shapes early perceptual development. For example, infants who grow up in a mono-racial environment would develop biased perceptual capabilities for own- vs. other-race faces. However, it is unclear regarding the breadth of experiential impacts on early development. To this end, we explored how face-race experience affects early social development.

In three lines of research, we investigated the impacts of face-race experience in Asian countries, where people mostly see Asian faces, but rarely see faces of other-races. Thus, the mono-racial environment in Asian countries provides an ideal tool to examine whether infants’ social development is biased by asymmetrical face-race experience.

We first studied how face-race affects infants’ social perception via their associations of face-races with different emotional signals (happy vs. sad music). With increased age, infants gradually associated own-race faces with happy music, but other-race faces with sad music, which were evident at 9 months of age. To probe how this biased social perception further influences infants’ social interactions, we investigated infants’ social learning behaviors via learning to follow other’s gaze. Seven-month-olds tended to learn from own- but not other-race adults under uncertain situations. Moreover, similar race- based social learning bias was found when infants learned from multiple own- vs. other-race adults: infants formed a stronger stereotype from a group of other-race adults as opposed to a group of own-race adults. Together, these evidence convergently demonstrate social consequences of asymmetrical face-race experience in infancy. These findings stress the broad experiential impacts on early development beyond perceptual domains.

## The Development and Challenges of Becoming a Native Face Expert: Insights from Taiwanese Children

**Sarina Hui-Lin Chien**

China Medical University

## 

People are remarkable at processing faces. In a split second, one can recognize a person’s identity, gender, age, and race. Importantly, such face processing expertise is not equally prominent for all classes of faces; it works the best for faces belonging to one’s own racial group. In this talk, I will highlight the development and challenges of becoming a native face expert based on my recent studies with Taiwanese children. First, despite many cross-cultural studies reported an early emergence of the own-race advantage (ORA) in the first year of life, adult-like proficiency in discriminating own-race faces is not fully manifested until late childhood. Second, although encoding of race is fast and automatic, categorizing racially ambiguous faces is biased and cognitively taxing. Adults and children with racial essentialist beliefs tend to categorize ambiguous bi-racial faces as other race. Third, when do children judge people by their races? We found that a rudimentary race-based social preference emerges in late pre- school years, and the influence of social status becomes increasingly important as children go to elementary school. In sum, the collective findings suggest that our perception of race emerges early in life and continues to develop through childhood. Lastly, the implications for race-based perceptual and social biases and avenues for future research will be discussed.

## Multisensory Contributions to the Development of Body Representation

**Elena Nava**

University of Milan-Bicocca

## 

The representation of the body and the sense of body ownership are the product of complex mechanisms, and adult studies have suggested that a crucial role is played by multisensory interactions of body-related signals, such as vision, touch and proprioception. In my talk I will present a series of studies conducted at different stages of development (from infancy to childhood) that suggest that multisensory cues not only shape body representation but play either a facilitating or constraining role depending on age. In particular, I will show that very early in life, infants are able to extract the amodal invariant that is common across senses (e.g., rhythm, tempo), and this predisposes them to be naturally attracted to redundant multisensory stimuli. Infants can also extract the social component conveyed by multisensory stimuli, as observed in a recent study in which we found that 4 months-old infants show less arousing responses (as indexed through skin conductance response) to slow/affective touches coupled with a female face than to multisensory non-social stimuli (a discriminative-type of touch coupled with seeing houses). Interestingly, I will show that later in development, children lack to integrate the senses, and this prevents them from being susceptible to classical multisensory body illusions, such as the rubber hand illusion. Finally, I will show that sensory experience, such as vision, contributes to the development of multisensory interactions, and that lack of visual input – as in congenital blindness – prevents blind individuals to have a typical body representation.

## The Effect of Tactile-Visual Interactions on Body Representation in Infants

**Jiale Yang**

University of Tokyo

## 

The representation of the body, which is closely related to motor control and self-awareness, relies upon complex multisensory interactions. Humans new born have been observed to perceive their own bodies (Rochat, 2010), and recent studies showed that visual tactile interactions facilitate the body perception in the early months of life (Filippetti et al., 2013; Freier et al., 2016). In the present study, we used the steady-state visually evoked potentials (SSVEP) to investigate the development of tactile-visual cortical interactions underlying body representations in infants. In Experiment 1, twelve 4-month-old and twelve 8-month-old infants watched a visual presentation in which a hand was stroked with a metal tube. To elicit the SSVEP, the video flashed at 7.5 Hz. In the tactile-visual condition the infant’s own hand was also stroked by a tube whilst they watched the movie. In the vision-only condition, no tactile stimulus was applied to the infant’s hand. We found larger SSVEPs in the tactile-visual condition than the vision-only condition in 8-month-old infants, but no difference between the two conditions in the 4-month-olds. In Experiment 2, we presented an inverted video to 8-month-old infants. The enhancement of tactile stimuli on SSVEP was absent in this case, demonstrating that there was some degree of body-specific information was required to drive the tactile enhancements of visual cortical processing seen in Experiment 1. Taken together, our results indicate that tactile influences on visual processing of bodily information develops between 4 and 8 months of age.

## Development of Bodily Movement Perception in Preverbal Infants

**Masahiro Hirai**

Jichi Medical University

## 

Understanding another’s actions or behavior is one of the vital abilities that allows us to live in a dynamic and socially fluid world. In this talk, two aspects of body perception in preverbal infants will be discussed. The first aspect concerns the developmental mechanisms that underlie the perception of bodily movements—particularly the visual phenomenon of “biological motion” (Johansson, 1973), whereby our visual system detects various human actions through point–light motion displays. The second aspect concerns the cognitive mechanisms of the communicative aspect of bodily movement. The theory of natural pedagogy (Csibra & Gergely, 2009) proposes that infants use ostensive signals such as eye contact, infant-directed speech, and contingent responsivity to learn from others. However, the role of bodily gestures such as hand-waving in social learning has been largely ignored. We explored whether four-month-old infants exhibited a preference for horizontal or vertical (control) hand-waving gestures. We also examined whether horizontal hand-waving gestures followed by pointing gestures facilitated the process of object learning in nine-month-old infants. Results showed that four-month-old infants preferred horizontal hand-waving gestures to vertical hand-waving gestures. Further, horizontal hand-waving gestures enhanced identity encoding for cued objects, whereas vertical gestures did not. Based on our series of studies on body perception in preverbal infants, I will discuss the developmental model of body perception and its role in social communication.

## Symposium 2-3 (July 30, 2019)

## Novel Developmental, Metabolic, and Signaling Mechanisms in the Retina

**Organizers: Chieko Koike^1^ and Steven H. DeVries^2^**

^1^Ritsumeikan University

^2^Northwestern University

## 

The retina is a laminarly organized, self-contained, and accessible piece of the central nervous system that performs the task of early visual processing. The ability to isolate piece of the nervous system that remains fully functional allows us to examine intact pathways including those that underlie development, metabolism, and neural circuits. This symposium will present recent progress in our understanding of mammalian retinal function and development in the areas of cell fate determination, regeneration, synaptic function, and hibernation by vision researchers in the United States. In this symposium, Dr Seth Blackshaw will exploit both transcriptomics and cross-species comparisons to identify the pathways that are essential for retinal cell fate determination and regeneration capacity. Dr Wei Li will focus on the thirteen-lined ground squirrel and describe metabolic pathway adaptations that permit the retina to tolerate long periods of time at near freezing temperatures during hibernation. Finally, Dr Steven DeVries will focus on the cone circuitry in the cone-dominant retina of the ground squirrel and describe how parallel processing pathways get their start.

## Seeing in the Cold: Vision and Hibernation

**Wei Li**

National Institutes of Health

## 

The ground squirrel has a cone-dominant retina and hibernates during the winter. We exploit these two unique features to study retinal biology and adaptations during hibernation. In this talk, I will discuss an optic feature of the ground squirrel retina, as well as several forms of adaptation during hibernation in the retina and beyond. By exploring the mechanisms of adaptation in this hibernating species, we hope to shed light on therapeutic tactics for retinal injury and diseases, which are often associated with metabolic stress.

## Building and Rebuilding the Retina: One Cell at a Time

**Seth Blackshaw**

Johns Hopkins University School of Medicine

## 

The retina is an accessible system for identifying the molecular mechanisms that control CNS cell fate specification and is a prime target for regenerative therapies aimed at restoring photoreceptors lost to blinding diseases. I will discuss our recent large-scale single-cell RNA-Seq analysis of multiple vertebrate species that is aimed at identifying gene regulatory networks that drive the acquisition of neuronal and glial identity in the developing retina. I will discuss our identification of transcription factors that control both temporal identity and proliferative quiescence, new tools we and our collaborators have developed to identify core evolutionarily-conserved gene regulatory networks controlling retinal development, and mechanisms controlling injury-induced neurogenic competence in retinal glia.

## Parallel Signal Processing at the Mammalian Cone Photoreceptor Synapse

**Steven H. DeVries**

Northwestern University

## 

The brain has a massively parallel architecture that supports its prodigious computational abilities. In the visual system, parallel neural processing begins at the cone photoreceptor synapse. At this synapse, an individual cone signals to ∼12 anatomically distinct bipolar cell types that comprise two main classes, On and Off, each consisting of about 6 types. To better understand the first steps in parallel visual signaling, we record in voltage clamp from synaptically connected pairs of cones and identified Off bipolar cells in slices from the cone-dominant ground squirrel retina. At the same time, we capture the detailed structure of the recorded synapse using super-resolution microscopy. Our results show how the molecular architecture of the synapse, including the placement of ribbon transmitter release sites, glutamate transporters, and postsynaptic ionotropic glutamate receptors, can enable the flow of different signals to the different bipolar cell types.

## Symposium 2-4 (July 30, 2019)

## Studying Attention without Relying on Behavior

**Organizers: Yaffa Yeshurun^1^ and Satoshi Shioiri^2^**

^1^University of Haifa

^2^Tohoku University

## 

Attention—the selective processing of relevant information at the expense of irrelevant information— has been subject to scientific inquiry for over a century. One fundamental challenge to the study of attention is that most of our current knowledge was established using paradigms that depend on assumptions regarding the fate of unattended information, or rely in some other way on properties of the participants’ responses (e.g., accuracy or response time). Yet, the assumptions on which these paradigms are based may not always hold, and in general participants’ response can be influenced by many other factors than attention allocation, including response history, biases, higher-level strategies, experience, and so on. Moreover, response time, which is likely the most prevalent measurement in attention studies, is also linked to motor preparation, not just perception. Fortunately, several recent studies were set out to study attention with novel and exciting methodologies that do not rely on the participant’s response, and therefore provide a more objective measure of attentional deployment. Some of these novel methodologies rely on measurements of brain activity (e.g., SSVP, ERP) instead of accuracy or response time, while other methodologies rely on pupil size or eye movements. The four presentations included in this symposium illustrate how such methodologies can be utilized to overcome obstacles that prevail with more traditional paradigms.

## The Characteristics of the Attentional Window When Measured with the Pupillary Response to Light

**Yaffa Yeshurun**

University of Haifa

## 

This study explored the spatial distribution of attention with a measurement that is independent of performance - the pupillary light response (PLR), thereby avoiding various obstacles and biases involved in more traditional measurements of spatial covert attention. Previous studies demonstrated that when covert attention is deployed to a bright area the pupil contracts relative to when attention is deployed to a dark area, even though display luminance levels are identical and central fixation is maintained. We used these attentional modulations of the PLR to assess the spread of attention. Specifically, we examined the minimal size of the attentional window and how it varies as a function of target eccentricity and the nature of other non-target stimuli (i.e., distractors). We found that when the target was surrounded by neutral task-irrelevant disks (i.e., bright/dark disks that did not include response-competing information) the attentional window had a diameter of about 2°. However, when the disks included competing information this size could be further reduced. Interestingly, the size of the attentional doesn’t seem to vary as a function of eccentricity, but it is affected by stimuli size. Finally, we also examined whether the spatial spread of attention is influenced by perceptual load. Load levels were manipulated by the degree of stimuli heterogeneity or task complexity. We found that the size of the attentional window was larger when load levels were low than when load levels were high. These findings demonstrate the flexibility and constraints of spatial covert attention.

## Differences in Attention Modulations Measured by Steady-State Visual-Evoked Potentials and by Behaviors

**Satoshi Shioiri**

Research Institute of Electrical Communication, Tohoku University

## 

One of well-established methods to investigate spatial attention of the human visual system is to ask subjects to attend on a location intentionally (endogenous attention). The modulation in attention has been observed by subjective method such as reaction time and detection rate, and objective method such as electroencephalogram (EEG), fMRI or others. We have been comparing several aspects of spatial attention between behavioral measures and EEG measures, focusing on steady state visual evoked potential (SSVEP). Steady state visual evoked potential (SSVEP) is a technique to realize measurement of attentional effect at unattended locations with stimuli tagged by temporal frequency. We have succeeded to measure spatial and temporal characteristics of visual attention using SSVEP. The measurements showed both similarity and differences between the behavioral and EEG measurements and also between different measures of EEG measures. The EEG measures we compares were amplitude of SSVEP and phase of SSVEP as well as event-related potential (ERP). Time course of spatial attention shift estimated by detection rate is similar more to that by SSVEP phase than others, spatial spread of attention is similar to P3 of ERP but very different of either of SSVEP amplitude or SSVEP phase. Measurements of object-based attention showed similar object effect between SSVEP and reaction time. These results suggest that attention modulation is not at a single site of the visual process, but perhaps at multiple processes. Different effects of attention at different processes may be related to different role of attention at different processes.

## Unified Audio-Visual Spatial Attention Revealed by Pupillary Light Response

**Hsin-I Liao**

NTT Communication Science Laboratories

## 

Recent evidence shows that pupillary light response (PLR) reflects not only the physical light input to the retina, but also the mind’s eye, i.e., where covert visual attention is directed to (see review in Mathôt & Van der Stigchel, 2015). While visual and auditory systems rely on different peripheral mechanisms to represent locations of distal stimuli in the environment, it remains unclear how the spatial representations of the visual and auditory objects are formed in the brain, and how attention plays a role there. Do audio- and visual-spatial attention share the common mechanism or not? To investigate the issue, we examined whether PLR also reflects spatial attention to auditory object. In series of studies, participants paid attention to an auditory object, which was defined by a spatial (e.g., sounds presented in the left or right ear) or non-spatial (e.g., voices from a male or female talker) cue. Results showed that PLR reflected the focus of spatial attention regardless of whether the auditory object was defined by the spatial or non-spatial cue. Furthermore, the amount of spatial attention induced PLR was modulated by the reliability of the spatial information of the auditory object. Cognitive effort (e.g., task difficulty) or physical gaze position could not explain the result. Taken together, the overall results indicate that PLR reveals not only the focus of covert visual attention but also that of auditory attention. Auditory objects share the common space representation associated with visual spatial attention.

## Estimation of Attentional Location Based on the Measurement of Unconscious Eye Movements.

**Hirohiko Kaneko^1^ and Kei Kanari^2^**

^1^Tokyo Institute of Technology

^2^Tamagawa University

## 

Eye and attentional locations are closely related but they are not always the same. Although many eye tracking systems have been developed and used to roughly estimate the location of attention in the scene, but attention tracking system to accurately estimate the location of attention has not been developed yet. In our series of studies, we found that the relationships between the characteristics of eye movements and stimulus properties can be used to estimate attentional location. One of the examples is the relationship between optokinetic nystagmus (OKN) and motion in attention area. We presented two areas with different directions of motion arranged on the left and right, top and bottom, or center and surrounding (concentric) areas in the display. Observers kept their attention to one of the areas by an attention task, which was to count targets appearing on the area. The results indicated that attention enhanced the gain and frequency of OKN corresponding to the attended motion. Another example is small vergence eye movements that occurs when paying attention to an approaching or receding object while fixating a stationary object. The magnitude of the eye movements when paying attention to a certain area was smaller than those when directing eyes to the area, but the relationships between the characteristics of eye movements and stimulus were the same in both cases. Using these relationships, it is possible to determine the attentional location in the visual scene containing objects with various depth and motion. We also mention some applications of the present method for estimating attentional location based on the measurement of unconscious eye movements.

## Symposium 3-1 (July 31, 2019)

## Neural Oscillations and Behavioral Oscillations

**Organizers: Kaoru Amano^1^ and Rufin VanRullen^2^**

^1^Center for Information and Neural Networks (CiNet), National Institute of Information and Communications Technology (NICT)

^2^Centre de Recherche Cerveau et Cognition (CerCo), CNRS

## 

Neural oscillations, such as delta (0.5–4 Hz), theta (4–8 Hz), alpha (8–13 Hz), and gamma (30–100 Hz), are widespread across cortical areas and are related to feature binding, neuronal communication, and memory. Accumulating evidence suggests that alpha oscillations correlate with various aspects of visual processing. Typically, the amplitude of intrinsic alpha oscillation is predictive of the performance on a visual or memory tasks, while the frequency of intrinsic occipital alpha oscillations is reflected in temporal properties of visual perception. Other lines of evidence suggest that behavioral performance such as detection thresholds oscillates at the theta or alpha frequencies. While the connection between neural oscillations and behavior seems to be tight, the underlying mechanisms of these phenomena are not fully understood.

In this symposium, five researchers will present their recent studies on neuronal and/or behavioral oscillations and will discuss the possible functional roles of these oscillations. Dr. Amano will show the causal relationship between intrinsic alpha oscillations and a visual illusion called the motion-induced spatial conflict, possibly suggesting cyclic processing at the frequency of alpha oscillations. Dr. Nakayama will report discretized motion perception at 4–8 Hz, which may reflect the slow attentional process. Dr. Ding will present about the relation of temporal attention to sensorimotor processes. Dr Luo will present the evidence suggesting the causal role of temporally ordered reactivations in mediating sequence memory. Finally, Dr. VanRullen will present perceptual echoes originating from alpha oscillations and their relation to predictive coding.

## Illusory Jitter Perceived at the Frequency of Alpha Oscillations

**Kaoru Amano**

Center for Information and Neural Networks (CiNet), National Institute of Information and Communications Technology (NICT)

## 

Although accumulating evidence suggests that alpha oscillations correlate with various aspects of visual processing, the number of studies proving their causal contribution to visual perception is limited. Here we report that illusory visual vibrations are consciously experienced at the frequency of intrinsic alpha oscillations. We employed an illusory jitter perception termed the motion-induced spatial conflict that originates from the cyclic interaction between motion and shape processing. Comparison between the perceived frequency of illusory jitter and the peak alpha frequency (PAF) measured using magnetoencephalography (MEG) revealed that the inter- and intra-participant variations of the PAF are mirrored by an illusory jitter perception. More crucially, psychophysical and MEG measurements during amplitude-modulated current stimulation showed that the PAF can be artificially manipulated, which results in a corresponding change in the perceived jitter frequency. These results suggest the causal contribution of neural oscillations at the alpha frequency in creating temporal characteristics of visual perception. Our results suggest that cortical areas, dorsal and ventral visual areas, in this case, are interacting at the frequency of alpha oscillations. Possible neuroanatomical basis of the inter-individual differences in the PAF and the peak alpha power (PAP) will also be discussed.

## Temporal Continuity of Vision and Periodic Feature Binding

**Ryohei Nakayama^1,2^**

^1^The University of Sydney

^2^Center for Information and Neural Networks (CiNet), National Institute of Information and Communications Technology (NICT)

## 

Psychophysical and physiological evidence reveal that sensory information is processed periodically despite the subjective continuity of perception over time. How does the visual system accomplish the subjectively smooth transitions across perceptual moments? To address this issue, we analyzed a novel illusion: a continuously moving Gabor pattern appears temporally discrete when its spatial window moves over a carrier grating that remains stationary or drifts in the opposite direction. This discretization depends on the speed difference between window and grating, but the apparent rhythm is constant at 4–8 Hz regardless of the stimulus speeds (Nakayama, Motoyoshi & Sato, 2018). In the light of recent studies reporting the theta-rhythmic function of attention, we hypothesize that different dimensional features of window and grating whose positional estimates are biased by opposite directional motions would be bound in a periodic manner. Accordingly, we found that temporal binding of visual features is performed periodically at ∼8 Hz between spatially separated locations, while depending on pre-stimulus neural oscillatory phases locked by voluntary action (Nakayama & Motoyoshi, under review). (As one would expect from previous studies on pre-attentive binding, such a periodicity was not observed between superimposed locations.) Therefore, periodic attention serves to bind sensory information across different dimensions and locations to produce unified perception, subserved by neural oscillations in synchrony with action. The present combined results imply that slow attentional process can cause the discretized perception, while perhaps fast automatic process may underlie the temporal continuity of vision.

## Temporal Attention Requires Sensorimotor Mechanisms During Visual and Auditory Processing

**Nai Ding**

Zhejiang University

## 

We live in a dynamic world and sensory information comes rapidly and overwhelmingly. Temporal attention provides a mechanism to preferentially process time moments that carry more critical sensory information. It has been proposed that the motor system is critical to implement temporal attention and here I will present recent evidence that temporal attention involves sensorimotor processes. It is shown that blinks and related eyelid activity are synchronized to temporal attention. When processing a visual sequence, a task is used to force the participants to attend to specific time moments. We find that blinks are suppressed at the attended time moment and the blink rate rebounds about 700 ms after the attended moment. This phenomenon can be interpreted as a form of active sensing that actively avoids the loss of important visual information caused by blinks. Nevertheless, further evidence from the auditory modality shows that attention-related eyelid activity is a more general intrinsic property of the brain. It is shown that blinks are similarly modulated by temporal attention in auditory tasks. Even when the eyes are closed, eyelid activity measured by EOG is still suppressed at the attended time moment. Furthermore, when listening to speech and performing a speech comprehension task that does not explicitly requires temporal attention, eyelid activity is synchronized to spoken sentences. Taken together, these results suggest that the motor cortex is activated when allocating attention in time and activity in motor cortex can be reflected in eyelid activity.

## Serial, Compressed Memory-Related Reactivation in Human Sequence Memory: Neural and Causal Evidence

**Huan Luo**

Peking University

## 

Storing temporal sequences of events in short-term memory (i.e., sequence memory) is fundamental to many cognitive functions. However, it is unknown how the sequence order information is maintained and represented in human subjects. I will present two studies in the lab to address the question. First, using electroencephalography (EEG) recordings in combination with a temporal response function (TRF) approach, we probed the item-specific reactivation activities in the delay period when subjects held a sequence of items in working memory. We demonstrate that serially remembered items are successively reactivated, in a backward and time-compressed manner. Moreover, this fast-backward replay is strongly associated with recency effect, a typical behavioral index in sequence memory, thus supporting the essential link between the item-by-item replay and behavior. Based on the neural findings, we further developed a “behavioral temporal interference” approach to manipulate the item-specific reactivations in retention, aiming to disrupt and change the subsequent sequence memory behavior in recalling. Our results show that the temporal manipulation on the replay patterns – synchronization and order reversal –successfully alters the serial position effect, as typically revealed in sequence memory behavior. Taken together, the results constitute converging evidence supporting the causal role of temporally ordered reactivations in mediating sequence memory. We also provide a promising, efficient approach to rapidly manipulate the temporal structure of multiple items held in working memory.

## Alpha Oscillations, Travelling Waves and Predictive Coding

**Rufin VanRullen**

Centre de Recherche Cerveau et Cognition, CNRS

## 

Alpha oscillations are not strictly spontaneous, like an idling rhythm, but can also respond to visual stimulation, giving rise to perceptual “echoes” of the stimulation sequence. These echoes propagate across the visual and cortical space with specific and robust phase relations. In other words, the alpha perceptual cycles are actually travelling waves. The direction of these waves depends on the state of the system: feed-forward during visual processing, top-down in the absence of inputs. I will tentatively relate these alpha-band echoes and waves to back-and-forth communication signals within a predictive coding system.

## Symposium 3-2 (July 31, 2019)

## Science of Facial Attractiveness

**Organizer: Tomohiro Ishizu**

University College London

## 

Visual attraction pervades our daily lives. It influences and guides our moods, behaviours, and decisions. Scientists apply psychological and cognitive neuroscientific methods to disentangle the seemingly complex attractiveness evaluation, and the rigorous scientific findings are growing quickly. Facial attractiveness has been a central interest in the science of attraction. In this symposium, we present new insights on attractiveness judgments with a focus on face perception from a wide range of methods including behavioural testing, computational modelling, neuroimaging, and brain-stimulation. We anticipate that it will engage interests of the APCV attendance and that it will draw a large and lively audience.

Firstly, we show a data-driven mathematical modelling which reveals physical features of a face contributing to attractiveness judgments (Nakamura). We, then, present a study which visualises a ‘mental template’ of attractive faces by applying the reverse correlation technique and deep convolutional neural network (Naito). The first two talks can elucidate physical and measurable features of attractive faces and what contributes to the judgment. Secondly, we show how facial attractiveness judgment can be formed. We present evidence that attractiveness judgment is a dynamic process in which each facial feature (e.g., eyes, nose, hairstyle) is integrated over time to construct a final evaluation (Saegusa). Next, we discuss the brain systems, that are possibly underlying in attractiveness judgment on faces and compare them with non-facial/non-biological stimuli in relation to cortical-subcortical networks (Ishizu). Finally, we demonstrate the ‘causal role’ of those brain sites when judging attractiveness of faces and other visual stimuli with the application of non-invasive brain-stimulation techniques (Cattaneo). Understanding attractiveness evaluations and the impact of visual experiences is an indispensable part of understanding human interaction with the visual world. This symposium, showcasing diverse methods to approach the question, will provide new insights into the studies on attraction and attractiveness.

## Data-Driven Mathematical Modeling of Facial Attractiveness

**Koyo Nakamura^1,2,3^**

^1^Waseda University

^2^Japan Society for the Promotion of Science

^3^Keio Advanced Research Centers

## 

Facial attractiveness can be assessed in a rapid and spontaneous manner, long before we can tell what features make a face attractive. Faces vary in many different ways and many of these variations affect facial attractiveness judgments. In this talk, I will present a series of studies that examine how people judge facial attractiveness from a combination of multiple cues such as facial shape and skin properties. To identify important cues to attractiveness, we first sampled many different East-Asian faces and collected ratings on their attractiveness, then built a data-driven mathematical model for how facial features vary on attractiveness. The results revealed that faces with larger eyes, smaller noses, and brighter skin are judged as more attractive, regardless of the sex of the faces. Furthermore, our model allows for quantitatively manipulating the attractiveness of any arbitrary faces by transforming such facial features, thus helping discover as yet unidentified cues to attractiveness. The attractiveness manipulation technique provides a tool to produce well-controlled East-Asian face stimuli that quantitatively differ in attractiveness, which can be used to elucidate further the visual processes related to attractiveness judgments.

## Transplantation of Taste for Facial Attractiveness of Individuals to Deep Convolutional Neural Network

**Tomoyuki Naito**

Osaka University

## 

Deep convolutional neural network (DCNN) has a lot of attention for its capability of image category classification that is comparable to that of human. A recent study reported that the DCNN obtained hierarchical representations and learn the concept of facial attractiveness. However, it is still unclear whether the judgment mechanisms of the DCNN under the facial attractiveness judgment was similar with humans or not. In this study, we show that from facial attractiveness judgment scores of individuals, DCNN learned the taste of individuals with high accuracy. Then, we reconstructed visual mental template of facial attractiveness of both participants and the DCNN using psychological reverse correlation technique. For all participants and corresponding DCNN, the visual mental template was successfully reconstructed. We confirmed that the mental template of DCNN was significantly correlated with that of the participant who provided the facial attractiveness scores for learning. Our results suggested that the DCNN that learned one’s taste for facial attractiveness reconstructed similar judgments mechanisms with humans in it.

## Judgments of Facial Attractiveness as A Dynamic Combination of Internal/External Parts

**Chihiro Saegusa^1^, Katsumi Watanabe^2,3^, Shinsuke Shimojo^4^ and Janis Intoy^4^**

^1^Kao Corporation

^2^Waseda University

^3^The University of Tokyo

^4^California Institute of Technology

## 

Although the importance of facial attractiveness has been widely researched, how attractiveness of internal/external facial parts and whole interacts in a time course of attractiveness judgment is still unclear. In our research, visual information integration in the facial attractiveness judgment has been investigated in a series of psychological experiments in which presentation of facial images to be evaluated their attractiveness was constrained spatially and/or temporally. Attractiveness evaluation of briefly-presented facial images demonstrated that 1) contribution of the eyes to the whole facial attractiveness judgment remains high even after short exposure duration as 20 milliseconds to the face, while contribution of other facial parts changed over time, and 2) either the gaze of the face is directed to or averted from the evaluator affected the dynamic integration of facial parts information to the judgments of whole facial attractiveness. Different experiments examining the influence of external feature on the perceived facial attractiveness revealed the mutual, but not symmetrical influence between facial attractiveness and hair attractiveness. These findings together suggest the dynamic feature of facial attractiveness judgment where information from internal/external features is integrated over the time while it is affected by social cue such as gaze direction of the face.

## Varieties of Attractiveness and their Brain Responses

**Tomohiro Ishizu**

University College London

## 

Over the past decade, cognitive neuroscience of attractiveness has been maturing and has found that experiencing something as attractive, such as viewing a beautiful face, engages brain’s reward circuit, namely the medial orbitofrontal cortex/ventromedial prefrontal cortex (mOFC/vmPFC) and structures in the ventral striatum (VS). Interestingly, these core regions are thought to be stimulated by attractiveness regardless of their source and to encode a ‘common neural currency’ (Levy & Glimcher, 2012). This is not contradicting to daily experiences: we feel pleasure when we find something attractive. However, assuming that attractiveness is closely related and intertwined to pleasure, it gives rise to the question; the activation within the mOFC/vmPFC and the VS with attractiveness experience may be merely attributed to the pleasurable experience, and it is little to do with attractiveness per se. To address this question, I propose to categorise attractiveness into two types; attractiveness derived from biologically-based stimuli, such as faces, bodies, or nutritious foods (biological attractiveness), and one derived from higher cognitive processes, such as art appreciation or a person with good morality (higher-order attractiveness). The stimuli categorised in the former relate to the fulfilment of biological needs, such as mating, having sex, intake of nutrition (primary rewards), whereas, stimuli of the latter category do not require biological needs and primary rewards. Recent findings and discussions from our lab and others (e.g. Wang et al., 2015; Ishizu & Zeki, 2017) suggest that judgments of both biologically-based attractiveness (i.e. facial attractiveness) and higher-order attractiveness (i.e. moral attractiveness) similarly engage both the mOFC/vmPFC and VS, but that the higher-order attractiveness alone lacks the VS activity. These findings suggest that, even though attractiveness is hardwired to pleasure, it may not be a one-to-one relationship and different types of attractiveness may engage dissociable brain mechanisms.

## The Impact of Brain Stimulation in Modulating Visual Preference for Faces and Paintings

**Zaira Cattaneo**

University of Milano-Bicocca

## 

Neuroimaging evidence has shown that beauty appraisal correlates with activity in a complex network of brain areas involved in sensory processing, reward, decision making, attentional control, and the retrieval of information from memory. In my talk, I aim to present an overview of a series of recent experiments I conducted with non-invasive brain stimulation (both transcranial magnetic and transcranial direct current stimulation) that shed light on the causal role of different brain regions in underpinning aesthetic appreciation for different stimulus categories, ranging from faces to paintings. By informing about whether the activation of a particular cortical site is necessary (vs. epiphenomenal) for an ongoing task, brain stimulation critically adds to the correlational evidence provided by neuroimaging techniques, and may be a promising tool in the field of neuroaesthetics.

## Symposium 3-3 (July 31, 2019)

## Two-Photon Calcium Imaging of Architecture and Computation of Primate Visual Cortex

**Organizers: Ichiro Fujita^1^, Kristina Nielsen^2^ and Ian Nauhaus^3^**

^1^Osaka University

^2^Johns Hopkins University

^3^University of Texas Austin

## 

We aim to bring together researchers working actively on the primate visual system using 2-photon calcium imaging and related techniques. Two-photon imaging is now a standard tool in systems neuroscience. It provides unique advantages for addressing questions at multiple levels of function and structure which are not accessible by other techniques; e.g., high temporal resolution of detecting signals originating from single action potentials and high spatial resolution of determining the position and distribution of individually monitored neurons. These technical merits allowed us to reveal the functional microarchitecture of the visual and other cortices. Thus far, the vast majority of two-photon imaging studies in the mammalian brain have been performed in the rodent. However, the primate is the preferred animal model for linking cells and circuits to more sophisticated sensory, motor, and cognitive functions. This holds true especially for visual functions which has evolved elaborately in primates. Given the merit and potential, there currently is a large push to overcome the technical challenges of performing two-photon imaging in primates. In this symposium, we provide a platform for discussing and sharing the scientific impacts, current technical advancement, and ideas of future directions in this research field. The speakers include researchers from Asia (Fujita, Ohki, Tang) and USA (Nauhaus, Nielsen, Priebe), working on various stages of the visual cortical hierarchy (V1, V2, V4) in macaque and marmoset monkeys. All speakers have agreed to participate in this symposium. The symposium will start with a brief introduction (5 min) by one of the chairs followed by 20-min presentation of the 6 speakers.

## Zooming In on Neural Circuits in Macaque Visual Cortex

**Shiming Tang**

Peking University

## 

My lab focuses on the neural mechanisms of visual object recognition and the development of techniques for neuronal circuit mapping. We have established long-term two-photon imaging in awake monkeys — the first and critical step toward comprehensive circuit mapping — to identify single neuron functions. We have systematically characterized the V1 neuronal responses with unprecedented detail in awake monkeys. We found a large percentage of V1 neurons exhibit complex pattern selectivity beyond orientation tuning, to sparsely responded to natural scenes. This finding suggests an early stage of local complex pattern detection in V1 in the visual object recognition hierarchy. Recently, we performed high resolution dendritic imaging in macaque monkeys, and mapped the fine spatiotemperal organization of excitatory inputs on dendrites of macaque V1 neurons. Our results suggested V1 neurons integrate rich and heterogeneous inputs for complex local pattern detecting. With these modern imaging technologies now functioning in macaque monkeys, we can untangle the neuronal micro-circuits in visual cortex and finally uncover fundamental computational principles in visual information processing.

## Neural Tuning in Superficial V1 as a Function of Scale Invariant Inputs

**Ian Nauhaus**

University of Texas Austin

## 

Recent imaging studies in macaque primary visual cortex (V1) have revealed maps of spatial frequency (SF) preference that systematically align with maps of orientation preference and ocular dominance. Additional V1 maps are predicted under the assumption of “scale invariance”, whereby scale parameters - RF size, SF bandwidth, SF preference – are proportional to one another. However, prior studies show that scale invariance fails for “complex cells”, which make up the majority of our two- photon recordings in layer 2. Given that scale parameters cannot be predicted from SF preference for complex cells, a general model of the V1 architecture is still lacking for superficial V1. Here, we compared maps of SF preference to maps that would be correlated under scale invariance (RF size and SF bandwidth), in addition to maps that would be independent from SF under scale invariance (orientation and phase selectivity). In each case, the data deviated from scale invariance much more strongly than a population of V1 simple cells. Finally, we are fitting a model whereby scale invariant simple cells converge to build for a population of RFs like the ones in our data. In summary, we have provided a more complete description of V1 architecture in L2/3 – the principal output layer - that provides improved constraints to decoding models over basic assumptions of scale invariance.

## Clustering of 3D and 2D Shape Information in Area V4

**Kristina Nielsen**

Johns Hopkins University

## 

long the ventral pathway, image information is converted into object and scene understanding. Area V4, an intermediate stage in this pathway, has previously been shown to represent 2D contour shape. We have recently demonstrated that a substantial fraction of V4 neurons are more responsive to 3D volumetric shape (shape-in-depth) than to 2D shape in the image plane. Here, using 2-photon functional microscopy, we investigate the spatial organization of 3D and 2D shape tuning in V4. Our results demonstrate that neurons with 2D and 3D shape tuning form segregated clusters in V4.

We used realistic shading cues to render simple volumetric shapes (Cs and Vs with cylindrical cross-sections and smoothly curved joints and endcaps), presented at a range of 3D orientations. In addition, we measured responses to the 2D silhouettes of the same stimuli. More precisely, for each 3D stimulus there was a matching 2D stimulus that shared the same 2D contours. While in the 3D case these contours appeared as self-occlusion boundaries of volumetric objects, they appeared as sharp edge boundaries of planar shapes in the 2D case.

3D and 2D stimuli were used to probe the responses of V4 neurons in 2-photon experiments in anesthetized animals, in which neurons were labeled with the calcium indicator Oregon Green BAP-TA-1AM. In each imaging region, we consistently observed strong local clustering of 3D- and 2D-responsive neurons in separate patches on the order of several hundred microns. At the same time, neighboring 3D and 2D patches were most responsive to congruent 3D and 2D shapes. These results suggest that derivation of 3D volumetric shape from 2D image information is a major constraint on micro-organization in area V4.

## Functional Architecture for Processing Visual Texture in V4

**Ichiro Fujita**

Osaka University

## 

Visual texture is an important clue for recognizing objects. Representing a texture requires computation of a collection of products between V1-like filter outputs across scales, orientations, and positions (i.e., higher-order image statistics). Previous studies using static texture stimuli demonstrate that neuronal selectivity for higher-order image statistics is not evident in V1, and gradually develops in mid-level ventral visual areas, V2 and V4. Here, we aimed to extend our understanding on the processing of texture in V4 by examining the functional architecture of texture representation. We recorded activity of V4 neurons with the aid of in vivo 2-photon calcium imaging in two immobilized macaque monkeys under opiate-analgesia. We presented naturalistic movie stimuli, in which higher-order image statistics dynamically changed. We evaluated contributions of higher-order image statistics on V4 responses by using a general encoding-model approach in which regularization and cross-validation were implemented. Consistent with the previous studies, V4 neurons overall preferred higher-order image statistics over low-level image statistics (i.e., V1-like filter outputs), whereasV1 neurons recorded from the same animals preferred spectral stimulus features such as orientation and spatial frequency. The 16 different sites we examined in V4 exhibited a variety in preference for higher-order image statistics. Most sites contained many neurons preferring higher-order image statistics. Some other sites were abundant with neurons preferring low-level image statistics, and thus were indistinguishable from V1 sites. From these results, we conclude that neurons representing higher-order image statistics are locally clustered in V4, and the cluster size is between several hundreds of micrometers (size of a recording site) and several millimeters (distance between recording sites). Together with known functional structures in V4 for color and orientation with this scale, we suggest that V4 consists of mosaic-like compartments (∼mm size) each responsible for a specific visual feature such as color, orientation, and texture.

## Multiscale Calcium Imaging of the Visual Cortex in Marmoset Monkeys

**Kenichi Ohki**

The University of Tokyo

## 

Primate neocortex analyzes visual scenes with a hierarchical neuronal network. To understand how such network interactively process visual scenes, we developed a method to monitor neuronal activity at multiple spatial scales. Based on Tet-off system (Sadakane et al., 2015), we first designed new AAV2/9 vectors which contain TLoop system (Cetin and Callaway, 2014) or two in-tandem of GCaMP, and successfully increased the level of GCaMP expression in a large volume of the marmoset neocortex.

Using the improved vectors, we first performed wide-field 1-photon calcium imaging. In addition to orientation map in the primary visual cortex (V1), we found that full-field luminance increment and decrement evoked regular patches of responses in V1 (luminance polarity map). We then studied cellular activity using 2-photon imaging. In addition to orientation-selective cells, we found “non-tuned cells” that were responsive to drifting gratings but not selective for orientation, and “non-responsive cells”. Interestingly, non-tuned cells selectively responded to the luminance increment, whereas non-responsive cells selectively responded to the luminance decrement.

The present method is applicable to higher visual areas beyond V1. Smooth neocortex of marmosets allowed us to monitor neuronal activity in multiple brain areas spanning occipital to parietal cortices These results demonstrate usefulness of the marmoset brain to study the visual cortical network.

## The Functional Organization of Area MT Neurons Revealed by 2-Photon

**Nicholas Priebe, Jagruti Pattadkal and Boris Zemelman**

University of Texas at Austin

## 

Area MT contains neurons that are exquisitely sensitive to visual motion and, based on extracellular recordings, is functionally organized for direction. To uncover the fine-scale functional architecture of area MT and assay the selectivity of inhibitory neurons, we used the marmoset (*Callithrix jacchus*). These primates have lissencephalic brains in which we have access to activity of large neuronal populations. We used 2-photon microscopy to record from several hundred neurons at single-cell resolution over a 1 mm^2^ region of area MT in awake marmosets. GCaMP expression was induced by injecting AAV constructs with promoters that provided specific expression in interneurons within area MT. GCaMP signals from inhibitory neurons revealed similar degrees of motion selectivity as that found from excitatory neurons (median DSI = 0.38, n = 301 cells). Nearby neurons tend to share direction preference, forming a map of direction preference with a period of approximately 300 microns. Finally, we found that the degree of orientation selectivity in MT neurons is weaker (median OSI = 0.13) than direction selectivity. In sum, we have revealed the fine functional organization of area MT using 2-photon microscopy in awake marmosets and have demonstrated that MT inhibitory neurons are as direction selective as their excitatory neuron counterparts.

## Symposium 4-1 (August 1, 2019)

## Color Vision in Naturalistic Objects and Environments

**Organizer: Yoko Mizokami**

Chiba University

## 

Color perception in real life is adjustable and stable. Color adaptation and color constancy are good examples of this flexibility of color vision, but recent researches have revealed much more complexity and the influence of various factors such as color distribution in an environment, naturalistic change in illumination, the property of material, cue integration from different visual dimensions, memory and learning, the recognition of naturalness of objects and scenes.

This symposium focuses on the property of our color vision for real or realistic objects and environment and also discuss how we should test those properties, by introducing the latest researches of five researchers working on complex color vision in different viewpoints, but their interests are overlapping each other.

Dr. Webster will talk about environmental influences on color appearance through his extensive work related to adaptation to various environments. Dr. Olkkonen will talk about how learning and memory affect our color perception. Dr. Sarrela will talk about how cue integration from different visual dimensions helps color and material perception. Dr. Nagai will talk about the effects of specular reflection components on color constancy using the various combination of complex stimuli generated by computer graphics. Dr. Mizokami will talk about color and material perception under different lighting conditions in a real environment.

## Blue and Yellow in the World and the Brain

**Michael A. Webster**

University of Nevada

## 

Blue-yellow variations are a prominent property of the natural environment. For example, different phases of daylight vary along a blue-yellow axis, and the gamut of colors in many natural scenes varies from blue sky to predominantly yellowish or brown terrain. These stimulus biases may have shaped many aspects of human color vision, including chromatic sensitivity and color appearance. Sensitivity can be weaker along the blue-yellow axis, and this may reflect adaptation to the stronger blue-yellow contrasts in scenes. This bias is also manifest in the scaling chosen for many perceptually uniform color spaces. Similarly in color appearance, blue and yellow can seem more pure or “unique,” even though these hues do not clearly reflect special states in the underlying neural code for color. There are also important differences between blue and yellow percepts which suggest they are not treated as two poles of a common underlying dimension. In particular, bluish tints are more likely to be attributed to the illuminant, while yellowish tints are more likely to be associated with surface color. These asymmetries are largely restricted to the blue-yellow axis, and may again be shaped by high-level inferences about the chromatic properties of the world.

## Learning and Memory in Color Perception

**Maria Olkkonen**

Durham University

## 

We often have the experience of perceiving the colors and materials of objects in our environment effortlessly. But estimating the material properties of objects is in fact a computationally hard problem for the visual system, because the light signal that reaches our eyes from surfaces in our environment depends not only on the reflectance of the surface, but also on the illumination impinging on the surface. Statistical regularities about surfaces and illuminants, learned through interacting with our environment and through social communication, may contribute to our ability to compensate for changes in illumination when estimating object color. In this talk, I will link human color constancy to a probabilistic framework of perceptual estimation, and will give an overview of experiments testing predictions from this framework. The results so far suggest that human color constancy can be modeled in the probabilistic framework, but more work remains to be done to uncover the computational mechanisms of color constancy in natural scenes. I will end by discussing ongoing research to push the study of color constancy to more realistic scenes and tasks.

## Cue Integration in Color and Material Perception

**Toni Saarela**

University of Helsinki

## 

Visual features do not occur in isolation, and surfaces and materials in our visual environment differ from each other in several respects: in color, lightness, glossiness, and texture, for example. Integrating information from several such sources, or “cues”, is a fundamental property of the visual system and can enable us to perform better in several visual tasks. Visual cue integration can occur “across space”, as when integrating color information from distinct spatial locations to estimate the body color of an object. It can also happen “across dimensions”, as when integrating different types of spatially overlapping cues, for example color and glossiness when discriminating or identifying materials. I will present examples of both types of integration. First, as an example of spatial integration, I will discuss results from experiments characterizing the sampling of hues when estimating the mean hue of an ensemble of colors. Color discrimination was measured for a range of stimulus sizes, and stimulus colors where perturbed by noise. Through modeling, we derived estimates of the observers’ ability to sample the stimulus when discriminating mean color. The estimates far exceed those previously reported in the literature based on a more limited set of experimental conditions. To illustrate spatially local integration of cues from different visual dimensions, I present results from optimal integration color, texture, and glossiness cues when evaluating surface properties. Finally, I will highlight the importance of manipulating the extrinsic uncertainty in a psychophysical task when measuring integration, and will show how mandatory integration of visual cues can also have its downsides, as it can in some cases prevent the selection of individual feature dimensions for further processing.

## Effects of Specular Reflection Components on Color Constancy

**Takehiro Nagai**

Tokyo Institute of Technology

## 

The visual system is considered to employ various heuristics in retinal images which reflect illumination colors in scenes for color constancy. Specular highlight is one of such candidate cues, because they typically reflect spectral components of illumination directly. Although some previous studies reported small improvement of color constancy in scenes with glossy objects, the degree of the improvement largely differed across the studies. We have investigated psychophysically 1) if effectiveness of specular highlights is valid under different stimulus conditions, and 2) which image features explain such highlight effects on color constancy.

The stimulus consisted of a test sphere and many background objects, which had different levels of specular reflectance (SR) and fixed diffuse reflectance. Observers performed achromatic-setting tasks on the test sphere under D65, A, or 25000K illuminant.

First, the color constancy index increased by maximum of 30% from minimum to maximum SR under A. In contrast, SR did not significantly affect color constancy index under 25,000K. These results suggest that the conditions under which specular components contribute to color constancy are limited; the roles of specular components seem just supportive, not very pronounced. Second, when we performed almost the same experiments except that the images of background objects were phase-randomized while keeping luminance-chromaticity histograms unchanged, the improvement of color constancy was dramatically diminished. Also, by removing high-luminance components in specular reflections the improvement of color constancy was completely lost. The high-luminance components look like specular reflections seem to be crucial for the improvement effect in color constancy.

## Color and Material Perception in Real Illuminating Environment

**Yoko Mizokami**

Chiba University

## 

It has been suggested that the specular reflection occurring on a surface of an object would contribute to color and material perception. First, we examined the effect of the surface and specular reflection of objects on color constancy using real vegetables as familiar objects in real space. Observers evaluated stimuli with different glossiness under white and reddish color illumination, and we compared those color appearances. As a result, in the real space, specular reflection hardly affected color constancy, but under the limited view condition, color constancy was a little bit better for the glossy surface than the matte surface. These results suggest that the specular reflection slightly contributes to color constancy under limited conditions. Second, we examined how the color appearance of object surface was influenced by the diffuseness of lighting in real miniature rooms. We used two miniature rooms illuminated by a diffused light and a direct light, respectively. We presented a test sample with sine-wave surface. Both glossy and matte surface materials with five colors were prepared for the samples. Observers judged the color appearance of samples by selecting their corresponding colors. The corresponding color for test samples were similar under both diffused and direct lighting conditions. The color appearance of object surface would be quite stable among the change in material and illumination.

## Symposium 4-2 (August 1, 2019)

## Multi-Dimensional Approach to Understand Anatomical Basis of Visual Functions

**Organizers: Hiromasa Takemura^1^ and Toru Takahata^2^**

^1^CiNet, NIC

^2^Zhejiang University

## 

Although visual system has been widely studied over several last decades, there is one major question remains largely unanswered: how functions of visual system are related to underlying anatomical properties. This symposium features investigators working with cutting edge approach for addressing this question, by using a various type of methods spanning from molecular, microscale to macro-scale level. The symposium will address how anatomical measurements will help to understand disorders, organization, plasticity and evolution of the visual system.

## Understanding Major White Matter Pathways in Visual System: From Neuroimaging to Neuroanatomy

**Hiromasa Takemura^1,2^**

^1^Center for Information and Neural Networks (CiNet), National Institute of Information and Communications Technology

^2^Osaka University

## 

Human and non-human primate visual system is composed of a number of geniculo-cortical and cortico-cortical white matter pathways, which support communication between distinct visual areas. This talk describes recent progress in analyzing these pathways to understand disorders, organization and function of the visual system. First, I will demonstrate the evidence showing retinal ganglion cell disease, Leber’s Hereditary Optic Neuropathy, caused different types of neurobiological change among different part of visual pathways (optic tract and optic radiation) by combining two types of neuroimaging measurements, diffusion MRI (dMRI) and quantitative MRI (qMRI; Mezer et al., 2013). Second, I will describe recent progress in analyzing the vertical occipital fasciculus (VOF; Yeatman et al., 2014) by combining dMRI and anatomical measurements. The VOF is an important white matter tract to understand visual processing streams because it connects dorsal and ventral streams (Takemura et al., 2016). To improve our understanding of this pathway, we first analyzed high-resolution dMRI data obtained from non-human primate brains. The analysis of dMRI data reveals that inter-species similarities of VOF across primate species, but also provides consistency of VOF cortical endpoints among dMRI and previous invasive studies. Furthermore, I will also describe an analysis of data obtained by using polarized light imaging (PLI; Axer et al., 2011), which provide fiber orientation at micrometer resolution. PLI data not only supports the existence of the VOF, but also disentangles current controversies in the visual white matter pathways, such that how much the VOF is distinct from a pathway connecting occipital and inferotemporal cortex. Finally, I will discuss how accurate understanding of white matter pathways help us to understand the organization of extrastriate cortex.

## Across the V1 Orientation Map Long-Range Lateral Inputs onto Local Inhibitory Neurons Sharpen Orientation Tuning of Principal Neurons

**David C. Lyon**

University of California, Irvine

## 

Specific cell types and their connectivity are a key determinant in neural function and selectivity. Visual cortex is among the most complex and detailed brain structures and several recent technological advances have enabled more detailed probing of cell type specific relationships to connectivity and function. Yet, such studies leave many questions unresolved and are largely limited to transgenic mice which lack more complex organization found in higher visual species such as cat and monkey. Of particular interest, is the role of inhibitory neurons in modulating orientation selectivity. Orientation tuning has been shown to improve dramatically when visual stimuli expand beyond the classical receptive field (CRF) into the extraclassical surround (ECS). Moreover, in addition to sharper orientation tuning, firing rate is also reduced, suggesting a role of inhibition. Long-range horizontal projections, which allow for integration across the visual field within V1 and represent visual space corresponding to parts of the ECS, preferentially connect regions, or domains, of neurons with like-orientation preference. Using a novel cell-type specific rabies virus tracing strategy we have shown that a major target of these orientation tuned inputs is local inhibitory neurons (Liu et al., 2013, Curr Biol). We hypothesize that these inputs play a key role in the orientation selectivity of the suppressive effects attributed to the ECS. To test this, we retrogradely delivered light gated opsin, ChR2 or ArchT, to these long-range inputs through our rabies virus technique. We then measured the effects of their light mediated activation or blockade, respectively, on single unit responses to various center-surround visual stimulus conditions. When only a CRF sized stimulus was shown, ChR2 activation simulated surround suppression, reducing firing rate and sharpening orientation tuning. Conversely, ArchT mediated suppression of long range inputs under conditions including the ECS, blocked the effects of suppression and orientation tuning broadened. Because the labeled long range inputs are largely excitatory neurons synapsing onto local inhibitory neurons these results show that interconnectivity between orientation domains play a major role in modulating orientation tuning.

## Possible Parallel Visual Pathways Between the Lateral Pulvinar and V2 Thick/Thin Stripes in Macaques

**Toru Takahata**

Interdisciplinary Institute of Neuroscience and Technology (ZIINT), Zhejiang University

## 

It has been known that primate V2 is subdivided into at least three sub-compartments: thick stripes, thin stripes, and pale stripes, according to their reactivity to cytochrome oxidase (CO) histochemistry. Later, it has been revealed that these histochemical sub-compartments are associated with functional reactivity to distinct types of visual stimuli, such that thick stripes are more responsive to directional movement and depth coding, thin stripes are more responsive to color stimuli, and pale stripes are more responsive to form and orientation of the visual stimuli. Furthermore, these physiological properties are reasonably associated with connectivity from V1, as color-coding neurons in CO blobs preferentially project to thin stripes and neurons in interblobs preferentially project to thick and pale stripes. They are recognized as “parallel visual pathways”: The “P” pathway that goes through geniculate parvocellular layers/V1 CO blobs/V2 thin stripes and the “M” pathway that goes through geniculate magnocellular layers/V1 interblobs/V2 thick stripes. On the other hand, it was previously revealed that thick and thin stripes receive direct projections from the pulvinar complex of the thalamus, but pale stripes do not. Furthermore, previous electrophysiological studies also revealed that there are two distinct visuotopic maps within the lateral pulvinar. Thus, we hypothesized that there is another set of parallel pathways between the pulvinar and V2. To address this possibility, we injected different kinds tracers, BDA, CTB-Alexa-488 and CTB-Alexa-555, into three consecutive thick/thin stripes in V2 after identifying V2 stripe maps by intrinsic signal optical imaging and examined retrograde labeling in the pulvinar of macaques. As a result, we found that there are a few patchy distinct labeling for each retrograde tracer, and that thick stripe-projecting compartments and thin stripe-projecting compartments are segregated, although they are located next to each other within the lateral pulvinar. Our study indicates a possibility that there are several parallel pathways within the pulvinar-V2 projection, similar to the manner of geniculo-striate projections.

## Development of Pulvino-Cortical Circuits: Implications for Visual Behaviours and Disorders

**James Bourne**

Australian Regenerative Medicine Institute, Monash University

## 

The pulvinar is the largest collection of nuclei of the thalamus in primates, including humans, comprising 3 nuclei and further subdivisions. Even though it has been demonstrated to be embedded within sensory systems and connect with the majority of the neocortex, its function remains unclear. Over the past decade, my group have been instrumental in demonstrating in the marmoset monkey the role of the medial subdivision of the inferior pulvinar in the development of the dorsal stream visual cortex and the manifestations of a lesion to this region of the brain in early life. To this end, we now know that this area plays an implicit role in the development of the visual cortex and establishment of visuomotor behaviours, such as reaching and grasping. Furthermore, we have evidence that the pulvinar can route visual information to the visual cortex following a lesion of the geniculostriate pathway in early life in both monkeys and humans. Collectively, these data demonstrate an essential role for the inferior pulvinar thalamic nuclei in early life. Furthermore, up to this point, it was suggested that the thalamocortical circuits were ‘hardwired’ by birth yet we now have evidence and an example of their inherent plastic nature early in life and ability to reroute sensory information.

## Oral Session 1-1 (July 29, 2019): Stereopsis

## Luminance-Disparity Interaction in Human Visual Cortices

**Pei-Yin Chen^1^, Chien-Chung Chen^1,2^**

^1^Department of Psychology, National Taiwan University, Taipei

^2^Center for Neurobiology and Cognitive Science, National Taiwan University

## 

The perceived depth from disparity in random dot stereograms depends on luminance contrast in the image (Chen et al., 2016). Here, we investigated the neural mechanisms underlying such effect by using a block-design fMRI experiment. We measured the BOLD activation in KO and first-tier retinotopically defined visual areas as a function of luminance contrast and disparity. The test stimuli were square random dot stereograms (20.16 × 20.26 degree) that gave the percept of either a flat surface (zero disparity) or a sinewave modulated in depth (corrugated surface). The luminance contrast ranged from 5% to 80%. In the first-tier visual areas (V1, V2, and V3) and hV4, BOLD signals increased progressively with luminance contrast but were independent from disparity modulation. In area V3A, V3B and KO, the BOLD response was independent of luminance contrast in the zero disparity condition, but increased with luminance contrast whenever there was a visible depth modulation. In sum, the disparity sensitive areas, such as V3A, V3B and KO, can display a contrast dependent activation but only when there is a depth modulation in the stimuli. Such disparity specific luminance contrast activation may relate to the luminance contrast effect on perceived depth from disparity.

**Grant:** MOST(Taiwan) 107-2420-H-002-029-DR

## 

**Keywords:** stereopsis, fMRI, binocular vision, kinetic occipital

## Neural Substrate for Reversed-Depth Perception Generated by Anti-Correlated Random-Dot Stereograms in the Human Brain

**Bayu Gautama Wundari^1^, Hiroshi Ban^1,2^, Ichiro Fujita^1,2^**

^1^Graduate School of Frontier Biosciences, Osaka University

^2^Center for Information and Neural Networks (CiNet), Osaka University & NICT

Grant: none

## 

Disparity-selective binocular neurons in the primary visual cortex show attenuated and inverted tuning for binocular disparity in anti-correlated random-dot stereogram (aRDSs). Such disparity tuning result in faint and reversed-depth perception where observers perceive “near” for uncrossed disparities and “far” for crossed disparities. Which neurons in the brain encode binocular disparity represented by that attenuated and inverted tuning, leading to reversed-depth? We investigated this issue by measuring the brain activities of 23 human observers with functional magnetic resonance imaging (fMRI). Throughout the fMRI scanning, they observed an engineered random-dot stereogram (RDS) that had variation in dot-contrast match level (anti-correlated, half-correlated, and full-correlated) for the RDS in the center and a correlated RDS (cRDS) in the surround. We performed decoding analysis on each brain region by training a linear support vector machine with voxel patterns of cRDSs to classify near-far disparity voxel patterns of aRDSs. Brain region V3A showed the classification accuracy significantly below chance, suggesting that this area was associated with reversed-depth. This finding demonstrated that the higher visual cortex exploits the representations of attenuated and inverted disparity tuning from the lower visual cortex. It therefore sheds new light on the hierarchical depth computation in the human brain.

**Grant:** none

## 

**Keywords:** Stereopsis, Reversed-depth perception, Random-dot stereograms, Brain encoding-decoding analysis

## Oblique Effect in 3D Gradient Discriminations Revealed by Psychophysics and MEG

**Huining Wu^1^, Yuji Ikegaya^2,3^, Hiroshi Ban^1,3^**

^1^Graduate School of Frontier Biosciences, Osaka University

^2^Graduate School of Pharmaceutical Sciences, The University of Tokyo

^3^Center for Information and Neural Networks (CiNet), National Institute of Information and Communications Technology

## 

Discrimination performance for oblique orientations is relatively lower compared to that for horizontal/ vertical contours (Oblique effect). Recent studies have shown that the oblique effect is not limited to orientation judgements but is commonly observed for the other visual features such as motion. To elucidate these perceptual anisotropies is important since it sheds insights on how visual neurons are organized and it can be utilized for modeling visual systems. Here we report a novel oblique effect in 3D vision. Specifically, we found that surfaces tilted nearby the frontoparallel plane were more discriminable compared to those tilted more to the eye-sight line. We further explored the neural substrate of the 3D oblique effect by comparing MEG (Elekta, Neuromag, 360 ch, 1 kHz sampling) responses evoked by eight tilted surfaces (-52.5 to 52.5 deg). Multivariate pattern analyses revealed distinct peaks of the 3D gradient discrimination performance at 150, 240, and 320 ms after the stimulus onset. The latter two peaks, which were thought to be derived from V3A, reflected the 3D oblique effect. These results suggest that the 3D oblique effect has its ground in the middle stage of dorsal hierarchical depth processing, not in the anisotropies of neural organizations in early areas.

**Grant:** none

## 

**Keywords:** 3D visions, Oblique effect, MEG

## A Deterministic Approach to 3D Vision from Multiple Cues

**Jovan T. Kemp and Fulvio Domini**

Brown University

## 

Perceived 3D properties, such as depth, are typically modeled as an average of depth estimates from individual image signals (or cues) weighted by their reliabilities. Notably, it is assumed that depth cues must be processed independently by unbiased estimators maximizing accessible likelihood functions. Here, we propose an alternative account to this probabilistic framework whereby a deterministic function maps a vector of scaled image signals to a depth estimate. This function maximizes depth discriminability while minimizing sensitivity to variations in image properties unrelated to changes in physical depth. Under this formulation we predict a fixed relationship between (1) a vector magnitude and perceived depth and (2) a change in vector magnitude and a Just-Noticeable Difference (JND). The first study investigating these predictions tasked participants to adjust a 2D probe to indicate the perceived depth of a 3D stimulus. In the second study participants adjusted a series of cue-conflict stimuli until their perceived depth matched the size of the 2D probe. The JNDs were measured in both studies, with the second study directly comparing the predictions of our model and typical Maximum Likelihood Estimation models. Results show that this novel approach provides the best fit of the data without free parameters.

**Grant:** none

## 

**Keywords:** 3D Vision, Psychophysics, Cue Combination

## The Width Underestimation of 3D Objects with Image Rotation

**Marie Morita^1,2^, Yoshitaka Fujii^3^ and Takao Sato^4^**

^1^Department of Psychology, Ritsumeikan University

^2^Research Fellow of Japan Society for the Promotion of Science

^3^Research Organization of Open Innovation and Collaboration, Ritsumeikan University

^4^College of Comprehensive Psychology, Ritsumeikan University

## 

The gaze and body orientation of a person depicted in a portrait painting appears to follow the observer even when observers move around. This is called the Mona Lisa effect. In addition, the face appears thinner when this effect occurs. This is a break-down of shape constancy and suggests the physical orientation of the image is not obtained or not used by the observer. In this study, we investigated whether the width underestimation of object with slanted image occurs with general 3D objects or space, not only with human portrait. In experiments, we presented 2D images of 3D objects (car, clock and building) and a scenery (road extending in depth direction) as test stimuli, and a H-shaped line-drawing as a reference. The rotation angle was varied in 5 steps between －30 to －30 deg. Observers were asked to compare width of the object or road and reference H, and to judge which appeared wider. The results showed that object width was perceived narrower with increasing rotation angle. However, road width was perceived narrower, but not as much as other objects. These results suggest that width underestimation with rotation occurs only with 3D objects.

**Grant:** none

## 

**Keywords:** depth perception, binocular vision, shape perception

## A Joint Motion/Stereo Constraint

**Jiawei Zhou^1^, Yiya Chen^1^, Zhimo Yao^1^, Pi-Chun Huang^2^ and Robert F. Hess^3^**

^1^School of Ophthalmology and Optometry and Eye hospital, and State Key Laboratory of Ophthalmology, Optometry and Vision Science, Wenzhou Medical University, Wenzhou

^2^Dept. Psychology, National Cheng Kung University

^3^McGill Vision Research, Dept. Ophthalmology, McGill University

## 

In clinical practice, stereo acuity isassessedonly using stationary stimuli. The purpose of this study was to develop a novel test toexamine the effect of lateral motion on stereo acuity. In particular, 50 Gabors with randomized position were presented in a circular display window in each eye; half of them were moving coherently to the leftor the right and were assigned a disparity relative to fixation plane corresponding to the plane of the screen, while the other half of the elements were moving in the opposite direction and were assigned an equal and opposite disparity. Observers were instructed to detect whether the Gabors in the front plane moved to left or right. A staircase method was used to determine the stereo acuity. Sub-pixel stereo accuracy was achieved by recomputing rather than simply shifting element position. For the range of motion speed that we measured (from 0.17 to 5.33 degree/second), we show clear speed tuning of the stereo sensitivity in normal adults (F(5,35) = 7.839, p < 0.001). This motion/stereo constraint may reflect the processing of stereopsis within the dorsal pathway.

**Grant:** This work was supported by the National Natural Science Foundation of China grant NSFC 81500754, the Qianjiang Talent Project (QJD1702021) and the Wenzhou Medical University grant QTJ16005 to JZ, the ERA-NET Neuron grant (JTC2015) to RFH

## 

**Keywords:** motion, stereopsis, psychophysics

## Poster Session 1 (July 29, 2019)

## Effects of Emotional Facial Expression on the Temporal Resolution of Visual Processing

**Misa Kobayashi^1,2^ and Makoto Ichikawa^1^**

^1^Chiba University

^2^Japan Society for the Promotion of Science (DC1)

## 

Some previous studies have shown that viewing fearful facial expressions enhances the temporal resolution of visual processing. We investigated if the magnitude of this effect would vary with positional relationship between face and target stimuli. We presented a fearful or neutral facial stimulus for 500 ms at the fixation point, and 100 ms after removal of the facial stimulus, we presented sequential two 35-ms-square-targets with an interval (ranging from 12 to 82 ms) at the overlapping position with the facial stimulus or 1.5 arc deg above the facial stimulus. As an index of temporal resolution of visual processing, we measured the threshold duration to detect the interval of the target stimuli by using the methods of constant stimuli. Only in the overlapping condition, we found that the temporal resolution of visual processing reduced after viewing fearful face. While previous studies found enhancement in temporal resolution of visual processing, the present study found its reduction. The duration of facial stimulus and ISI between face and target stimulus were longer in the present study than those in the previous studies We are proposing that the difference in these temporal factors in stimulus presentation would cause differences in temporal resolution among studies.

**Grant:** Grant-in-Aid for JSPS Fellows

## 

**Keywords:** ATR facial expression database, method of constant stimuli, ANOVA

## Infants’ Perceptual Insensitivity to the Other-Race-Face in Multisensory Speech Perception

**Yuta Ujiie^1^, So Kanazawa^2^ and Masami K Yamaguchi^3^**

^1^Chukyo University

^2^Japan Women’s University

^3^Chuo University

## 

Infants’ perceptual sensitivity to faces is broader in their early months, but gradually narrows to their own-race-face during the first year of life. Such perceptual narrowing is considered a modality-general, pan-sensory process. However, it is poorly understood whether perceptual narrowing to own-race-face appears in the development of audiovisual speech integration. Here, using functional near-infrared spectroscopy, we demonstrated increased superior temporal region activity in response to the integration of audiovisual speech for the own-race-face speaker, but not for the other-race-face speaker, in infants aged 8–9 months. Using the familiarization/novelty preference procedure, we further show that these infants can integrate audiovisual speech of the own-race-face, but not that of the other-race-face. These results imply that infants’ ability to integrate audiovisual speech narrows gradually to speech by the own-race-face speaker by the second half year of life, supporting the hypothesis that perceptual narrowing is a modality-general, pan-sensory process.

**Grant:** none

## 

**Keywords:** Own-race, the McGurk effect, Infant development, fNIRS

## Is Human Rapid Face Categorization Viewpoint Dependent?

**Charles C.-F. Or^1^, Talia L. Retter^2,3^, Bruno Rossion^2,4^**

^1^Division of Psychology, School of Social Sciences, Nanyang Technological University

^2^Psychological Sciences Research Institute & Institute of Neuroscience, University of Louvain

^3^Department of Psychology, University of Nevada

^4^Université de Lorraine, CNRS, CRAN, & CHRU-Nancy, Service de Neurologie

## 

Face-selective responses of single neurons in the monkey temporal cortex largely depend on head orientation, suggesting dominant view-dependent representations. In the human brain, however, whether early face-selective neural responses depend on head orientation remains largely unknown. To address this question, we presented 16 human observers with natural images of different objects alternating at a fast rate (F = 12 Hz), with face images appearing at F/9 = 1.33 Hz. Faces posed all full-frontal or at 3/4 views appeared in separate sequences. Significant face-selective responses were recorded in high-density EEG at 1.33 Hz and its harmonics mainly over the occipito-temporal regions. There were no amplitude differences between head orientations. Critically, alternating between full-frontal and ¾ views within a sequence led to significant responses at 0.67 Hz (F/18) and its harmonics, objectively isolating view-dependent face-selective responses over occipito-temporal regions. However, this response represented only a small (20-23%) fraction of the total face-selective activity, and did not reflect any difference in amplitude, being accounted for by a 21-ms earlier response for full-frontal than 3/4 views. Overall, these findings point to predominant view-independent face-selective processes in the human brain, with face categorization achieved earlier for full-frontal than rotated faces.

**Grant:** This work was supported by Singapore MOE AcRF Tier 1 Grant 2018-T1-001-069 to C.O. & B.R., NTU HASS-SUG to C.O., F.R.S.-FNRS grants to C.O. (FC 2773) & T.L.R. (FC 7159), and ERC Grant facessvep 284025 to B.R.

## 

**Keywords:** Face perception, viewpoint dependence/invariance, EEG, Face detection

## Seeing Money Images Explicitly Increases People’s Trust towards Emotionally Neutral Faces of Strangers

**On-Ting Lo**

College of Professional and Continuing Education, The Hong Kong Polytechnic University

## 

Is money the root of all evil? In the recent decade, studies consistently demonstrated that by merely activating the concepts of money in mind, people would become more self-oriented and have other psychological consequences that do not favor social interactions. It is arguable that first impression formation is the very first step for face-to-face social interaction. In this study, the money “self-orientation” effect was examined to test whether seeing a money image would decrease people’s trust towards newly met strangers’ faces. 40 participants were randomly assigned into two experimental conditions. In “money” condition, they were visually exposed to an image of banknote for 2 s then required to rate emotionally neutral faces’ (both in own race and other race) trustworthiness level. In contrast, being required to perform the same task, a phase-scrambled money image was shown in control condition instead. The results showed that participants rated the faces more trustworthy in the “money” condition. In addition, the trustworthiness rating was higher in other race faces. No conditionXrace interaction effect was found. This study demonstrated money would not necessarily promote self-orientation behaviors. By prolonging the explicit exposure time, money actually promotes prosocial behaviors such as making a stranger’s face look more trustworthy.

**Grant:** none

## 

**Keywords:** social vision, visual subliminal influence, visual perception

## Self-Perception Induces Visual Size Illusion

**Ying Zhang^1,2^, Li Wang^1^, Yi Jiang^1,2^**

^1^Institute of Psychology of the Chinese Academy of Sciences

^2^University of Chinese Academy of Sciences

## 

Previous studies have demonstrated that visual size perception is highly context-dependent and involves multiple neural computations. Here we report a novel perceptual illusion that self-face, being a unique and distinctive self-referential stimulus, can enlarge its perceived size. By using a size discrimination paradigm, we found that self-face was perceived as significantly larger than other faces of the same size, and this size overestimation effect was not observed when a famous face was compared with other faces. Moreover, such illusion effect could extend to a cartoon face repeatedly associated with one’s own face and further exert contextual influences on visual size perception of other objects. These findings together highlight the role of self-awareness in visual size perception, and point to a special mechanism of size perception tuned to self-referential information.

**Grant:** none

## 

**Keywords:** self face, size perception, size discrimination paradigm, association paradigm

## Less Is More—“Incomplete Beauty” of Facial Attractiveness Perception

**Cuihu Zhang, Mengliang Cao and Guomei Zhou**

Sun Yat-sen University

## 

Researchers have suggested that information shortage would enhance facial attractiveness rating, and the enhancement may be due to participants’ expectation of whole face’s attractiveness. The aim of the present study is to further explore this hypothesis. In the current study, covered faces (faces covered by sunglass, mask, vertical hand, lean hand) and uncovered whole faces were presented as stimuli. In three sequential sessions, participants were asked to (1) rate the attractiveness of uncovered parts of faces, (2) predict the whole face‘s attractiveness based on a covered face, (3) evaluate the facial attractiveness of whole faces. Our result showed that (1) attractiveness ratings of covered faces were higher than those of uncovered whole faces, (2) predicted attractiveness of whole faces were higher than attractiveness of uncovered faces, (3) male participants’ predicted attractiveness of female faces were higher than their predicted attractiveness of male faces. Besides, these effects were modulated by attractiveness of faces. Furthermore, we found a significant positive correlation between perceived attractiveness of uncovered faces and predicted attractiveness of whole faces based on uncovered faces. The present findings provide further evidence to attractiveness enhancement under information shortage (we call this phenomenon as Incomplete Beauty), and support the hypothesis that the enhancement is due to participants’ expectation of whole face’s attractiveness.

**Grant:** none

## 

**Keywords:** facial attractiveness, covers faces, information shortage, incomplete beauty

## Easterners Cannot Inhibit Fixations to Eye and Nose Regions in Face

**Toshikazu Kawagoe^1^, Kazuki Kihara^2^ and Wataru Teramoto^2^**

^1^College of Contemporary Psychology, Rikkyo University

^2^Division of Cognitive Psychology, Kumamoto University

## 

It is known that Western observers cannot inhibit their gaze to the eye region even if they are told to do so when they observe face stimuli. This might be due to the fact that focusing on the eye region is needed for the holistic processing that is essential for face perception. However, previous studies have indicated that the nose region is also important for face processing by Eastern observers. The question asked in this study was whether the Eastern observer can inhibit fixations on the eyes and nose. We have found that not only the eye, but also the nose region, automatically attracts Easterners’ gaze although they fixate on the eyes more than on the nose. We replicated and extended previous studies, providing some insights into the characteristics of the Eastern observer’s cognition.

**Grant:** none

## 

**Keywords:** face perception, eye tracking

## Consistency Is Key: Face Learning Strategies in Developmental Prosopagnosics

**Morgan Reedy, Hazel K. Godfrey, Tirta Susilo and Christel Devue**

Victoria University of Wellington

## 

Developmental prosopagnosics (DPs) have deficits in face recognition. Anecdotal evidence suggests some DPs use extra-facial information (e.g., hair, ears, jawline) and body information (e.g., clothing) as identity cues. These features can be diagnostic and allow identification in some cases. However, most laboratory-based studies use cropped images, neglecting their potential contribution, and perhaps exaggerating DPs’ deficits. This leads to the question, can DPs use extra-facial information to effectively learn an identity, when extra-facial information is consistent across learning and test? Thirty DPs studied videos of three identities and then performed a recognition task in which extra-facial features were consistent with learning (i.e., same hairstyle & makeup to learning) or were inconsistent with learning. To assess how extra-facial features contribute to recognition, half of the images were cropped to conceal extra-facial features and half were full headshots. Consistent with self-reported deficits, DPs’ recognition was impaired when extra-facial features were absent or not diagnostic of identity. However, controls also showed impairments in these conditions, and DPs were as accurate as controls when extra-facial features matched learning. Therefore, when extra-facial features are consistent with learning, both DPs and controls use this information to recognize face they have learned.

**Grant:** none

## 

**Keywords:** Face Recognition, Face Learning

## The ERP Components Reveal the Interactions Between Configurations and the Other Race Effect In Facial Expression Perception

**Tsung-Tien Hsiung^1^, De-Syun Chang^2^, Chien-Chung Chen^2,3^**

^1^Taipei First Girls High School

^2^Department of Psychology, National Taiwan University

^3^Center for Neurobiology and Cognitive Science, National Taiwan University

## 

We investigated the effect of feature configurations on the other-race effect (ORE), or it is more difficult to identify a face of another race, in facial expression perception. The stimuli were frontal view images of 15 Asian and 14 Caucasian models in seven expressions, including neutral, happy, sad, fearful, angry, surprised, and disgusted, in both in both upright and inverted orientations. The ERP waveform were recorded on 20 Asians and 20 Caucasians who have stayed in Asia less than 6 months while they performed an expression categorization task. Each trial contained a brief presentation of a fixation mark, followed by a 400 ms presentation of an emotional face and a response interval in which a participant was required to indicate the category of the expression. The ERP components at 170 ms and 200 ms after stimuli onset (N170 and P200 respectively) showed a response intensity difference between in- race and other-race both in Asian and Caucasian Participants. However, While Asian participants showed ORE in both upright and inverted faces, Caucasian participants only showed ORE in upright faces. Our results suggest that the Caucasian participants are more susceptible to facial configuration effects than Asian participants in configuration judgements.

**Grant:** none

## 

**Keywords:** N170, Electroencephalogram, Expression Identification, Face Inversion Effect

## The Face Inversion Effect for Facial Expression Judgement Is Culture Dependent

**De-Syun Chang^1^, Tsung-Tien Hsiung^2^, Chien-Chung Chen^1,3^**

^1^Department of Psychology, National Taiwan University

^2^Taipei First Girls High School

^3^Center for Neurobiology and Cognitive Science, National Taiwan University

## 

We investigated the effect of familiarity on face inversion effect (FIE), or that it is more difficult to identify an up-side-down face, face expression perception and its neural substrates. The stimuli were frontal view images of 15 Asian and 14 Caucasian models in seven expressions: neutral, happy, sad, fearful, angry, surprised, and disgusted, in either upright or inverted orientation. The event-related potential was recorded on 20 Asians and 20 Caucasians who have stayed in Asia less than 6 months while they performed an expression categorization task. Each trial contained a brief presentation of a fixation mark, followed by a 400 ms presentation of an emotional face and a response interval in which a participant was required to indicate the category of the expression. The ERP component at 170 ms after stimuli onset (N170) showed an overall difference between the upright and the inverted faces, demonstrating a strong FIE. However, the Asian participants showed such FIE only for Asian faces but not Caucasian faces in the frontal and parietal electrodes. The Caucasian participants showed FIE in N170 for both Asian and Caucasian. In sum, such culture dependent FIE related ERP component is inconsistent with the universality theory for facial expression.

**Grant:** none

## 

**Keywords:** Other Race Effect, N170, Expression Identification, Event-Related Potential

## Relationship Between FaceFamiliarity and Pupil Mimicry

**Licheng Zhu, Hirohiko Kaneko and Rumi Hisakata**

Department of Information and Communications Engineering, Tokyo Institute of Technology

## 

The human eyes provide resourceful insights about the mental state. Recent studies reveal that there is a synchronicity of pupil responses during communication, which could be the result of a positive emotion (trust) (Kret et al., 2015). We considered that if pupil mimicry is due to the positive mental state, the familiarity of faces would affect the pupil mimicry response. In the current study, we examined this possibility. In addition, we examined the effect of luminance change due to the pupil change in the image because Madou et al. (2018) suggested that there was a significant effect of physical luminance change in stimuli on the pupil responses. We tested several stimuli sets, including luminance-equalized faces of different familiarity levels and scrambled luminance-equalized image as a control. We presented two black circles changing in size on the positions of the eyes in the image to simulate pupil fluctuation. Results showed that pupil mimicry occurred in all the patterns and stronger for the face images than for the scrambled images, indicating that the pupil mimicry was not just due to the luminance changes in the stimuli. However, at this point, we didn’t find the difference of pupil mimicry between the faces with different familiarity level.

**Grant:** none

## 

**Keywords:** Human Vision

## Effect of Viewing Direction on the Light from Above-Left Assumption

**Aki Tsuruhara^1^, Masashi Arake^1,2^, Yuko Aiba^3^ and Takenori Nomiyama^1^**

^1^Aeromedical Laboratory, Japan Air Self-Defense Force

^2^Department of Integrative Physiology and Bio-Nano Medicine, National Defense Medical College

^3^Advanced Defense Technology Center, Acquisition, Technology & Logistics Agency

## 

The ‘lean-on-the sun’ illusion is known among aircraft pilots, in which during flight in cloud the brightest part of the cloud is regarded as ‘up’ even if the sun is not directly overhead. By contrast, previous studies about shape-from-shading indicated human has an assumption that illumination is biased to the above-left rather than directly above. To elucidate this contradiction, we examined the effect of viewing direction. Aircraft pilots usually look down the ground and clouds while the previous shape-from-shading studies presented stimuli on a frontal parallel plane. In our experiment, we measured the time that took to detect a target within distractors under a single light that came from directly above or biased to left. There were two conditions of object array: in one condition, the array was simulated as it were looked down in 45 degrees; and in the other condition, in 90 degrees, that is, the object array appeared as if on the frontal parallel plane of the participants. The results showed the shortest detecting time was achieved under left-biased illumination in the both viewing conditions. Our results suggest that the ‘lean-on-the-sun’ illusion might have left-right asymmetry though action-perception dissociation must be examined in the future study.

**Grant:** none

## 

**Keywords:** the light from above-left assumption

## Relative Contribution of S and L–M Mechanisms to Perceptual Grouping

**Lee Lin^1^, Chien-Chung Chen^1,2^**

^1^Department of Psychology, National Taiwan University

^2^Center for Neurobiology and Cognitive Science, National Taiwan University

## 

We investigated color mechanisms in perceptual grouping with tripole Glass patterns, which consist of randomly distributed sets of three dots, including one anchor dot and two context dots. Observers may perceive a clockwise (CW) or counter-clockwise (CCW) spiral by grouping the anchor with one of the context dots. The chromaticity of the anchor and one context dots was placed at halfway between the L–M and S axes, while that of the other dots varied between ±90° from anchor chromaticity in the DKL color space. The contrast of the context dots changed from one to four times of the detection threshold. Participants were to respond whether they perceived a CW or CCW spiral. When the CCW dots contained a positive L–M component, the probability of perceiving a CW spiral, PCW, first increased and then decreased with CW contrast. PCW saturated early when the CCW chromaticity was near the S axis, suggesting a weak S-mechanism contribution. The result can explained by a model containing global templates that each receives inputs from chromatic-spatial linear filters tuned to either L–M or S mechanisms respectively. The percept is determined by the response difference between the CW and CCW templates.

**Grant:** MOST(Taiwan) 106-2410-H-002-074-MY2

## 

**Keywords:** perceptual grouping, color tuning mechanisms, divisive inhibition, psychophysics

## Large Color Contrast Effect Induced by a Thin White-Gap; Evidence for Interaction Between Color and Luminance

**Tama Kanematsu^1^, Kowa Koida^1,2^**

^1^Department of Computer Science and Engineering, Toyohashi University of Technology

^2^Electronics-Inspired Interdisciplinary Research Institute (EIIRIS), Toyohashi University of Technology Department of Computer Science and Engineering

## 

We found thin (0.031 deg) gray lines on a cyan background appeared red when the lines were surrounded by thin (0.031 deg) white-gaps. This effect was known as simultaneous color contrast, however, its magnitude was greatly enhanced than the line without gaps. We quantified an appearance of the gray line for no-gap, white-gap and black-gap conditions. Matched color of no-gap condition showed either color assimilation or contrast depending on its luminance, whereas that of white-gap condition were significantly shifted toward a complementary hue irrespective of its luminance. Almost no color shift was observed for black-gap condition. This indicates that any explanation based on spatial frequency or proximity is insufficient. Interaction between color and luminance would be critical. The illusion was prominent for thin lines and dots, however optical blur and chromatic aberration were not major factors. The illusion occurred regardless of hues if the appropriate thickness of lines were used; preferred thickness was larger for S cone axis background than L-M cone axis background. This phenomenon may be considered as an inference of color of thin objects which exceeds spatial resolution of color by remaining luminance pattern, such as thin branches on the sky background.

**Grant:** none

## 

**Keywords:** psychophysics, color, luminance

## Categorical Perception of Color in Tracking Depends on Language

**Mengdan Sun^1,2^, Xiaoqing Gao^1^ and Xuemin Zhang^2^**

^1^Center for Psychological Sciences, Zhejiang University

^2^Faculty of Psychology, Beijing Normal University

## 

Is our perception of the world shaped by the language we speak? This subject has provoked controversy over the past decades. Categorical perception (CP) of color suggests that cross-category colors are discriminated better than within-category colors, initially serving as the supporting evidence for the penetrability of language on perception. However, recent findings seem to suggest language-independent CP effects. Following our previous study that revealed CP effects in a tracking task, the current study investigated the effects are dependent on language or not. We conducted two experiments where two types of verbal interference task were implemented and assessed whether the CP effects in tracking would be disrupted. In Experiment 1, the verbal interference task was an eight-digit memorization task, while Experiment 2 replaced the digits by color words. It showed that the CP effects were not influenced by the digit memorization task (Exp.1) but reduced by the memorization of color words (Exp.2). Our results suggested that the CP effects in tracking derive from the use of color labels, supporting the role of language in dynamic visual organization. Furthermore, the ability of different verbal interference tasks differs in blocking the access to color labels.

**Grant:** none

## 

**Keywords:** color perception, categorical perception, behavioral

## Pupillary Responses to the Perceived Brightness of Simultaneous Contrast

**Kei Kanari**

Applied Brain Science Research Center, Brain Science Institute, Tamagawa University

## 

It has been reported that the pupillary response depicts not only the physical intensity of the stimulus but also the brightness of the stimulus. However, whether the perceived brightness processed in the primary visual cortex affects the pupillary response remains unclear. Hence, this study aims to examine whether the perceived brightness of the simultaneous contrast affects the pupillary response. The test stimulus consisted of a test disk and a ring surrounding it. The perceived brightness was manipulated by changing the width of the ring. Subjects responded to the brightness of the test disk by adjusting the luminance of the comparison disk. In addition, I measured the pupillary responses to the test stimuli. Results showed that the contrast effect increased as the ring width increased only when the ring luminance was lower than the disk luminance (increment condition), and the pupillary response corresponded to the perceived brightness in increment condition. This study suggests that the pupil is modulated by the perceived brightness processing in the primary visual cortex.

**Grant:** none

## 

**Keywords:** brightness, simultaneous contrast, pupillary response

## Asymmetric Brightness Effects with Dark vs Light Glare-like Stimuli

**Yuki Kobayashi^1,3^, Daniele Zavagno^2^ and Kazunori Morikawa^1^**

^1^School of Human Sciences, Osaka University

^2^Department of Psychology, University of Milano Bicocca

^3^Japan Society for the Promotion of Science

## 

A white surface surrounded by luminance gradients which are darker in the outer ends appears brighter than an equiluminant surface surrounded by uniform gray (i.e., the glare effect). Although the glare effect has been a subject of much research, its photometrical reversal (i.e., the center is dark and the outer ends of the surrounding gradients are lighter), which we dubbed as photometrical negative glare (PNG), is still left surprisingly unexplored. In the present study, perceived luminance in the center of PNG was examined. Twenty participants compared equiluminant central areas of a PNG target and a comparative one, and rated how much the latter appeared to be brighter or darker than the former. Five luminance values were employed for the equiluminant (black and four levels of gray) and three for the background (black, dark gray, and light gray). Similar conditions were set up for the glare effect, and all stimuli were repeated ten times in random order. We expected a darkening effect with PNG stimuli compared to brightness enhancements experienced with glare stimuli. Results showed instead a substantial brightness enhancement for PNG, with the exception of the black target, which showed a tendency to appear darker than the comparative one.

**Grant:** none

## 

**Keywords:** lightness and brightness, illusion

## Luminance Variability Discrimination in Brief Presentation

**Yusuke Takano^1^ and Eiji Kimura^2^**

^1^Graduate School of Science and Engineering, Chiba University

^2^Department of Psychology, Faculty of Letters, Chiba University

## 

We previously showed that, when asked to compare mean brightness of heterogeneous luminance arrays of disks, observers’ judgements were efficient but exhibited a bias toward the highest or lowest luminance depending on task requirement (Takano & Kimura, ICVS2015). There were also some implications that luminance variation of the stimulus could be quickly extracted. This study further examined tentative visual processes that efficiently code luminance variation. In Experiment 1, we investigated luminance distribution discrimination of briefly-presented (47 ms) arrays composed of heterogeneous 24 disks. The percentage of correct responses in the task could be described as a function of the difference in the standard deviation (SD) between the standard and comparison luminance distributions. In Experiment 2, we specifically asked observers to discriminate luminance variability between the standard and comparison stimulus arrays. The SD of the standard luminances was varied in four levels. Results showed that observers could efficiently and accurately discriminate luminance variability regardless of the level of the standard SD. Moreover, unlike in mean brightness judgements, no clear evidence was found for relying on more readily available proxies such as luminance range. Together, these findings suggest that luminance variability was processed in a qualitatively different fashion from mean luminance.

**Grant:** Supported by JSPS KAKENHI #26285162

## 

**Keywords:** ensemble perception, brightness perception, method of constant stimuli

## Fast Contrast Adaptation in Area 17

**Kota S Sasaki^1,2^, Kohei Kurihara^1^, Izumi Ohzawa^1,2^**

^1^Osaka University

^2^CiNet

## 

Images fade from perception when they continue falling on the same set of photoreceptors in retina without eye movements. After experiencing this fading, sudden removal of images yields perception of their negative images. To understand neurophysiological basis underlying this illusion, single unit activity was measured in the area 17 of anesthetized and paralyzed cats. The contrast response functions were measured using flashed gratings by changing the contrast randomly within a limited range while the other parameters (e.g., orientation) were customized and fixed at the optimal values for each neuron. Stimulus in some conditions included gratings whose contrast polarity was inverted (i.e., negative contrast). In pedestal conditions where the temporal average of contrasts was not zero, the contrast response functions shifted so that the pedestal could be cancelled almost perfectly. This adaptation completed much faster than well-known contrast gain control. As a result of this contrast cancellation, a blank screen produced the strongest responses when neurons were adapted to gratings of negative contrasts. This means that these neurons fires vigorously when adaptation to negative contrast is released by removing an adapting image. These results appear to be consistent with fading illusion and extraordinarily rapid fading reported by Coppola and Purves (1996).

**Grant:** KAKENHI 15H05921

## 

**Keywords:** contrast, adaptation, neurophysiology, illusion

## Color Naming Is More Reliable than Position Detection in Peripheral Visual Field

**Tatsuya Oikawa^1^ and Kowa Koida^2^**

^1^Department of Computer Science and Engineering, Toyohashi University of Technology

^2^Electronics-Inspired Interdisciplinary Research Institute, Toyohashi University of Technology

## 

Detection and identification are two different aspects of visual function. How these performances differ around threshold level stimuli? We measured the detection threshold of colored targets, and compared with accuracy of color appearance for the targets at the perithreshold contrast. In the detection task, observers were asked to respond the position of colored target from 6 possible sites on peripheral visual field. Using staircase method, the psychometric functions and thresholds were determined. In the appearance task, the observers were asked to identify the name of color from 6 options (red, green, yellow, purple, white, and black). Colored targets were determined by cone isolating axes, and the contrast was determined around detection thresholds. Contrary to expectations, the color naming performance was significantly higher than detection performance across sub- and supra-threshold levels. These results suggest that discrete processing of detection and identification. Recent neurological study of macaques indicates that superior colliculus-pulvinar-cortical pathway, is thought to be involved in spatial attention, also carry color information. Our results suggest that high contrast stimuli would be required to drive pulvinar pathway.

**Grant:** none

## 

**Keywords:** Psychophysics, Color vision

## Assessing Peripheral Visual Function in Myopia—a qCSF Study

**Jinrong Li^1^, Zhipeng Chen^1^ and Zhong-Lin Lu^2^**

^1^Zhongshan Ophthalmic Center, Sun Yat-sen University

^2^Department of Psychology, The Ohio State University

## 

### Purpose

This study evaluated contrast sensitivity function in peripheral vision in myopia and emmetropia with the qCSF method. Methods: The study recruited 19 myopia subjects and 12 normal subjects. Their BCVA were 0.00±0.00 logMAR. All subjects performed the qCSF test in foveal vision and fifteen peripheral locations (Superior, inferior, temporal and nasal quadrants at 6, 12, 18 and 240 eccentricities) and optical quality assessment with the double-pass Optical Quality Analysis System II, OQAS. The myopes wore soft contact lens with best corrected visual acuity (BCVA). The cutoff spatial frequency (cutoff SF) and the area under log CSF (AULCSF), and contrast thresholds were derived from qCSF test results. Results: Results from the OQAS assessment found that there was no significant optical quality difference between two groups (p>0.05). There was also no significant difference between two groups in any of the CSF metrics in foveal vision(p>0.05). Further analysis showed that myopes had significantly increased AULCSF in the superior(p = 0.026), inferior(p = 0.024) and nasal(p = 0.022) quadrants at 120, but not in other eccentricity. Conclusions: We speculate that these results may be attributed to compensatory improvements of peripheral vision from its extensive use during near visual activities in the emmetropization process of myopic visual system.

**Grant:** The National Natural Science Foundation of China (81770496) to Jinrong Li and the National Eye Institute (EY021553) to Zhong-lin Lu

## 

**Keywords:** myopia, peripheral vision, contrast sensitivity

## Sizing Up the Crowd When Perceiving Body Size

**Jason Bell^1^, Georgia Turnbull^1^, Joanna Alexi^1^, Georgina Mann^1^, Yanqi R Li^1^, Manja Engel^2^, Donna Bayliss^1^ and Simon Farrell^1^**

^1^School of Psychological Science, University of Western Australia

^2^Faculty of Social and Behavioural Sciences, Experimental Psychology/ Helmholtz Institute, Utrecht University

## 

Recent research has shown that serial dependencies in perception can bias body size estimates towards prior experience. The current study sought to build on that work by examining whether body size judgments are also biased by surrounding spatial information. To do this we created an Ebbinghaus Illusion with bodies. We used the HTC Vive Pro to present six inducers in a virtual annulus surrounding the central test body. The configuration resembled a small crowd, all facing the observer. We tested two conditions: one where the to-be-judged body was surrounded by overweight inducers and the other, containing thin inducers. Participants (N = 412) were randomly assigned to one condition each. Participants were instructed to ignore the inducers and judge the size of the central female body by clicking on a VAS. Results were consistent with the Ebbinghaus illusion: the central body appeared larger when surrounded by small inducers and vice versa. The nature of the interaction between the spatial (Ebbinghaus) and temporal (serial dependence) biases will also be described. Our findings shed further light on the perceptual causes of body size misperception, as well as the processes underpinning serial dependencies in perception.

**Grant:** none

## 

**Keywords:** object perception, serial dependencies, 3D

## Haptic Detection of Radial Frequency Patterns

**Goro Maehara^1^ and Jason Bell^2^**

^1^Department of Human Sciences, Kanagawa University

^2^School of Psychological Science, University of Western Australia

## 

An old psychological controversy is whether our perceptions have their intrinsic structures as whole entities or consist of combinations of local features. Recent studies on pattern vision addressed this issue using radial frequency (RF) patterns and suggest that a simple contour is processed globally. The present study attempts to investigate whether the haptic system similarly globally processes the simple RF contour shapes. We measured haptic detection thresholds of RF patterns at different numbers of radial deformations. The mean threshold significantly decreased as the number of deformations increased for RF5 (pentagon like shape). The slope of the function was −0.49 with the 95% CI ranging from −0.21 to −0.78. This slope was slightly steeper than the slope value of −0.33, which is predicted from probability summation. Since the haptic system could not simultaneously detect multiple deformations for the present stimuli, it seems reasonable to assume that haptic system globally processes RF5 contours. On the other hand, detection thresholds were comparable among different numbers of deformation cycles for RF3. Here, deformation detection is equivalent to detection of a single local curvature. We discuss the similarities and differences in contour perception between visual and haptic perception.

**Grant:** none

## 

**Keywords:** radial frequency, contour, psychophysics

## Seeing Sounds: The Role of Consonants in Sound Symbolism

**Yang-Chen Shen^1^, Yi-Chuan Chen^2^ and Pi-Chun Huang^1^**

^1^Department of Psychology, National Cheng Kung University

^2^Department of Medicine, Mackay Medical College

## 

Sound symbolism refers to associations between phonemes and certain visual properties. In one classic example, the meaningless sounds “bouba” and “kiki” are mapped onto rounded and angular shapes, respectively. We investigated whether the classification dimensions of English consonants (voiced/ voiceless, sonorant/obstruent, and abrupt/continent) correspond to the round/sharp contrast of visual features. We adapted twenty meaningless spoken words from previous studies, ten of which belonged to the round category and ten of which belonged to the sharp category. In each experimental trial, the participants heard one of the words and saw a rounded shape and an angular shape on the monitor. Participants then had to judge which shape provided a better match to the spoken word. The results of the sound–shape matches were predictable using the voiced/voiceless and sonorant/obstruent dimensions, but not the abrupt/continent dimension. Specifically, spoken words that consisted of voiced consonants and sonorant consonants were more likely to be matched to rounded shapes. Hence, we verified sound symbolism effects by demonstrating systematic mapping of contrasting dimensions among voiced/ voiceless consonants, sonorant/obstruent consonants, and round/sharp visual features. The English phonemes and visual shapes used in the current study provide useful tools to examine the universality of sound symbolism in future studies.

**Grant:** This work was supported by Ministry of Science and Technology in Taiwan (MOST 104-2628-H-006-001-MY3 to PCH and MOST 107-2410-H-715-001-MY2 to YCC)

## 

**Keywords:** sound-shape correspondence, Bouba/Kiki effect

## Perception of the Müller-Lyer Illusion in Budgerigars and Pigeons

**Sota Watanabe^1^, Noriyuki Nakamura^2^ and Kazuo Fujita^3^**

^1^Osaka Kyoiku University

^2^Toyo Gakuen University

^3^Kyoto University

## 

Whereas budgerigars (Melopsittacus undulatus) are one of the most popular species as companion animals, little is known about their visual perception. This study explores how budgerigars and pigeons (Columba livia) perceive the Müller-Lyer illusion. We first trained our birds to choose the shorter/longer of two isolated horizontal lines. After transfer of this performance to the Judd figures, which had two brackets on each end pointing in the same direction, and to the Müller–Lyer illusory figures, which had two inward- (>- < ) or outward-pointing ( < ->) brackets at the end of the target lines, we displayed novel Müller–Lyer figures, whose lengths of horizontal lines are different from those used in the previous phase, to our birds and determined whether their discriminations were affected by Müller–Lyer illusion. The results suggest both budgerigars and pigeons perceive the Müller–Lyer illusion and their illusionary tendency is the same as that of humans. We have confirmed that the birds’ responses could not be accounted for by overall lengths of the figures. This tendency of budgerigars and pigeons shown in our study is consistent with those reported of pigeons in Nakamura, Fujita, Ushitani, and Miyata (2006).

**Grant:** none

## 

**Keywords:** comparative cognition, geometric illusion

## Contrast Sensitivity Functions Measured under Different Dynamic Range Widths

**Misaki Hayasaka^1^, Takehiro Nagai^2^, Tomoharu Sato^3^, Tomonori Tashiro^1^, Yasuki Yamauchi^1^ and Ichiro Kuriki^4^**

^1^Yamagata University

^2^Tokyo Institute of Technology

^3^National Institute of Technology, Ichinoseki College

^4^Tohoku University

## 

In classical studies, contrast sensitivity functions (CSFs) were measured under full adaptation to a uniform background. However, this environment is extraordinary as a visual scene as compared with those in our daily life, which typically contain variegated regions with a wide luminance range. Here, we investigated effects of the dynamic range widths of the textured background on CSFs. The test stimulus was a Gabor patch on a uniform square pedestal, whose luminance was randomly chosen from five levels (0.8 to 107.4 cd/m^2^) in each trial. The background was a one-dimensional (line) texture orthogonal to the test stimulus with various luminance values, randomly chosen within one of four dynamic ranges widths, with the same mean luminance (16.4 cd/m^2^). In the results, the effects of dynamic range widths were not very pronounced in general; 1) spatial frequency profiles of CSFs were not very different across dynamic range widths, and 2) overall sensitivities were the highest around the average luminance of the background, regardless of the dynamic range widths. However, the highest sensitivity range spanned wider luminance levels of pedestal under wider dynamic range conditions. These results indicate that the overall sensitivity of CSF is optimized to the dynamic range of visual scene.

**Grant:** none

## 

**Keywords:** Contrast sensitivity functions, Psychophysics, High dynamic range

## Size of Global Arrangement Affects the Perceived Size of Local Elements

**Taiichiro Uechi^1^ and Makoto Ichikawa^2^**

^1^Graduate School of Science and Engineering Chiba University

^2^Faculty of Humanities Chiba University

## 

We found that perceived size for local circular elements, which form a circular arrangement, illusorily decreases with the expansion of the global circular arrangement (Uechi & Ichikawa, ECVP2018). In this study, we examined the effects of the amount of elements, size of elements, and size of global circular arrangement on perceived size of the local elements. Each stimulus was presented for 600 ms at the center of the display. After each stimulus observation, participants selected one of the circles, which is perceptually equivalent to the size of the circular elements in the global arrangement, from a chart that shows a row of white circles with different diameters. As we found in previous study, perceived size for local elements reduced as the global size increases. From this result, we are proposing that the information of reduction in viewing distance, which is extracted from global expansion, would cause perceptual reduction of the elemental size in terms of the “size-distance invariant hypothesis”. Furthermore, we found interaction of the three factors; for large elements size, perceived size of local elements increases as amount of elements increases with small global sizes. We will discuss the bases of this three-way interaction.

**Grant:** none

## 

**Keywords:** size illusion, circular arrangement, ANOVA

## Spatial Summation on a Pattern Mask

**Chia Hua Chen^1^, Chien Chung Chen^1,2^ and Christopher W. Tyler^3^**

^1^Department of Psychology, National Taiwan University

^2^Center for Neurology and Cognitive Science, National Taiwan University

^3^Smith-Kettlewell Eye Research Institute

## 

We investigated the mechanisms underlying spatial summation with a masking paradigm. The targets were Gabor patterns placed at 3 deg eccentricity to either the left or right of the fixation and elongated along an arc of the same radius on a concentric Gabor mask of the same radius. The observer’s task was to indicate whether the target in each trial was on the left or the right of the fixation. The Ψ staircase procedure was then used to measure the threshold at 75% accuracy. When the mask contrast was low, the target threshold initially decreases with size with slope −1 until target length reached 45′ half-height full-width (HHFW) and further decreased with slope −1/2 on log–log coordinates. At high mask contrast, the threshold also showed a −1 slope up to 45′ HHFW. However, the threshold was constant between 45′–210′ HHFW, followed by another −1 slope drop. Our results suggest that summation across local channels, which accounts for −1/2 slope decrease, can be eliminated by a high contrast mask, but not the summation within the receptive field of one channels, which accounts for the -1 decrease. The second drop in threshold suggests two summation mechanisms tuned to different size.

**Grant:** none

## 

**Keywords:** ideal observer, threshold, contrast detection, gain control

## Neural Networks for Computing Touch Topology

**Keiji Miura^1^ and Kazuki Nakada^2^**

^1^Kwansei Gakuin University

^2^Tsukuba University of Technology

## 

While tactile or visual image data have been conventionally processed via filters or perceptron-like learning machines, the advent of computational topology enabled us to extract the globally consistent features from the local pixelwise data. For example, the invariants under continuous deformation such as the number of islands or holes in an image are informative for digits discrimination. However the real time computation is still hard and the parallelized algorithms are desired for the quickness to achieve interactive touch screens. We show that the invariants (#islands or #holes), irrespective of detailed touch shapes, can be obtained in a recurrent neural network after the iterative updates of its state (IEEE Access, 2014). Here we can count not only isolated touches but also “overlapped” touches by the Euler integral (1). When you only need the difference (#islands#holes), it suffices to instantly count the Poin-care–Hopf indices only for a handful of salient pixels (IEEE Access, 2015, 2017), as if only a few sensory neurons activated and mattered for global consistency. These neural implementations of computational topology may give a hint for the consistency-based sensory signal processing in the brain.

**Grant:** KAKENHI 18K11485

## 

**Keywords:** image processing, touch screens, topology, object recognition

## A New Demonstration of Amodal Completion Implied in Oyama’s (1960) Figure-Ground Reversible Image

**Kentaro Usui and Akiyoshi Kitaoka**

Ritsumeikan University

## 

When an object is occluded by another one, the occuded part is invisible but the object is perceived as a perfect one. The visual function to fill in the lacked part is called ‘amodal completion.’ In many cases, T-junctions are regarded as a critical cue of occlusion. In this contex, the area above the horizontal bar of ‘T’ appears to be closer to the observer and to occlude the ‘stem’ of ‘T.’ Here we propose a new type of amodal completion phenomenon that accompanies T-junctions in a different fashion, in which either side of the ‘stem’ appears to be closer.

**Grant:** none

## 

**Keywords:** amodal completion

## Angular Tuning of Tilt Illusion Depends upon Duration

**Saki Takao^1,2^, Colin WG Clifford^2^, Katsumi Watanabe^1,2^**

^1^Waseda University

^2^University of New South Wales

## 

Brief presentation induces larger simultaneous contrast effects (Kaneko et al., 2017), including the tilt illusion. This was explained in that the brief presentation led to higher uncertainty in determining the orientation of the test stimulus, which enhanced contextual modulation. Here, we examined the effect of duration on the tilt illusion with a wider range of center-surround orientation differences to examine whether higher uncertainty changes the angular tuning function. Centre and surround gratings were presented for 10 to 640 ms. Surround orientation was manipulated between ±7.5 to ±75 degrees for each duration. An interleaved Bayesian adaptive staircase method adjusted the orientation of the central grating to estimate a point of subjective verticality for each combination of duration and surround orientation. The results showed that the surround orientation at which the tilt illusion peaked changed with duration. Specifically, as the duration became shorter, the illusion became larger and the peak shifted toward a larger center-surround difference. These results suggest that presentation duration influences not only the magnitude but also the orientation profile of the processes underlying the tilt illusion.

**Grant:** none

## 

**Keywords:** visual perception, contextual effect, psychophysics

## Velocity of Self-Induced Optical Flow Contributes to Body Control Through Unconscious Speed Estimation of Self-Motion during Rotational Movement

**Ryo Tokuyama, Rumi Hisakata and Hirohiko Kaneko**

Department of Information and communications Engineering, Tokyo Institute of Technology

## 

In tune with VR technology development, some recent studies have revealed the effects of visual information on body control. For example, Langbehn et al. (2018) demonstrated that the under-threshold distortion of the virtual path induced subconscious bending of walking trajectory. Most people will be able to image visual information affects body control, however, how visual information has an impact on body control is still unclear. To investigate this issue, we focused on optical flow and body movement during self-rotation about the yaw axis. We manipulated the relative velocity of optical flow during self-rotation and instructed the subjects to keep rotating at constant speed. The subjects wore a head-mount display (HTC VIVE) and observed a spherical space composed of floating dots. The subject’s movement was detected by the VIVE-tracking system, resulting in that we could manipulate the subject’s optical flow velocity in real time. Because the subjects were asked to ignore the visual stimulus, they were presumed to keep the physical speed of self-rotation. However, the physical velocity of self-rotation decreased as the relative velocity of optical flow increased, namely they couldn’t ignore it. The result suggests that the estimation of the velocity of the self-rotation is inevitably affected by visual information even if enough information from the vestibular system is available during self-rotation.

**Grant:** none

## 

**Keywords:** perception of self-motion, optic flow, self-rotation, body-control

## Motion Priming Reveals Visual Instability under Sudden Change in Ambient Light Level

**Sanae Yoshimoto^1^ and Tatsuto Takeuchi^2^**

^1^Hiroshima University

^2^Japan Women’s University

## 

When you dance on a club floor under heavily flashing lights, you might feel as though your body is floating while the world around you is shaking. Since spatiotopic representation in the brain is key to the perception of visual stability, we speculate that a sudden change in ambient light level deteriorates the construction of spatiotopic representation. This hypothesis was examined using a phenomenon called visual motion priming, in which the perceived direction of a directionally ambiguous test stimulus is influenced by the moving direction of a priming stimulus. Participants performed saccades after the termination of the primer and then judged the perceived direction of the test stimulus. A test stimulus was presented in the retinotopic location or screen-based spatiotopic location. The average luminance of the display changed from photopic to mesopic level or vice versa at a time point between primer offset and test onset. We found that when the average luminance was changed, spatiotopic priming disappeared, whereas retinotopic priming was not influenced. The different responses of cones and rods to luminance change would contribute to the disappearance of spatiotopic priming—a signature of the disturbance in construction of spatiotopic representation—which would eventually lead to visual instability.

**Grant:** none

## 

**Keywords:** motion perception, visual stability, eye movement, ambient light level

## Development of Human Infants’ Receptive Field Mechanisms in Visual Motion Processing

**Yusuke Nakashima^1^, So Kanazawa^2^ and Masami K Yamaguchi^1^**

^1^Department of Psychology, Chuo University

^2^Department of Psychology, Japan Women’s University

## 

Perceiving motion direction is more difficult when the size of high-contrast motion stimuli is increased. This perceptual phenomenon is considered to reflect surround suppression, a receptive field property observed in MT neurons. Here, we demonstrate that this phenomenon can be observed in human infants. We measured motion direction discrimination with small and large drifting gratings in 3- to 8-month-old infants using familiarization/novelty-preference procedure. Infants at 7–8 months of age showed higher sensitivity for a small motion stimulus than for a large one. However, infants under 6 months showed the opposite result; motion sensitivity was higher for a large stimulus. These results suggest that suppressive surround regions beyond classical receptive fields develop in the second half of the first year. Moreover, we investigated the size of receptive fields using this phenomenon and found that the center region of receptive fields was larger in 3–4-month-old infants than in 7–8-month-old infants. Our findings suggest that receptive fields related to motion processing are broad and do not have extra-classical receptive fields in early infancy, and that they become narrower and acquire suppressive surround regions in the first year of life.

**Grant:** none

## 

**Keywords:** visual development, motion, infant

## Interactions Between Luminance-Defined and Orientation-Defined Visual Rotations on Visually Induced Self-Rotation Illusion (Roll Vection)

**Shinji Nakamura**

Nihon Fukushi University

## 

Uniform motion of large visual display which mostly occupies observer’s field of view can induce illusory self-motion perception toward the opposite direction (visually induced self-motion perception, also known as vection). Our previous studies have been indicated that visual rotation defined by orientations of visual elements (fractal rotation) can effectively induce roll vection, even in the situation where the visual inducer didn’t contain any luminance modulations (APCV 2017). The present investigation further examined the effects of the non-luminance-based motion, using a visual situation where the luminance-defined (luminance rotation) and orientation-defined (fractal rotation) visual rotations were convoluted with each other and employed as a visual inducer. Psychophysical experiment with 13 participants revealed that in the condition where the luminance and the fractal rotations were contradicted, the luminance rotation became dominant to determine perceived strength and direction of roll vection. The fractal rotation cannot overcome the dominating effects of the luminance rotation even if its luminance contrast was quite low, but still seems to have modifying effects on visual self-rotation perception in some cases.

**Grant:** none

## 

**Keywords:** self-motion perception, vection

## Interaction Between Form and Motion Processing: Neural Basis Investigated with Glass Patterns and Repetitive TMS

**Rita Donato^1^, Andrea Pavan^2^, Massimo Nucci^1^ and Gianluca Campana^1^**

^1^University of Padova

^2^University of Lincoln

## 

Glass patterns (GPs) are a class of visual stimuli useful to investigate global form perception and the interaction between form and motion processing. GPs are dot patterns formed by applying different geometric transformations to change the spatial relationship between dot pairs (dipoles), to create visual patterns that convey a specific global form. A rapid succession of different GPs also gives the impression of motion (dynamic GPs). In the present study, we investigated the neural basis of circular dynamic GPs by interfering with the use of repetitive transcranial magnetic stimulation (rTMS). Rotating random dot kinematograms (RDKs) were used as control stimuli. Participants performed a 2-interval forced choice task and had to discriminate between the presentation of GPs (or RDKs) vs. a random pattern (i.e., noise). The results showed that rTMS over V1/V2 interfered with the processing of both dynamic GPs and RDKs, while rTMS over V5/MT only interfered with motion processing, but not with the processing of dynamic GPs. These findings suggest that partially different neural substrates subtend the processing of dynamic GPs and circular motion.

**Grant:** none

## 

**Keywords:** Circular motion, Dynamic Glass patterns, Form-motion processing, repetitive transcranial magnetic stimulation

## Representation of Spatial Feature of Complex Motion in Areas MT and FST

**Takahisa M Sanada**

Kansai Medical University

## 

Spatial feature of visual motion in natural scene is typically non-uniform. For example, in liquid flows, various directions/speeds of motion vectors are spatially distributed in a complex manner. Recently, it was reported that the spatial smoothness of local motion vectors, characterized using the mean discrete Laplacian of motion vectors, correlated with rated liquidness impression by human psychophysics (Kawabe et al., 2015). Neurons in area FST are selective to spatial structure defined by motion (Mysore et al., 2010), and we hypothesized that FST neurons represent spatial features of complex motion by integrating multiple motion components. In the present study, we tested this possibility by recording responses of FST neurons to complex motion stimuli with various degrees of spatial smoothness. 8 different levels of spatial smoothness of motion were generated by manipulating mean discrete Laplacian of motion vectors. Sixty-four stimuli were prepared in combination of 8 spatial smoothness and 8 directions. If a neuron is related to representing spatial feature of complex motion, it should respond at particular Laplacian levels. We found that subset of FST neurons which showed broad direction selectivity responded when the spatial smoothness was high, suggesting that spatial feature of complex motion might be represented in area FST.

**Grant:** This research is supported by JSPS (JP16H01681, JP16K00384, JP18H05016)

## 

**Keywords:** Electrophysiology, Motion, Shitsukan

## The Disappearance of Global Apparent Rotational Motion with Local Drifting Sinusoidal Gratings

**Hoko Nakada, Katsuhito Yamoto and Ikuya Murakami**

Department of Psychology, The University of Tokyo

## 

Apparent rotational motion is perceived when Gaussian blobs of two different luminances are alternately arranged to configure a ring and switch their luminances. We added local sinusoidal gratings drifting upward or downward to each blob and switched their motion directions simultaneously with their luminance switching. We found that the appearance of rotational motion was significantly decreased and sometimes completely abolished. This indicates that correspondence matching, motion energy, etc., necessary for perceiving a global apparent rotational motion, are obscured by local sinusoidal gratings drifting in vertical directions, not along the trajectory of apparent rotation. One possibility is that the temporal frequency power in the first-order motion masks the frequency of the luminance switching. However, this is unlikely because, regardless of the frequency of the switching, the apparent rotational motion was suppressed by the sinusoidal gratings drifting above certain speed. We will discuss other possibilities that may be related to the disappearance of apparent rotational motion, such as local motion directions and the synchronization of luminance switching, aiming to elucidate the mechanisms underlying the global perception of apparent rotational motion.

**Grant:** Supported by JSPS KAKENHI (JP18H05523)

## 

**Keywords:** motion

## Decoding Image Motion Using Deep Neural Network Features

**Ken U Shirakawa^1^, Yukiyasu Kamitani^1,2^**

^1^Graduate School of Inforoto University

^2^ATR Computational Neuroscience Laboratories

## 

While visual images are represented in hierarchical neural representations with diverse complexity, motion information is often assumed to be processed by detecting monolithic spatio-temporal features. To explore diverse neural representations of image motion, we used spatio-temporal features derived from a deep neural network (DNN) model pre-trained to classify action in a moment. The DNN feature values of natural movie stimuli were predicted (decoded) from the fMRI responses in the visual cortical areas, and the decoding performance was compared across the combinations of DNN layers and visual areas. We found that low-/high-layer DNN feature values were predicted better from the brain activity patterns in low-/high-level visual areas, respectively, replicating the hierarchical homology static image features found in our previous work. Decoded feature patterns were useful to identify seen movies. These results suggest that diverse motion-related features could be represented in the hierarchical visual cortical areas.

**Grant:** none

## 

**Keywords:** Brain decoding, Image motion, deep neural network

## Visual Confidence on Global Motion Depends on Local Motion Ambiguity and Type of Motion Noise

**Angela M.W. Lam and Alan L.F. Lee**

Department of Applied Psychology, Lingnan University

## 

Perceptual confidence has been found to correlate with task performance in general, and is believed to be independent of stimulus features. However, certain stimulus feature could induce a subjective sense of uncertainty, which could potentially influence confidence judgments beyond task performance. The present studies aimed at assessing the effects of the ambiguity of local motion signals on perceptual confidence on a global-motion task. Participants first discriminated the global motion directions of two multiple-aperture, global-motion patterns, one generated using multiple Gabor elements and the other using multiple Plaid elements. They then performed a two-interval, forced-choice confidence task by choosing which of the two perceptual responses they were more confident in being correct. In Experiment 1, when perceptual performance was controlled by varying coherence, we found that participants chose plaids more often than Gabors, even with perceptual performance matched between the two patterns. In Experiment 2, when perceptual performance was controlled by varying luminance contrast of noisy pixels in every motion frame, such “plaid preference” in confidence bias was significantly weakened. Our results show that, at the same level of objective task performance, subject perceptual confidence depends on both the ambiguity of local motion signals and the type of noise.

**Grant:** Direct Grant from Lingnan University (DR18A7)

## 

**Keywords:** Motion, Psychophysics, Motion integration, Visual confidence

## Visual Confidence Depends on Serial Task Difficulty and Explicit Report of Confidence Judgments

**Frankie H.F. Law and Alan L.F. Lee**

Department of Applied Psychology, Lingnan University

## 

In a series of hundreds of trials, confidence on a visual task demonstrates serial dependence. However, it remains unclear what serial factors give rise to such serial dependence. In the present study, we addressed this question by building a series of motion-discrimination tasks based on trial sequences with manipulated properties. In every trial, participants performed a left-right, direction-discrimination task on a random-dot motion pattern, and simultaneously indicated their confidence on a four-point scale. We first calibrated task difficult to individual participants’ discrimination sensitivity. Then, we manipulated serial task difficulty by controlling motion coherence, so that a trial with medium difficulty level was preceded by either easy trials or hard trials. In Experiment 1, we found that confidence rating for the medium-difficulty trial was higher when it was preceded by easy trials than when preceded by difficult trials, although direction-discrimination accuracy remained constant. In Experiment 2, such serial dependence on task difficulty was found to weaken significantly when participants were instructed to judge motion speed instead of to give confidence judgment in the preceding trial. Our findings suggest that both task difficulty and explicit report of confidence in preceding trials contribute to the serial dependence of judgments on visual confidence.

**Grant:** Direct Grant from Lingnan University (DR18A7)

## 

**Keywords:** psychophysics, serial dependence, motion, visual confidence

## The Effect of the Relative Depth Positions of Disparity-Defined Objects for Apparent Motion Perception and Object Tracking

**Hidetoshi Kanaya^1^, Marie Morita^2^ and Takao Sato^1^**

^1^College of Comprehensive Psychology, Ritsumeikan University

^2^Department of Psychology, Graduate School of Letters, Ritsumeikan University

## 

We examined the effect of depth positions of the stimulus for both perception of apparent motion and object tracking. The stimulus was disparity-defined rectangular objects that were generated within a dynamic random-dot stereogram. In Experiment 1, perception rates were measured for two-frame apparent motion. The two rectangular stimuli were located either near or far relative to the fixation plane, and were successively presented with an ISI varied between 0 and 533.3 ms. The disparity was plus or minus 12.24 min. It was found that motion perception rates were lower for far object than for near objects when ISIs were less than 100 ms. In Experiment 2, object tracking scores in multiple object tracking task were measured when the tracked objects were located either near or far. ISI was fixed at 0 ms. It was found that the tracking scores were significantly lower for far objects than for near objects. In sum, the performance was worse with far objects for both motion perception and object tracking. These results indicate both motion and tracking were mediated by mechanisms that distinguish depth, and suggest that mechanisms underlying the two tasks are not the regular second-order motion mechanism.

**Grant:** none

## 

**Keywords:** motion, binocular vision, psychophysics, tracking

## Perceived Spatial Alignment of Moving Objects Varies with Properties of Abrupt Events

**Junji Yanagi and Makoto Ichikawa**

Chiba University

## 

When two moving objects reverse their directions abruptly and asynchronously, the perceptual alignment of their reversal locations is systematically affected according to the temporal relationship between two events. For instance, the second reversal tended to be perceived to overshoot against the first reversal (Fechner Day 2017). In this study, we investigated whether this effect of the temporal asynchrony on the spatial alignment perception was specific to the motion reversal. Instead of the two motion reversals, we used motion offset (termination) and onset (initiation) as the two events to be judged their spatial alignment. Using the method of constant stimuli, we measured the spatial shift of the two events to be perceived as spatially aligned under various conditions of their temporal relationships. The results did not coincide with that of the motion reversals. The termination location tended to be perceived to undershoot against the initiation when the termination occurred after the initiation. Furthermore, there were large individual differences. These tendencies were quite contrasting with those found for the motion reversals. These results suggest that the perceptual process of the motion reversals should be different from that of other types of motion events. We will discuss the underlying mechanisms of these phenomena.

**Grant:** none

## 

**Keywords:** psychophysics, motion, spatial alignment

## The Relationship Between Eye-Dominance for Motion Perception and Postural Control

**Toshiki Fukui^1^ and Miyuki G. Kamachi^2^**

^1^Graduate School of Engineering, Kogakuin University

^2^Faculty of Informatics, Kogakuin University

## 

Humans in the standing position utilize visual information related to body sway to maintain a stable posture. Previous studies have shown that visual stimuli caused forward or backward slanting, thus resulting in body sway. In this study, we investigated whether body sway deviation could be attributed to the difference in a person’s eye dominance when perceiving motion directions and/or velocities. We conducted experiments to investigate how the participant’s body sway changed when vection occurred by viewing a visual motion stimulus. Visual movement velocities of 0.25, 1.0, and 4.0 deg/s were utilized as velocity parameters. Visual motion stimuli were monocularly presented independently on a monitor. Real-time data at 50 Hz was recorded for gravity centers with each participant in the standing position during stimulus presentation. The results showed that the body sway deviation was greater when a motion stimulus was presented to the non-dominant eye than to the dominant eye. Moreover, the sway occurred even when the velocity of the motion stimulus was almost undetectable (0.25 deg/s). More sensitive body sways were generated by visual processing of information presented to the non-dominant eye, suggesting that our visual system may obtain environmental information from the non-dominant eye.

**Grant:** none

## 

**Keywords:** motion perception, eye dominance, body sway, peripheral vision

## Visual and Tactile Perception of the Wind in the Virtual Reality environment

**Yuya Nishimaki^1^, Naokazu Goda^2^ and Miyuki G. Kamachi^3^**

^1^Graduate School of Engineering, Kogakuin University

^2^National Institute for Physiological Sciences

^3^Faculty of Informatics, Kogakuin University

## 

We can get a realistic sensation by integrating visual and tactile information in the virtual reality (VR) environment. Since many VR devices are attached to our body directly, if their synchronization is temporally imbalanced, users’ realistic sensations are going to be low. On the other hand, environmental winds around users can be presented without any direct devices. The integration process of visual and tactile perceptions for wind is unknown. We conducted experiments to explore the integration intensity under the combination of the two senses. We compared perceived intensity of wind under the conditions of visual only, tactile only, and a combination of visual and tactile senses, using the HMD and/or wind displays. We focused on intensities and directions of wind in visual (a flapping flag) and tactile (wind from fans) information as experimental parameters. In experiments 1 and 2, the sensitivity of each modality was measured. Results showed that humans can accurately discriminate the intensity of wind, if the information is presented independently. In experiments 3 and 4, we measured whether human sensitivity is affected according to the difference of matched and mismatched directions between visual and wind stimulations. Results showed that visual presentation influences intensity of wind perception.

**Grant:** none

## 

**Keywords:** virtual reality, realistic sensation, multi-sensory integration, wind perception

## Relationship Between Occurrence of 2D- and 3D- Footsteps Illusion and “The Law of Inertia” Hypothesis-based Bidirectional Reinforcement Learning in Human Perception

**Naoya Torisato, Hiromaru Nakagawa, Yoshitaka Fukaya, Shoko Hira and Sakuichi Ohtsuka**

Kagoshima University

## 

Visual illusions offer good cues for elucidating how perception normally works and why it sometimes fails. In this study, we examined the footsteps illusion (FI) in occluded- and non-occluded-conditions in 2D and 3D environments. A 24 inch stereo LCD (BENQ ZL2420T) was employed. Ten observers, who had adequate dynamic stereoscopic acuity, participated in the experiments. First, all results showed that FI onset delay in all non-occluded-conditions was long enough that the observers were assured of being able to discriminate low-contrast contours clearly. Taking into account that the delay was longer in 3D- than in 2D- non-occluded-conditions for the complete camouflage condition (Kumasaki 2015), it is suggested that the observers adopted “the law of inertia” hypothesis with highest priority if they were able to perceive full contours of the target, real or subjective, at a distance offset from the referenced plane. Next, the variation in onset delay between in 2D- and 3D- occluded-conditions strongly depended on observer history. In conclusion, it is suggested that (1) object contours are essential for perceiving FI, and (2) the illusion is not the simple consequence of edge contrast deterioration but is triggered by top-down reinforcement learning, both positively and negatively, as developed by attention experience.

**Grant:** none

## 

**Keywords:** visual illusion, perception, psychophysics, motion

## Motion Aftereffects with Different Adaptation Duration Investigating Color- and Luminance-Motion Processes

**Wakana Koshizaka^2^, Weijing Ren^2^, Ichiro Kuriki^1,2^, Yasuhiro Hatori^1,2^, Chiahuei Tseng^1,2^, Satoshi Shioiri^1,2^**

^1^Research Institute of Electrical Communication, Tohoku University

^2^Graduate School of Information Sciences, Tohoku University

## 

While separate pathways for processing color and luminance signals have been identified physiologically, perceptual interactions between luminance- and color-defined motion (LM and CM) were reported in several behavioral studies. How are the functional interactions of LM and CM signal take place in the cortical loci of visual information processing? To address this question, we investigated motion aftereffects (MAEs) by using functional magnetic resonance imaging (fMRI) technique. After presenting CM for 21s as adapting stimulus, we found evoked responses to the adapted direction larger than that to opposite direction, which is peculiar in MAE experiments and inconsistent with MAEs that we perceptually confirmed in the same condition. When adaptation period was 3s, following a previous study that showed normal MAEs for LM stimuli, we found normal MAE which is consistent with perceptual ones. These results suggest that the interaction between CM- and LM-processing mechanisms strongly depends on the duration of pre-adaptation. It might be the case that the interaction takes place at multiple loci, and some of them are irrelevant with perception.

**Grant:** none

## 

**Keywords:** motion aftereffect, fMRI, color, luminance

## The Perception of Motion Direction for Deformation-Defined Flow

**Takahiro Kawabe**

NTT Communication Science Laboratories

## 

In natural scenes, a transparent water flow deforms image information of its background scene and consequently produces deformation-defined flow. It is still unclear how human observers perceive deformation-defined flows. The purpose of this study was to check whether the human visual system discriminated the motion direction of the deformation-defined flow. To generate a deformation-defined flow, a natural scene image was deformed by means of deformation maps to which one of three spatial frequencies and a fixed translation speed were given. The task of observers was to judge whether the deformation-defined flow translated leftward or rightward. As a result, the observers reported the correct motion direction of the deformation flow when the spatial frequency of image deformation was relatively high. On the other hand, the observers robustly reported incorrect (that is, reverse) motion direction when the spatial frequency was relatively low. A deformation-defined flow possibly contains deformation-specific image features that contribute to motion direction discrimination. Otherwise, human observers possibly judge motion direction on the basis of the dominant first-order motion signals.

**Grant:** none

## 

**Keywords:** Motion perception, Deformation perception, Material perception

## Influence of Uniform Body Rotation on Perceived Speed of Visual Moving Objects

**Ryosuke Fukasaku, Rumi Hisakata and Hirohiko Kaneko**

Department of Information and Communications Engineering Course Tokyo Institute of Technology

## 

When we observe moving objects, we can perceive the actual velocity while we are moving, indicating that our visual system estimates it with taking the body movements into account. Hogendoorn et al. (2017) showed that visually perceived velocity changed depending on the direction of acceleration when the observer’s body rotated. However, it’s not clear whether the effect is due to the acceleration signals from the vestibular/somatosensory systems or other factors. Here, we examined the motion perception under the situation where there was no acceleration on the body along the direction of visual motion. Two circular objects moving to the left and right were simultaneously presented as visual stimulus and the observer judged which object moved faster. The observer’s body was rotated about the vertical axis with a constant angular velocity using a rotating platform. Results showed that they perceived the object moving in the same direction as the body faster under the condition of no acceleration along the direction of motion. This result suggested that the effect of body motion on the velocity perception is not due to the acceleration signals from vestibular/somatosensory systems and probably due to the mechanism to calculate the velocity on the spatial coordinates.

**Grant:** none

## 

**Keywords:** motion, multi sensory perception

## Motion Detection Sensitivity in the Same Direction as Motion Parallax Decreases Depending on the Binocular Disparity and Head Movement

**Rumi Hisakata and Hirohiko Kaneko**

School of Engineering, Tokyo Institute of Technology

## 

A static target appears to move in synchrony with head movement depending on the apparent distances (e.g., Tietz & Gogel, 1978). The direction of the concomitant motion is the opposite (same) to that of self-movement when observed at far (near) distance (e.g., Ono & Ujike, 2005). Based on the phenomenon, we presumed that visual motion sensitivity to horizontal directions would change depending on the distance from observers during head movement. The visual stimulus was a vertical Gabor patch with 2D noise. A moving chin rest guided subjects’ head movement. Both the Gabor and fixation point were moving in synchrony with the head movement, resulting in that the visual stimulus was almost static on the retina and there was no pursuit eye movement. We added binocular disparity to both the Gabor and 2D noise to define their depth relative to the display. Subjects answered which stimulus contained motion, former or later, with 2IFC. The result showed that the velocity of detection thresholds in the opposite direction from the head movement was greater on the near depth plane than the far depth plane. We will discuss about the possibility that visual system suppresses the motion signal concomitant with self-movement presented on the depth plane consistent with the motion parallax cue.

**Grant:** none

## 

**Keywords:** motion, psychophysics, head movement, binocular disparity

## The Role of Internal State in Monocular Deprivation-Induced Ocular Dominance Plasticity

**Zhifen He^1^, Yiya Chen^1^, Peng Zhang^2^, Robert F. Hess^3^ and Jiawei Zhou^1^**

^1^School of Ophthalmology and Optometry and Eye hospital, and State Key Laboratory of Ophthalmology, Optometry and Vision Science, Wenzhou Medical University

^2^Institute of Biophysics, Chinese Academy of Sciences

^3^McGill Vision Research, Dept. Ophthalmology, McGill University

## 

It is well known that there are characteristic differences in internal state when eyes are open versus eyes closed in the dark. It is however not clear, how the alternation in the internal state affects stimulus induced plasticity. In this study, we directly address this question by comparing the monocular deprivation-induced ocular dominance plasticity under conditions were the patched eye is either open or closed under the patch. Previous studies have shown that 2.5-hour of monocular deprivation temporarily strengthens the previously patched eye’s contribution to binocular perception. Here, we show that this form of visual plasticity is enhanced if the patched eye behind the occluder is kept open, even though the visual input is unchanged. We document these enhancements using both binocular combination and binocular rivalry end point measures. This effect could not be accounted for in terms of the change in the spontaneous alpha power in the eyes open/eyes close condition and may involve a separate change in internal state at a binocular site.

**Grant:** This work was supported by the NSFC 81500754 to JZ, the ERA-NET Neuron grant (JTC2015) to RFH, and the Wenzhou Public Welfare Science and Technology Project (Y20170765) to ZH

## 

**Keywords:** visual plasticy, ocular dominance, psychophysics

## Smallest Detectable Depth Difference on Multiview Autostereoscopic Displays

**Vincent Nourrit^1^, Di Zhang^2^, Peng Wang^3^, Xinzhu Sang^3^ and Jean-Louis de Bougrenet de la Tocnaye^1^**

^1^IMT Atlantique

^2^Communication university of China

^3^Beijing University of Posts and Telecommunications

## 

Autostereoscopic technology can provide an immersive viewing experience compared with 2D technology. Studies have shown that the number of views could impact the viewing experience but little information is available on the relation between the number of views and depth perception. In this study, we measured, in 14 healthy subjects (23.8±2.5 years old), the smallest detectable depth difference (SDDD) in normal viewing conditions on a multiple view autostereoscopic 3D display to assess the influence of the number of views on the measurement, as well as the nature of the stereogram stimulus (contour or randots). According to our results, the number of views and the stimuli has a significant impact on SDDD. When clinical measurements are conducted on such displays, 7 views is the minimum number of views required to ensure that the ability to perceive small disparities (30″) is tested effectively. Also, the use of random dots stereogram can lead to significantly better performances when compared to contour stereograms when the number of views is small (<7). For this reason, random dots stereograms should be chosen over contour stereograms when the view number is small, such as 2 views or 4 views.

**Grant:** none

## 

**Keywords:** 3D Displays

## Distance and Orientation Effects on Perceived Slant of Physical and Artificial Stimuli

**Fletcher John Hammond^1^, Andrew Hill^2^, Mark Horswill^1^ and Philip Grove^1^**

^1^University of Queensland

^2^Clinical Skills Development Service, Royal Brisbane & Women’s Hospital

## 

We investigated the differences in distance and orientation effects for physical and artificial stimuli. Previous studies have shown that changes in viewing distance and orientation lead to depth distortions for artificial 3D stimuli that do not occur for physical stimuli. This is because changes in distance and orientation lead to changes in the left and right eyes’ images of a real object that are different from changes in the images from an artificial 3D image (e.g., anaglyph). We investigated how the perceived slant of an anaglyph 3D stimulus scales with viewing distance and orientation in comparison to analogous physical stimuli. Participants viewed physical and artificial stimuli depicting two vertically abutting surfaces slanted relative to one another. Participants manually estimated the slant between the two surfaces with an unseen probe. We found that an increase in viewing distance led to a significant increase in perceived slant. We found no effect of orientation, with all viewing angles yielding similar reports of slant. Finally, there were no effects of viewing distance or orientation for the analogous physical stimuli. Constancy breaks down for artificial stimuli with changes in viewing distance but not orientation. Constancy holds for physical stimuli under similar viewing conditions.

**Grant:** University of Queensland Postgraduate Scholarship

## 

**Keywords:** Stereoscopic Vision, Constancy

## The Disparity Tuning Symmetry Explains the Degree of Solving the Correspondence Problem in Macaque Visual Areas MT and V4

**Toshihide W Yoshioka^1,2^, Takahiro Doi^3^, Mohammad Abdolrahmani^4^, Ichiro Fujita^1,2^**

^1^Grad Sch Front Biosci, Osaka Univ

^2^CiNet, Osaka Univ and NICT

^3^Dept Psychol Univ of Pennsylvania

^4^Lab for Neural Circuits and Behavior, RIKEN Brain Sci Inst

## 

To use binocular disparity as a depth cue, the visual system should find the corresponding visual features between the two eyes that originate from the same surface point in the 3D space. This correspondence problem is not solved at the first binocular stage in the primary visual cortex (V1). Here we examined the degree of solving the correspondence problem in areas MT and V4 of the monkeys by analyzing neural responses to graded anti-correlation of binocular visual stimuli. The responses of V4 neurons were more consistent with the solution to the correspondence problem than those of MT neurons. In each area, neurons with even-symmetric tuning curves tended to show a higher degree of correspondence computation than neurons with odd-symmetric ones. We further found that MT neurons exhibited a variety of disparity tunings ranging from odd-symmetric to even-symmetric and that most V4 neurons were even-symmetric. The latter two findings, together with an assumption of output nonlinearity following the initial disparity computation in V1, explain the difference in the degree of solving the correspondence problem. Thus, the mid-tier stages of the dorsal and ventral pathways, MT and V4, implement a common mechanism (i.e., disparity tuning shape) for distinct representations of stereoscopic depth.

**Grant:** KAKENHI JP (23240047,15H01437, 17H01381)

## 

**Keywords:** binocular stereopsis, neurophysiology, MT, V4

## Discrimination of 3D Spaces Generated by Binocular Disparity and Pictorial Cues

**Satoko Ohtsuka**

Saitama Institute of Technology

## 

It is plausible that perceptual 3D space generated from different depth cues, i.e., binocular disparity and pictorial cues, have different nature. This research was designed to seek to evidence suggesting the difference in characteristics of resultant 3D spaces between binocular and pictorial cues. Here we employed a modified compatibility technique. A target in the first experiment was painted in red or green, and was displayed in front of or behind the fixation plane with binocular or composite pictorial cues. Participants responded the target’s color by keys which were arranged in the depth direction. While results in the disparity condition showed a significant compatibility effect, they were not in the pictorial cues condition. The targets in the second experiment was manipulated to be in depth positions with the cues as in the previous experiment, further their depth position was indicated by 3D arrows. The participants responded the target’s position. The result showed an effect of the arrows on the participants’ response. A manipulation of temporal property of depth information presentation, to separate that was indicated by the cues and arrows, revealed distinct compatibility curves among the cues. These suggests the action-oriented nature for disparity while is not for the pictorial cues.

**Grant:** none

## 

**Keywords:** depth, psychophysics

## Common Cortical Representation of Convex–Concave Shapes from Different Depth Cues

**Zhen Li^1^ and Hiroaki Shigemasu^2^**

^1^Graduate School of Engineering, Kochi University of Technology

^2^School of Information, Kochi University of Technology

## 

We investigated whether common representation of convex–concave shapes from different depth cues (binocular disparity or perspective) were involved in ROIs by assessing shape classification accuracy using multi-voxel pattern analysis. ROIs included retinotopic areas and higher visual areas. Shapes which consisted of two slanted planes were depicted by three types of stimuli separately (random dot stereogram; black–white dotted lines with disparity; black-white dotted lines with perspective). Two different disparity stimuli types were used to examine whether shapes from disparity using different elements share common representation. We evaluated the accuracy of transfer classification between combinations of stimuli. Results showed that accuracy was significantly higher than chance level for all types of classification in dorsal intraparietal sulcus (DIPS). To further investigate whether the high accuracy was based on global shape information or the orientation information of the slanted planes, three similar types of stimuli were used to depict slanted planes in two different orientations on which transfer classification to distinguish orientation was performed. Results showed classification accuracy was around chance level. In summary, DIPS may be involved in global shape representation from different cues and this is not based on the orientation of slanted plane itself.

**Grant:** none

## 

**Keywords:** Shape perception, 3D VISION, MVPA, fMRI

## Generalized Representation of 3D Object Orientation in Human Visual Cortex

**Thanaphop Threethipthikoon^1^, Zhen Li^1^ and Hiroaki Shigemasu^2^**

^1^Graduate School of Engineering, Kochi University of Technology

^2^School of Information, Kochi University of Technology

## 

The representation of three-dimensional (3D) orientation is a fundamental feature of human vision which has been broadly studied in recent years. The cortical representation of stereoscopic 3D surface was investigated in our previous study, and the result showed that some regions of interest (ROI) in intraparietal sulcus (IPS) had a tendency for 3D shape orientation classification. Since it is well known that IPS area is involved in vision for action such as visually-guided pointing, grasping, and object manipulation, we adopted different stimuli that were expected to produce a better classification. We used 3D objects related to action for orientation classification with two different types of orientation, (1) slant-tilt 3D orientations and (2) 2D rotations, while the blood oxygen level-dependent signal was recorded from visual cortices. Multivariate pattern analysis classification was utilized to find relation between object orientation and ROIs in visual cortices. The results showed that some areas in IPS have consistency of prediction accuracy for 3D orientation while other ROIs showed high prediction in 2D orientation but had low accuracy in 3D orientation. These results suggested that IPS area is likely to represent 3D object orientation in human cortices.

**Grant:** none

## 

**Keywords:** object orientation, 3D orientation representation, MVPA, fMRI

## Can Changing Brightness with Head Movement Deliver Depth Perception Like as Motion Parallax?

**Masataka Kurokawa, Masahiro Furukawa and Taro Maeda**

Osaka University

## 

It is widely known that motion parallax delivers quite strong depth perception. Our question is whether the gradually and brightness change provide motion parallax cues. The display was placed in a darkroom and subjects observed the light spots while actively moving their heads. The distance between two light spots was reduced and measured the threshold for depth perception. Two methods were compared as a method of presenting images. The first was a conventional method of causing two light spots to follow the movement of their head. In the second method, one light spot followed their head movement as in the conventional method. Another light spot was presented by extracting two points on the trajectory along which the light spot moves, and using the two points alone to gradually change the brightness of each other based on their head position.

**Grant:** This research was supported by collaborative with research Komatsu MIRAI Construction Equipment Cooperative Research Center.

## 

**Keywords:** depth perception, motion parallax, head movement

## How Does Body Analogy Help Mental Rotation? Disentangling Bottom-Up and Top-Down Processes

**Hiroyuki Muto^1,2^**

^1^Research Organization of Open Innovation and Collaboration, Ritsumeikan University

^2^Japan Society for the Promotion of Science

## 

Object-based mental rotation can be easily performed when a to-be-rotated object is likened to a human body. Such body analogy is known to be effective when human bodies are used as stimuli instead of abstract objects and when a to-be-rotated abstract object is merely regarded as a human body, as bottom-up and top-down processes, respectively. The present study aims to evaluate the bottom-up and top-down contributions of body analogy to mental rotation in a same-different judgment task. Forty-four participants performed mental rotation of human-like objects (human-shaped cubes with a pattern like a human head) and abstract objects (human-shaped cubes with a nonsense pattern) in counterbalanced order. We assumed that those who completed the human-like condition first would take advantage of body analogy in a top-down manner for the subsequent abstract objects while those who completed the abstract condition first would not. Results showed faster mental rotation in the human-like condition, suggesting that the bottom-up process was beneficial. However, the condition order did not affect mental rotation speed, suggesting that the top-down process made a negligible contribution. These findings indicate that body analogy helps mental rotation via the bottom-up rather than topdown process at least during a same-different judgment task.

**Grant:** none

## 

**Keywords:** spatial cognition, mental rotation, embodied cognition

## Object-Motion and Self-Motion Differently Affect Peripersonal Space Representation

**Naoki Kuroda and Wataru Teramoto**

Department of Psychology, Graduate School of Social and Cultural Sciences, Kumamoto University

## 

Peripersonal space (PPS) is the space around the body parts. The present study investigates how object-motion and self-motion affects the range of PPS, using visuotactile interaction. Visual stimuli were presented through a head-mounted display to the participants in the study. These participants were then required to detect vibrotactile stimuli delivered to their chest as quickly as possible while watching an approaching object (Experiment 1) or while approaching a static object by a visually-induced self-motion (Experiment 2). The object was presented at various distances from the participant’s body (120, 240, 360, 480, and 600 cm). In the baseline condition, a tactile stimulus was delivered without the approaching object. PPS was defined as the range at which tactile detection was facilitated by the approaching visual object. Two approaching speeds was compared: 1.5 m/s and 6.0 m/s. The results showed that the PPS was larger for the faster speed condition when static participants watched the approaching object. This is consistent with a previous single-unit recording in monkeys. In contrast, when participants approached the static object by self-motion, the PPS expanded more than the distance tested in the present study, irrespective of the speed. These results suggest that the mechanisms underlying the PPS expansion were different for object-motion and self-motion.

**Grant:** This work was supported by JSPS KAKENHI Grant (17K00263)

## 

**Keywords:** multisensory integration, self-motion, peripersonal space, psychophysics

## Relationship Between Vection and Head Displacement in Sitting and Standing Postures

**Kanon Fujimoto and Hiroshi Ashida**

Graduate School of Letters, Kyoto University

## 

Visual optic flow that simulates self-motion often causes standing observers to adjust their postures as well as to perceive self-motion illusion (i.e., vection). Previous research has suggested common neural mechanisms underlying visually induced postural response and vection. Although body postures such as standing or sitting could affect both postural responses and vection, the relationship between these responses has not been sufficiently studied in non-standing postures. In this study, we presented radial or lateral optic flows through an Oculus Rift CV1 head-mounted display and tracked head displacement in sitting and standing postures. Vection was also measured concurrently. For radial optic flow, we found head displacement bias in the opposed direction as stimulus motion in the sitting condition, while we found a tendency of head displacement bias in the same direction as stimulus motion in the standing condition. Similar but less clear tendency was found for lateral optic flow. Vection measures were not significantly different across conditions. Our results showed that postural responses to optic flow are modulated by the body postures, whereas vection is not substantially changed, suggesting partially distinct neural mechanisms underlying postural control and vection.

**Grant:** HAYAO NAKAYAMA Foundation for Science & Technology and Culture; JSPS KAKENHI (19K03367)

## 

**Keywords:** vection, posture, sitting, optic flow

## Influence of Pre- and Post-Saccadic Contrast on Displacement Detection Across Saccades

**Shuhei Takano^1,2^, Kazumichi Matsumiya^2^, Chia-huei Tseng^1,2^, Ichiro Kuriki^1,2^, Heiner Deubel^3^, Satoshi Shioiri^1,2^**

^1^Research Institute of Electrical Communication, Tohoku University

^2^Graduate School of Information Sciences, Tohoku University

^3^Department Psychology, Ludwig-Maximilians-Universität München

## 

The visual system is less sensitive to displacement of visual stimuli during saccadic eye movements than that during fixation (saccadic suppression of displacement or SSD). SSD may have an important role to realize perceptually stable world with drastic retinal changes due to saccade. We measured the effect of stimulus contrast to investigate how pre- and post-saccadic stimulus influence displacement detection respectively and compared accuracy of saccade for ten individuals. Experimental results showed that increase of post-saccadic stimulus contrast decrease the detection rate, which suggests that SSD is an active suppression after saccade. We analyzed the correlation between the strength of suppression during saccade and saccadic accuracy, and found that the person with larger saccadic errors had stronger suppression of displacement. This may show that the active suppression during saccade have an important role for stabilizing perceived world under the condition with large retinal changes due to saccade.

**Grant:** none

## 

**Keywords:** eye movement, saccade, visual stability, psychophysics

## Measuring Cognitive Impairment Using a Test of Visual Scene Comprehension

**Stephanie Reeves^1^, Victoria L Williams^3^, Deborah Blacker^2,4^, Russell L Woods^1,5^**

^1^Schepens Eye Research Institute

^2^Gerontology Research Unit, Mass General Hospital

^3^Department of Neurology, Harvard Medical School

^4^Department of Psychiatry, Harvard Medical School

^5^Department of Ophthalmology, Harvard Medical School

## 

The process of interpreting and acting upon the visual environment requires functioning cognitive and visual systems. The Information Acquisition (IA) method is an objective measure of the ability to perceive and understand a visual scene, using verbal descriptions in response to an open-ended question. Previously, we showed that subjects with central vision loss and hemianopia have reduced IA scores. Since the task requires functional working memory and linguistic fluency, we asked if IA could be applied to cognitively impaired populations. We recruited 56 participants across the spectrum from normal cognition to mild dementia (mean age 81, 66 to 99 years) at Massachusetts General Hospital, Boston, Massachusetts. Subjects watched twenty 30-second video clips and described the visual content without time constraints. Each response was compared to a control group of responses using a “wisdom of the crowd” natural-language approach that generated a score for each response. Linear mixed models accounted for random effects of education, gender, and age. IA decreased with increasing levels of cognitive impairment with a dose response effect. This suggests that this test might be applied in cognitive as well as visually impaired populations for longitudinal monitoring. Additional analyses show relationships between specific cognitive domains and IA performance.

**Grant:** none

## 

**Keywords:** visual perception, cognition, impaired populations

## Effective Visual Space for a Simplified First-Person Shooter Game in a VR Environment Measured by Visual Masking Method

**Daiki Matsushita^1^, Yasuhiro Seya^2^ and Hiroyuki Shinoda^1^**

^1^Ritsumeikan University

^2^Aichi Shukutoku University

## 

Virtual reality (VR) headset provides a wearer a visual display of an entire field of view. However, the spatial extent of the visual field in which observers can utilize visual information is generally limited, and such a visual field associated with particular visual tasks is called an effective visual space (EVS). The present study examined EVS for a simplified first-person shooter game presented in a VR environment. The peripheral visual field was restricted by masks of various sizes, while participants played the game in which normal (Experiment 1) and horizontally inverted images (Experiment 2) were presented. The EVS was defined as the smallest size of the restricted field of view where a game score (i.e., number of hits) reached a plateau at the same level as that measured without visual masking. Experiment 1 showed that the game score increased with increasing the size of observable area of visual masking. The score reached a plateau at 80 degrees. Similar results were found in Experiment 2, even though the task was more demanding than in Experiment 1. From both experiments the EVS was estimated to be about 80 degrees in diameter.

**Grant:** none

## 

**Keywords:** Effective visual space, VR, visual masking method

## Human Factors in Navigating within Train Stations

**Zac Je Sern Yeap, Nidhi Deepu Rajan and Hong Xu**

Nanyang Technological University

## 

Successful and efficient navigation is often influenced by the design of the environment, including signage, lighting, or distinctive color schemes. In this study, we investigated the human factors influencing wayfinding in train stations in Singapore. Using focus groups discussions, a field survey, and a lab experiment using psychophysical methods, we investigated how brightness and color temperature affects people’s perception and judgment of wayfinding in the stations. We found that subjects preferred the station lights to be dimmer at night, contrary to current literature on lighting conditions in stations. Warmer light temperatures were preferred in small enclosed spaces, whereas cooler temperatures were preferred in large open spaces. We also found that using color schemes in stations increases identification accuracy, and training subjects to rely on color schemes rather than signage results in faster reaction times to identify the stations. Thus, our findings suggest that lighting temperature and color are important human factors in efficient wayfinding. While these findings would be useful in architectural design, it provides insights for using virtual reality to investigate such questions. Replicating our behavioral results in virtual reality would open up avenues for behavioral experiments without the constraints of a physical environment.

**Grant:** Nanyang Technological University

## 

**Keywords:** Visuospatial navigation, Visual identification, Psychophysics, Virtual Reality

## Cyber Sickness was Affected by Apparent Gravity of Virtual Environment at Side-Lying Position

**Yue Song^1^, Toshiya Tsuchihasi^1^, Jyumpei Matsumura^2^, Masaki Ogawa^1^, Atsuo Kawai^1^ and Naoki Isu^1^**

^1^Department of Information Engineering, Graduate school of Engineering, Mie University

^2^Department of Information Engineering, Faculty of Engineering, Mie University

## 

In sensory conflict theory, conflict of visual and vestibular inputs is a cause of cyber sickness. One example is “vection”, which is visually induced self-motion perception. In present virtual reality technology, we often experience this phenomenon and sometimes experience cyber sickness. In the previous study, we found that a sensation of attitude change relative to the gravity (SACRG) has important effects on cyber sickness. In this study, we investigate what effect gravity (true gravity, subjective gravity, etc...) has on cyber sickness. There were two conditions about the virtual environment (VE): axes and body axes (same with real environment, 90 degree side-lying from real environment). Three types of combination of them (VE and body axes were same with real environment, body axes were rotated, and both VE and body axes were rotated) were examined. Additionally, there were 2 conditions of virtual environment’s motion from participant (yaw, pitch), so totally 6 conditions in this experiment. Three subjective evaluations (discomfortness, vection strength, SACRG strength) were measured. Results showed that subjective values were increased with a sensation of attitude change relative to the “virtual environment’s” gravity. It should be confirmed by experiment that employs another virtual environment that has no apparent gravity.

**Grant:** This work was supported by JSPS KAKENHI Grant Number 18K11395

## 

**Keywords:** cyber sickness, vection, side-lying position, sensation of attitude change relative to the gravity

## Covariance Structure Modeling of Biosignal for Detection of Cyber Sickness

**Takumi Nara^1^, Tomoya Kai^1^, Yuichiro Suzuki^2^, Masaki Ogawa^1^, Atsuo Kawai^1^ and Naoki Isu^1^**

^1^Department of Information Engineering, Graduate School of Engineering, Faculty of Engineering, Mie University

^2^Department of Information Engineering, Mie University

## 

Recently, virtual reality technology is increasing and becoming pervasive to our daily life. However, the present virtual reality technology come with the problem of causing cyber sickness. There is need for a method for quantitatively expressing the discomfort of cyber sickness, but it was difficult and we need more time to study for it. Thus, as a first step, we aimed at detecting the onset of cyber sickness. In this research, we tried to make a covariance structure model of biosignals. Twelve biosignals and three subjective evaluations were measured. Participants operated a driving-simulator in seven types of scene and made responses every two minutes about subjective discomfort, arousal levels, and stress levels. Covariance structure analysis was conducted to measured biosignals and subjective evaluations. In the analysis, three common factors corresponding to discomfort, arousal levels, and stress levels were employed. Results were estimated by three indices (goodness of fit index.983, normed fit index.895, and root mean square error approximation.032), and we concluded this model is good but still improvable. Especially, we identified scope for improvement by clustering of individual differences. In fact, there was a better model than the result model when we consider about a personal model.

**Grant:** This work was supported by JSPS KAKENHI Grant Number 18K11395

## 

**Keywords:** Cyber sickness, Biosignal, Covariance structure analysis

## Research on Onboard Movie Showing Methods to Suppress Seasickness

**Akihiro Emoto^1^, Ryota Ishikawa^2^, Hikaru Hasegawa^1^, Masaki Ogawa^1,2^, Atsuo Kawai^1,2^, Naoki Isu^1,2^**

^1^Department of Information Engineering, Mie University

^2^Department of Information Engineering, Graduate School of Engineering, Mie University

## 

In order to suppress sensory inconsistencies that are the cause of motion sickness, we have created a method to suppress seasickness by watching a movie displaying an image that makes the visually perception of own motion together with the movie. A virtual environment (VE) that reproduces a landscape that allows subjects to perceive the horizontal and vertical were displayed / projected with a movie. Because the VE was counter-moving to the ship’s motion (rolling, pitching, and yawing), horizontal and vertical axes of the VE were perceived as static, like a distant view. Our previous report, this method were succeed to suppressed discomfortness to about 70% of that of the control (non VE) condition. In this study, effect of scale factor of VE’s motion was investigated in two experimental environments (actual ship and onboard theatre simulator). Our purpose was that make clear the best scale factor of counter-motion of the VE to ship motion. Results for both experimental environments showed tendency that the best scale factor is at about 50% of ship motion. However, these results were not supported by statistical indices (Dunnett test: alpha level is .05). The reasons of these results were discussed.

**Grant:** This work was supported by JSPS KAKENHI Grant Number 18K11395

## 

**Keywords:** Seasickness, Onboard Movie, Virtual Reality

## Contributions of 3D Circular Images to Incidence of Visually Induced Motion Sickness

**Toshiya Tsuchihashi^2^, Kenta Taniyama^2^, Kyohei Koike^1^, Naoki Isu^2^, Masaki Ogawa^2^ and Atsuo Kawai^2^**

^1^Department of Information Engineering, Mie University

^2^Department of Information Engineering, Graduate School of Engineering, Mie University

## 

A factor of great importance in Visually Induced Motion Sickness (VIMS) during 3D viewing is the visually induced self-motion sensation called vection. When the vection is felt, there is a mismatch between vestibular and visual sensation. On the sensory conflict theory, this mismatch generates VIMS. On the other hand, our previous work showed another important factor about VIMS: The sensation of attitude change relative to the gravity (SACRG). SACRG is difficult to explain in words, but easy to feel when playing on the bar. However, at the first report of a SACRG, there were many factors to modulate VIMS. In this study, we employed three factors: orientation of circular vection, axes of virtual environment, and moving speed. Participants answered to subjective evaluations discomfort, vection strength, and SACRG strength. The result of the experiment indicates that SACRG is one of the most important factors of VIMS. Results of regression analysis, specifically in the orientation of circular vection was roll condition, SACRG was larger effects than vection. We should confirm the effects of SACRG with more experiments to investigate the mechanisms of VIMS.

**Grant:** This work was supported by JSPS KAKENHI Grant Number 18K11395.

## 

**Keywords:** motion sickness, virtual reality, vection

## The Role of Parahippocampal Cortex in Scene Integration

**Seoyoung Lee and Olivia S Cheung**

New York University Abu Dhabi

## 

Humans perceive a coherent visual world across time and space. To update incoming sensory information while maintaining a stable representation of the environment, previous studies have suggested the role of the parahippocampal place area (PPA) in scene processing and ensemble processing. Using functional magnetic resonance imaging, we examined how the PPA might integrate information by relying on continuous spatial-temporal sequence of information flow, or mere shared elements in a scene. We used scene images that were divided into three segments, with 66% overlap between the first and second segments and 33% overlap between the first and the third segments. Participants (N = 17) viewed the three scene segments either sequentially or in a displaced order, identical segments for three times, or three completely different scenes. We found that the bilateral PPA not only showed significantly stronger activations for different scenes than identical scenes (p’s<.0001), but also for different scenes than three segments of the same scenes (p’s<.03). Moreover, the sequence order of the three segments did not affect the response amplitude (p’s>.56). These results suggest that although the PPA may not be sensitive to the sequence of information flow, it appears to integrate segments with shared elements to form a coherent representation.

**Grant:** none

## 

**Keywords:** scene perception, neuroimaging, PPA

## Oral Session 2-1 (July 30, 2019): Physiology

## Bifurcation Pathway in the Macaque Fovea for Unifying the Left and Right Halves of a Visual Field

**Yoshihiko Tsukamoto^1,2^ and Naoko Omi^2^**

^1^Hyogo College of Medicine

^2^Studio EM-Retina

## 

The left half of a visual field is represented in the right visual cortex, and the right half in the left cortex. The fact that we can perceive a uniform visual field with no discontinuity can be attributed to the complex cells that combine the simple cells representing the identical orientation and the identical visuotopic location from both left and right hemispheres via the corpus callosum. Correspondingly, a group of ganglion cells along the midline border project onto either the right or the left hemisphere as a cue for left-and-right unification. However, this currently postulated mechanism hardly explains the ultimate precision at the foveal center of the primate retina under the Hebbian rule of synaptic plasticity. By serial section transmission electron microscopy, we have found a novel pathway of bifurcation from a midget bipolar cell to two (so-called parvocellular or midget) ganglion cells. Two almost same copies of the single-cone driven neuronal activity are thought to be conveyed to both right and left hemispheres. The synchronously occurring and highly correlated neuronal activities of these two cells may contribute to the formation of synapses between correctly paired simple cells and a complex cell for left-and-right unification at the highest resolution.

**Grant:** none

## 

**Keywords:** neural circuits, foveal retina, electron microscopy, Hebbian rule

## White Matter Connections of the Human Cingulate Sulcus Visual Area (CSv)

**Maiko Uesaki^1,2,3^, Michele Furlan^4^, Andrew T Smith^5^, Hiromasa Takemura^6,7^**

^1^Open Innovation & Collaboration Research Organization, Ritsumeikan University

^2^Japan Society for the Promotion of Sciences

^3^School of Social Sciences, Nanyang Technological University (NTU)

^4^Scuola Internazionale Superiore di Studi Avanzati (SISSA)

^5^Department of Psychology, Royal Holloway, University of London

^6^Center for Information and Neural Networks (CiNet), National Institute of Information Communications Technology, and Osaka University

^7^Graduate School of Frontier Biosciences, Osaka University

## 

Human cingulate sulcus visual area (CSv) is located in the posterior part of the cingulate sulcus, and responds selectively to visual (Wall & Smith, 2008) and vestibular (Smith et al., 2011) cues to self-motion. Understanding of the structural connections associated with CSv could shed light on its roles in self-motion perception and locomotion. To this end, Smith et al. (2017) studied the structural connectivity of CSv using diffusion MRI. While their probabilistic method suggests cortical endpoints of CSv connections, it does not reveal the trajectories of the white matter tracts adjacent to CSv. Here, we investigate the white matter connections of CSv, in relation to known white matter tracts and association fibres by analysing data from Smith et al. (2017). We found that some CSv connections estimated by tractography likely belong to the superior longitudinal fasciculus I and cingulum, which are associated with motor planning and visuospatial processing (Thiebaut de Schotten et al., 2011; Kantarci et al., 2011). We also observed short-range tracts connecting CSv with areas in the parieto-occipital sulcus, superior parietal lobule and insula. Our findings provide further evidence for CSv’s role in guiding locomotion, by demonstrating its spatial proximity to the white matter tracts supporting relevant functions.

**Grant:** none

## 

**Keywords:** Human cingulate sulcus visual area, White matter tracts, Diffusion MRI, Functional MRI

## Attentional State Modulates Connectome-Based Predictions of Cognitive Performance

**Christopher L. Asplund^1,2,4,5,6^, Esther X.W. Wu^1,2^, Gwenisha J. Liaw^2^, Rui Zhe Goh^1^, Alisia M.J. Chee^3^, Tiffany T.Y. Chia^2^, B.T. Thomas Yeo^2,3,4,6^**

^1^Division of Social Sciences, Yale-NUS College

^2^Singapore Institute for Neurotechnology (SINAPSE), National University of Singapore

^3^Department of Electrical and Computer Engineering, Faculty of Engineering, National University of Singapore

^4^Clinical Imaging Research Centre, Yong Loo Lin School of Medicine

^5^Department of Psychology, Faculty of Arts and Social Sciences, National University of Singapore

^6^Centre for Cognitive Neuroscience, Duke-NUS Medical School

## 

### Purpose

Individual differences in sustained attention (Rosenberg et al., 2016), selective attention (Rosenberg et al., 2018), and fluid intelligence (Finn et al., 2015) can be linked to neural patterns through Connectome-based Predictive Models (CPMs; Shen, 2017). Here we tested whether these predictions held when fMRI-based functional connectivity data were collected during a demanding task, the attentional blink (AB).

**Methods:** fMRI data were collected during resting state and again while performing the AB task, for which participants (n = 73) searched for two items in a stream of distractors. Outside the scanner, sustained attention (Gradual Continuous Performance Task), selective attention (Attention Network Task; ANT) and fluid intelligence (Raven’s Progressive Matrices) were assessed. CPMs based on resting state (rs-fcMRI) and the AB task state (task-fcMRI) were used to account for behavioral task performance.

**Results:** In general, AB magnitude and selective attention could be best predicted from task-fcMRI, whereas fluid intelligence could be best predicted from rs-fcMRI. Sustained attention could be predicted by both rs-fcMRI and task-fcMRI. Strikingly, the functional connectivity patterns that predicted better AB performance predicted worse selective attention (ANT) performance, and vice versa.

**Conclusions:** Attentional state is an important modulator of CPM-based task predictions from fMRI data.

**Grant:** Yale-NUS startup grant, DSO National Laboratories grant

## 

**Keywords:** visual attention, fMRI, attentional state, individual differences

## Probing Contextual Influences in Macular Degeneration: Is the Reduced Inhibition a Sign of Cortical Reorganization?

**Giulio Contemori^1,2,3^, Luca Battaglini^1,2^, Clara Casco^1,2^**

^1^Department of General Psychology, University of Padova

^2^Neuro.Vis.U.S. Laboratory, University of Padova

^3^Université de Toulouse-UPS, Centre de Recherche Cerveau et Cognition

## 

Complete bilateral central vision loss leads macular degeneration patients (MD) to develop a preferred retinal locus for fixation (PRL) that presumably undergoes some use-dependent cortical changes over time. In this study, we challenge this hypothesis by comparing spatial integration in the PRL, in a symmetrical retinal position (non-PRL) and in a region with matched eccentricity in a control group. To do this, we probed the contextual influences by measuring the contrast gain for a vertical Gabor target, flanked by two high-contrast collinear masks with respect to the orthogonal baseline condition. Surprisingly, the between-groups analysis revealed that in both PRL and non-PRL, at the shortest target-to-flankers distance (2λ), the contextual influence was facilitatory rather than inhibitory as in controls. Further analysis with data collapsed across groups showed that this effect depends on the individual contrast sensitivity at the baseline. When the target-to-flankers contrast ratio increases the inhibition decreases and then switch to facilitation. However, when ratio surpasses 1 the facilitatory effect progressively reduces and then disappear. This relationship is well expressed by a ‘dipper’ function similar to those previously reported by Zenger and Sagi (1996) for normal vision. Contrary to previous interpretations, we concluded that this modulation reflects neither a phenomenon of spontaneous nor use-dependent cortical plasticity.

**Grant:** University of Padova

## 

**Keywords:** psychophysics, macular degeneration, brain plasticity, contrast sensitivity

## Combined Use of Intrinsic Optical Imaging and 2-Photon Ca2+Imaging for Determining Distribution of Stimulus-Specific Responses Across Macro-Architecture in Macaque Visual Cortex

**Yang Fang^1,2^, Gaku Hatanaka^1^, Mikio Inagaki^1,2^, Ryosuke F Takeuchi^1^, Ken-ichi Inoue^3^, Masahiko Takada^3^, Ichiro Fujita^1,2^**

^1^Graduate School of Frontier Biosciences, Osaka University

^2^Center for Information and Neural Networks (CiNet); Osaka University and National Institute of Information and Communications Technology (NICT)

^3^Primate Research Institute, Kyoto University

## 

Area V2 is a key stage where various types of visual information are routed to the dorsal and ventral streams. V2 CO stripes, thick, thin and pale, have their specific input/output routes. Elucidation of functional neurons distributed across stripes provides a clue for understanding the visual processing along the two streams. To relate individual neuron responses to micro-architecture of V2 stripes, we combine intrinsic signal optical (ISO) imaging and 2-photon Ca2+imaging in macaque visual cortex. We implanted an imaging chamber in a monkey (Macaca fuscata) and injected adeno-associated virus vector carrying a calcium indicator (GCaMP6s) downstream of CaMKIIα or Syn. We first performed ISO imaging, using different visual cues to activate and identify V2 stripes. Calcium responses recorded with wide-field single-photon imaging appeared locally (∼2 mm) at the virus injection sites. Orientation maps and color blob patterns in V1 and orientation specific stripes in V2 in these regions were spatially matched to those visualized with ISO imaging. This combined application of multiscale imaging will be useful to resolve the questions on the functional architecture of area V2 and to obtain insights into the question of exactly how different visual cues are segregated into the dorsal and ventral streams.

**Grant:** Ministry of Internal Affairs and Communications; KAKENHI JP18H05007; JST JPMJPR1683; AMED JP18DM0307021

## 

**Keywords:** Macaque visual cortex, Multiscale calcium imaging, Genetically Encoded Calcium Indicator

## Cross-Inhibition and Cross-Pattern Detectors in Macaque V1

**Cong Yu, Shu-Cheng Guan, Nian-Shen Ju and Shi-Ming Tang**

Peking University

## 

V1 neuronal responses to a grating are suppressed by a superimposed orthogonal grating. This cross-inhibition effect suggests divisive normalization, a crucial computation that determines the non-linearity in neuronal contrast response functions. There is also sporadic evidence that some V1 neurons may respond to cross grating patterns. We used two-photon calcium imaging to record the responses of superficial-layer neurons to a single Gabor or a cross pattern (two orthogonal Gabors) in macaque V1. Many V1 neurons (～70%) were orientation-tuned, and the peak responses to a 0.32-contrast grating were suppressed by an orthogonal grating at the same or lower (0.08) contrasts. There was no evidence for a winner-take-all effect, in which responses to a 0.32-contrast grating would be unaffected by a 0.08-contrast grating. The remaining neurons (～30%) showed weak and orientation non-selective responses to a 0.32-contrast single grating, but strongly responded to 0.32 + 0.32 cross gratings with pattern orientation tuning. The two groups of neurons formed a bimodal distribution in terms of their responses to single gratings and cross grating patterns, suggesting distinctive neural subpopulations in V1. The cross-pattern neurons cannot be revealed via traditional single-unit recordings that first map the RFs with oriented stimuli, and then assess the responses to cross gratings.

**Grant:** none

## 

**Keywords:** visual cortex / 2-photon imaging

## Neural Ensemble Representation of View Orientation in Monkey Inferior Temporal Cortex: A Comparison Between Face and Object Processing

**Ryusuke Hayashi**

Human Informatics Research Institute, National Institute of Advanced Industrial Science and Technology (AIST)

## 

Previous studies have revealed an extensive network devoted to face and object recognition along ventral visual pathway in monkey brain. Within this network, several interconnected areas where face selective neurons are clustered have been functionally distinguished by the way of coding view orientation and individual identity difference (Freiwald & Tsao, 2010). However, it remains unclear how the representation of face orientation and identity changes depending on image morphing toward the faces of different species or other objects. To address this question, I implanted three multi-electrode arrays on the surface of area TE and recorded multi-unit activities in response to the images randomly presented on a computer monitor. The images of 3D photorealistic models of human faces, monkey faces, cars, foods and shoes (4 exemplars per category) and their intermediate morphed models (25–75, 50–50, and 75–25% of two different categories) viewed from 5 different angles in azimuth were used as stimuli. Then, I analyzed the representational similarity (Kiegeskorte et al., 2008) change of the recorded neural data as a function of categories and morphing level. The results showed the different neural ensemble representation of orientation between face and other object images, as well as own-species bias of face identity representation.

**Grant:** MEXT KAKENHI (Grant-in-Aid for Scientific research) Grant numbers JP16K13117, JP18H05019, JP18H04208 and a NEDO grant in Aid of Research.

## 

**Keywords:** face recognition, object recognition, electrophysiology

## Oral Session 2-2 (July 30, 2019): Engineering

## Centrifugal Signal to the Avian Retina Improves Stimulus Detection and Target Discrimination

**Hiroyuki Uchiyama, Hiroshi Ohno, Takuto Kawasaki, Yuhki Ohwatari, Takahiro Narimatsu, Yusaku Miyanagi and Taiga Maeda**

Kagoshima University

## 

The centrifugal pathway to the avian retina is composed of three serially-connected neurons; 1) tecto-isthmo-optic (tecto-IO) neurons in the optic tectum (OT), 2) isthmo-optic (IO) neurons in the isthmo-optic nucleus (ION) and 3) IO target cells (IOTC) in the retina. Furthermore, the IOTCs may contact with a particular kind of bipolar cells that are immunoreactive for protein kinase C (PKC-BCs). The IO neurons are passively activated by visual stimuli and are also voluntarily activated just before head movements oriented toward their receptive fields. The activity of the IO neurons, or the centrifugal signal to the retina, facilitates visual responses of the retinal ganglion cells (RGCs) transiently and locally via the IO target cells and perhaps the PKC-BCs. Inactivation of the IO neurons during search and peck task revealed that the centrifugal signal to the retina improved stimulus detection and target discrimination by the intraretinal facilitation mechanisms. Then, topographically-biased distribution of RGCs’ population activity with a peak at the target location may correctly induce orienting to the target through visuomotor transformation probably by tecto-bulbar neurons.

**Grant:** KAKENHI

## 

**Keywords:** optic tectum, retina, centrifugal projection, attention

## Animal Experiment Platform with Wireless Multi-Channel Microstimulation System Usable for Cortical Vision Prosthesis

**Yuki Hayashida, Yuichi Umehira, Naoki Satoshiro, Kosuke Takayama, Shinnosuke Ishikawa and Tetsuya Yagi**

Graduate School of Engineering, Osaka University

## 

We have been studying on intra-cortical visual prostheses, in which spatiotemporal microstimulation corresponding to the visual scene is delivered to the cortices in visually impaired people in order to provide artificial vision. In this study, we aimed to build a platform of animal experiments that is indispensable to determine the optimum range of stimulation parameters in the visual prosthesis. Thus far, we have designed and prototyped a VLSI chip with the stimulation output of 64 channels, and wireless electronic modules that can control this up to 64 chips. Utilizing the voltage-sensitive dye imaging of the visual cortex in head-fixed rodents in vivo, we verified that microstimulation with high accuracy can be achieved as designed by using our stimulator chip. In addition, the quantitative relationship between the stimulation intensity and the cortical response was obtained from the imaging experiments, and the stimulation parameters considered to be effective for the visual prosthesis were determined. Subsequently, the above-mentioned electronic modules were modified to be wearable in order to conduct, as the next step, behavioral experiments in free-moving rodents. We are currently developing a basic control program for the wearable system and a behavioral experiment environment to demonstrate the system’s operation.

**Grant:** Grant-in-Aid for Scientific Research from MEXT, Japan (25282130 and 18K12059 to Y.H. and 16717084 to T.Y.), and Grant-in-Aid for Challenging Exploratory Research from MEXT, Japan (25560197 to T.Y.)

## 

**Keywords:** Visual prosthesis, Electronic system, Physiological experiment

## OSX and iOS Applications for Vision Science Education and Research

**Izumi Ohzawa^1,2^, Koichiro Nishi^1^, Naoharu Iwai^1^ and Takuma Hanaya^1^**

^1^Osaka University

^2^CiNet

## 

We have developed a number of applications for educational and research purposes for vision research. These applications will be available via App Store. [1] Two-dimensional Fourier transform and image filtering application (for OSX and iOS). This application allows loading an arbitrary image in standard formats and applies filtering in the 2D Fourier domain. Filters such as low-pass, band-pass and orientation filters are available. These filter parameters such as cut-off frequencies and orientations may be manipulated by mouse, and filtered images are displayed immadiately. [2] Real-time Visual Neuron Simulator (OSX) allows applying Fourier-domain filtering to video camera input in real-time in the 3D spatiotemporal frequency domain. By loading spatiotemporal receptive field data to the simulator, reponses of visual neurons are simulated. [3] Dynamic Random-Dot Stereogram for testing stereoacuity (iOS) allows using iOS devices such as an iPhone to perform measurements of stereoacuity by using the device as a low-cost head-mount display. Results of measurements may be sent via email to a specified address.

**Grant:** KAKENHI 15H05921

## 

**Keywords:** neurophysiology, visual neuron simulator, OSX and iOS application, Fourier filtering

## Gamification of Vision Test Improves Usability for Internet Experiments

**Kenchi Hosokawa^1^, Kazushi Maruya^1^, Shin’ya Nishida^2^, Satoshi Nakadomari^3,5^, Masayo Takahashi^4,5^**

^1^NTT Communication Science Laboratories

^2^Kyoto University

^3^Kobe City Eye Hospital

^4^RIKEN Center for Biosystems Dynamics Research, Laboratory for Retinal Regeneration

^5^NEXT VISION

## 

Conventional methods in vision science cannot be applied in internet-based experiments due to long experiment time and requirements of special environment. We developed a test battery which can be used in vision experiments over internet. The test consists of a set of video games (contrast sensitivity function (CSF), visual field, multiple object tracking and character identification). We modified protocols of conventional test and embedded them into those game-tests. Next, we evaluated usability and validity of our game-tests. Thirteen persons participated in the evaluation experiment. They played game-tests, and evaluated subjective enjoyability, operability and difficulty. The playing times and performances were also recorded. The same participants performed experiments with conventional methods. As a results, more than 90% players finished every game-tests within 3 minutes. Subjective evaluation scores were higher in the game-test than in the conventional-tests. CSFs measured in the game-test were comparable to those of conventional tests if the players are familiar with the games (Hosokawa et al., VSS2019). In other tests, the performance decreased as game’s difficulty level increased, suggesting that players adequately performed tasks and their performance were correctly reflected on the data. Those results shows potential usability of the our tests for the internet experiments.

**Grant:** none

## 

**Keywords:** psychophysics, big data, gamification

## Effects of Display Compensation, Speed and StereopsIs on Motion Perception in an Immersive Virtual Environment Viewed on a Head-Mounted Display (HMD)

**Wilson Luu^1,2^, Barbara Zangerl^1,2^, Michael Kalloniatis^1,2^ and Juno Kim^1^**

^1^University of New South Wales

^2^Centre for Eye Health

## 

We used the Oculus Rift (CV1) HMD to better understand motion processing in participants with and without clinically-defined global stereopsis. We assessed the impacts of immersion, perceived scene rigidity and cybersickness on the seated perception of illusory self-motion in depth (i.e., vection) by manipulating simulated speed and display compensation for active lateral head movements. During active head movements, the display either presented pure radial optic flow (active uncompensated viewing) or a combination of radial and lamellar flow consistent with a constant spatial direction of self-motion (active compensated viewing). We also performed a control condition with viewing of pure radial flow (passive viewing). Eye movements and patterns of retinal motion were controlled with a static fixation point rendered on the display at the lower portion of the flow field. Interestingly, active head-movement displays with and without compensation generated equally superior vection strength, compared with passive viewing of pure radial flow in stereo-normal participants. However, stereo-impaired participants reported vection advantages in active uncompensated viewing conditions compared with active compensated and passive viewing conditions. Both stereo-impaired and stereo-normal participants also reported poorer rigidity in active viewing conditions compared with passive viewing conditions.

**Grant:** None

## 

**Keywords:** Motion Perception, Virtual Reality, Stereopsis

## Perceived Scene Stability Predicts Presence and Cybersickness

**Juno Kim^1^ and Stephen Palmisano^2^**

^1^University of New South Wales (UNSW Sydney)

^2^University of Wollongong

## 

Cybersickness is the adverse experience of oculomotor discomfort, disorientation or nausea commonly reported by users of head-mounted displays (HMDs). We instructed participants to generate pitch head movements in response to a metronome at either 0.5 Hz or 1.0 Hz. We systematically increased the display lag of the Oculus Rift CV1 above its benchmark latency (<9 ms). We found that increasing display lag increased perceived scene instability, and this perceived scene instability increased with the speed of head movement. Participants who reported greater perceived scene instability also reported weaker spatial presence (i.e., the illusory experience of being “there” in the virtual environment). Severity of cybersickness was also predicted by the magnitude of perceived scene instability. These findings suggest that the human visual system’s sensitivity to display lag depends on perceived scene instability, which in turn depends on the speed of head movement.

**Grant:** Australian Research Council (ARC) Future Fellowship

## 

**Keywords:** Virtual Reality, Presence, Cybersickness, Psychophysics

## AR/VR Safety Implications for Training

**Logan B. McIntosh, Guy M. Wallis and Philip M. Grove**

University of Queensland

## 

Virtual Reality Head-Mounted Displays and their application in virtual reality (VR) have increased in popularity. However, user discomfort and sickness hinder more widespread use. Although “cybersickness” is common in VR, individuals differ in how quickly or severely they are affected, given the same virtual environment. One explanation for these differences is that they are, at least in part, due to differences in sensory processes across individuals. We investigated whether individual differences on five binocular sensory processes were related to reports of past experiences with sickness stimuli. 108 participants completed a sickness history questionnaire, and psychophysical measures (interleaved staircase) of vergence eye movement duration, vergence latency, vergence range, range of fusible disparities, and static stereoacuity. Thresholds on all measures varied across participants, though the questionnaire data was markedly negatively skewed. We found a relationship between Gender and susceptibility to cybersickness, replicating previous studies. We did not find relationships between stereoacuity and cybersickness nor between the eye movement measures and cybersickness. One explanation for the null results is the inaccuracy and skew of the introspective, retrospective questionnaire measure.

**Grant:** none

## 

**Keywords:** Virtual Reality, Cybersickness, Visual Perception, Psychophysics

## Sensory Feedback Reduces Weber’s Law in Perception and Action Tasks

**Ailin Deng, Evan Cesanek and Fulvio Domini**

Brown University

## 

Weber’s law—the variability scales with physical stimulus magnitude—has been widely observed in perceptual responses. Recent studies have shown that action is immune to this law, which has been touted as a feature of specialized vision-for-action processing, separate from perceptual processing. In this study, we investigated the alternative hypothesis that sensory feedback plays a central role in the reduction of scalar variability. First, we investigated the role of online visual feedback and final haptic feedback in grasping. Weber’s law was only observed without either type of feedback. Instead, introducing visual feedback or haptic feedback substantially diminished scalar variability. In the two follow-up studies, we also tested the effect of sensory feedback on manual size estimation (MSE) — a widely used perceptual task. In one experiment we found that repeated exposure to feedback during grasping reduced the Weber’s law in MSE followed the grasping session. Remarkedly, we also found that indicating the accuracy of an MSE task through explicit visual feedback dramatically reduced scalar variability. We conclude that sensory feedback plays a central role in the reduction of scalar variability, regardless of any functional distinction between perception and action tasks.

**Grant:** National Science Fundation (#BCS 1827550)

## 

**Keywords:** sensorymotor, goal-directed action, perception and action

## Poster Session 2 (July 30, 2019)

## Cue Categories but Not Cue Identities Modulate the Sequence Effects of Cueing Paradigm

**Qian Qian^1^, Chen Cai^1^, Yunfa Fu^1^, Miao Song^2^ and Keizo Shinomori^3^**

^1^Kunming University of Science and Technology

^2^Shanghai Maritime University

^3^Kochi University of Technology

## 

An influence of previous cueing status on the current cueing RTs has been found in symbolic cuing tasks: VV<IV<II<VI, while IV represents the RTs of a valid trial preceded by an invalid trial. As a result, cueing effects are larger after a valid trial than after an invalid trial. This sequence effect has been explained by some researchers as feature integration of low-level features of cues and targets. The present study investigated the influence of cue identity and cue category on the sequence effect. In experiment 1, two different arrow cues (along with different targets) pointed horizontally or vertically to the possible target locations, respectively. In experiment 2, arrow cues and gaze cues were tested instead of two different arrow cues. Sequence effects were not totally abolished when both stimuli (two different arrows along with different targets) and cueing axes (horizontal or vertical) were manipulated in Experiment 1, but it was indeed abolished when different cue categories (gaze and arrow) were presented as cues in Experiment 2. The findings support the task-file theory which considers sequential modulations of attentional tasks as multi-level processes within specific task contexts, rather than the feature-integration hypothesis.

**Grant:** none

## 

**Keywords:** visual attention, attention orienting, cueing effects, sequence effects

## Does Attribute Amnesia Occur with the Presentation of Complex, Meaningful Stimuli? The Answer Is “It Depends”

**Jiahan Yu, Yingtao Fu, Ping Zhu, Wei Li, Jifan Zhou, Mowei Shen and Hui Chen**

Department of Psychology and Behavioral Sciences, Zhejiang University

## 

Attribute amnesia (AA) refers to the phenomenon whereby participants fail to report a salient attribute of a simple stimulus (e.g., the color or identity of a target letter) on which their attention has just been focused during a prior task. The current study sought to explore boundaries of AA by investigating whether it persists when participants encounter complex, meaningful stimuli (e.g., pictures) that hold an advantage in cognitive processing and memory. In Experiments 1a–1d, we examined whether AA was observed with different types of complex stimuli. In Experiments 2a–b, 3a–b we linked the type of stimuli (simple vs. complex and meaningful stimuli) to the other two potential boundary factors of AA (i.e., repetitiveness of target stimulus and set effects of Einstellung) to see whether there were interactions between these boundary factors. The results demonstrated that AA persisted for complex stimuli in a typical AA paradigm wherein participants encountered many trials and the targets were repeated across trials. However, this effect appeared only for simple stimuli when target stimuli were never repeated through the experiment, or when the surprise test appeared on the first trial of the experiment. These findings have crucial implications in understanding the boundaries of AA.

**Grant:** NNSF (No.31771201); National Science Foundation for Distinguished Young Scholars of Zhejiang Province, China (No. LR19C090002); Humanities and Social Sciences Foundation of the Ministry of Education of China (No.17YJA190001)

## 

**Keywords:** working memory, attention, expectation

## The Correspondence Between Monkey Visual Areas and Layers in DCNN Saliency Map Model for Representations of Natural Images

**Nobuhiko Wagatsuma^1^, Akinori Hidaka^2^, Hiroshi Tamura^3,4^**

^1^Toho University, Faculty of Science

^2^Tokyo Denki University, School of Science and Engineering

^3^Osaka University, Graduate School of Frontier Biosciences

^4^Center for Information and Neural Networks (CiNet)

## 

Recent saliency map models based on deep convolutional neural networks (DCNNs) represent the highest performance for predicting the location of attentional selection and human gaze. However, the relationship between artificial and neural representations for determining attentional selection and predicting gaze location has not been unveiled. In order to understand the mechanism of the saliency map model based on DCNN and the neural system of attentional selection, we investigated the correspondence between layers in DCNN saliency map model and monkey visual areas about representations for natural images. We compared the characteristics of the responses on each layer of DCNN saliency map model to that of neural representation on visual areas V1, V4 and IT. Irrespective of the level of the DCNN layer, the characteristics of responses on the DCNN saliency map model were consistent with that of neural representation in the primary visual area V1. Intriguingly, we found marked peaks of correspondence of V1 at early-level and higher-intermediate-level layers of saliency map model based on DCNN. These results suggested that the neural representations in V1 played an important role for determining saliency mediating attentional selection and for predicting human gaze location.

**Grant:** KAKENHI, Japan (no. 17K12704)

## 

**Keywords:** Deep Convolutional Neural Networks, Saliency Map, Visual Areas

## Taller Individuals may See our Visual World Better than the Rests: Influence of Different Eye-Heights on Visual Short-Term Memory Performance

**Yageum Ka^1^, Hyeonbin Gwon^2^, Juyeong Lee^3^ and Joo-Seok Hyun^1^**

^1^Department of Psychology, College of Social Sciences, Chung-Ang University

^2^School of Computer Science and Engineering, College of Computer Science and Software, Chung-Ang University

^3^School of Electrical Electronics Engineering, College of ICT Engineering, Chung-Ang University

## 

Height differences between tall and short individuals may derive different visual experiences against the same visual scene. Based on this idea, the present study examined the influence of different eye- heights on visual short-term memory (VSTM) performance for a simplified visual scene. In the experiments, a memory array of 3 or 6 colored boxes was displayed in each trial, and participants had to remember their colors. The boxes were placed across different depth positions selected under one of three linear perspectives representing views from different eye heights. After the memory interval of about 1 second, the same array or an array with one of the boxes changing its color was displayed as a test array upon which participants had to report presence or absence of the color change. The results showed a substantial drop in change detection performance in the lowest eye-height condition if the array set size was 6, but showed no such drop if it was 3. The results indicate that, as the eye-height became lower, sensory interference among memory items increased to impair VSTM encoding process. They further suggest that different eye-heights have a clear influence on the cognitive process subsequent to VSTM encoding process.

**Grant:** This work was supported by the National Research Foundation of Korea Grants funded by the Korean Government (NRF- 2016S1A5A2A01026073 & NRF-2017R1D1A1B03033965)

## 

**Keywords:** visual short-term memory, visual perception, change detection, individual difference

## Comparison of Attentional Bias to Negative and Positive Stimuli Between People with and without Sleep Disturbance Using the Dot Probe Task and the Implicit Association Task (IAT)

**Siyung Chin, Yung Jae Suh and Kyong-Mee Chung**

Yonsei University

## 

The purpose of this study is to compare the attentional bias of negative stimuli between people who self-reported mild, severe, or no sleep disturbance. A total of 52 participants were included in the study. Among 51 recruited participants (aged 18-65), 21 people reported having mild or severe sleep disturbance symptoms, while 30 people reported having no sleep disturbance symptoms. In order to compare the attentional bias to negative and positive stimuli between the two groups, the participants completed a pictorial dot-probe task under two conditions, Neutral-Sleep positive and Neutral-Sleep negative, and a word Implicit Association Task(IAT) under two conditions, Me-Sleepy vs. Others-Active and Me-Active vs. Others-Sleepy. An independent t-test was conducted to determine the differences of reaction time on each condition of the dot-probe task and the IAT. As a result, there was a significant difference between the two groups in the IAT, while there was no significant difference between the two groups for the dot probe task. The results implied that people who had sleep disturbance symptoms, spend less time categorizing sleep relate words with self. The study implied that people may have an attentional bias with words, rather than pictures, in a self-reporting setting.

**Grant:** none

## 

**Keywords:** attention, bias, stimuli, sleep disturbance

## The Effect of Representation Structures on Visual Working Memory Retrieval

**Pengpeng Liu, JiFan Zhou and MoWei Shen**

Department of Psychology and Behavioral Science, Zhejiang University

## 

Visual information is encoded into structured mental representation to enter visual working memory. The current study examined the effect of representation structures on the retrieval process. Participants were required to memorize a colored image that has several parts with a hierarchical structure, and retrieve two parts of the image in a specified order. By manipulating the structural distance between two parts, we found that the retrieval performance of the second part decreased with the distance to the first part. And this effect could not be triggered by merely priming of the first part, indicating that retrieval of the first part is necessary for the effect of mental structure to occur on the second part. Those findings suggested that the retrieval of information stored in visual working memory is based on the structured representation, the retrieval performance could be impaired when the retrieval structure is inconsistent with the structure of representation.

**Grant:** none

## 

**Keywords:** visual working memory, mental representations, hierarchical structures, memory retrieval

## Effects of Visual Working Memory on Individual Differences in Echolocation Performance

**Tomoki Maezawa and Jun Kawahara**

Department of Psychology, Hokkaido University

## 

Echolocation is a method to localize objects based on the reflection of sound. Sighted individuals can use echolocation following suitable training, while the performance of echolocation shows considerable individual difference. Several factors, such as working memory and processing of visual images are involved in this difference;(e.g., Ekkel, van Lier, & Steenbergen, 2017). Thus, visual spatial component of working memory should be involved in echolocation. The present study examined the association between echolocation performance in sighted individuals and visual working memory capacity. Subjects performed an object detection task by using echolocation at different target distances (20, 30, 40, or 50 cm) across three-separated days, and completed a visual working memory task in the third day. The results indicated that the subjects improved the performance of the detection task across the initial two days, while the performance showed large individual differences. We found a significant positive correlation (r = 0.62) between the performance and the visual working memory capacity. The present results reflect that visual spatial processing is involved in an object detection using echolocation for sighted individuals. We suggest that visual working memory capacity would be predictive of higher performance of the detection task.

**Grant:** none

## 

**Keywords:** visual working memory, echolocation, individual difference

## Long-term Retention of Multi-Attribute Stimulus-Response Mapping

**Yumiko Fujii^1^ and Hiromi Morita^2^**

^1^Graduate School of Library, Information and Media Studies, University of Tsukuba

^2^Faculty of Library, Information and Media Science, University of Tsukuba

## 

The human visual system processes different attributes of an object separately and then integrates them to elicit a specific response. Ishizaki et al. (2015) proposed a paired-attribute model in which bound feature pairs are units of multi-attribute stimulus-response associations. However, our previous study of stimulus-response mapping suggested that color and shape are bound and associated with a response, whereas location is not, when added to the association; consequently, we proposed the location-singleton model. The present study aims to confirm that location is not bound with other attributes in the retention stage after learning, as observed for the learning stage. One week after participants learned the mapping of eight stimuli comprising color (red/green), shape (circle/triangle), and location (left/right) to four responses, they were tested with the same mapping. As a result, they could respond as accurately and quickly as they did immediately after learning. Moreover, their responses to the color–location and shape–location pairs were more difficult than their response to the color–shape pair as observed immediately after learning. The results suggest that the location-singleton model is applicable to the representation of multi-attribute stimulus–response mapping during the retention period.

**Grant:** JSPS KAKENHI Grant Number 17K00200

## 

**Keywords:** experimental psychology, memory, learning, feature integration

## The Interaction of Grouping and Salience in Visual Search: The Electrophysiological Evidence for the Collinear Masking Effect

**Li Jingling^1^, Chia-huei Tseng^2,3^, Shin Ono^2,3^, Satoshi Shioiri^2,3^**

^1^Graduate Institute of Biomedical Sciences, China Medical University

^2^Research Institute of Electrical Communication, Tohoku University

^3^Graduate School of Information Sciences, Tohoku University

## 

Visual search is impaired when neighboring items to a target are aligned (i.e. collinear visual search masking, Jingling and Tseng, 2013). To elucidate its involved mechanisms, we measured the event-related potential when 16 participants completed the search task. The display was filled with the same vertically- (or horizontally-) oriented bars, except for one distractor column consisting of bars orthogonal to the others. The bars in the distractor column were grouped by collinearity or by similarity at equal chance, and the distractor location was task-irrelevant. The target, a tilted bar, was located at three possible locations with equal likelihood (1/3 overlapping with distractor column and 2/3 not). Results showed that the distractor column, in both collinear and similarity grouping, elicited a strong P1 component at around 100 ms and a Pd component around 200 ms after display onset. This indicated that the well-grouped distractor was salient but suppressed during search. The target, regardless of overlapping with distractor or not, elicited an N2pc component at 200–300 ms, suggesting focused attention on target processing. Interestingly, targets overlapped with a collinear distractor reduced P3 amplitude than non-overlapping conditions, implying additional cognitive processing other than suppression contributed to the collinear search impairment.

**Grant:** FY2018 RIEC Nation-wide Cooperative Research Projects, MOST106-2420-H-039-002-MY3

## 

**Keywords:** visual search, ERP, perceptual grouping, N2pc

## Gaze Cues Stored in Working Memory Trigger Automatic Attentional Orienting

**Haoyue Ji^1,2^, Yi Jiang^1,2^, Li Wang^1,2^**

^1^Institute of Psychology, Chinese Academy of Sciences

^2^University of Chinese Academy of Sciences

## 

Previous research has shown that social cues including eye gaze can readily guide our focus of attention. Here, we demonstrate that merely maintaining social cues in working memory (WM) can elicit a similar attentional orienting effect. Using the delayed-match-to-sample paradigm combined with the dot-probe task, we found that holding a face image with task-irrelevant averted eye gaze in WM could automatically induce attentional orienting to the gazed-at location. Importantly, such WM-induced attention effect could not be explained by the perceptual attentional process, because the identical gaze cues that were only passively viewed and not memorized in WM didn’t trigger attentional orienting beyond the time window of typical social attention. Furthermore, non-social cues (i.e., arrows) held in WM also failed to elicit the automatic attentional orienting effect. Taken together, the current study provides clear evidence that social but not non-social cues stored in WM can guide spatial attention akin to that with actual presentation of stimuli, and highlights the uniqueness of brain mechanisms underlying social attention as compared to non-social attention.

**Grant:** none

## 

**Keywords:** social attention, working memory, eye gaze, arrows

## How Professional Stereotype and Warning Work in Episodic Memory: Modulation of Morality

**Minye Li and Aiqing Nie**

Department of Psychology and Behavior Science, Zhejiang University

## 

Previous research on stereotypes indicates that in general, the effect of source memory of the contents conforming stereotypes is better. However, negative or immoral contents that violate stereotypes come with even better source memory. There has been no systematic study to illustrate the roles of ethics on the influence of stereotypes on source memory. We assume that in contrast to the situation of conforming professional ethics, violating them will result in better source memory. Therefore, we add the professional ethics factor in our study to explore its regulation effect on the influence of stereotypes on source memory. The results show that: (1) contents that conform professional stereotypes have better source memory; (2) contents that conform professional ethics have better memory; (3) there has been interacting effect between professional ethics and professional stereotypes and professional ethics have a regulation effect.

**Grant:** none

## 

**Keywords:** source memory, social cognition, stereotype, morality

## Attentional Cueing Effect by Pointing Hand and Other Cues: Differences and Interactions

**Ryosuke Niimi**

Niigata University

## 

Images of pointing hand act as attentional cue and guide spatial attention. Is this attentional effect identical to that of other cues (arrows, eye gaze)? Experiment 1 (standard Poster paradigm with pointing hand and arrow cues) demonstrated that pointing hands, either upright or inverted, yielded cueing effect comparable with arrow cues. In Experiment 2, a pair of pointing hand and eye gaze served as cue stimuli. Validity of the hand cues and eye gaze cues was manipulated independently; the hand and eye gaze were in the same direction in some trials, while in other trials they were conflicting with each other. Results showed that the cueing effect of eye gaze almost disappeared if the pointing hand cue was invalid, and vice versa. The cueing effect by pointing hands is basically equivalent with that of arrows, while it interacts with gaze cueing effect.

**Grant:** This work was supported by a grant for InterdisciplinaryJoint Research Project from Brain Research Institute, Niigata University.

## 

**Keywords:** attention, body perception, object perception

## The Visual Cocktail Party Problem: Enhancement of Visual Selective Attention Through Phase Entrainment to Auditory Streams

**Xiangyong Yuan^1,2^, Xilei Zhang^1,2,3^, Ying Wang^1,2^, Yi Jiang^1,2^**

^1^State Key Laboratory of Brain and Cognitive Science, CAS Center for Excellence in Brain Science and Intelligence Technology, Institute of Psychology, Chinese Academy of Sciences

^2^Department of Psychology, University of Chinese Academy of Sciences

^3^School of Biological Sciences and Medical Engineering, Southeast University

## 

One’s auditory selective attention under noisy conditions, such as in a cocktail party, can be facilitated by congruent visual inputs. Here we demonstrate that in a “visual” cocktail party situation, visual selective attention can be significantly enhanced through phase realignment to a concurrent auditory stream. In an EEG experiment, participants were asked to discriminate a visual object among distractors, in the meanwhile they were presented with an auditory stream that changed either congruent or incongruent (i.e., in- or out-of-phase) with one of the visual features embedded in the target object. We found that the presentation of a congruent auditory stream not only improved the target discrimination accuracy but also increased the power of the parieto-occipital neural oscillations specifically entrained to the auditory-congruent feature of the target object. Moreover, the functional connectivity between the parieto-occipital and fronto-central regions, which reflects the phase coherence between audiovisual oscillations, could significantly predict the behavioral discrimination accuracy when accompanied by congruent sounds. Taken together, our findings suggest that a congruent auditory stream can enhance visual selective attention by boosting the neural entrainment through strengthening the phase coherence between the auditory and visual neural activities.

**Grant:** none

## 

**Keywords:** multisensory integration, selective attention, neural oscillation

## Early VEP Reflects Affective Priming Modulated by Temporal Attention

**Takuya Imani, Hikaru Ishisaka, Tomokazu Urakawa, Yuki Kurita and Osamu Araki**

Department of Applied Physics, faculty of Science, Tokyo University of Science

## 

Orienting attention to specific timing of an upcoming visual event has been reported to enhance visual processing of the event. By recording visual evoked potentials (VEPs), the present preliminary study tried to elucidate temporal profile of visual processing relevant to the affective priming effect enhanced by temporal attention (tAPE). In the experiment, we employed a stimulation paradigm of subliminal affective priming for face. At the beginning of each trial, participants were informed about the presentation timing of primer with a visual cue (Short or Long). A primer was either a happy or a fearful face, and participants then judged the affect of a neutral face target following the primer. Our results for behavioral data showed that tAPE was not consistently observed across participants. However, in terms of the inter-individual differences in behavioral and VEP data, we found, for the trials with happy primer, that a diminishment of N75 (a negative VEP component at a latency of around 75 ms) significantly correlated with an increase of tAPE across participants. This finding suggests, by focusing on interindividual differences, that the affective priming effect enhanced by temporal attention is reflected as early as about 75 ms following the primer’s onset.

**Grant:** none

## 

**Keywords:** temporal attention, VEP, consciousness

## The Effect of Aging on Gaze-Induced Inhibition of Return

**SyuanRong Chen and Li Jingling**

China Medical University

## 

Inhibition of return (IOR) is an inhibition mechanism of attention orienting. Previous studies showed that aging did not alter the size of IOR induced by peripheral cues (the tradition IOR). The goal of this study aimed to explore how aging alter the size of gaze-induced IOR. Two experiments were carried out, each collected 30 younger (age ranged from 20 to 30 years old) and 30 older adults (older than 65 years old), in total 120 participants. Both experiments were localization tasks using photographs of real faces to introduce gaze cues. The cues validity was set as uninformative. Experiment 1 followed the classical cue-target paradigm and Experiment 2 used the target-target paradigm. Results showed that both younger and older adults can reach high accuracy (above 95%). Younger adults showed facilitation at 200 ms and IOR at 2400 ms SOA. Nevertheless, older adults did not have statistical significant differences between valid and invalid trials in any SOA conditions. Since traditional IOR did not altered with aging, our data thus did not support for the hypothesis that gaze-induced IOR was elicited by the same inhibition mechanism from that elicited by periphery cues.

**Grant:** MOST106-2410-H-039-001-MY2

## 

**Keywords:** inhibition of return, gaze cue, age, the cueing effect

## Attentional Shift to The Newer Object in the Preview and Simultaneous Search

**Takayuki Osugi**

Yamagata University

## 

When some distractors (old items) appear before others (new items) during an inefficient visual search task, observers exclude the old items from the search (preview benefit). Previous studies demonstrated that preview benefit occurred when items were presented in two-time steps (i.e., the first and second displays). However, it remains unclear whether observers can shift their attention to newer object if the target appeared after the onset of the other new items; the target includes in the third display. To test this, we compared search times for the target in the third display under the preview condition and the simultaneous condition in which no items were presented in the first display (i.e., blank display). In two- thirds of the trials, the target was included in the second display, and it appeared in the third display on the remaining trials. The results indicated that preview benefit occurred when the target appeared in the second display, but target detection in the third display was slower under the preview condition relative to the simultaneous condition. It suggests that attentional shift to the newer target requires more time if search items were already divided into two groups by temporal information.

**Grant:** JSPS KAKENHI Grant Number 17K13960

## 

**Keywords:** Visual search, Inhibition, attentional control

## Working Memory Is Corrupted by Strategic Changes in Search Templates

**Garry Kong, Jessica Meehan and Daryl Fougnie**

New York University Abu Dhabi

## 

When searching for an item, we often conjure an image of that item to act as a search template. This search template is thought to be stored as, and is no different from, a visual working memory representation. Evidence for this primarily comes from studies demonstrating that the contents of working memory influences search behavior. However, whether this interaction applies in both directions is unknown. Here, we present evidence that changes in search templates can influence working memory. Participants remembered the orientation of a target line and on some trials (75%) performed a visual search for that orientation, but on other trials (25%) recalled the target’s orientation. Critically, we manipulated the search template by introducing a predictable context—distractors in the search task were always clockwise (or counterclockwise) from the search target. We found that the context biased search away from the distractors (i.e., counterclockwise), t(27) = 15.00, p<.001, Cohen’s *d* = 2.83, and crucially, also similarly biased orientation reports during recall, t(27) = 2.97, p = .006, Cohen’s *d* = 0.56. This demonstrates that working memory and search templates were not held as separate, isolated representations, supporting the conclusion that search templates are equivalent to memory representations.

**Grant:** none

## 

**Keywords:** Visual Working Memory, Visual Search, Attention, Psychophysics

## Involvement of Visual Mismatch Negativity in Access to Visual Consciousness

**Yuki Kurita, Tomokazu Urakawa and Osamu Araki**

Department of Applied Physics, Graduate School of Science, Tokyo University of Science

## 

Electroencephalographic brain response to a violation (or a change) of preceding sequential regularity of visual events, called visual mismatch negativity (vMMN), has been well known to reflect automatic visual change detection. A recent study using a modified version of binocular rivalry paradigm reported that the vMMN was evoked even when a violation occurred during binocular rivalry suppression (Jack et al., 2017). In the present study, we report that vMMN’s occurrence under binocular suppression is relevant to rendering an invisible image evoking vMMN visible. In stimulation, we intermittently presented a sinusoidal grating and abruptly changed its orientation by 90 degrees (a violation of preceding visual sequence) during binocular suppression. Under this scheme, vMMN under binocular suppression was recorded and the proportion of perceptual switch from before to after the presentation of the violation was calculated. With a focus on the inter-individual differences in the behavioral index and vMMN’s amplitude, we found that an enhancement of vMMN significantly correlated with an increase of the proportion of perceptual switch across participants. We propose, besides visual change detection in the absence of consciousness, that visual processing underlying vMMN is relevant to triggering access to visual consciousness.

**Grant:** none

## 

**Keywords:** visual mismatch negativity, visual awareness, EEG

## Quick Buildup of Suppression Revealed by Eye-Swapping Technique in Continuous Flash Suppression

**Motomi Shimizu^1^ and Eiji Kimura^2^**

^1^Graduate School of Advanced Integration Science, Chiba University

^2^Department of Psychology, Faculty of Letters, Chiba University

## 

Continuous flash suppression (CFS) can render a static stimulus in one eye invisible by presenting a dynamic stimulus in the other eye. To elucidate the underlying mechanism of CFS, we investigated the effects of the eye of presentation (which eye receives which stimulus) by using an eye-swapping technique. If CFS is mediated by an eye-based process, swapping the stimuli between the eyes should disrupt suppression. To test this possibility, we measured the time it takes for a suppressed stimulus to be detected, with manipulating the frequency of eye swapping. Results showed that the detection time was not much affected and the suppression was strong when the swapping frequency was low (1.2 Hz). However, the detection time was significantly reduced when the swapping frequency was high (3.5 Hz). Further investigation with measuring detectability of a briefly presented target revealed that the suppression was weak just after an eye swap but became stronger by the time of the next eye swap in the 1.2-Hz eye-swapping condition. These results can be understood by the contribution of an eye-based process that exhibits a quick buildup of suppression to CFS.

**Grant:** Supported by JSPS KAKENHI (26285162 & 18K18686)

## 

**Keywords:** binocular vision, continuous flash suppression, psychophysics

## Effects of Eyeblinks on Perceptual Switching during Continuous Flash Suppression

**Ryoya Sato^1^ and Eiji Kimura^2^**

^1^Chiba University Graduate School of Science and Engineering

^2^Chiba University Graduate School of Humanities

## 

Eyeblinks have been reported to modulate perceptual switching during multistable perception, but their reported effects were not unequivocal. This study investigated how different types of eyeblinks, i.e., spontaneous (unintentional) and voluntary (intentional) eyeblinks, modulate perceptual switching during continuous flash suppression (CFS). CFS refers to an experimental paradigm, where a high-contrast dynamic Mondrian stimulus presented to one eye renders invisible a static target stimulus presented to the other eye. In the experiments, we gradually increased target contrast after exclusive dominance of the dynamic Mondrian had been established, and measured the time required for the suppressed target to break through the suppression. Results showed that spontaneous eyeblinks that occurred before target detection increased the detection time, relative to that found on the control trials where no eyeblinks occurred. By contrast, voluntary eyeblinks generated in response to a visual cue shortened the detection time. In the present paradigm, physical blackout, which had comparable timing and duration to eyeblinks, did not significantly affect the detection time. Moreover, a close temporal relation was found between voluntary eyeblinks and target detection; the frequency of target detection was peaked 0.5 sec after the eyeblinks. These findings suggested that extra-retinal signals associated with voluntary eyeblinks induce perceptual switching.

**Grant:** Supported by JSPS KAKENHI (26285162 & 18K18686)

## 

**Keywords:** Eyeblink, multistable perception, awareness

## The Spatial Profile of Interocular Suppression Is Modulated by Mask Speed

**Egor Ananyev^1^, Gerrit Maus^1^ and Po-Jang (Brown) Hsieh^2^**

^1^Nanyang Technological University

^2^Duke-NUS

## 

Interocular suppression is a phenomenon where a salient stimulus in one eye (mask) suppresses visual awareness of another stimulus (target) presented to the other eye. Despite the popularity of the paradigm in the study of unconscious processing, the mechanism of interocular suppression is yet unclear. It is assumed that the interocular suppression is spatially uniform, and that this should not depend on mask speed. To test this assumption, we manipulated mask speed, target speed, and target eccentricity. We found that the masking strength varied with all three factors. Slower masks were more effective in suppressing slow, centrally presented targets. Faster masks were more effective in suppressing fast, peripherally presented targets. To account for this phenomenon, two explanations are considered: neurophysiological dichotomy between parvocellular and magnocellular pathways and a computational model of divisive normalization.

**Grant:** none

## 

**Keywords:** interocular suppression, divisive normalization, motion, binocular rivalry

## ON Response does not Reflect Access to Visual Consciousness: A VEP Study

**Erika Takahashi, Tomokazu Urakawa, Yuki Kurita and Osamu Araki**

Department of Applied Physics, Faculty of Science, Tokyo University of Science

## 

Automatic detection of a visual change in relation to a preceding sequential regularity of visual events is reflected in electroenephalograpgic brain response, called visual mismatch negativity (vMMN). Our recent study using a modified version of binocular rivalry found that vMMN’s occurrence driven by the change under binocular suppression is relevant to rendering an invisible image evoking vMMN visible. In the present study, we further attempted to determine whether ON-response, evoked by a visual change without a preceding sequential regularity, under binocular suppression would also be related to triggering access of a perceptually-suppressed image to consciousness. In stimulation, a sinusoidal grating was presented under binocular suppression and its luminance was abruptly increased. With this scheme of experiment, P1 and N1 (visual evoked potentials, VEPs) were recorded and the proportion of perceptual alternation was calculated. By focusing on the interindividual differences in VEP’s amplitude and the behavioral index as in our preceding study regarding vMMN, our current results showed that each amplitude of P1 and N1 did not significantly correlate with the facilitation of perceptual alternation across participants. We propose that vMMN but not ON response is relevant to triggering access to visual consciousness.

**Grant:** none

## 

**Keywords:** VEP, ON response, binocular rivalry

## Dissociating Conscious and Unconscious Privileges of Temporal Structures in Visual Competition

**Ruichen Hu, Peijun Yuan, Yi Jiang and Ying Wang**

State Key Laboratory of Brain and Cognitive Science, CAS Center for Excellence in Brain Science and Intelligence Technology, Institute of Psychology, Chinese Academy of Sciences; Department of Psychology, University of Chinese Academy of Sciences

## 

Dynamic events with regular changing patterns provide abundant sources of temporal structures in our environment. Among them, we have previously demonstrated that feature- and semantics-based temporal structures, relative to their non-structured counterparts, prolonged the predominance of dynamic information streams during the competition for visual awareness. Here we disentangled the underlying mechanisms for these multi-source facilitation effects, especially regarding their reliance on conscious processing. During binocular rivalry, temporal structures built on dynamic change of visual features held an advantage largely attributable to reduced durations when suppressed from awareness, whereas those emerging from semantic-level regularities benefited mainly from prolonged perceptual durations while dominating awareness. A further experiment using the bCFS paradigm yielded consistent results that only the feature-based structured streams, when initially suppressed, gained privileged access to awareness over the random counterparts, indicating a benefit for unconscious processing. Together, these findings provide evidence for dissociable mechanisms to prioritize different types of temporal regularities for conscious experience. Extraction and utilization of semantic-level temporal structures engage conscious information processing, compared with that of perceptual-level temporal structures may occur even without awareness, pointing to their distinct privileges and functional roles in conscious and unconscious processes.

**Grant:** none

## 

**Keywords:** binocular rivalry, visual awareness, temporal structure, unconscious processing

## Can Semantic Information be Temporally Integrated Under Interocular Suppression? An fMRI study

**Yung-Hao Yang^1,2^, Tsung-Ren Huang^1,4,5^, Su-Ling Yeh^1,3,4,5^**

^1^Department of Psychology, National Taiwan University

^2^Human Information Science Laboratory, NTT Communication Science Laboratories, Nippon Telegraph and Telephone Corporation

^3^Graduate Institute of Brain and Mind Sciences, National Taiwan University

^4^Neurobiology and Cognitive Science Center, National Taiwan University

^5^Center for Artificial Intelligence and Advanced Robotics, National Taiwan University

## 

Awareness of visual stimuli in one eye can be interocularly suppressed by high-contrast masks from the other eye; such interocular suppression provides a powerful tool to examine the function of consciousness along visual pathway. We used fMRI to investigate whether multiple words presented sequentially can be unconsciously integrated into higher-level meanings. Temporal sequences of four characters were manipulated to form either a Chinese idiom or a meaningless random sequence. Word sequences from one eye were accompanied with or without masks from the other eye to render the unaware or aware condition, respectively. BOLD signals showed reliable activation in bilateral fusiform areas regardless of temporal sequence and visual awareness, suggesting processing of single word form. Critically, despite idiom judgment task showed chance level performances in the unaware condition, the activation patterns of contrast between random and idioms were nevertheless modulated by awareness state: In the unaware condition, Chinese idioms generated higher activation than random sequence in bilateral inferior frontal gyrus (IFG), while the same contrast in the aware condition showed higher activation in bilateral superior temporal gyrus (STG). The distinct neural mechanisms between unconscious and conscious processes suggest that different semantic processing was modulated by conscious states.

**Grant:** none

## 

**Keywords:** Semantic integration, Interocular Suppression, unconscious processing, functional magnetic resonance imaging

## Establishing a Massive-Report Paradigm to Reveal the Richness of Consciousness

**Naotsugu Tsuchiya^1^, Jasmine Walter^1^, Alon Loeffler^1^, Zhao Koh^1^, Jeroen van Boxtel^1^ and Shinji Nishimoto^2^**

^1^Monash University

^2^Osaka University

## 

While conscious perception of the world seems rich, this richness has proven difficult to verify in traditional experiments. One of the problems with traditional psychophysical studies is that they used tasks which primarily reflected participants’ capacity for recalling specific items of interest (e.g., letters). Here, we implemented a novel approach for gauging experiential richness, the Massive-Report paradigm to overcome many limitations of previous experiments. This paradigm allows people to demonstrate the richness of their experience to a far greater extent than previous methods. Participants viewed briefly-displayed natural images (133 ms) and were asked to demonstrate their detailed experience of the image through answering a Massive series of questions (640+). Each question consisted of a word which may or may not be an appropriate description of some component of the image. Participants could express what they experienced in a way that was at least 35 times richer than in traditional paradigms. The Massive-Report paradigm has the potential to bring significant revision to our view on the nature of consciousness.

**Grant:** Australian Research Council Discovery Project DP180100396 and DP180104128

## 

**Keywords:** psychophysics, natural scene, consciousness

## Perceptual Decision for Average Orientation over Space and Time

**Ryuto Yashiro^1^, Hiromi Sato^1^, Takumi Oide^2^ and Isamu Motoyoshi^1^**

^1^Department of Life Sciences, The University of Tokyo

^2^Department of Integrated Sciences, The University of Tokyo

## 

The visual system can rapidly compute spatial statistics in stochastic stimuli. The present study investigated mechanisms underlying average-orientation discrimination over space and time. Six observers viewed a dynamic texture (4 or 32 frames of texture patterns with Gabor elements serially presented at 30 Hz) and indicated whether its average spatiotemporal orientation was tilted clockwise or counter-clockwise. The spatial mean orientation of each frame was varied according to a Gaussian distribution with a particular mean and a temporal SD of 0–8 deg. Element orientation in each frame was varied according to a Gaussian distribution with a spatial SD of 0–16 deg. We found that discrimination thresholds increased as a function of spatial SD if temporal SD was small but remained nearly constant if temporal SD was large. Reverse correlation analysis revealed that observers put emphasis on the last few frames when judging spatiotemporal average. The results are inconsistent with visual mechanisms that integrate local information equally over space and time but support distinct spatial and temporal mechanisms: a sensory mechanism that rapidly averages spatial orientation and a perceptual decision mechanism that accumulates averaged signals over time.

**Grant:** Supported by the Commissioned Research of NICT, and by JSPS KAKENHI JP15H05916, JP15H03461 and JP16J07259.

## 

**Keywords:** perceptual decision making, psychophysics

## Reverse-Correlation AnalysIs of Real-Time Perceptual Decision for Dynamic Visual Stimuli

**Hironori Maruyama, Hiromi Sato, Ryuto Yashiro and Isamu Motoyoshi**

Department of Life Sciences, The University of Tokyo

## 

To understand how humans utilize information until the moment when they make a decision for visual events, the present study applied a reverse correlation analysis to the behavioral response in a reaction-time task for stochastic stimuli. The observers viewed a dynamic texture (15 Hz frame rate) composed of Gabor elements whose orientation randomly varied according to a Gaussian noise both in space and time. During the stimulus presentation, they pressed a button as immediately as possible to indicate whether its average spatiotemporal orientation was tilted clockwise or anti-clockwise. We calculated logistic regression coefficients of the observer’s response to the spatial mean orientation at each temporal frame with respect the moment of reaction time, which ranged from ∼500 to ∼2000 ms. The analysis revealed a sharp peak in the regression coefficient at 300–400 ms before the manual response. The peak was less profound for trials with longer RTs. The subsequent analysis also showed that within a temporal period at around this peak, SD of orientation was lower than the other periods. These results indicate that, at least under urged situations, human perceptual decision is triggered dominantly by information 200–400 ms before behavioral response.

**Grant:** Supported by the Commissioned Research of NICT, and by JSPS KAKENHI JP15H05916, JP15H03461 and JP16J07259.

## 

**Keywords:** psychophysics, descion making

## Analyzing Eye Fixation Patterns to Explore the Factors Affecting Visual Balance in Visual Art with Depth Perspective

**Cong-Wei Ho^1^, Shwu-Lih Huang^1,2^**

^1^Department of Psychology, National Chengchi University

^2^Research Center for Mind, Brain & Learning, National Chengchi University

## 

It is well known that even distribution of visual weight in a frame will achieve visual balance. However, this phenomenon has not been thoroughly studied. Also little research has been done to examine the factors that influence visual weight empirically. In this study, we aimed to verify Arnheim’s (1974) claim that an element with greater depth will carry more visual weight in a picture. Using eye-tracker as a tool, we conducted our research on visual balance first with real visual art—photography, and gradually simplified the research stimuli to rule out confounding variables. Normalized eye fixation measures were used to estimate the visual weight. The visual balance of three kinds of colorful photographs with two objects was compared, including (1) larger near-object and small far-object (Arnheim-consistent), (2) small near-object and larger far-object (Arnheim-violated), (3) unbalanced control. We found that both Arnheim-consistent and Arnheim-violated photographs can achieve visual balance. Larger visual weight of far-object than near-object was observed in the Arnheim-consistent condition, but it reversed in the Arnheim-violated condition. Our results implied that Arnheim’s balance theory of spatial depth is not totally complete. Background knowledge of the real size of objects also is an important factor to influence visual balance.

**Grant:** none

## 

**Keywords:** eye-tracker, visual balance, depth perspective, art

## Bayesian Optimal Decision Making in Risk-Return Trade-off under Spatiotemporal Motor Variability

**Qirui Yao and Yutaka Sakaguchi**

Graduate School of Informatics and Engineering, University of Electro-communications

## 

Are humans capable of achieving optimal motor planning in controlling the risk-return tradeoff under their own motor variability? Two experiments adopting different motor decision tasks were conducted to tackle this problem. In the temporal task, participants were asked to reproduce the target interval as accurately as possible by mouse click on a semi-circled ring. The spatial task required participants to click on an arched ring as proximate to its midline (hidden from view) as possible within a predefined time window. In both tasks, participants received a “gain” according to the task performance determined by temporal/spatial accuracy, where the steepness of gain function (high-risk high-return vs. low-risk low-return) was modulated by the click position. Participants were asked to choose the risk-return configuration (i.e., the click position) so as to maximize the gain. Performance of individual participants was evaluated by a Bayesian decision-making model. We found that participants’ performance reached and maintained the Bayesian optimality within the experimental session of 210 trials in a manner of balancing the risk/return, both in the temporal and spatial tasks. The present result indicates that humans can achieve the optimal motor planning for risk-return control under spatiotemporal motor variability.

**Grant:** none

## 

**Keywords:** Bayesian Modeling, Movement Planning, Decision Making

## Impacts of Spatial and Verbal Cognitive Activities on SNARC effect

**Shena Lu^1^, Yongchun Cai^1^, Shuangxia Li^1,2^**

^1^Department of Psychology and Behavioral Science, Zhejiang University

^2^Institute of Education, Henan Normal University

## 

The spatial numerical association of response codes (SNARC) refers to the observation that responses are faster for small numbers (1–4) with left-side response buttons and faster for large numbers (6–9) with right-side response buttons. The underlying mechanism remains controversial. The SNARC effect can be interpreted either as the association between number and space (i.e., small and large numbers are associated with left-side and right-side spatial locations, respectively) or as the association between number and linguistic concept (i.e., small and large numbers are associated with the “left” and “right” concepts, respectively). Here, participants were required to perform either a spatial rotation task or a verbal fluency task. The SNARC effect was measured both before and after this task. If the SNARC effect was due to the spatial/conceptual association, it would be enhanced after the spatial rotation/ verbal fluency task accordingly. When the SNARC effect was measured by the magnitude comparison task (numerical information was explicitly processed), it was enhanced only after the spatial task. When the SNARC effect was measured by the parity judgment task (numerical magnitude was implicitly processed), it was enhanced after both the spatial and verbal tasks. Results suggested that the SNARC effect was mainly modulated by spatial operations.

**Grant:** This work was supported by Project of Humanities and Social Sciences, Ministry of Education of China (18YJA190011, 18YJA190001), Zhejiang Provincial Natural Science Foundation of China (LY18C090001), National Natural Science Foundation of China (61876222)

## 

**Keywords:** numerical cognition, the spatial numerical association of response codes effect (SNARC)

## Drifting Head Movement in a Micrometer Order during Visual Fixation

**Yasuto Tanaka^1^, Hiroyuki Fujie^2^ and Taro Maeda^3^**

^1^Neuromathematics Laboratory

^2^Miki Holdings

^3^Osaka University

## 

Micro-meter order drifting head movement is found during visual fixation. The video oculography was used to detect miniature head movement together with eye movement. Three markers on the forehead together with left and right corners of each eye were shot by the high speed camera with 300 Hz. The image was processed by the SURF algorithm yielding sub-micrometer resolution. Affine transformation was used to match the three data points that determines the vector components of head motion. Subjects were asked to move their head within a tiny amount towards the right, left, upper, and lower direction from the central fixation. Visual feedback was given by a red central fixation spot. The motion trajectories at the foreheads and two corners of eye exhibited the directions of left, right, up, and down with the velocities of 1.9 deg/sec, with the movement vector size 5.75+/−0.06 deg(average+/-SE) , corresponding saccadic elements of eye. During the stopping period (targeting location and back-fixation position), a slower component was found with 13 Hz frequency with the magnitude 0.5+/−0.09 arcmin(average+/−SE). The trajectory patterns were topologically identical between the position, indicating that the subject shifted their head intentionally. Such drifting head movement is to be utilized for exact fixation, attentional orienting, and vestibular ocular reflex (VOR) in a micrometer scale together with eye movement.

**Grant:** none

## 

**Keywords:** visiomotor coordination, head movement, eye movement

## The Effect of Word’s Visual Complexity Distribution on Saccade Targeting in Reading Chinese Sentences

**William Alberto Cruz Molina^1^, Jie Li Tsai^2^ and Chia Hsing Chen^3^**

^1^National Cheng Kung University

^2^National Cheng Chi University

^3^National Cheng Chi University

## 

Previous studies about the factors that influence the decision about where to move the eyes next suggest a strong influence from low-level features; some studies also assume that this decision involves the computation of a saccade target before the oculomotor program is executed. In order to test whether the distribution of visual components within Chinese words influence the saccade targeting mechanism, we devised a new parameter that reflects the distribution of visual information along 2-character words’ area: the Visual Complexity Distribution (VCD) index. Three groups of words with a marked VCD index were identified and embedded in natural sentences; the eye movement of Chinese native speakers was recorded in order to contrast first-pass duration, position and probability eye movement measures between conditions. The experimental effects were estimated through contrast between conditions using Linear Mixed Models, providing evidence about the VCD index’s influence on both, the decision about when and where to move the eyes next. These results indicate that the luminance patterns within 2-character Chinese words can influence the specification of a saccade target when those words are about to be fixated as well as modulate the fovea load when those words are currently fixated.

**Grant:** Embedding the VCD as regressor in Linear Mixed Models for different oculomotor measures resulted significant for some conditions

## 

**Keywords:** Saccade Targeting Mechanism, Initial Landing Position, Reading Chinese

## AnalysIs of the Relationship Between Eye Movement and Performance during Dance Rotation Using a Wireless Eye Movement Measurement Device

**Takuya Sarugaku, Yasuyoshi Kobayashi, Reiko Koyama, Shinya Mochiduki and Mitsuho Yamada**

Tokai University

## 

Sports science is attracting a lot of attention along with the Tokyo Olympics and Paralympics near at hand in 2020. In particular, analyzing gaze movement during sports is considered to commit insights into the athlete’s excellent performance, and is supposed to be useful for coaching athletes. For gaze analysis during sports, particularly those sports in which the athlete moves vigorously, a wireless eye movement measurement device is indispensable. Previous eye movement measurement devices were expensive and often difficult to use outside of a laboratory. In recent years, compact and easy-to-use measuring devices have been put into practical use, but these experimental devices cannot operate the devices after the start of measurement. For athletes who exercise and move vigorously, the calibration may shift during the experiment, so it can be said that the experimenter can remotely control the device during the measurement is ideal to analyze eye movement while an athlete move quickly. We have developed a wireless eye movement measurement device that can be operated remotely while the athlete is wearing it. In this paper, we describe the outline of this wireless measurement device and the relationship between head, body and eye movement during dance rotation measured using the device.

**Grant:** none

**Keywords:** Wireless eye movement measurement device, Sport, Gaze movement

## A Real Time Fatigue Evaluation with Standard Deviation of the Gaze Point During Calculation Task

**Yuki Kurosawa, Shinya Mochiduki, Yuko Hoshino and Mitsuho Yamada**

Tokai University

## 

In Japan, it is said that the eyes are the window of the mind. The eyes condition is often used as an indicator of feeling and physical condition. Among eye movements, the small involuntary eye movements that continually occur even during gazing are known to be particularly important for the vision. In addition, these movements can be sorted into micro-saccades, drifts and tremor components, also a visual image cannot be maintained without these movements. However, few reports have measured changes in small involuntary eye movements during a task in real time and analyzed them. Therefore, in this study, we measured the eye movements within the gaze points while the subjects are performing a calculation task and examined the relationship between the standard deviation of eye movements and the critical fusion frequency (CFF) value, which is used as an indicator of fatigue. As a result, it was shown that the change in the eye movements within gaze point can be an effective parameter to evaluate the levels of fatigue and concentration.

**Grant:** none

## 

**Keywords:** Eye movement, fatigue, CFF

## Examination of Physiological Evaluation by Investigation of Influence of Movies on Eye Movement and CFF

**Takahide Otomo^1^, Shinya Mochiduki^1^, Eriko Ishii^2^, Yuko Hoshino^1^ and Mitsuho Yamada^1^**

^1^Tokai University

^2^Kagoshima Prefectural College

## 

Nowadays, we can enjoy many video contents such as movies in various ways with advancement of information technologies and an opportunity and time for watching images was increased. Therefore, it can be said that analysis of influence from watching video contents on eye movement is an important task. As a major way to analyze influence when watching movies, measurements of electroencephalogram are often used and reported. However, few reports have been studied changes in eye movement caused by watching video contents. Research using various parameters in addition to eye movement is desirable. We used two types of movie contents to analyze influence on physiological parameters such as eye movement and Critical Fusion Frequency. As a result, it was confirmed that watching movies affected the latency of the saccade and the variance degree of fixation eye movement. Moreover, constant changes by each movie content were confirmed in several subjects. This study examined the influence that a different movie gave to an audience by the change of eye movement and that of physiological parameter. Moreover, the possibility of evaluating physiological information such as arousal level with eye movements was seen from the change tendency.

**Grant:** none

## 

**Keywords:** Eye movement, Image, CFF, Saccades

## Examination of Gaze Characteristics while Displaying Vibrational Motion Stimuli in the Peripheral Field of Vision

**Takahiro Ueno and Minoru Nakayama**

Information and Communications Engineering, Tokyo Institute of Technology

## 

Perceptual reactions to vibration stimuli orientated in 12 directions were analyzed in order to examine human gaze characteristics while vibrational motion stimuli were displayed in the peripheral field of vision. In the experiment, viewers were asked to detect the vibrational motion of a white circle shaped stimulus in their peripheral field of vision while they conducted a RSVP task using letters positioned in the central field of vision to examine the characteristics during the gaze. Viewer’s eye movements were also measured at a sampling rate of 400 Hz in order to examine the change in attention level during task execution. Fields of view were classified into three regions using cluster analysis of the percentage of correct detection of the direction of vibrating stimuli. Perception ability was higher for movement in a horizontal direction and for the area immediately below this. Results of cross spectral density analysis of eye movement during the presentation of vibration stimulus also suggested that the cross spectral power increase in the frequency band below 4 Hz shows the relationship between the dispersion of attention and activation of eye movement.

**Grant:** none

## 

**Keywords:** eye movement, peripheral vision field

## An Invisible Target Caused by Backward Masking Induces a Saccadic Eye Movement

**Takuto Araki^1^, Yasuaki Tamada^1^, Masayuki Sato^1^ and Keiji Uchikawa^2^**

^1^Graduate School of Environmental Engineering, The University of Kitakyushu

^2^Human Media Research Center, Kanagawa Institute of Technology

## 

It is known that eye movements and visual perception are processed in separate neural pathways. One might expect that a target, made invisible due to backward masking, could still cause a saccadic eye movement. To verify this expectation, we measured a saccade latency between a stimulus presentation and the saccade onset. We presented a circular target at 10 deg either in the right or in the left visual field from a fixation point, then an annular mask at a certain stimulus onset asynchrony (SOA). Subjects made a saccade to the target (or to the mask) as soon as possible. It was obtained that the saccade latency was 235 ms when subjects could not detect the target due to the mask with SOA of 60 ms, and that it was 202 ms for the mask only. The saccade latency should be 262 ( = 202 + 60) ms if the mask only would cause the saccade. This indicates that the invisible target contributed to the saccade. Furthermore, when subjects could detect the target the saccade latency was 215 ms, shorter than when the target only was presented (234 ms), suggesting that the mask enhanced the eye movement signal of the visible target.

**Grant:** none

## 

**Keywords:** eye movement, psychophysics

## Influence of Frequency of Eye Movements on Decision Making

**Dan Uemura and Kenji Yokoi**

National Defense Academy of Japan

## 

There had been a controversial issue about the relationship between eye movements and decision making in the gaze cascade effect. In our previous study, we demonstrated the involvement of eye movements in preferential decision by using the gaze-contingent method, in which stimuli were shifted according to the gaze while keeping the retinal image constant irrespective of saccades (Uemura et al., 2019). The results also suggested that the frequent eye movements to the chosen face might reinforce the preference bias. So, in this study, we investigated the influence of frequency of eye movements on preference formation. Similar to the previous studies, two faces were presented alternatively with different durations (900 ms or 300 ms) and the participants selected the more attractive one. To manipulate the frequency of eye movements, the longer-presented face was moved in a circular trajectory up to six times within 900 ms and the participants were asked to chase it. The relationship between the frequency of eye movements and preferential bias was discussed in relation to the proposed gaze cascade model.

**Grant:** none

## 

**Keywords:** gaze cascade effect, eye movements, decision making, face recognition

## Change in Posture and Body Sway by Looking Down

**Masahiro Ishii**

Sapporo City University

## 

An eccentric visual target, which requires a large lateral gaze shift to project it onto the fovea, elicits not only an eye and head movement but also a trunk and foot movement in the same direction (Hollands, et al., 2004; Land, 2004; Solomon, et al., 2006). Although the temporal and spatial characteristics of the body movements while gazing at eccentric targets have been studied, it seems that none has studied the body movements while looking down. In this study, the posture and the center of foot pressure of subjects were measured while they were looking down. The posture was measured using a video camera, and the center of foot pressure was measured using a sway meter device. The downward angle from the horizontal to a line of sight from the observer to the visual target was 70 degrees. We found that the subjects moved their shoulder forward and their buttocks backward while looking down. We also found that the center of foot pressure shifted posterior with deteriorated postural stability.

**Grant:** none

## 

**Keywords:** eye-body coordination

## Automatic Detection of Oculomotor Disorders

**Jean-Louis de Bougrenet^1^, Mathieu Lamard^2^, Johanne Bolloch^1^ and Vincent Nourrit^1^**

^1^IMT Atlantique

^2^University of Western Brittany (UBO)

## 

Assessment of oculumotor functions is a key part of any eye examination and several studies have shown that it could also help in the diagnostics of various neurological conditions. Objective measurement of oculomotor performances using eye tracking is however little used in orthoptics or clinical practice, perhaps partly because current eye tracking tools provide raw or little processed data that are of limited value without deeper analysis. The aim of this study was to develop more sensitive tests and analysis tools to be able to identify automatically patients with oculomotor disorders. 40 subjects (25 normals and 15 orthoptics patients) participated to the study at Brest University Hospital. Subjects had to perform series of 3 different orthoptics exercises (fixation, saccades, small pursuit) during which their eyes’ movements were recorded with a Tobii Pro Spectrum eye tracker. The spatial and temporal statistics of the trajectories were analysed using conventional metrics and a recurrent neural network. The study shows that an eye tracker is a valuable tool to quantify oculomotor performances in orthoptics practice. The results of this study open up interesting possibilities for the use of the various algorithms in future work.

**Grant:** none

## 

**Keywords:** eye tracking, Orthoptics

## Do ‘No’ Responses Arise from the Same Processing as ‘Yes’?: A Two-Stage Model for Object Detection Using Fragmented Contours

**Kosuke Taniguchi^1^, Kana Kuraguchi^2^ and Yukuo Konishi^1^**

^1^Center for Baby Science, Doshisha University

^2^Faculty of Psychology, Otemon Gakuin University

## 

In an object detection task, participants respond ‘yes’ when an object is detected, or ‘no’ when no object is detected. Previous studies have not argued significant differences in the processing of yes/no responses in the 2-alternative forced choice task. In this study, we investigated whether yes/no responses differ in response time and accuracy by controlling the task difficulty through fragmented contours (short and long length) and stimulus duration (50, 100, and 200 ms). The results showed a significant difference in the processing of yes/no responses, in that ‘yes’ responses were accurate regardless of task difficulties, whereas ‘no’ responses differed by task difficulty. Here, accurate and slow ‘no’ responses correlated with easy task difficulty, and inaccurate and fast ‘no’ responses correlated with difficult tasks. Therefore, we suggest a two-stage model involving early and later processing. In the early processing stage, inaccurate and quick ‘no’ responses are generated when information received is insufficient. In the late processing stage, accurate ‘yes’ and delayed ‘no’ responses are generated as a result of object processing, such as grouping. Thus, ‘yes’ responses arise as a result of accurate decision making, whereas ‘no’ responses, depending on task difficulty, arise from both intuitive and accurate decision making.

**Grant:** none

## 

**Keywords:** yes/no response, contour detection, 2-alternative forced choice, decision making

## Influence of Optical Flow on Unconscious Horizontal- and Cyclo-Vergence

**Kenji Murase^1^, Yusaku Takeda^1^, Toshihiro Hara^1^ and Hirohiko Kaneko^2^**

^1^Mazda Motor Corporation

^2^Tokyo Institute of Technology

## 

In order to present appropriate visual information while driving an automobile, it is necessary to know the behavior of unconscious eye movement in addition to the intentionally controlled gaze direction. In this study, we measured and analyzed horizontal- and cyclo-vergences, while observing optical flow simulating the visual stimulus when driving an automobile. In the experiment, randomly scattered white dots on a black background were presented on the display and they moved to simulate the optical flow when moving forward in a three-dimensional virtual space. The velocity and spatial configuration were stimulus variables. As a result, we found that unconscious out-cyclovergence (right and left eyes move clockwise and counter clockwise, respectively) occurred when observing the optical flow while moving forward. This result indicates that the shape of horopter, which is the spatial locus producing the images on the corresponding points of two eyes, is different during driving and during stopping and then the human space perception would also change. Based on this results, it will be possible to define the appropriate positions for presenting visual information in the cockpit of an automobile by measuring or predicting unconscious eye movements due to the optical flow while driving.

**Grant:** none

## 

**Keywords:** eye movement, vergence, optical flow

## Infant Brain Detects Inconsistency Between Belief and Action: An Electroencephalography Study

**Yu-Ju Chou^1^ and Jung-Tai King^2^**

^1^Department of Early Childhood Education, National Tsing Hua University

^2^Brain Research Center, National Chiao Tung University

## 

The ability to predict the actions of others on the basis of their perspectives is crucial to successful social interactions. A current debate concerns whether preverbal infants possess this ability; the ability can be investigated by examining their dynamic brain activity. In this study, we manipulated the inconsistency between others’ beliefs and subsequent actions to examine the conflict detection ability of preverbal infants with respect to the beliefs and actions of others. We examined the mid-frontal theta-band power activation of 18 six-month-old infants when they were presented with two false-belief scenarios that led to different action consequences. An additional eleven infants were excluded from the analysis because they failed to complete the task or had high EEG noise. The results showed that ten infants’ frontal theta-band power significantly increased after the onset of an agent’s action in the incongruent condition compared with the congruent one. These findings indicate that six-month-old infants already possess the ability to represent an agent’s intended action and detect a different outcome. Other factors, such as parent–child interaction quality and infant temperament, may also influence the time at which this ability develops and must be studied further.

**Grant:** none

## 

**Keywords:** infant, belief-based action prediction, conflict detection, electroencephalography

## A Normative Formalism for Internal Criterion Inference and Its Validation in Human Visual Classification and Brain Activity

**Heeseung Lee^1^, Kyoungwhan Choe^2,3^ and Sang-Hun Lee^1^**

^1^Department of Brain and Cognitive Sciences, Seoul National University

^2^Department of Psychology, The University of Chicago, Chicago

^3^Mansueto Institute for Urban Innovation, The University of Chicago

## 

Everyday life requires humans to make discrete decisions on continuous perceptual quantities. To this end, we learn and apply ‘criterion’ that partitions the continuous perceptual quantities into discrete classes. Reflecting this intuition, criterion has been an essential theory constituent on perceptual decision-making. However, criterion has mostly been assumed simply as a constant lacking a computational rigor commensurate to other theory constituents such as ‘stimulus’ and ‘decision variable’. Here, we redressed this inconsistency by developing a unified computational model that has the optimal inference process also for criterion not only for the other theory constituents, and the normative interplay between them. The criterion inference manifested its empirical impacts in a decision, decision time, and neural activity concurrently by revealing the relativity in decision-making and discerning neural loci for each theory constituents. Therefore, we hope to supersede the conventional constant-criterion framework in systems neuroscience by the comprehensive framework with a criterion inference.

**Grant:** The Brain Research Programs and Basic Research Laboratory Program through the National Research Foundation of Korea (NRF- 2015M3C7A1031969; NRF-2017M3C7A1047860; NRF-2018R1A4A1025891)

## 

**Keywords:** decision-making, internal criterion, Bayesian inference, fMRI

## Learning Decision Criteria from Feedback for Visual Classification

**Hyang-Jung Lee and Sang-Hun Lee**

Department of Brain and Cognitive Sciences, Seoul National University

## 

Every day we perform numerous decision-making tasks, with a specific goal for each. To maximize performance, one must learn the true structure of contingencies imposed by the environment, typically based on feedback. The contributions of feedback to such learning in perceptual decision-making remain elusive, compared to those in reward-based or motor decision-making. The present work focuses on how people utilize feedback to learn decision criteria for a visual classification task. While people classifying ring stimuli of different sizes into ‘small’ and ‘large’ over consecutive trials, we stochastically sampled criteria used for giving ‘correct’ or ‘incorrect’ feedback. Taking a Bayesian approach, we (i) developed a generative model—people’s causal account for the statistical structure of our task, (ii) created several Bayesian agents who differ in the way of propagating the information provided by feedback to infer decision criteria, and (iii) examined which agent’s inference best matched those of people by comparing psychometric and chronometric curves. We found one strong winning agent, who ‘rationally’ resolves the logical inconsistency between her own decision (e.g., ‘small’) and feedback (e.g., ‘large’) by allowing neither her own decision nor feedback to propagate over her generative model when inferring decision criteria.

**Grant:** The Brain Research Programs and Basic Research Laboratory Program through the National Research Foundation of Korea (NRF- 2015M3C7A1031969; NRF-2017M3C7A1047860; NRF-2018R1A4A1025891)

## 

**Keywords:** psychophysics, computational modeling

## Chromatic Information Modifies Gaze Patterns in Visual Search

**Natsuko Wasaki^1^, Tatsuto Takeuchi^1^ and Sanae Yoshimoto^2^**

^1^Japan Women’s University

^2^Hiroshima University

## 

Experts in comprehension of visualization, who engage in deliberate practice, such as radiologists, perform very well in tasks involving finding targets from images. Superior visual search skills generally accompany the modification of gaze patterns while searching. Meanwhile, chromatic information is known to enhance the comprehension of various types of images, such as metro maps, and therefore, finding a target is easier on a colored map than on a gray-scale map. The question is whether gaze patterns on a colored map is similarly modified as that of experts. In this study, participants searched a target station on the colored or gray-scale metro map seen for the first time. We found that the gaze patterns for the colored map were modified as follows: reduced time to first fixation, shorter fixation duration and dwell time, and fewer fixation counts. However, these patterns were dependent on the areas of interest and the saccade amplitude did not differ between the colored and gray-scale maps. No significant effect of practice was found for the colored map. These results indicate that the gaze patterns could be modified by adding color, but the underlying mechanisms enabling performance enhancement of visual search by color and practice would be different.

**Grant:** none

## 

**Keywords:** visual search, eye movement, color, learning

## Visual Hallucinations in Parkinson’s Disease – Understanding Fixation Potentials in a Free-Viewing Face Detection Experiment

**Gajanan S. Revankar^1^, Noriaki Hattori^1^, Tomohito Nakano^1^, Yuta Kajiyama^1^, Etsuro Mori^2^ and Hideki Mochizuki^1^**

^1^Department of Neurology, Graduate School of Medicine, Osaka University

^2^Department of Psychiatry, Graduate School of Medicine, Osaka University

## 

In Parkinson’s disease (PD) patients a precursor phenomenon to Visual hallucinations (VH) known as Pareidolia occurs. These are misperceptions of meaningful objects/faces arising from ambiguous forms. The main purpose of this study is to understand the mechanism of VH in PD, using Pareidolias as a marker to elucidate the temporal dynamics of brain in face recognition and misperceptions. We performed co-registered eye-tracking – electroencephalography (EEG) recordings in 20 PD patients and 12 age matched healthy controls using a computer based modified Noise pareidolia test which involves identifying faces from noisy images. To assess cognitive status, a series of neuropsychological assessments were done. Half of the PD patients (n = 10) showed pareidolic responses (misperceptions) whereas the other half did not. Those with pareidolias had much higher pareidolias when compared to either group. Fixation related parameters showed poorer accuracy and increased latency in PD patients. Presaccadic potential was seen to be less negative in PD compared to controls explaining the failure of visual encoding and transfer of attention during visual exploration. Fixation potentials changes between the groups did not correlate well with neuropsychological examination. We describe the potential use of pareidolias as early markers for identifying PD patients prone to hallucinations.

**Grant:** none

## 

**Keywords:** Eye-tracking, Electroencephalography, Unconstrained visual exploration, Eye-fixation related potentials

## Proposal of New Evaluation Method of Mental Work-Load Using Eye-Head Coordination

**Kimihiro Yamanaka^1^ and Kenji Kobayashi^2^**

^1^Intelligence and Informatics, Konan University

^2^Graduate School of Natural Science, Konan University

## 

Experiments on mental work-load and eye-head coordination were carried out to propose the evaluation index of mental work-load on the task, without distracting subject’s main task. Eye and head movement was measured by non-contact device under the condition which subject’s mental work-load was controlled. In particular, Subjects performed tasks which are three kinds of numerical task such as hard, middle and easy for inducing an increase the mental work-load, and driving task for inducing eye-head coordination. In the experiment, subjective work-load and the ratio of correct answers for numerical task were measured. As a result of consideration on numerical task, it was found that subject’s mental work-load was increased under numerical task of hard conditions. Also, the ratio of correct answers was decreased. As a result of eye and head movement, it was found that eye-head coordination to visual target was influenced by mental work-load. Eye movement precedes on viewing to visual target which under numerical task of easy conditions. On the other hand, head movement precedes on viewing to visual target under numerical task of hard conditions.

**Grant:** none

## 

**Keywords:** eye and head movement, visual information processing, mental work-load

## Suitable Dwell Time for Eye-Gaze-Based Object Selection with Eye Tracking

**Yesaya Tommy Paulus^1^ and Gerard B Remijn^2^**

^1^Kyushu University, Graduate School of Design, Department of Human Science

^2^Kyushu University, Faculty of Design, Department of Human Science

## 

Dwell time refers to the time needed to select specific objects on a screen with eye-gaze-based input, for example with eye tracking. This study investigates the dwell time necessary to select objects on a display for three types of visual password formats. The formats were an alphanumeric format, a pattern format, and a picture format. The participants were asked to memorize a 4-object or a 6-object password and register, confirm, and log in the password using eye-gaze-based input, with a minimum dwell time set at 250 ms, 500 ms, 1000 ms, and 2000 ms per object. The task was performed on 4 grids, consisting of 3 × 4, 4 × 3, 4 × 5, and 5 × 4 cells (columns-by-rows), respectively. The participants were also asked to evaluate each dwell time with a rating scale. The results showed that the participants required more time to authenticate a 4-object or 6-object password as the dwell time increased, but with fewer input mistakes for each of the three password formats. These results suggest that a dwell time in between 500 and 1000 ms is suitable for visual password object selection using eye-gaze-based input, depending on individual performance and preferences.

**Grant:** none

## 

**Keywords:** dwell time, eye-gaze-based input, eye tracking, visual password

## A Social Interaction-Based Model for Human Locomotion

**Mingcheng Miao^1^, Chen Zhou^1^, Yifei Hu^1^, Xinran Chen^1^, Shuguang Kuai^1,2^**

^1^Shanghai Key Laboratory of Brain Functional Genomics, Key Laboratory of Brain Functional Genomics, Ministry of Education, School of Psychology and Cognitive Science, East China Normal University

^2^NYU-ECNU Institute of Brain and Cognitive Science, New York University Shanghai

## 

As social animals, people tend to keep a comfortable distance with other ones, named as personal space. When people bypass others, they are not only avoiding a physical collision, but also avoiding intruding personal spaces of others. Here, we conducted three experiments to testify the hypothesis and established a social interaction model to predict human walking behaviors in a social environment. In Experiment 1, participants were asked to steer towards a pillar while a virtual human was standing halfway between the pillar and participants in a virtual environment. We found that participants tend to bypass the virtual human from the side with weaker potential social interaction. In Experiment 2, participants were asked to bypass a virtual human who was oriented to different directions. We then quantitatively measured the space participants avoided during their walking. Based on that, we built a social interaction model to predict walking routes. In Experiment 3, we asked participants to bypass virtual human at random positions and with different orientations. The social interaction model achieved a good performance in predicting the routes, supporting the robustness and generalization of the model. Our results indicate that human walking behavior is influenced by the perception of others’ personal space.

**Grant:** National Natural Science Foundation of China Grants (No. 31771209)

## 

**Keywords:** Perception and action: Locomotion, Behavior/Psychophysics

## The Presence of Pupillary Responses Confounded by Eyeblink, and a Statistical Solution for That Confound

**Sang-Hun Lee and Kyung Yoo**

Department of Brain and Cognitive Sciences, Seoul National University

## 

Pupillometry has become one of the most popular measurement tools among cognitive and systems neuroscientists, earning an honorable sobriquet “window for cognition.” Pupillometry indeed has many merits as indirect measures of internal cognitive states. Despite its popularity and merits, however, users must be warned of the fact that potential factors that influence pupil size have not been fully catalogued, and, more importantly, that it is currently unknown how those factors interact with one another. The present work notes ‘eyeblink’ as one of so far unattended yet substantive factors and demonstrates theoretical and empirical repercussions of failing to handle ‘eyeblink’ properly. Our findings are three folds. First, we show that each blink triggers a specific temporal profile of pupil size change, 1-second long period of constriction followed by slower recovery, which we named BPR (blink-locked pupillary response). Second, we demonstrate that BPR confounds pupillary changes caused by cognitive factors by showing that the prevalence of eyeblink is associated with cognitive factors such as working memory loads. Lastly, we develop a statistical toolbox that corrects pupillary responses for BPR. We expect the work to help users of pupillometry recognize the presence of BPR and fix the confounds due to BPR effectively.

**Grant:** The Brain Research Programs and Basic Research Laboratory Program through the National Research Foundation of Korea (NRF- 2015M3C7A1031969; NRF-2017M3C7A1047860; NRF-2018R1A4A1025891)

## 

**Keywords:** pupillometry, eyeblink, deconfounding

## Patterned Silencing of Parvalbumin Neurons in Rodent Visual Cortex

**Akira Masuda and Susumu Takahashi**

Doshisha University

## 

Stimulation of visual cortex can induce artificial sense of vision, called phosphenes, and be utilized for recovery of visual loss. Optogenetic stimulation promises higher resolution and more specific neural targets than other forms of stimulation including mechanical, electrical, or magnetic ones. However, few studies tested which combination of cell-types and optogenetic proteins is applicable for the visual restoration. Here, in rats, we electrophysiologically characterized optogenetic silencing of parvalbumin (PV) positive inhibitory neurons in monocular region of primary visual cortex (V1 m) to locally disinhibit neural activity. We used PV-Cre rats which had given adeno-associated virus (AAV) fused with DIO-NpHR3.0 into V1 m. Red LED light (630 nm) was emitted from photo-stimulator system consisting of mini-LED (8 × 8 pixels in 20 × 20 mm, 2.8 mm pitch) and two convex lenses (biconvex and pleno-convex lens) installed above the V1 m. We recorded physiological responses evoked by illumination (optogenetic stimulation) or visual stimuli with high-density neural probes on the anesthetized conditions. We found that large number of excitatory neurons showed phasic increase in response to illumination. This suggests that the optogenetic silencing PV neurons is an applicable strategy of artificial vision.

**Grant:** none

## 

**Keywords:** visual cortex, optogenetics, artificial vision, electrophysiology

## Using Macromolecular Tissue Volume Mapping to Identify Subdivisions in Human Lateral Geniculate Nucleus

**Hiroki Oishi^1,2^, Hiromasa Takemura^1,2^, Kaoru Amano^1,2^**

^1^Center for Information and Neural Networks (CiNet), National Institute of Information and Communications Technology and Osaka University

^2^Graduate School of Frontier Biosciences, Osaka University

## 

Lateral geniculate nucleus (LGN) consists of magnocellular (M) and parvocellular (P) subdivisions. While these subdivisions have different anatomical properties, identifying these subdivisions from living human brain using structural MRI has been difficult, partly due to their small size and image inhomogeneity in a standard T1-weighted image. In this study, we tried to identify human LGN subdivisions using macromolecular tissue volume (MTV) mapping (Mezer et al., 2013), which provides quantitative map corrected for image inhomogeneities. We first identified the entire LGN from four healthy participants using high-resolution proton density-weighted image. We then collected MTV data and classified 20% of voxels with the lowest MTV to the M-group and the remaining 80% of voxels to P-group. This classification is because the area size of P-group is roughly four times larger than that of M-group (Andrews et al., 1997). As a result, we found that estimated M- and P-group voxels are clearly separated and are located ventromedial and dorsolateral respectively in all participants. These patterns were consistent with LGN anatomy for human and primates. This result suggests that MTV mapping provides stable parcellation for LGN subdivisions, which will be useful to study human LGN anatomy with respect to retinal disorders or visual functions.

**Grant:** Japan Society for the Promotion of Science (JSPS) KAKENHI (JP17H04684, H.T)

## 

**Keywords:** Lateral Geniculate Nucleus, Quantitative MRI, Tissue segmentation

## Inter-Individual Deep Image Reconstruction

**Tomoyasu Horikawa^1^, Yukiyasu Kamitani^1,2^**

^1^Department of Neuroinformatics, ATR

^2^Graduate School of Informatics, Kyoto University

## 

Recent studies demonstrated the utility of deep neural networks (DNNs) for characterizing visual representations in the brain, offering opportunities to develop advanced applications via brain–DNN interfacing. By decoding brain activity measured by functional MRI into DNN feature patterns, it has become possible to reconstruct visual images via decoded feature patterns (“deep image reconstruction”). However, modeling the relationship between DNNs and the brain requires large amounts of data (several hours of measurements) from each individual, limiting the generalizability of such brain–DNN technology. Here, we examined whether models for deep image reconstruction can generalize across individuals by functionally aligning brain data. We used hyperalignment techniques to construct a common representational space from brain activity patterns of multiple individuals, using identical sequences of natural scene stimuli. Reconstruction was performed using one individual’s data for model training and another’s for testing. The inter-individual analysis successfully reconstructed viewed images with recognizable silhouettes of objects even with ∼30 min brain data for functional alignment. Our results demonstrate that functional alignment with a small amount of data preserves detailed visual feature representations across individuals, providing an efficient way to create visual image reconstructions without training data from each individual.

**Grant:** none

## 

**Keywords:** brain decoding, visual image reconstruction, deep neural networks, functional magnetic resonance imaging

## The Response Property and Efficacy of Prosthetic Retinal Stimulation by Single-Unit Analysis

**Tomomitsu Miyoshi^1^, Hiroyuki Kanda^2^, Takeshi Morimoto^2^ and Takashi Fujikado^2^**

^1^Dept. Integrative Physiology, Graduate School of Medicine, Osaka University

^2^Dept. Applied Visual Science, Graduate School of Medicine, Osaka University

## 

We have been developed a novel retinal prosthesis, Suprachoroidal Transretinal Stimulation (STS) for photoreceptor degenerating diseases. The stimulating electrode array of STS is implanted into sclera and do not directly contact retinal tissue to avoid physical damage. To investigate the response properties by STS, we recorded the single-unit activities from cat lateral geniculate nucleus (LGN) relay neurons. The size of each electrode head in the implanted array was 0.5 mm in diameter and 0.3 mm in height, which was the same as the clinical device. The waveform of single pulse was biphasic, 0.5 or 1 mA amplitude, and 0.5 ms/phase duration. The single pulse STS elicited the burstic discharges, which occurred alternately on ON and OFF cells. This burstic response was due to the prolonged change of excitability by the interaction in the stimulated retinal circuits. With the continuous stimulation, the elicited number of spikes was not proportional to stimulating frequency. The maximum discharges per second was achieved by the stimulation not greater than 50 Hz, suggesting that high frequency stimulation may less effective.

**Grant:** none

## 

**Keywords:** retinal prosthesis, electrophysiology

## The Double Representation of the Fovea, If There Are Ipsilateral Connection from the Eye to the LGN, Why Is There No Cortical Representation?

**Mark Matthias Schira**

University of Wollongong

## 

The debate about a double representation of the fovea in human visual cortex is still ongoing (Jordan et al., 2014). Ipsilateral connections from the retina to the LGN exist and are reasonably well described (Stone, 1973; Bunt & Minkler, 1977; Fukuda, et al., 1989). The retinotopic representation of the foveal confluence in human visual cortex, specifically the central 0.5 degree, is substantial with more that 2000 mm^2^ for V1, V2 and V3 alone (Schira et al., 2009) exclusively representing the contralateral visual field. No ipsilateral visual field representation can be seen in V1. Reanalysing the data by Bunt & Minckler and Fukuda et al., only a very small count between 110 and 130 cells was estimated. For conscious perception it needs be considered: Firstly, while the ipsilateral overlap is relatively large in the periphery (up to 15 degree), close to the fovea it is either less 0.5 degree or absent. Secondly, there are a substantial number of transcallosal fibers along the representation of the vertical meridian at the boundary of V1 and V2, especially in the foveal confluence (Zeki et al., 1969, Van Essen et al., 1986) suggesting a double representation would be superfluous. Finally, macular sparing, clearly cannot be well explained by an ipsilateral representation of the visual field. Many patients with hemianopia have no macular sparing whatsoever (Reinhard &Trauzettel-Klosinski, 2003), which is irreconcilable with a significant ipsilateral representation.

**Grant:** none

## 

**Keywords:** Foveal vision, Anatomy, Retinotopy, Physiology

## Quantifying the Impacts of Transcranial Electrical Stimulation on Cortical Activity in Human Visual Cortex

**Jeongyeol Ahn^1^, Juhyoung Ryu^1^, Sangjun Lee^2^, Chany Lee^3^, Chang-Hwan Im^2^ and Sang-Hun Lee^1^**

^1^Department of Brain and Cognitive Sciences, Seoul National University

^2^Department of Biomedical Engineering, Hanyang University

^3^Department of Structure & Function of Neural Network, Korea Brain Research Institute

## 

Transcranial electrical stimulation (tES) has become a popular interventional method of stimulating human brains noninvasively. Despite reports of modulation of membrane potentials or BOLD responses by tES, it is far from conclusive whether and how tES affects neural activity. One prominent factor contributing to this inconclusion is that the baseline variability of noises intrinsic to measurements, which occur with diverse origins not just between but also within experimental sessions, have not been properly handled in previous studies. For example, the intrinsic variability of hemodynamic responses within and between scans causally confounds tES and thus complicates the attribution of observed effects in BOLD. To overcome this problem, we developed an experimental protocol that allows for statistically dissecting tES effects and other intrinsic noises in BOLD activity. By applying this protocol to human visual cortex, we demonstrate that tES induces substantial changes not only in the temporal dynamics of hemodynamic response function (HRF) but also in cortical population responses to dynamic stimuli, which cannot be reduced to the changes in HRF. Our findings imply that tES, when applied in protocols with statistical rigor and power, can manifest its impacts on BOLD signals in much more complicated and nuanced ways than previously reported.

**Grant:** The Brain Research Programs and Basic Research Laboratory Program through the National Research Foundation of Korea (NRF- 2015M3C7A1031969; NRF-2017M3C7A1047860; NRF-2018R1A4A1025891)

## 

**Keywords:** Transcranial Electrical Stimulation, Visual Cortex, fMRI

## Quantitative Analyses of Cortical Responses to Prosthetic Microstimulations Using Voltage-Sensitive Dye Imaging on Mice

**Celine Audrey Vergne, Lucas de Levy Oliviera, Tamas David Fehervari, Keisuke Morisada, Naofumi Suematsu, Yuki Hayashida and Tetsuya Yagi**

Graduate School of Engineering, Osaka University

## 

Microstimulation with penetrating electrodes is now considered to be one of the key technologies in visual cortical prosthetics for restoring visual function in blind patients. However, it is still not well understood how the excitation distributes and propagates in the cortical circuits in response to the microstimulations, although it is crucial to design the cortical prostheses. The purpose of the study is to elucidate a quantitative relationship between the microstimulation and the spatiotemporal properties of cortical response. We applied a single pulse and repetitive pulse stimulations to the mice primary visual cortex and imaged the responses using voltage-sensitive dye imaging. The response induced by a single pulse spread in wide region of the primary visual cortex. In contrast, the spreading region of response to repetitive stimulation gradually shrank following the transient propagation to wide region. We modeled these spatiotemporal properties of cortical responses with a combination of excitatory and inhibitory synaptic responses. The present results quantified the spatiotemporal properties of cortical responses to microstimulations and thus provides critical insight to evaluate the efficacy of the stimulation patterns for evoking phosphene perception.

**Grant:** Grant-in-Aid for Scientific Research from MEXT, Japan (25282130 and 18K12059 and 16717084), and Grant-in-Aid for Challenging Exploratory Research from MEXT, Japan (25560197)

## 

**Keywords:** Visual prostheses, Microstimulation, Imaging, Model analysis

## Wearable Phosphene Image Simulator for Cortical Visual Prosthesis

**Fumiaki Nakao, Nobuaki Kishigami, Jinhwhan Choi, Yuki Takano, Naofumi Suematsu and Tetsuya Yagi**

Graduate School of Engineering, Osaka University

## 

Previous clinical and animal experiments suggeted that microstimulations delivered to visual cortex with multi-electrode induced apparent image consisting of phosphenes, which could partially restore the visual function in blind patients. In order to evoke appropriate perception that is useful for object recognition, self-navigation and so on, the electrode arrangement in the cortical area and current stimulation patterns must be designed so that phosphene image should effectively convey the image information of surrounding world. However, it is not straight forward to predict the feasible phophene images, since the visuo-topic map from the visual field to the cortical surface is not simple. In the present study, we built a wealable phosphen image simulator consisting of an image processing unit, a visual cortex model expressed with the wedge-dipole formulation and a HMD. The phosphene images were reconstructed in real time on the HMD while modifing the electrode arrangemet and current stimulation patterns in the cortex model. We conducted psychophysical experiments on normally sighted subjects wearing the phosphen simulator to assess the effective and feasible electrode arrangement and stimulation patter. The phosphen simulator deviced in the present study accelerates the development of the cortical prosthesis.

**Grant:** Grant in Aid for Scientific Rsearch (C), MEXT (16k01354)

## 

**Keywords:** VIsual prosthesis, Phosphene image, Simulation, Psychophysics

## In vitro Comparison of Orbital Preadipocyte AdipogenesIs Between Pediatric and Adult Graves’ Ophthalmopathy

**Jung Hyo Ahn**

Pusan National Universtiy Medical College

## 

### Purpose

To evaluate whether orbital preadipocytes obtained from pediatric Graves ophthalmopathy (GO) patients differ from that from adult GO under lipopolysaccharide (LPS)-induced inflammatory condition. Materials and methods: The pediatric and adult orbital preadipocytes were differentiated in adipogenesis media without LPS intervention. The remaining pediatric and adult orbital preadipocytes were stimulated to differentiate into mature adipocytes with LPS intervention. The expression of adipogenic transcription factor, peroxisome proliferator-activated receptor-gamma (PPAR-γ), and CCAAT-enhancer-binding protein α (C/EBP-α) were determined by real-time PCR. The cells were stained with oil red O to observe the intracellular lipid accumulation. Results: Without LPS-induced inflammation, the pediatric orbital preadipocytes showed increased expression of PPAR-γ, C/EBP-α, and accumulation of intracellular lipids than the adult orbital preadipocytes. When treated with LPS, both pediatric and adult preadipocytes showed increased expression of adipogenesis as compared to without treatment. Especially, the pediatric orbital preadipocytes had increased expression of PPAR-γ, C/EBP-α, and accumulation of intracellular lipids than adult orbital preadipocytes. Conclusions: Adipogenesis of the pediatric orbital preadipocytes was more affected and up-regulated as compared to that of the adult preadipocytes in GO by LPS-induced inflammation.

**Grant:** none

## 

**Keywords:** orbit

## Female Bias in Face Memory

**Jenna Jaewon Lee^1,2^, Koyo Nakamura^2,3,4^, Katsumi Watanabe^2,5^**

^1^Johns Hopkins University

^2^Waseda University

^3^Japan Society for the Promotion of Science

^4^Keio Advanced Research Centers

^5^UNSW Sydney

## 

People tend to expect to see male than female faces under perceptual uncertainty (Watson et al., 2016). On the other hand, people are better at recognizing female faces when attention is less available during encoding (Palmer et al., 2013). Many previous studies of gender bias in face memory focused on the encoding stage of face memory. In the present study, we examined whether any gender bias would exist in storage and/or retrieval stages in memorization of faces. In the first experiment, participants memorized faces and chose the corresponding face from an array of face stimuli morphed along the sexual dimorphism spectrum. We found that both male and female participants tended to choose the slightly feminized stimuli on the spectrum than what they actually had seen. In the second experiment, we prepared pairs of original and slightly feminized face stimuli. Participants memorized an original face and then the two faces were presented side by side: one original and the other feminized face. We observed similar effects on face memory, namely, the bias to choose feminized faces as remembered ones. Overall, these results suggest that there is a significant bias toward feminine faces in the storage and/or retrieval stages in face memory.

**Grant:** none

## 

**Keywords:** Cognitive Science, Memory, Face

## Cultural Differences in the Generalization of the Mere Exposure Effect

**Hidekazu Yarimizu^1,2^, Koyo Nakamura^3,4,5^, Katsumi Watanabe^3,6^ and Masami Kanazawa Yamaguchi^1^**

^1^Faculty of Letters, Chuo University

^2^Faculty of Psychology, Meijigakuin University

^3^Faculty of Science and Engineering, Waseda University

^4^Japan Society for the Promotion of Science

^5^Keio Advanced Research Centers

^6^Art & Design, University of New South Wales

## 

This study investigated the cultural differences in the generalization of the mere exposure effect, which was examined using Asian face stimuli for Asians. The generalization of the mere exposure effect refers to the increase in liking of a previously exposed face evaluated again later, regardless of face directions presented earlier. Exposure to upright and inverted faces was compared to confirm that the face is specific in the generalization of the mere exposure effect. The vertical orientation (upright and inverted) and the horizontal angles (0, 45, 90 degrees) of Asian faces were manipulated, and those faces were presented to Asian participants. The participants then evaluated their liking of the upright face presented at 0°. Regarding upright faces, exposure at 45° increased their liking when the face was evaluated later than at 0°; however, there was no difference between 0 and 90 degrees. Inverted faces did not change the participants’ liking for them depending on the angles. These results show that exposure to inverted faces increases liking of the same faces in upright orientation in Asians, which is different from the result of a previous study with Caucasians.

**Grant:** none

## 

**Keywords:** Face, Cultural Difference, The Mere Exposure Effect

## Activation Pattern Classification Revealed Facial Expressions Encoding in N170 and P200

**Ka Lon Sou^1^, Manuel Seet^2^ and Hong Xu^1^**

^1^Psychology, School of Social Sciences, Nanyang Technological University, Singapore

^2^Singapore Institute of Neurotechnology (SINAPSE), National University of Singapore

## 

How does the brain encode emotional stimulus? It has been debated whether the activation of the face-sensitive N170 is modulated by emotion. Most previous studies focused on the peak activation of the N170, but largely ignored the activation pattern of the N170 or P200 which is often overlooked in emotion perception. In this study, we investigated whether the activation patterns of the N170 and P200 can predict the emotion category of the presented face stimuli. Fifteen subjects were recruited to complete an emotional face categorization task, while EEG signals were recorded simultaneously. We trained the support vector machine to classify emotion of the presented emotional faces based on the N170 activation pattern and the P200 activation pattern. Three main findings emerged: 1) The P200 peak amplitude was less negative for happy faces. 2) Both the N170 activation pattern and the P200 activation pattern could predict the emotion category of the presented face above chance level. 3) Neutral faces were more accurately classified than angry faces with the P200 activation pattern. Our results argue that the N170 and the P200 might be both activated in facial emotion perception but play different roles in emotion categorization.

**Grant:** none

## 

**Keywords:** Emotional face perception, EEG, Pattern classification analysis

## Unattractive Faces are Identified more Easily than Attractive Ones

**Kana Kuraguchi^1^ and Hiroshi Ashida^2^**

^1^Otemon Gakuin University

^2^Kyoto University

## 

Attractive faces capture or gaze and we can judge facial attractiveness immediately even in peripheral vision. These previous studies suggest that attractive face is detected easily but that unattractive face is rather ignored. We hypothesize that perceiving unattractiveness precedes perceiving attractiveness for distracting attention from unattractive faces and then focusing on attractive ones. To test this idea, we investigated whether facial attractiveness affects performance of face identification. In this study, we conducted a face identification task, in which participants judged whether a face matched one of the two faces presented earlier, and then compared the hit rates of attractive and unattractive faces. Result showed that hit rate of unattractive faces was significantly higher than of attractive ones, which suggests that facial attractiveness affects face identification. This effect of facial attractiveness on face identification, however, appeared only when both attractive and unattractive faces were presented and were directed attention to. Identification of unattractive faces with comparative ease may facilitate directing our gaze to attractive faces, which is considered adaptive behavior.

**Grant:** This study was supported by JSPS KAKENHI Grant Number 17K13901.

## 

**Keywords:** face

## Data-Driven Mathematical Modeling Reveals Hidden Cues to Attractiveness: Are Attractive Faces Always Feminine-Looking?

**Koyo Nakamura^1,2,3^, Katsumi Watanabe^1,4^**

^1^Faculty of Science and Engineering, Waseda University

^2^Japan Society for the Promotion of Science

^3^Keio Advanced Research Centers

^4^Art & Design, University of New South Wales

## 

Facial attractiveness is judged through a combination of cues. Sexual dimorphism (facial differences between the sexes that emerge at puberty) is an important factor influencing perceived attractiveness. Previous studies have demonstrated that female-looking faces are often preferred over male-looking ones among both males and females. However, it remains unclear how critically facial femininity affects attractiveness judgments, relative to other facial features. To examine the relationship between facial attractiveness and sexual dimorphism, we applied data-driven mathematical techniques to the ratings of attractiveness and sexual dimorphism for computer-generated male (n = 200) and female (n = 200) faces, provided by Japanese observers. Then, we built a quantitative model for how facial shape and texture may vary on attractiveness, while controlling for perceived femininity. As per the model, we generated faces manipulated on attractiveness, while keeping femininity constant. We found a positive correlation between attractiveness and femininity, but we were still able to identify facial features contributing to attractiveness, even after controlling for perceived femininity. This suggests that femininity affects perceived attractiveness, but attractive faces are not always feminine-looking.

**Grant:** none

## 

**Keywords:** Face perception, Facial attractiveness, Data-driven mathematical modeling

## Serial Dependence in Facial Appearance along the Attractiveness Dimension

**Aki Kondo^1,2^, Koyo Nakamura^2,3,4^, Katsumi Watanabe^3,5^**

^1^Department of Advanced Fibro-Science, Kyoto Institute of Technology

^2^Japan Society for the Promotion of Science

^3^Faculty of Science and Engineering, Waseda University

^4^Keio Advanced Research Centers

^5^Art & Design, UNSW

## 

When we make attractiveness judgment for sequentially presented faces, each judgment assimilates toward the judgment for the preceding trial (Kondo et al., 2012). Recent studies have suggested that the assimilation effect also occurs at the perceptual level; the facial appearance of a person is assimilated toward that of another person presented in the preceding trial (Liberman et al., 2014). In the present study, we examined whether the perception of the facial attractiveness is also assimilated toward the facial attractiveness for the preceding trial. Nine facial images that differed in attractiveness levels were generated from two different identities by using a computational model of facial attractiveness (Nakamura & Watanabe, 2017). On each trial, a randomly selected target face was presented, and participants were asked to match the adjustment face to the target face by changing facial appearance along the attractiveness dimension. The results showed that the facial appearance of the adjusted faces was assimilated toward that of the target attractiveness appeared in the preceding trial. This finding suggests that the serial dependence of face attractiveness occurred at the perceptual level.

**Grant:** This research was supported by Japan Society for the Promotion of Science (JSPS) under Grant-in-Aid for Scientific Research (KAKENHI) grant numbers 15K16008

## 

**Keywords:** Serial dependence, Attractiveness perception, Face perception

## Spatial Frequency Manipulation Reveals Impaired Ensemble Coding of Facial Attractiveness in High Autistic Traits

**Wenxuan Cheng, Haojiang Ying and Hong Xu**

Nanyang Technological University

## 

We are able to perceive the attractiveness of a group of faces through ensemble coding. Manipulating spatial frequency of the faces can highlight the local curvature or global information. How does this manipulation affect ensemble coding? In the present study, 58 participants rated the attractiveness of 4 unattractive and 4 attractive faces individually or in a group with 1) High Spatial Frequency (HSF; >32 cycles per face), 2) Low Spatial Frequency (LSF; <8 cycles per face) and 3) Original Full Bandwidth (FB). We found significant positive correlations between grouped and individual faces ratings, in both unattractive (all rs>.65, ps<.001) and attractive (all rs>.55, ps<.001) conditions, confirming the occurrence of ensemble coding. Past research suggested autistic population demonstrated distinct face processing and preference for HSF. Here we measured participants’ autistic traits by Autism-Spectrum Quotient (AQ). Results showed AQ scores were significantly correlated with the rating difference between the FB and LSF unattractive face ensembles (r = .417, p = .001). It thus suggests the distinct ensemble coding of facial attractiveness in high autistic traits individuals who extract the local high spatial frequency information more than the global low spatial frequency information.

**Grant:** none

## 

**Keywords:** Ensemble Coding, Autistic Traits, Spatial Frequency, Facial Attractiveness

## The Effect of Implicit Racial Bias on Recognition of Own- and Other-Race Faces

**Araz Aslanian and Olivia S. Cheung**

New York University Abu Dhabi

## 

Previous research has established a possible link between recognition performance and implicit racial bias of other-race faces. Here we further examined how recognition of own- and other-race faces might be modulated by observers’ face recognition ability, social experience, and implicit racial bias. Caucasian participants (N = 53) completed a memory task for Caucasian and Asian faces, an implicit association test and a questionnaire on social experience towards Caucasians and Asians, and a face recognition ability test (Cambridge Face Memory Test). The memory performance for own-race faces was positively predicted by increased face recognition ability (β = .54, t(49) = 4.49, p<.0001), whereas the memory performance for other-race faces were positively predicted by increased face recognition ability (β = .45, t(48) = 4.14, p = .0001), social experience with Asians (β = .33, t(48) = 2.77, p = .008), and negatively predicted by increased positive bias towards Asians (β = −.32, t(48) = −2.67, p = .01), which was modulated by an interaction between face recognition ability and implicit bias (β = −.36, t(48) = −3.3, p = .002) with the effect of bias observed only in observers with high face recognition ability. These findings suggest the complexity in understanding the perceptual and socio-cognitive influences on the other-race effect, and that observers with high face recognition ability may involuntarily allocate spare cognitive resources to evaluate racial factors when recognizing other-race faces.

**Grant:** none

## 

**Keywords:** Face recognition, Other-race effect, Implicit racial bias, Psychophysics

## Exploring Visual Processing Strategies of Self-Face and Other-Face Recognition

**Jasmine Lee Kar Wye and Alejandro J. Estudillo**

University of Nottingham Malaysia

## 

Some studies have shown an advantage for the processing of the self-face compared to other familiar and unfamiliar faces. However, it is largely unknown whether the self-face is also processed qualitatively different to other faces. With an eye tracking study, we explore the visual processing strategies for self-face, age- and gender- matched personally familiar face and age- and gender- matched unfamiliar face. Thirty individuals freely explored face images presented on screen without performing any task demands. Images were presented in two different conditions: vertical (i.e., inverted or upright) and horizontal orientation (i.e., mirror-inverted or normal). While in Experiment 1, external features of the faces (i.e., jawline, hairline) were concealed with an oval mask, in Experiment 2, internal features and shape information of the faces were available. In comparison to familiar and unfamiliar faces, self-face received generally more fixations. Interestingly, observers showed a preference for the mouth region when seeing the own face, and for the nose region when seeing unfamiliar and familiar faces. These results might suggest that the self-face is processed in a distinct manner to other faces, wherein self-faces may be processed in a more featural manner than both familiar and unfamiliar faces.

**Grant:** none

## 

**Keywords:** self face recognition, eye-tracking

## The N170 Component Fluctuations Between Upright/Inverted Face Processing Might Predict Behavioral Performance of Holistic Face Processing

**Peter Kuan-Hao Cheng^1,2^, Jun-Hua Lai^1,3^, Chao-Chih Wang^1^, Gary C.-W. Shyi^2,4^, Tzu-Hua Wang^1,5^**

^1^Research Center for Education and Mind Sciences, National Tsing Hua University

^2^Department of Psychology and Center for Research in Cognitive Sciences, National Chung Cheng University

^3^Department of Educational Psychology and Counseling, National Tsing Hua University

^4^Advanced Institute of Manufacturing with High-tech Innovations, National Chung Cheng University

^5^Department of Education and Learning Technology, National Tsing Hua University

## 

In this study, the neurophysiological face-sensitive N170 component of the event-related potential (ERP) were explored through an orientation discrimination task using natural faces, face-like emoji, and scenes. After one month later, the participants were called back to evaluate their face processing ability using composite task. About ERP results, the mean amplitude of the N170 component located in the bilateral occipito-temporal region (P7/P8) was analyzed. The participants elicited an occipito-temporal N170 component for the faces and emoticons, and the N170 is significantly larger for the inverted faces than upright faces while this effect was not appeared in emoticons. And the effect of orientation for faces on P8 (right hemisphere) is more significant than the effect on P7 (left hemisphere). For the composite task, participants performed significantly better with congruent than with incongruent trials only in the aligned condition, but not in the misaligned condition. Correlation analyses revealed that participants with greater N170 fluctuations between upright and inverted face processing demonstrated better performance on the aligned composite task, suggesting that individual differences in global/holistic perceptual processing of faces as early as the N170 time-window constructing a neural representation of faces that might predict behavioral performance of holistic face processing.

**Grant:** Yin Shu-Tien Educational Foundation

## 

**Keywords:** Face perception, Composite task, N170 Component, EEG/ERP

## Recognising Own- and Other- Race Faces Through Static Versus Dynamic Stimuli: Chinese, Malay and Indian Compared

**Hoo Keat Wong**

University of Nottingham Malaysia

## 

To date, many face-related studies have used static images as stimuli; however, faces that we encounter in the real world involve dynamic motions that may affect the way how faces are processed. Previous face studies involving dynamic stimuli in naturalistic settings have revealed a different eye movement pattern, with more fixations directed toward the mouth (e.g., Pillai et al., 2012; Russo et al., 2011; Vo et al., 2012). This study aims to employ own- and other-race face stimuli that closely represent natural social situations to study the own-race bias (ORB) in face memory (for a review, see Meissner & Brigham, 2001). In a classic old-new recognition paradigm, we investigated (a) to what extent observers’ recognition memory is affected by dynamic facial motions and (b) whether observers of different races in a multi-racial population use different eye movement strategies when instructed to recognise dynamic faces as compared to static faces. Our findings revealed that, regardless of stimulus race, dynamic faces not only yielded better recognition performance, but also greater number of fixations on the nose and mouth regions than static face images did. Most interestingly, despite the weak ORB, we detected statistically significant, though subtle, differences in eye movement pattern between race groups.

**Grant:** none

## 

**Keywords:** face recognition, eye-tracking

## Cross-cultural Differences in Sensitivity Detection of Yawning Faces

**Kazufumi Sato^1^, Hassan Matout^1^, Meingold Chan^2^, Yasuhiro Hatori^1^, Ichiro Kuriki^1^, Satoshi Shioiri^1^ and Chiahuei Tseng^1^**

^1^Research Institute of Electrical Communication, Tohoku University

^2^Ohio State University

## 

Yawning is a universal action that involves very distinct facial and body expressions, but why we yawn remains a topic of debate. Past theories emphasized on its physiological functions (e.g. respiratory circulation, brain temperature regulation, arousal), while a newly emerging view suggests a possible social communication function. In this study, we examine the social communication hypothesis by asking whether yawning expression processing shares similar features as other emotional expressions (e.g. happiness, anger). We used the standardized yawning expression stimuli developed by Chan and Tseng (2017) to examine the detection sensitivity in 40 Japanese and Hong Kong students. We discovered that Hong Kong participants had a significantly lower success rate to identify a yawning face than Japanese participants, especially at high intensity images. But such difference was exclusive in yawning expression, not in other types of facial emotion detections (i.e., happiness, anger). Historical literature has suggested that yawning contains different meanings across culture, but whether this contributes to the observation in our behavioral experiment remains unclear. We suggest to follow-up with eye-tracking studies to further elucidate this interesting phenomenon.

**Grant:** Cooperative Research Project Program from Research Institute of Electrical Communication at Tohoku University, and Grant-in-Aid for Scientific Research on Innovative Areas (No.18H04180)

## 

**Keywords:** Face, Social

## Effects of Object Category and Visual Representations on Recognition Accuracy

**Chun-Cheng Hsu**

Institute of Applied Arts, National Chiao Tung University

## 

Some graphics can be easily distinguished and desired, while some cannot be desired or comprehended by the general public. What are the causes behind such outcomes? Relevant investigations in great deal have been found in the gestalt theory, design discipline, ergonomics, and object recognition theory. The purposes of this study are centered on understanding the means of measuring whether a graphic can be easily recognized. A 2 × 2×9 mixed factorial design (N = 360) was conducted to investigate the effect of the three independent variables simplification designs, object category, and participant profile- on the visual recognition. Based on the evaluation results generated from the dependent variable recognition accuracy, the most optimal graphic simplification values could be comprehended. Analysis results of the ANOVA for total scores indicated that the visual recognition accuracy was both significantly impacted by the main effects and interactions of ‘the simplification designs’ and ‘the object category’. The results of this study shown that graphic details were conducive for the facilitation of promoting visual recognition accuracy, however, the visual recognition accuracy could no longer be enhanced, even with an increase in the graphic details, after reaching a certain threshold value. We would recommend the designers pay exceptional attention to the effects exerted by the object category on the recognition accuracy when simplifying the design, especially the simplification of components of natural objects.

**Grant:** none

## 

**Keywords:** Graphic Design, Degrees of Simplification, Shape Feature, Recognition Accuracy

## Oral Session 3-1 (July 31, 2019): Texture

## The Tuning of Early Visual Cortex to the Fractal Structure of Natural Scenes

**Zoey J Isherwood^1^, Colin WG Clifford^3^, Mark M Schira^1,2^ and Branka Spehar^3^**

^1^University of Wollongong

^2^Neuroscience Research Australia

^3^UNSW Sydney

## 

Despite large differences in their visual appearance, natural scenes share many statistical regularities. Firstly, they have similar photometric properties as they share a unique distribution of luminance intensity variations known as the 1/fα amplitude spectrum (α ≈ 1). Secondly, they are similar in their geometric properties as they share a similar density of structure across spatial scales—a property that makes them fractal (e.g., how the branching pattern of a tree is similar across scales). It is currently unclear whether the visual system is reliant on photometric characteristics which change depending on the illumination of a scene—or whether it is tuned to geometry which remains stable. Here we use psychophysics and fMRI to measure responses to three different stimulus image types—greyscale, thresholded, and edges. Each image type shares the same geometric properties, but differ in their photometric properties. Sensitivity and BOLD activity (in visual areas V1–V4) display a characteristic dependency on the geometric properties of an image, peaking for stimuli that had the most natural geometry across all image types despite differences in their photometric properties. This suggests that the visual system is critically reliant on the fractal structure of nature—which remains stable irrespective of scene illumination.

**Grant:** none

## 

**Keywords:** natural scene statistics, fMRI, fractal, early visual cortex

## Antagonistic Receptive-Field Structure of V4 Neurons Detects Local Figure-Ground Organization in Natural Image Patches

**Kouji Kimura^1^, Yukako Yamane^2^, Hiroshi Tamura^2^ and Ko Sakai^1^**

^1^University of Tsukuba

^2^Osaka University

## 

Figure-ground (FG) processing has been suggested to take place in the intermediate-level visual areas. Recently, we have reported that approximately one-third of V4 neurons exhibit the response modulation based on the organization of FG in natural images with respect to their CRF location. To investigate the neural mechanisms underlying the FG modulation, we estimated the spatial structure of their responsive regions corresponding to FG (the receptive field and surrounding region with respect to FG). We presented natural image patches that extended approximately three times larger than the CRF and their variants to the monkeys, and recorded from V4 with 32-channel electrodes. To extract the preferred FG structure of the neurons, we tagged luminance intensity with figure. Specifically, we grouped the natural images with figure regions brighter than ground (bright-figure set), or vice versa (dark-figure set). Spike-triggered average (STA) was computed from each set of stimuli, and the difference was taken to cancel out contrast dependence. The estimated STA showed a facilitative region on the preferred (figure or ground) side including the CRF center, and a suppressive region on the other side, indicating the antagonistic structure for the detection of FG organization in and around the CRF.

**Grant:** none

## 

**Keywords:** Receptive-field estimation, Figure-ground organization, V4, Electrophysiologicalexperiment

## Parietal tACS at Beta Frequency Improves Visual Crowding

**Luca Battaglini^1^, Clara Casco^1^, Andrea Ghiani^1^ and Luca Ronconi^2^**

^1^University of Padova

^2^Vita-Salute San Raffaele University

## 

Crowding can be described as the deleterious influence of nearby contours on visual discrimination. Beta cortical oscillations were found to play a key role in crowding, with a higher beta amplitude related to better crowding resilience. In the present study, we tested the effect of right parietal-tACS at 10 Hz (within the alpha band), 18 Hz (within the beta band) and sham on a letter crowding task, with the prediction that by increasing parietal beta activity would improve performance. Resting electroencephalography was measured before and after stimulation to test the influence of tACS on neural oscillations. An increment in the participants’ performance for parietal 18-Hz tACS, as compared to 10-Hz tACS and sham was measured. This improvement was found specifically in the hemifield contralateral to the stimulation site and was accompanied by increased amplitude of EEG beta oscillations. Furthermore, correct discrimination was associated with a specific phase angle of the ongoing beta tACS at the single trial level. These results support a causal relationship between beta oscillations and visual crowding. Furthermore, they provide evidence that tACS at relevant frequencies can improve crowding-related performance, paving the way for application in clinical conditions such as dyslexia and amblyopia that are severely impaired by crowding.

**Grant:** none

## 

**Keywords:** Crowding, tACS, oscillations

## The Interaction Between Attention and Perceptual Grouping Revealed by Contrast Masking Paradigm

**Chiahuei Tseng^1^, Chien-Chung Chen^2^ and Satoshi Shioiri^1^**

^1^Research Institute of Electrical Communication, Tohoku University

^2^Department of Psychology, National Taiwan University

## 

Attention and perceptual grouping both enhance visual efficiency by selecting relevant information for further processing. They share similar modulations on mid- and high-level visual phenomena and it is likely that they share common mechanisms. In this study, we quantitatively investigated the underlying mechanisms for both attention and grouping. We measured participants’ detection thresholds of a target (2.5 cpd vertical Gabor) on pedestals with same spatial profile but different contrasts. A valid or invalid location cue was presented before the target appeared at one of the four possible locations. Several u-shapes outlines were arranged in a way that they can be grouped into competing configurations based on the applied grouping principles (e.g., similarity and closure). This allows us to assess the relative grouping strength quantitatively. The target threshold vs. pedestal contrast (TvC) function for valid cues was a vertical downward shifted copy of that for the corresponding invalid cue conditions, suggesting that that attentional cueing effect originated from excitatory sensitivity enhancement, not from uncertainty reduction. The TvC functions for grouped and ungrouped conditions showed difference only in high pedestal contrasts, implying a change of divisive inhibition level. Thus, the interaction between attention and perceptual grouping is readily observed by the change of TvC function changes.

**Grant:** Tohoku RIEC Collaborative Research Grant

## 

**Keywords:** Attention, Psychophysics, Perceptual Grouping

## More Efficient Semantic than Phonological Extraction in Reading Chinese/Kanji for Taiwanese/Japanese Skilled Readers

**Su-Ling Yeh^1^, Jen-Tse Dong^1^, Pokuan Ho^1^, Shuo-Heng Li^1^, Te-Chi Huang^1^, Shiho Hirai^2^, Yoshiyuki Ueda^3^ and Jun Saiki^4^**

^1^Department of Psychology, National Taiwan University

^2^Faculty of Education, Kyoto University

^3^Kokoro Research Center, Kyoto University

^4^Graduate School of Human and Environmental Studies, Kyoto University

## 

Previous reading models, regardless of written forms, have largely assumed sequential processing in the order of orthography, phonology, and semantics. However, we found evidence against such assumption from a novel approach, which brings the hierarchical nature of previous models into question. We adopted a visual crowding paradigm where a prime word was crowded by meaningless flankers in the periphery, and it was either a homophone or non-homophone (or semantically related/unrelated) to the target word. In the isolated condition, an isolated prime word was used to compare with results from the crowded condition. Results showed that, while semantic priming occurred in both crowded and isolated conditions, phonological priming was found only in the isolated condition. That is, semantic extraction survives visual crowding and thus semantic priming occurs even with unrecognizable (crowded) primes, but word recognition is required for phonological extraction in reading Chinese. When Japanese participants were tested with Kanji characters in the isolated condition, semantic priming was found but not phonological priming. Taken together, our results indicate better efficiency of semantic than phonological extraction in reading Chinese/Kanji characters, and Chinese/Kanji characters are excellent tools for testing general reading models (e.g., Perfetti, Liu, & Tan, 2005) across different writing systems.

**Grant:** none

## 

**Keywords:** reading, visual crowding, psycholinguistics, Chinese/Kanji characters

## Oral Session 3-2 (July 31, 2019): Attention

## Motion Extrapolation and Time Compression during Eye Blinks

**Gerrit Maus^1^, Hannah Letitia Goh^1^ and Matteo Lisi^2^**

^1^School of Social Sciences, Nanyang Technological University Singapore

^2^School of Health Sciences, City University of London

## 

Eye blinks cause disruptions of the visual input that generally go unnoticed. The brain uses active suppression to prevent awareness of the gaps, but it is unclear how suppression would affect the perception of dynamic events, when visual input changes across the blink. Here we studied the perception of moving objects around the time of eye blinks. In Experiment 1, we presented a moving stimulus that disappeared upon detection of a blink, and instructed participants to indicate the last perceived location of the stimulus. We observed that when motion terminates during a blink, the last perceived location is extrapolated ahead. In Experiment 2, a similar moving stimulus jumped either backward or forward by a variable amount during a blink. Participants were instructed to indicate the perceived direction of the jump. We found that motion trajectories were perceived as more continuous when the object jumped backward during the blink, cancelling a fraction of the space it travelled. This suggests a subjective underestimation of the duration of a blink. These results reveal the strategies used by the visual system to compensate for disruptions and maintain perceptual continuity: time elapsed during eye blinks is perceptually compressed and filled with extrapolated information.

**Grant:** none

## 

**Keywords:** eye blinks, motion, time perception, psychophysics

## Microsaccades Reveal Anticipation of Cognitive Conflict in a Cued-Flanker Task

**Mario Dalmaso, Luigi Castelli and Giovanni Galfano**

University of Padova

## 

Microsaccade are tiny and rapid eye movements that we perform during fixation. In the last years, several studies reported a link between microsaccadic frequency and higher-level cognitive processes, such as attention and memory. Here, we investigated whether microsaccade can be shaped by anticipated cognitive conflict. A version of the flanker task was administered, which is known to elicit cognitive interference. In more detail, at the beginning of each trial, a central colour cue informed the participant regarding the upcoming target frame. In two thirds of the trials, the colour cue reliably informed the participants that in the upcoming trial the flankers either matched the central target letter (e.g., HHH) or not (e.g., HSH). Hence, participants could accurately anticipate whether cognitive conflict would arise or not. In the remaining one third of trials, the colour cue provided no useful information. Taken together, the results showed that microsaccadic rate, time-locked to colour cue onset, was reduced on trials in which an upcoming cognitive conflict was expected. In conclusion, this study shed fresh light on the possible top-down modulations of microsaccade dynamics.

**Grant:** none

## 

**Keywords:** eye movements, microsaccades, cognitive control

## Attentional Blink in 7- to 8- Month-Old Infants

**Shuma Tsurumi^1^, So Kanazawa^2^, Masami K Yamaguchi^1^ and Jun Kawahara^3^**

^1^Chuo University

^2^Japan Women’s University

^3^Hokkaido University

## 

Cognitive models of visual attention propose that perception relies on two-stage processing. The rapid initial stage processes all stimuli as representation followed by the slow resource limited consolidation stage involving working memory (Chun & Potter, 1995). Although this model has been tested in adults and children, it was unclear whether this model applies to preverbal infants’ cognitive systems. To address this question, we investigated the infants’ encoding of working memory during viewing rapid serial visual presentation. First, we examined infants’ ability to identify a target face embedded in visual streams at a rate of 100 ms/item, and found that 7- to 8- month-olds could identify a target face. Next, we tested the attentional blink effect, in which identification of the second of two brief targets is impaired when inter-target lags are short. The temporal lag between the first and second targets were 200 or 800 ms. We found that they could identify both targets at the longer lag but failed to identify the second target at the shorter lag, representing attentional blink comparable to adults. These results suggest that the working memory in 7- to 8 month old infants functions with the same temporal parameters as adults.

**Grant:** none

## 

**Keywords:** attention, development, working memory

## Perceptual Learning Induces Lower Alpha Power to Nonsalient Irrelevant Shapes

**Yulong Ding^1^, Chupeng Zhong^2^ and Zhe Qu^2^**

^1^School of Psychology, South China Normal University

^2^Department of Psychology, Sun Yat-Sen University

## 

Usually, attentional capture is provoked by salient-but-irrelevant stimuli. Our recent study showed that, a nonsalient shape can attract attention automatically as a consequence of perceptual learning. This bottom-up capture of attention was indexed by an involuntary N2pc component, which began at around 170–180 ms after stimulus onset and was predictive of stimulus-specific behavioral improvement. Here, by reanalyzing the electrophysiology data, we investigate whether any earlier top-down attentional effect is generated as a result of perceptual learning of nonsalient shapes. Time-frequency analysis showed that, after perceptual learning, alpha power around the period of stimulus onset was smaller under the trained condition than under the untrained condition. Importantly, this alpha effect was also predictive of stimulus-specific behavioral improvement. However, this alpha effect was not correlated with the following involuntary N2pc effect, and was irrespective of whether the target was presented or not. These results suggest that, after perceptual learning, top-down attentional system could be well prepared for the target ahead of time, and such attentional preparation is specific to the trained shape. Different from the involuntary N2pc, the voluntary effect indexed by alpha power could be considered as another neural mechanism underlying the behavioral improvement after perceptual learning of nonsalient shapes.

**Grant:** National Nature Science Foundation of China (No. 31471070) ; Leading Talents in BaiQianWan Project of Guangdong Special Support Program (No. 201626026)

## 

**Keywords:** visual attention, event-related potential, alpha power, perceptual learning

## Spatial Spread of Visual Attention on a Uniform Random-Dot Field

**Lin Shi^1^, Søren K. Andersen^2^ and Satoshi Shioiri^3^**

^1^Kunming University of Science and Technology

^2^University of Aberdeen

^3^Tohoku University

## 

Visual attention plays a critical role in selecting important information from the large amount of retinal inputs. Basic factors of selection in retinal inputs are the location and extent. Shioiri et al. reported spatial spread of visual attention measured by steady-state-visual-evoked-potential (SSVEP). However, it is possible that their measurements include effect of object based attention because the disks should be perceived as eight objects or as one group object, which may influence the extent of spatial attention. Here, we designed a stimulus without salient features to shape objects and measured spatial spread of visual attention by a similar paradigm as Shioiri et al. The stimulus is random-dot pattern divided into eight sectors flickering with different temporal frequencies. The random-dots suppresses the visibility of borders between sectors so that the stimulus appears to be uniform to the observer. Observer’s detected infrequent targets in a rapid serial visual presentation (RSVP) at attended locations. The spatial profile of visual attention was similar to that found in Shioiri’s independently of task difficulties. These results suggest that visual attention spreads spatially broadly in the absence of salient objects or borders.

**Grant:** none

## 

**Keywords:** Visual attention

## Transient Attention Does Change the Appearance: Excluding the Response Bias

**Fang Zhang^1^, Yongchun Cai^1,2^, Liufang Zhou^1,2^**

^1^Zhejiang University

^2^Huanan Normal University

## 

Previous studies have shown that transient attention enhances the perceived contrast of a low-contrast stimulus but attenuates the perceived contrast of a high-contrast stimulus. However, it is still unclear whether such an effect was genuinely perceptual or was due to response bias. In this study, participants were shown with a pair of grating stimuli, following a peripheral cue which directed exogenous attention to the left or the right. After the grating offset, an additional response cue was introduced to determine the response direction: participants were asked to report which stimulus looks higher or lower in contrast. This response cue was designed to eliminate the response bias as no particular response tendency towards left or right should be made before the instruction. By using psycho-physical methods, the apparent contrast of a standard stimulus was measured and compared when attention was directed to its location or its opposite location. The results successfully replicated previous findings, showing an enhancement effect at a low contrast level (attended 26.81% vs. unattended 22%) and an attenuation effect at a high-contrast level (attended 51.66% vs. unattended 60%). Furthermore, our results offered empirical evidence for supporting the view that human perceptual experience is modified by attention.

**Grant:** none

## 

**Keywords:** psychophysics, attention

## Perceived Time and the Accrual of Visual Information

**Kielan Yarrow^1^, Isla Jones^1^, Alan Johnston^2^ and Derek H Arnold^3^**

^1^Department of Psychology, City, University of London

^2^School of Psychology, University of Nottingham

^3^School of Psychology, The University of Queensland

## 

When people time intervals, they may be integrating some quantity directly related to the richness of sensory information (e.g., memories; change). This perspective suggests that (illusory) temporal dilation is corollary to an increase in the rate of information uptake. Such an increase in “bit rate” formed the original explanation for the temporal oddball illusion (TOI), where an infrequent stimulus embedded in a sequence of repeating standards appears of relatively greater duration. To test this account, we measured the TOI for the final stimulus in a sequence (contrasting oddballs with repetitions). We then assessed contrast discrimination (d′) under identical conditions. Critically, we also measured the linear increase in d’ that occurs when sub-second exposure duration is varied. This allowed us to predict how the subjective dilation implied by each participant’s TOI should affect discriminability, if both depend on identical information. The TOI was accompanied by increases in d′. However, this discriminability boost significantly exceeded predictions. Furthermore, the two effects failed to correlate across participants. We conclude that one or more factors (e.g., exogenous attention; repetition suppression) influenced two independent mechanisms (an internal clock, and a contrast detector) rather than a coupled system.

**Grant:** none

## 

**Keywords:** time perception, psychophysics

## Reading Direction Influences the Deployment of Visual Attention during Word Processing

**Honami Kobayashi and Hirokazu Ogawa**

Kwansei Gakuin University

## 

Previous studies demonstrated that the orthographic property of a N+1 word can be processed in parallel with the lexical processing of a currently attended N word. This bias towards the right-side word has been hypothesized to be consistent with a reading direction in alphabetical languages. We tested this hypothesis by using the flanker lexical decision task with Japanese words that were aligned horizontally or vertically. The results demonstrated that the traits of target words affected lexical decision, which is in line with the previous studies in alphabetical languages. In addition, the flanker words right of or below the target (i.e., N+1 words) were processed to the orthographic level in parallel with the target, in contrast to the previous studies that showed the lack of parallel processing of the word presented below a target. The contrast may be due to the fact that, unlike alphabetical languages, Japanese language can be written both vertically and horizontally. These findings highlight the possibility that variation in writing systems influences how attention is allocated during word processing.

**Grant:** none

## 

**Keywords:** word processing, attention

## Oral Session 3-3 (July 31, 2019): Appearance

## Divisive Inhibition Determines Orientation Discrimination Threshold after Adaptation to Center-Surround Sinusoidal Stimuli

**Yih-Shiuan Lin^1^, Chien-Chung Chen^2,3^ and Mark W. Greenlee^1^**

^1^Institute of Psychology, University of Regensburg

^2^Department of Psychology, National Taiwan University

^3^Neurobiology and Cognitive Science Center, National Taiwan University

## 

Ample evidence supports the claim that center-surround interactions in spatial vision is subject to a normalization process. We studied such lateral modulation in peripheral vision with an adaptation paradigm. Previously, we found that orientation discrimination thresholds of Gabor targets elevated after adaptation to a sinusoidal-grating adapter. Moreover, adding a surround annulus to the adapter decreased the adaptation effect, suggesting a center-surround inhibition during adaptation. To investigate whether this inhibition is orientation-specific, we varied the center and surround orientations of the adapter independently from 0° (vertical) to 90° (horizontal) in the counter-clockwise (CCW) direction. We found that the CCW threshold was the greatest when the adapter center had an orientation of 11.25°, regardless of the surround orientation. The threshold decreased as the adapter surround orientation approached that of the center. Our results indicate that the magnitude of lateral inhibition depends on the surround orientation. We fitted a divisive inhibition model to the data. The model contains an array of orientation selective mechanisms whose response corresponds to the stimulus elicited excitation, raised by a power, divided by an inhibitory component plus a constant. The surround modulation in the adapter is represented by a multiplicative parameter that captures sensitivity modulation between center-surround mechanisms.

**Grant:** none

## 

**Keywords:** spatial vision, computational model, lateral inhibition, psychophysics

## Falling Pitch Imitating Doppler Shift Facilitates Detection of Visual Motion in The Extreme-Periphery

**Takashi Suegami^1,2^, Mark Changizi^3^, Christopher C Berger^2^, Daw-An J Wu^2^ and Shinsuke Shimojo^2^**

^1^Yamaha Motor Corporation U.S.A

^2^California Institute of Technology

^3^2ai Labs

## 

Previous studies demonstrated that concurrent auditory stimuli can bias visual motion perception in the periphery more than in the fovea (e.g., Takeshima & Gyoba, 2013), and auditory becomes crucial when reliability of vision is reduced (e.g., Schmiedchen et al., 2012). We investigated if auditory affects detecting extreme-peripheral visual motion from behind, which is possibly one of the most salient situations since visual ambiguity is very high and detecting such motion can be ecologically critical to survive. In the experiment, a sequence of three 204 ms dots (255 ms SOA) was presented in the extreme-periphery (individually set by the largest eccentricity with 75% detection); each dot was presented at 3 adjacent locations with 2˚ distance so as to have apparent motion forward, or at the same location. As auditory stimuli, we employed concurrent beep with falling pitch, which roughly imitated Doppler pitch shift for passing-by object. We employed concurrent beep with rising pitch as a control, in addition to another no sound control. The results showed the concurrent beep with falling pitch increased the hit rate for motion detection, relative to that with no sound and rising pitch beep. Underlying mechanism was discussed with signal detection analysis.

**Grant:** Yamaha Motor Corporation U.S.A.

## 

**Keywords:** periphery, cross modal, motion detection, visual perception

## Motion-Generated Optic Flow Facilitates Perception When Visual Images are Blurry

**Jing Pan^1^, Hongge Xu^1^, Xiaoye M Wang^2^ and Geoffrey P Bingham^2^**

^1^Sun Yat-sen University

^2^Indiana University

## 

Traditionally, visual functioning is thought to correlate with visual acuity, and clear images precede successful event or scene identification. In natural viewing, however, there are two sources of optical information: static images and motion-generated optic flow. Each specifies spatial structures. Each functions largely independently of the other. When optic flow and blurry images coexist, they interact and yield effective and stable perception, because the detection of flow is unaffected by image blur and optic flow compensates for the loss of image details. In two studies, we tested how events and scenes were identified when visual images were blurry and with relative motions between observers and world structures. We found that observers did not identify events or scenes from static blurry images (as an experimental manipulation), but they did when motion, and thus optic flow, were added. We quantified optic flow strength and showed that it correlated with identification performances. Once observers had identified the events or scenes with motion, they continued to identify them from static blurry images, which previously did not afford identification. We replicated these results in age-related macular degeneration and amblyopic patients. Thus, optic flow and visual images should both be considered in visual assessment and rehabilitation.

**Grant:** National Science Foundation of China 31571116

## 

**Keywords:** Optic flow, Low vision, Event perception, Scene perception

## Eye Movement Correlates of Accurate Recognition of Balanced Painting Composition

**Piotr Francuz, Iwo Zaniewski, Paweł Augustynowicz, Natalia Kopiś and Tomasz Jankowski**

The John Paul II Catholic University of Lublin

## 

Aesthetic experience is a result of many factors accompanying the appreciation of a work of art. Two of them seem particularly important: the quality of the work of art and the level of expertise of the viewer. We were interested in whether visual arts experts more accurately recognize a balanced composition in one of the two paintings being compared simultaneously, and whether people who correctly recognize harmonious paintings are characterized by a different visual scanning strategy than those who do not recognize them. In other words, the aim of this study was to search for eye movement correlates of expertise in visual arts. We found that the eye movements of people who more accurately appreciated paintings with balanced composition differ from those who more liked their altered versions due to dwell time, first and average fixation duration and number of fixations. The familiarity of paintings turned out to be the factor significantly affects both the aesthetic evaluation of paintings and eye movement. To sum up, the experimental manipulation of comparing pairs of paintings, whose composition is at different levels of harmony, has proved to be an effective tool for differentiating people because of their expertise in visual art.

**Grant:** The National Science Center (Poland, UMO-2013/11/B/HS6/01816) and the Polish Ministry of Science and Higher Education (1/6-1-17-0501-8126) grants.

## 

**Keywords:** expertise in visual arts/eye movement

## Mccollough-Effect Induced Illusory Colour Biases Binocular Rivalry

**Shuai Chang^1,2^ and Joel Pearson^1^**

^1^School of Psychology, University of New South Wales

^2^School of Psychology, South China Normal University

## 

McCollough effect is a visual phenomenon that after adapting to two orthogonal gratings with complementary colour, individuals will have subjective experience of colour once they are presented with an achromatic grating in the orientation of either of the two prior stimuli. The colour experience induced by the McCollough effect can be categorised as a form of phantom colour, since it was not directly based on the external colour stimuli. After viewing the McCollough phantom colour for 4 seconds against a dark background, participants reported a significant perceptual bias in the subsequent colour rivalry perception, but the bias decreased to chance level when it was on a bright background. Further experiment showed that the direction of the perceptual bias depended on the contrast of the grating inducers. Whereas medium contrast induced a facilitatory effect, higher contrast induced suppression. In addition, the perceptual biases in the rivalry perception induced by neon phantom colour and voluntarily imagined colour had significant correlation, but neither of them correlated with the bias induced by McCollough effect. The current study demonstrates the perceptual nature of involuntary phantom vision and suggested that there were similarities and differences between voluntary and involuntary forms of phantom vision.

**Grant:** none

## 

**Keywords:** McCollough Effect, Phantom vision, binocular rivalry, visual perception

## Light Sources as Scene Components: Estimating Light Source Direction in Scenes with Multiple Objects

**Lindsay M Peterson^1^, Daniel J Kersten^2^ and Damien J Mannion^1^**

^1^UNSW Sydney

^2^University of Minnesota

## 

Humans can identify properties of a light source from an image of an illuminated object. However, it is unclear whether this capacity is based on an inference about the light source as a scene component or about the lighting conditions that are local to an object. We predicted that the precision of a scene-based light source representation would be enhanced by the presence of multiple objects. We presented observers (n = 28) with rendered scenes containing 1, 9, or 25 unfamiliar blob-like objects and measured their capacity to discriminate whether a directional light source was left or right of the vantage point. Consistent with a scene-based lighting representation, we find that the presence of more objects was associated with an increase in discrimination accuracy. However, we also find that renderings without cast shadows were associated with a decrease in discrimination accuracy with more objects. We suggest that the presence of more objects reduces the fidelity of the information from each object, and that this reduction occurs to an extent in the absence of cast shadows that outweighs the benefits from integrating across multiple objects. Future experiments will use equivalent noise methods to distinguish these contributions to illumination direction estimation accuracy.

**Grant:** none

## 

**Keywords:** illumination, psychophysics

## The Effect of Color Temperature on the Color-Dependent Fraser–Wilcox Illusion

**Akiyoshi Kitaoka**

Ritsumeikan University

## 

The color-dependent Fraser–Wilcox illusion is a motion illusion observed in stationary images that is characterized by perceptual dimorphism (Kitaoka, 2014). The direction of illusory motion is reversed depending on illuminance when we observe printed images. Here I report a new finding that this perceptual dimorphism is affected by color temperature. High color temperature promotes the motion illusion observed in a bright condition, while low color temperature enhances the illusion observed in a dark condition.

**Grant:** none

## 

**Keywords:** motion, color, illusion

## Poster Session 3 (July 31, 2019)

## Visual Working Memory Load Affects Visual Detection: Attention Resource Competition or Cortical Resource Competition?

**Nailang Yao, Yang Guo and Zaifeng Gao**

Zhejiang University

## 

It has been revealed that visual working memory (VWM) load reduces the detection of a low-priority target. This fact has been explained in terms of VWM load competes visual attention with the detection task (visual attention hypothesis). However, an alternative explanation existed for this finding: since processing visual representations in VWM requires the involvement of primary visual cortices, which also plays a key role in visual detection, the reduced detection was due to the competition of the resources of visual cortex (cortical resource hypothesis). To test this alternative, we required the participants to memorize both color and shape of the stimuli (feature load), or the bindings between color and shape (binding load). Moreover, a low-priority task was added in the maintenance phase of VWM. It has been found that extra attention was required to process binding representations than the constituent single features. The visual attention hypothesis predicted that the detection performance in the binding load was worse than that in the feature load condition, whereas the cortical resource hypothesis predicted a non-difference between the two conditions since the same visual cortices were employed. In line with the prediction of cortical resource hypothesis, we did not find any difference between the two load conditions. Meanwhile, once the memory load was manipulated in terms of number of visual features, we re-established the impaired detection under high feature load.

**Grant:** National Natural Science Foundation of China (No. No.31771202)

## 

**Keywords:** Visual working memory load, Visual detection

## Active Suppression of Nonsalient Irrelevant Shapes Induced by Perceptual Learning

**Zhe Qu^1^, Liping Hu^1^ and Yulong Ding^2^**

^1^Department of Psychology, Sun Yat-Sen University

^2^School of Psychology, South China Normal University

## 

Visual attention can be attracted by salient-but-irrelevant features, a phenomenon called attentional capture. Accompanying with attentional capture, a top-down inhibitory mechanism is usually enacted to suppress the attentional shift. Our recent study showed that, a nonsalient shape can also provoke a robust capture of attention as a consequence of perceptual learning. It remains unclear, however, whether the brain would initiate an active suppression process to a nonsalient irrelevant shape which could capture attention. Here, we show that detectability of a shape may be a key factor determining whether the shape would be actively suppressed or not. After extensive training as a target in visual search, a nonsalient shape could elicit an N2pc when it was an irrelevant stimulus in a visual search task or an RSVP task, indicating a capture of attention induced by perceptual learning. Following the N2pc, a Pd would be elicited by the irrelevant shape only if its detectability is relatively high. These findings suggest that an active suppression process could be applied not only to salient features, but to nonsalient shapes. A nonsalient shape could improve its salience through perceptual learning and would be actively suppressed when its learned salience reaches to some certain level.

**Grant:** National Nature Science Foundation of China (No. 31471070) ; Leading Talents in BaiQianWan Project of Guangdong Special Support Program (No. 201626026)

## 

**Keywords:** visual attention, perceptual learning, event-related potential, Pd

## The Effects of Attentional Area Size and Its Mean Luminance on Pupillary Response

**Xiaofei Hu, Rumi Hisakata and Hirohiko Kaneko**

Department of Information and Communications Engineering/Tokyo Institute of Technology

## 

It has been shown that pupil constricted more when people attended to a narrow area compared with a spread area for the same stimulus. It was explained by the effect of selective attention area. However, another possibility was that the mean luminance within the attended area decided the magnitude of pupillary response and it was the higher luminance that elicited a smaller pupil when people narrowed their attention in the Navon figure used. In this study, we focused on both the attentional area and its mean luminance. In Experiment 1, we controlled participant’s attentional area by presenting two imaginary circles with different radiuses consisting of eight disks placed equidistantly. The radius of the small circle was fixed and that of the large circle varied. They were presented simultaneously and participants were instructed to attend to one of them. In Experiment 2, the radiuses of the circles were fixed and the size of outer disks varied to change the mean luminance. Results showed that the attentional area didn’t affect pupillary response, while the distribution of stimulus luminance did. We conclude that the pupillary response is independent of the selective attention area itself, at least for the present stimulus condition.

**Grant:** none

## 

**Keywords:** pupillary response, attention, psychophysics

## Attentional Updating of Perceived Position Can Account for a Dissociation of Perception and Action

**Ryohei Nakayama^1,2^ and Alex O. Holcombe^1^**

^1^School of Psychology, The University of Sydney

^2^Center for Information and Neural Networks (CiNet), NICT

## 

It has been proposed that the neural pathways for perception and action depend on distinct visual information. In support of this notion, recent studies reported that although internal grating motion can accumulate over seconds into a large illusory position shift, this position shift is not reflected in saccade targeting (action). Another possibility however is that rather than saccades and other actions having privileged access to the correct position, the attention shift thought to precede saccades resets the accumulated position shift to zero. Here we found that the accumulation of illusory position shift is reset by transients near the moving object, resulting in a striking impression of the object jumping back to its actual position. The object motion is also perceived as having a veridical direction rather than a shifted direction when transients are given repetitively. The position shift however accumulates to alter the perceived direction despite repetitive transients when observer’s attention is removed by a concurrent letter identification task. These results suggest that attention can update the perceived position of moving objects and mediate the previously reported dissociation between perception and saccades.

**Grant:** none

## 

**Keywords:** motion, attention, psychophysics

## Individual Differences of The Collinear Masking Effect in Visual Search

**Pei-Chen Ye and Li Jingling**

China Medical University

## 

The ability to inhibit irrelevant distractors is crucial in efficient visual search, and was shown to vary with individual’s working memory capacity and anxiety. The current study examined whether such individual differences also exist in the collinear masking effect, which refers to the phenomenon that a target can be concealed when it overlaps with a salient but task-irrelevant collinear structure. Sixty participants completed a visual search task to probe the collinear masking effect, the Chinese Mandarin version State-Trait Anxiety Inventory Y form to estimate personal anxiety, and the backward digit span to probe working memory capacity. Results showed that the larger the collinear masking effect, the stronger the state anxiety score, r = .74, p = .XX. Meanwhile, trait anxiety score and backward digit span performance did not correlate to the size of the collinear masking effect. Our results suggest that the collinear masking effect, similar to the distractor suppression or attentional capture effect in visual search, related to current anxiety strength. Thus we concluded that the ability to inhibit the search for distractors may be the cause of individual differences in collinear masking effects. Our study may thus reveal the individual differences on camouflage.

**Grant:** none

## 

**Keywords:** Attentional capture, working memory capacity, anxiety, distractor suppression

## Attempt on the Measurement of Spatial Extent of Audiovisual Attention by EEG

**Shin Ono^1^, Shuichi Sakamoto^1,2^, Ryo Teraoka^1^, Yoshiyuki Sato^2,3^, Yasuhiro Hatori^1,2^, Chiahuei Tseng^1,2^, Ichiro Kuriki^1,2^, Satoshi Shioiri^1,2,3^**

^1^Graduate School of Information Sciences, Tohoku University

^2^Research Institute of Electrical Communication, Tohoku University

^3^Advanced Institute for Yotta Informatics, Tohoku University

## 

Several studies of crossmodal attention showed that attention to a visual stimulus facilitates the processing of an auditory stimulus that was presented at the same location, and vice versa. Since these crossmodal facilitation in behavioral performance depends on the spatial distance between visual and auditory stimuli, spatial characteristics are important to understand the mechanisms of crossmodal attention. Here, we developed an experimental procedure to measure the spatial extent of crossmodal attention with Steady-State Responses (SSRs) of electroencephalogram. In our experiments, participants were presented with letters as visual stimuli and vowels as auditory stimuli simultaneously. Participant’s task was to attend one location to detect either visual or auditory target appearing at the attended location. SSRs of visual and auditory stimuli at multiple locations were measured simultaneously to estimate the spatial extent of attention. We succeeded to measure the spatial extent of unimodal attention peaking near the attend location, while no clear crossmodal attention was found in the present condition. This failure to find the crossmodal attention was possibly because the binding between audio and visual stimuli was weak in our experimental conditions.

**Grant:** none

## 

**Keywords:** crossmodal attention, EEG, visual, auditory

## Task Difficulty at Fixation Location Modulates Attentional Bias Caused by Head Direction

**Ryoichi Nakashima^1,2^, Takatsune Kumada^2,3^**

^1^The University of Tokyo

^2^RIKEN

^3^Kyoto University

## 

During eccentric gaze, visual perception of a stimulus appearing in front of the head is facilitated. This indicates that attention is biased toward the natural eye direction, which is an involuntary attentional bias based on eye movement control by ocular-muscle constraints. The present study examined whether the intention to attend to a specific position influences the attentional bias caused by head direction. We conducted a target identification task where visual stimuli briefly appeared to the left and right of a fixation point, and manipulated the head direction relative to the fixation position. We included filler trials where a target stimulus appeared at the fixation position (the central task), manipulating the size of the stimulus (large/small) to vary the difficulty of the central task. It was assumed that observers would intend to focus more attention on the fixation position when the central task was difficult. Results confirmed the attentional bias based on the head direction under both conditions. However, the degree of this effect was smaller in the difficult central task condition than in the easy central task condition. These findings indicate that the influence of head direction on visual attention during eccentric gaze is modulated by intentional attention control.

**Grant:** none

## 

**Keywords:** attention, visual perception

## Does Visual Experience Abolish Search Asymmetry?

**Chia-Chun Tsai^1^, Sung-En Chien^1^, Yoshiyuki Ueda^2^, Jun Saiki^3^ and Su-Ling Yeh^1^**

^1^Department of Psychology, National Taiwan University

^2^Kokoro Research Center, Kyoto University

^3^Graduate School of Human and Environmental Studies, Kyoto University

## 

Search asymmetry is a change in search efficiency when target and distractors switch roles, and it has been considered as a diagnosis of basic feature or signal strength. However, most studies recruited western participants and indeed, found faster reaction times and shallower slopes in searching a long line among short lines than vice versa. Yet, this phenomenon was not observed in Japanese participants (Ueda et al., 2018), suggesting that visual experiences may affect the performance in search asymmetry. In Experiment 1, Taiwanese participants were asked to perform a line length search task. In Experiment 2, a session of English digit search where participants searched for Arabic number among English letters was added before each line search task, inducing a priming experience of English letters. Results from Experiment 1 showed no search asymmetry, similar to Japanese data from Ueda et al. (2018). Critically, we found faster reaction times for short lines than long lines, opposite to the results obtained from English users. In Experiment 2, Taiwanese participants still showed no search asymmetry but the difference in reaction times between the two kinds of lines disappeared. Conventional theories of search asymmetry need to modify to accommodate the malleable nature of search performance.

**Grant:** none

## 

**Keywords:** search asymmetry, culture

## Agent Identity Affects the Encoding of Biological Motion into Visual Working Memory

**Quan Gu, Jingyin Zhu, Mowei Shen and Zaifeng Gao**

Zhejiang University

## 

To recognize and predict social interactive behaviors, we have to encode human biological motions (BMs, e.g., walking and waving), into visual working memory (VWM). Critically, each BM in real life is produced by a distinct person, carrying a dynamic motion signature (i.e., identity). Whether the agent identity influences VWM processing of BMs remains unknown. Here we addressed this question by examining whether memorizing BMs with different identities promoted the VWM processing of task-irrelevant clothing colors. Two opposing hypotheses were tested: (a) VWM only stores the target action (element-based hypothesis), and (b) VWM stores both action and irrelevant clothing color (event-based hypothesis), interpreting each BM as an event. We required the participants to memorize actions that either belonging to one agent or distinct agents, while ignoring clothing colors. Then we examined whether the color was also extracted into VWM by probing a distracting effect: If the color was extracted, the change of color in probe would lead to a significant distracting effect on the action recognition. We found that VWM encoding of BMs was adaptive: Once the memorized-actions had different identities, VWM adopted an event-based encoding mode regardless of memory loads and probe identity. However, VWM used an element-based encoding mode when memorized-actions shared the same identity. Overall, these findings suggest that agent identity information has a significant effect on VWM processing of BMs.

**Grant:** none

## 

**Keywords:** identity, biological motion, visual working memory, adaptive processing

## An fNIRS-Based Investigation of Brain Activity Related to Focal Attention

**Tomoki Matsumoto, Aoi Kobayashi, Hisashi Yoshida and Takeshi Kohama**

Kindai University

## 

In order to establish a neuro-rehabilitation evaluation method for attentional dysfunction, we evaluated brain activity in right hemisphere during a dual-cued target detection task using functional near-infrared spectroscopy (fNIRS). It is well known that the impairment of the maintenance of focal attention brings various interferes in everyday life or social life. In the neuro-rehabilitation for attentional dysfunction, recovering the function in the brain area related to maintain focal attention is considered effective. In this study, we conducted a dual-cued target detection task which controls subjects’ attentional allocation with sequential presentation of a symbolic-cue and a direct-cue in the same trials. Two experimental conditions were implemented: (1) ignore direct-cue and maintain attention toward symbolically cued location and (2) redirect attention to directly cued location and ignore symbolic-cue. After confirming the cue effects based on the response time, fNIRS measurements were carried out. The results indicate that when subjects intentionally maintain their focal attention to the symbolically cued direction, the activity in the right prefrontal cortex increased and in the right temporo-parietal region decreased. This suggests that brain activation related to maintain focal attention can evaluate based on the fNIRS measurements.

**Grant:** none

## 

**Keywords:** fNIRS measurement, attention, Frontal lobe region, Prefrontal cortex

## The Effect of Bottom-Up Attention in the Frequency of Microsaccade

**Shogo Noguchi, Chinatsu Toriyama, Hisashi Yoshida and Takeshi Kohama**

Kindai University

## 

In order to investigate the properties of bottom-up attention induced microsaccades, we conducted an experiment in which subjects’ maintain their fixation while passively viewing of randomly presented peripheral spot lights. It has been suggested that focusing or reallocating of attention while actively fixating visual stimuli modulate the frequency of microsaccades. However, little is known about attentional modulation on the frequency of microsaccades in regards to passively viewing condition. In our experiment, the subjects instructed to fixate a crosshair pattern presented on the center of a LCD display and passively viewed peripherally displayed targets. The brightness of the target was changed stepwise around the predetermined perceptual threshold of each subject. The number of targets presented in each trial was randomly determined between 3 and 5. Microsaccades were detected by using a order-statistic time-window analysis (Ohtani et al., 2016) and the frequency transitions of microsaccades associated with the onset of the target were analyzed. The results indicate that around 300 ms after the onset of the target, the occurrence of microsaccades show transient suppression and rebound. The property of the frequency transition of microsaccades in this study consists with that in actively viewing condition shown in previous studies.

**Grant:** none

## 

**Keywords:** Microsaccade, attention, fixation eye movement, passive vision

## An Autoregressive Mathematical Model of Neuronal Spiking Responses

**Suguru Hirai^1^, Akihiro Masaoka^2^ and Takeshi Kohama^1^**

^1^Kindai University

^2^Tamagawa University

## 

Higher-order brain functions such as visual cognition are extremely complex. Recent studies have shown that constructive approaches are highly advantageous to understanding such brain mechanisms. Masaoka & Kohama (2018, in Japanese) described a neuron network model which reproduces the attentional modulation in several brain regions using an autoregressive mathematical representation. This model is configured with several functional unit which is correspond to specific brain regions. Such brain regions are actually constructed by networks of many individual neurons. Masaoka model, however, is a macroscopic expression of the regional brain activity and does not consider individual neuron responses. In this study, we proposed a modified Masaoka model to reproduce various spiking properties of individual neurons which can share mathematical expressions between macroscopic and microscopic brain functions. We evaluated the output of our proposed model by comparing with the output of Izhikevich model, which is known to be highly reproducible of neuron responses. The simulation results show that our model reproduces the various spiking response of neurons sufficiently.

**Grant:** none

## 

**Keywords:** Neuron model, Spike response, Autoregressive mathematical expression, Constructive approach

## Eye Movement Patterns When Driving in Virtual Reality Environment

**Bo Du^1^, Yiik Diew Wong^2^ and Hong Xu^1^**

^1^Psychology, School of Social Sciences, Nanyang Technological University

^2^Centre for Infrastructure Systems, School of Civil and Environmental Engineering, Nanyang Technological University

## 

Human errors are the major cause of most traffic accidents. Previous studies have investigated drivers’ visual features in hazardous driving conditions (e.g., tunnel and raining). However, few studies attempted to link drivers’ visual attention and mental workload under those situations. The current study examines drivers’ eye movement patterns in various roads and aversive weather conditions. We tested the driving behaviour and eye movement of 18 drivers when driving in virtual environment by a highly controlled driving simulator. The driving scenarios were simulated based on real driving route in Singapore and included open road and underground tunnel in rainy and sunny conditions. We found larger perceived saccadic velocity/acceleration/dispersion in open road than tunnel driving in both weather conditions; larger pupil size and longer blink duration in tunnel than open road in rainy condition. These findings indicate higher mental workload in tunnel than open road driving. Moreover, larger pupil size but fewer fixation numbers (negatively correlated with fixation duration) was observed in rainy than sunny driving in open road, indicating higher mental workload in rainy than sunny condition. Our findings of increased mental workload in tunnel and rainy driving than open road and sunny driving provide insights for autonomous vehicle designs.

**Grant:** none

## 

**Keywords:** Visual attention, Virtual Environment, Mental Workload, Human-vehicle interaction

## Age-Related Effects of Cross-Modal Duration Perception

**Fang Jiang, Dustin Dutcher, Daniela Lemus and Alexandra N Scurry**

University of Nevada

## 

Reliable duration perception of external events is necessary to perform daily functions, such as perception and action coordination and speech discrimination. Visual duration perception can be heavily influenced by concurrent auditory signals, however age-related effects on this cross-modal influence has received minimal attention. Recently, we examined the effect of aging on cross-modal duration perception by quantifying 1) duration discrimination thresholds, 2) auditory temporal dominance, and 3) visual duration expansion/compression effects induced by an accompanying auditory stimulus of longer/ shorter duration. Natural aging did not impact duration discrimination thresholds or auditory dominance over visual duration perception, however older adults did perform worse than young adults when comparing durations of two target stimuli (e.g., visual) in the presence of distractors from the other modality (e.g., auditory). Older adults also exhibited more robust visual duration expansion over a wider range of auditory durations compared to their younger counterparts, likely due to age-related enhancement in multisensory integration or inflexible decision strategies. Follow up experiments in young adults reveal expanded visual expansion percepts for more complex stimuli that allow a wider window for audiovisual integration. Results are discussed in terms of multisensory integration and possible decision strategies that differ with age.

**Grant:** EY023268 to FJ, 5U54GM104944, and P20 GM103650

## 

**Keywords:** Cross-modal duration perception, Aging, Multisensory integration

## Manual Reproduction of Visual and Auditory Pulse Sequence

**Gaku Sugawara and Ikuya Murakami**

The University of Tokyo

## 

Studies have shown features of sensorimotor synchronization for periodical pulses and mechanisms underlying the perception of short durations. We examined how accurately subjects reproduced the frequency (i.e., pulses per second) of visual and auditory pulses in three conditions: visual flashes periodically presented at the same position, visual flashes periodically presented at different positions from flash to flash, and auditory periodical clicks. In each trial, the ISI was chosen from six varieties within 140∼490 ms, and the subjects were requested to reproduce the frequency with finger tapping right after the stimulus sequence. In all conditions, reproduced frequencies in different ISI conditions differed from each other despite ISI difference being 40 ms at the minimum. Between the visual and auditory conditions, the reproduced frequencies exhibited significant differences at the three shortest ISIs, with the frequency being slightly under-reproduced for the visual flashes. However, the position(same/different) of the flashes made no difference. These results suggest that the mechanism underlying periodicity perception accurately retains timing information even if motor synchronization is not performed during stimulation, and that the responsible mechanism in the visual pathway is well above the level of temporal-frequency filtering that is only sensitive to events occurring within small receptive fields.

**Grant:** Supported by JSPS KAKENHI (JP18H05523).

## 

**Keywords:** Psychophysics

## Color and Motion Together Lead to Effective Perception of Blurry Events

**Hongge Xu^1^, Jian Wang^1^, Xiaoye Michael Wang^2^ and Jing Samantha Pan^1^**

^1^Department of Psychology, Sun Yat-sen University

^2^Department of Psychological and Brain Sciences, Indiana University, Bloomington

## 

Events consist of objects in motion. When viewing events, observers simultaneously receive static image information projected from objects and dynamic optic flow generated by motion. We investigate how the two contribute to perceiving blurry events. In Experiment 1, observers first identify common events from blurry colored images. The rate of identification was 49% and the eye fixations overlapped with saliently colored areas. When seeing blurry events in colored animations, accuracy increased to 97%. After motion stopped, when observers saw static blurry colored images again, the rate of identification was 94%. In the motion and post-motion conditions, observers’ fixations were mainly in areas with strong optic flow. This suggested that optic flow helped identifying blurry events and the effect of flow was preserved in blurry images. In Experiment 2, we removed colors and found similar trends. When first viewing static blurry grayscale images, observers identified 0.07% of events. By adding motion, this rate increased to 72%. After motion stopped, when seeing static blurry grayscale images again, the rate was 61%. Eye movement analysis suggested that when fixated on areas with strong optic flow, identification was better. Comparing the two experiments, optic flow enabled perceiving blurry events, but color made it better.

**Grant:** none

## 

**Keywords:** event perception, optic flow, eye tracking, low vision

## Effect of Stimulus Duration on Audio-Visual Temporal Recalibration

**Yaru Wang and Makoto Ichikawa**

Chiba University

## 

If visual and audio stimulus are repeatedly presented with a certain temporal gap for a few minutes, the perceived temporal gap between the stimuli would be reduced. This phenomenon is called as “audio-visual temporal recalibration”. Many previous studies found significant audio-visual temporal recalibration for stimuli with short duration while effects of stimulus duration on the audio-visual temporal recalibration. Here we investigated how the presentation of different durations of auditory sound and visual flash affects the audio-visual temporal recalibration. We prepared the short duration condition (500 ms visual flash was combined with 520 ms auditory sound) and long duration condition (20 ms visual flash was combined with 20 ms auditory sound). After exposure to a fixed time lag(0 ms or 230 ms) between the auditory and visual stimuli for about 3 minutes, participants judged whether the onset of the visual flash or that of auditory sound is the first. We found that the audio-visual recalibration for the short duration condition was larger than that for the long duration condition. These results suggest that the long duration of audio-visual may inhibit the audio-visual temporal recalibration. The bases of the effect of stimulus duration upon the audio-visual temporal recalibration will be discussed.

**Grant:** none

## 

**Keywords:** constant method, probit analysis, point of subjective equality

## Temporal Judgements under Conditions with and Without Conscious Perception

**Sofia Lavrenteva, Taro Miura and Ikuya Murakami**

Department of Psychology, The University of Tokyo

## 

When an odd stimulus is presented in a train of visual stimuli each having the same duration, the perceived duration of the oddball is longer than that of other standard stimuli. We investigated whether this effect requires conscious perception of the difference between standard and oddball stimuli. We presented stimulus trains consisting of digital letters in the periphery of the visual field. The target letter was presented either alone (“single” condition), or with two flanker letters located laterally in a way that the target letter could or could not be identified (“multiple” and “crowding” conditions respectively). The first three stimuli in each train were standard letters, and the last letter was the test letter, similar to the standard in half of trials and different (oddball) in the other half. We asked participants to judge whether the test letter had a longer or shorter duration than the standards and then to discriminate the test letter. The perceived duration was longer for the oddball stimulus, and in the crowding condition, this dilation effect positively correlated with the correct response rate in the discrimination task. These results emphasize the importance of conscious perception in temporal judgements.

**Grant:** none

## 

**Keywords:** time perception, psychophysics

## Audiovisual Synchrony Perception for Music and Body Movement

**Tsukasa Uehara, Akira Takehana and Yutaka Sakaguchi**

Graduate School of Information Science and Engineering, University of Electro-Communications

## 

Synchronization between music and body motion is an essential issue affecting the artistic impression of human body performance. However, it has not been well elucidated how people perceive their synchrony. To answer this question, we examined the characteristics of synchrony perception in observing the performance of Japanese Radio Calisthenics (i.e., body exercise to music). We performed psychophysical experiments using the video clips of exercise performance presented with different onset asynchronies between visual and auditory streams. Participants were asked to judge the synchrony of visual and auditory stimuli. Synchrony was perceived when the timing of endpoints or lowest points of the hand/foot motion agreed with the music beats, which implies that these feature points serve as the visual reference for synchrony judgement. On the other hand, interestingly, the participants were able to judge the synchrony even when these feature points were invisible, suggesting that some prediction mechanism contributes to the synchrony perception. We would like to discuss the role of the above feature points in synchrony perception, from the viewpoints of motor prediction and “common-coding of action and perception.”

**Grant:** none

## 

**Keywords:** Audiovisual synchrony perception, Human body movement, Psychophysics

## Unconscious Non-Target Stimuli Can Compress the Perceived Duration of Visual Stimuli

**Riku Asaoka**

Kanazawa University

## 

A visual stimulus is perceived as shorter when a non-target stimuli is presented immediately before and after the visual target than when the visual target appears alone. The present study examined whether the time compression effect (TCE) can occur even when a participant is unaware of the presentation of the non-target stimuli. In order to manipulate the visibility of the non-target stimulus, we manipulated the luminance contrast between the target and non-target stimuli. We assumed that the participants could not perceive the non-target stimuli when the luminance contrast was low while they can could when the contrast was high. A white circle was presented as the target stimulus while a light gray (low contrast), dark gray (medium contrast) or black circle (high contrast) was presented before and after the target as non-target stimuli. The participants were asked to reproduce the perceived duration of the target while ignoring the non-target stimuli. The results showed that the perceived duration of the target was shorter when the non-target stimuli were presented than when they were not presented (control), suggesting that TCE occurred when the participants could not perceive the non-target stimuli. We can conclude that unconscious non-target stimuli can affect time perception.

**Grant:** JSPS KAKENHI Grant Number JP50825057

## 

**Keywords:** time perception, luminance contrast

## Effects of Exogenous and Endogenous Attention on Duration Perception

**Ryosuke Katsumata and Makoto Ichikawa**

Chiba university

## 

We investigated how each of exogenous and endogenous attention affects perceived duration for a visual stimulus. By combining target search task with duration judgment task, we examined the relationship between reaction time (RT) in target search task and duration perception. In target search task, spatial attention was controlled by pre-cueing method; exogenous attention was directed 100 ms before target onset by luminance increase while endogenous attention was directed 400 ms before target onset by orientation of a square space holder. In each trial, participants responded to the target stimulus onset as soon as possible, and then, judged whether target stimulus duration was longer than a pre-learned standard duration. We found a significant correlation between the RT and perceived duration only for exogenous attention, and that perceived duration in the valid condition was longer than that in the invalid condition only for exogenous attention although cost, benefit, and difference in RT between the invalid and valid conditions were significant for both exogenous and endogenous attention. These results suggested that perceived duration for a visual stimulus would be affected more directly by exogenous attention than by endogenous attention. The bases of effects of visual attention on perceived duration would be discussed.

**Grant:** none

## 

**Keywords:** cognitive psychology, time perception, spatial attention, pre-cueing method

## Perceived (In)Congruency of Audiovisual Stimuli Using Gabor Patches

**Natalia Postnova and Gerard B. Remijn**

Human Science Department, Kyushu University

## 

In numerous audiovisual studies Gabor patches have often been combined with various auditory signals, mostly simple modulated tones. These combinations were used to create “congruent” or “incongruent” audiovisual stimuli, based on correlations between manipulated spatial and temporal frequencies. The congruency factor is highly important in multisensory integration research, however, little systematic empirical studies describing the differences in perceptual congruency of Gabor patches and auditory tones have been performed. The nature of Gabor patches suggests that similar stimuli in the auditory domain should be modulated noises or pure tones, where modulating frequency corresponds either to spatial frequency of a Gabor patch, its phase-changing rate or its flickering rate (in case of a non-static patch). Here we conducted a congruency experiment where moving and flickering Gabor patches of various spatial frequencies were presented to participants accompanied by amplitude-modulated or frequency-modulated tones. Participants rated the perceived congruency of each stimulus on a scale from 1 to 7, resulting in congruency curves for various audiovisual stimulus parameters. The data could be used as a reference when designing experiments and conducting studies in audio-visual perception.

**Grant:** none

## 

**Keywords:** Audio-visual perception, Psychophysics, Perceptual psychology, Multisensoryresearch

## The Intentional Binding of Auditory and Visual Action Effects

**Takumi Tanaka and Hideaki Kawabata**

Keio University

## 

Intentional binding is a subjective compression of the temporal interval between a voluntary action and its external sensory consequence, which is composed of two illusions in time perception called “action shift” and “outcome shift” (Haggard, Clark, & Kalogeras, 2002). Given the sensorimotor aspects of intentional binding, we could not ignore the effect of different sensory modalities, such as temporal resolution. Moreover, recent studies revealed that such compression could occur in the space perception as well as temporal perception. Although several studies have used auditory stimuli while others used visual or somatic cues, the effects of outcome modalities have not been sufficiently investigated (Hughes, Desantis, & Waszak, 2013). Therefore, in two experiments with different procedures to measure intentional binding, we directly compared the binding effects between auditory and visual effects. Our results showed that binding effects for auditory and visual could have different temporal characteristics, indicating the independent bases of these phenomena.

**Grant:** none

## 

**Keywords:** time perception, psychophysics

## Normal Visuo-Auditory and Visuo-Tactile Processing in High Functioning Adults with Autism Spectrum Disorder

**Iris H. E. Loh^1^, Takashi Obana^2,3^, Tze Jui Goh^4^, Elysia J. W. Poh^2^, Christopher L. Asplund^1,2,3,5,6^**

^1^Department of Psychology, Faculty of Arts and Social Sciences, National University of Singapore

^2^Division of Social Sciences, Yale-NUS College

^3^Singapore Institute for Neurotechnology (SINAPSE), National University of Singapore

^4^Institute of Mental Health, National Health Group

^5^Clinical Imaging Research Centre, Yong Loo Lin School of Medicine

^6^Centre for Cognitive Neuroscience, Duke-NUS Medical School

## 

### PURPOSE

Individuals with autism spectrum disorder (ASD) have been found to have anomalous or reduced multisensory processing as compared to typically-developing (TD) controls. Such differences may account for the social and linguistic impairments in ASD. Here we test whether visuo-tactile processing is also different in those with ASD.

**Methods:** High-functioning adults who had been previously diagnosed with ASD (n = 23) and matched TD controls completed three temporal order judgement (TOJ) tasks. Participants were presented with pairs of simple stimuli (flashes, beeps, or vibrations) with various relative temporal offsets and reported which stimulus appeared first. Different pairings (visuo-visual, visuo-auditory, or visuo-tactile) were presented in separate blocks of trials.

**Results:** Psychometric functions were fit to the data for parameter estimation. Temporal precision, temporal bias towards a given modality, and reaction times were comparable across groups (TD, ASD) for each of the TOJ tasks. Visuo-auditory and visuo-tactile judgments had similar precision, and both were less precise than visuo-visual judgments. No significant temporal biases were observed.

**Conclusions:** Multisensory processing for some individuals with ASD appears to be in the normal range, despite differences on other measures. Such individuals may have had normal multisensory processing throughout development or caught up to TD individuals by adulthood.

**Grant:** Yale-NUS College Start up grant

## 

**Keywords:** multisensory, temporal perception, autism spectrum disorder

## Visual and Cross-Modal Association Skills Predict Music Sight-Reading

**Pu Fan^1^, Yetta Kwai-Ling Wong^2^ and Alan Chun-Nang Wong^1^**

^1^Perception and Experience Lab, Department of Psychology, the Chinese University of Hong Kong

^2^Learning and Perception Lab, Department of Educational Psychology, Faculty of Education, the Chinese University of Hong Kong

## 

Can visual skills explain variations in music sight-reading (SR) performance? This study delineated a hierarchical cognitive structure behind SR with an emphasis on visual skills and visual-to-auditory and visual-to-motoric transformation abilities. Music sight-reading refers to playing a piece of music at sight fluently and accurately without practice. The task is extremely complex, involving a series of perceptual, motoric, and cognitive processes under strict time constraints. Although previous studies proposed various component skills as building blocks of SR ability, this study is among the first to include visual skills and visual-related transformation skills as component predictors. In a multiple regression model, performance in seven perceptual-cognitive tasks explained over 50% of variance in SR performance of 143 pianists. The most important predictors were the visual perceptual fluency for sequential matching of musical notes, the visuoauditory association ability, and the visuomotor association ability. Visual perceptual fluency for numbers, trilling speed, executive functions, or working memory capacity did not significantly predict SR performance. Theoretically, this study speaks to the contribution of basic visual skills in complex domains of expertise, such as skilled reading. Practically, the model sheds light on the utilization of perceptual learning paradigms in SR training and complex skill development generally.

**Grant:** none

## 

**Keywords:** visual perceptual fluency, visuoauditory association, visuomotor association, individual differences

## Word Information Influences Unconscious Processing of Single Chinese Character under Interocular Suppression

**Jiane Bai^1,2,3^, Jinfu Shi^1,2,3^, Yiming Yang^1,2,3,4^**

^1^School of Linguistic Sciences and Arts, Jiangsu Normal University

^2^Jiangsu Collaborative Innovation Center for Language Competence

^3^Jiangsu Key Laboratory of Language and Cognitive Neuroscience

^4^Institute of Linguistic Sciences, Jiangsu Normal University

## 

Word superiority effects indicated that word level information plays an important role on single character recognition. Although single Chinese character often has meaning by itself, two-character words are very common in Chinese reading matrials. In this study, we examined whether word information can affect the unconscious processing of single Chinese character under interocular suppression. In each trial, a visible prime Chinese character was presented to subject’s two eyes for 2 s,then a high-contrast dynamic noise pattern was presented to one eye meanwhile a target Chinese character was presented to the other eye. For half trials, prime and target characters can combine into a two-character word and for the other half trials, prime and target were unrelated. We measured how long it took for the target characters to overcome suppression induced by the dynamic noise patterns. We found that compared to unrelated condition, when the prime and target can be combined into a word, it took less time for the target character to break from suppression. This result indicated that word information can influence unconscious processing of single Chinese character during interocular suppression.

**Grant:** Supported by National Natural Science Foundation of China(31400866,31671170),Natural Science Foundation of Universities in Jiangsu Province (14KJB180006), Jiangsu Provincial Foundation for Philosophy and Social Sciences (12YYC015)

## 

**Keywords:** unconscious perception, Chinese Character, Interocular suppression, behavioral experiment

## The Effects of Fonts on the Chinese Reading Performance in Adults with Low Vision

**Li-Ting Tsai^1^, Chiun-Ho Hou^2^, Yi-Ling Peng^3^, Kuan-Chun Liu^4^, Kaun-Jen Chen^2^ and Chia-Yuan Chan^2^**

^1^School of Occupational Therapy, College of Medicine, National Taiwan University

^2^Department of Ophthalmology, Chang Gung Memorial Hospital

^3^Department of Endocrine and Metabolism, Zhong-Xiao branch, Taipei City Hospital

^4^Shi-Pai Shi-He Rehabilitation Clinic

## 

It is well accepted that font affects text readability, especially for patients with low vision. We investigated the reading performance using the traditional Chinese reading charts in adults with low vision. Four types of character fonts (SimHei, Microsoft JhengHei, Jingxihei, and Biauki) were used. Participants were adults (n = 27, age 41–84) with best-corrected visual acuity (BCVA), not more than 0.5 decimal unit. We also performed stratified analysis by acuity level (VA ≦ 0.5 and VA≦0.3) to investigate the effects between fonts and acuities. Eye diseases were the causes of low vision. Three indexes, reading acuity (RA), critical print size (CPS), and maximum reading speed (MRS), were used to estimate reading performance. The viewing distance was 40 cm, and the illuminance was kept around 680 to 720 lux. The RA of all participants was higher across four types of fonts than the BCVA (e.g., RA of SimHei = 0.89 logMAR, BCVA = 0.48 logMAR). Significant differences were showed between the RA of SimHei and Microsoft JhengHei fonts and CPS of SimHei and Biauki fonts. For the sub-group (VA≦0.3), there was a significant difference between the MRS of SimHei and Biauki fonts (n = 11). Further application is discussed.

**Grant:** none

## 

**Keywords:** low vision

## Associations Between Grapheme and Gender in Japanese People

**Yan Zhang, Na Chen and Katsumi Watanabe**

Waseda University

## 

Some synesthesia was involved with cognitive constructs, such as graphemes associated with personalities or genders (also known as ordinal linguistic personification, OLP). However, little has been known with the grapheme-personality associations in a normal population. In the present study, we investigated grapheme personification with gender using a questionnaire survey in Japanese non-synesthesia people. 214 participants (174 males, Mage = 18.9, SD = 0.55) were asked to make a gender judgment to each grapheme (including 48 Hiragana and Katakana, 10 numbers in Arabic and Japanese Kanji form, and 26 English alphabets in uppercase and lowercase forms). Those results showed that some graphemes were significantly associated with specific genders, for example, Katakana and Kanji numbers are considered more as male gender. Besides, the gender judgments between hiragana and its counterpart katakana alphabets, Arabic and Its kanji members, uppercase and lowercase English alphabets showed strong consistence. Those results suggested that there might exist some nonrandom grapheme-gender associations in non-synesthesia people, and the sound of graphemes might contribute more to those associations.

**Grant:** none

## 

**Keywords:** grapheme, gender, Japanese

## Developmental Dyslexia: A Deficit in Magnocellular-Parvocelluar Co-Activation, Not Simply in Pure Magnocellular Activation

**Ambra Ciavarelli^1^, Giulio Contemori^1,2^, Luca Battaglini^1,2^, Clara Casco^1,2^ and Michele Barollo^2^**

^1^Department of General psychology, University of Padua

^2^NeuroVis.U.S. laboratory, University of Padua

## 

The magnocellular deficit theory of dyslexia predicts selective impairment in contrast detection of stimuli involving pure magnocellular response (for example, 0.5 c/deg, 30 Hz, low contrast). A recent alternative hypothesis posits that dyslexia may be associated with a reduction of the facilitation expected in normal readers with stimuli relying on low-level magno-parvo co-activation, with respect to stimuli eliciting pure magno activation. According to this hypothesis, any advantage in contrast sensitivity, produced both by decreasing stimuli temporal frequency (from 30 to 10 Hz, Experiment 1) or using static stimuli of increasing spatial frequency (from 0.5 to 4 c/deg, Experiment 2), would be ascribed to the coexisting responses of both systems. Differently from controls, dyslexic individuals, while showing no deficit in the unmixed magnocellular response, showed also no advantage when the relative weight between magnocellular and parvocellular inputs, was thrown off balance in favor of the latter. Our results point out that in dyslexia, the relative contribution of these two systems to visual processing is perturbed and this may have a detrimental consequence in word processing, both within the parafovea and the fovea, during fixation. Collectively, these data may give a contribution to the advancement of diagnostic and training protocols for developmental dyslexia.

**Grant:** none

## 

**Keywords:** Developmental dyslexia, magnocellular, parvocellular, contrast sensitivity

## Effect of Microsaccades on Global Processing of Shape

**Ken Wei Sheng Tan, Elizabeth M. Haris, Neil W. Roach and Paul V. McGraw**

University of Nottingham

## 

The sensitivity of the human visual system to subtle changes in shape are often attributed to the existence of global pooling mechanisms, which integrate local form information across space. While global pooling is typically demonstrated under conditions of steady fixation, other work suggests that prolonged fixation leads to a collapse of global structure. Here we ask whether small ocular movements during periods of fixation affect global processing of radial frequency (RF) patterns—closed contours created by sinusoidally modulating the radius of a circle. Observers were asked to indicate which of two peripheral patterns was deformed from circular, while fixational eye movements were recorded binocularly at 500 Hz. Global processing was assessed as the rapid fall in discrimination thresholds as cycles of modulation increased, whereas changes in mean threshold was used as an index of overall performance. Microsaccades were detected using a velocity-based algorithm, allowing trials to be sorted according to the relative timing of stimulus and microsaccade onset. Results indicate that microsaccades have a generally detrimental effect on task performance, consistent with previous demonstrations of saccadic suppression. In contrast, the presence of global processing was unaffected, suggesting its relative robustness to small eye-movements.

**Grant:** none

## 

**Keywords:** Shape processing, Psychophysics, Fixational eye-movement

## Aspect-Ratio Discrimination Thresholds for Objects Defined by Short-Range Motion and Luminance Contrast

**Andrew Lachlan Stark and Philip MacVicar Grove**

School of Psychology, The University of Queensland, Australia

## 

Existing studies on spatial form defined by short-range motion (SRM) predominantly focus on the conditions necessary to yield the perception of a discrete object. However, the spatial acuity of our perception of such objects remains unknown. We measured aspect-ratio discrimination thresholds for objects made visible through a single iteration of SRM and compared them with thresholds for luminance defined objects. Participants viewed random-dot patterns where a central rectangular section was made visible through SRM or luminance contrast. In the SRM condition, two random-dot patterns were presented in rapid succession. The surrounding dots were identical for each presentation. However, the central section was shifted horizontally, making it briefly visible through SRM. In the luminance condition, mean luminance of the surrounding dots was increased to make the central section discernible. Target aspect-ratios ranged from 0.88 to 1.12. Participants indicated which dimension was greater, height or width. Thresholds were determined using the method of constant stimuli. For equated stimulus display times, no significant differences in aspect-ratio discrimination thresholds were found between the SRM and luminance conditions. We conclude that aspect-ratio discrimination thresholds for SRM are similar to those of luminance, a first-order visual perception pathway.

**Grant:** None

## 

**Keywords:** Spatial vision, Short-range motion, Psychophysics

## Neural Correlates of Semantic Priming under Visual Crowding: An MEG Study

**Sung-En Chien^1^, Yung-Hao Yang^2^, Shohei Teramoto^3^, Yumie Ono^3^ and Su-Ling Yeh^1^**

^1^Department of Psychology, National Taiwan University

^2^Human Information Science Laboratory, NTT Communication Science Laboratories, Nippon Telegraph and Telephone Corporation

^3^Meiji University

## 

Visual crowding refers to impaired object recognition when a peripheral visual target is closely surrounded by flankers. Yeh et al. (2012) found that semantic priming survived visual crowding: Faster response times in the semantically related (vs. unrelated) condition with unrecognizable (crowded) prime words. We used magnetoencephalography (MEG) to investigate the neuronal mechanisms of such semantic priming effect when an isolated or a crowded prime preceded an isolated target, and the prime and target was either semantically related or not. Participants were asked to judge whether the target was a word in a go/no-go lexical decision task. Results showed theta-band (4–7 Hz) activation when contrasting between unrelated and related primes in the left inferior frontal gyrus (left-IFG) during 300–500 ms after the onset of the target for isolated primes, but during 450–750 ms for crowded primes. Thus, the semantic priming effect with crowded primes was delayed compared to isolated primes. The phase locking values analysis showed difference in synchronization of theta-band between left- and right-IFG regardless of whether the primes were isolated or crowded. These findings provide neural evidence for semantic priming under visual crowding and additionally indicate a cross-hemisphere interaction in semantic relatedness for crowded and isolated primes.

**Grant:** none

## 

**Keywords:** visual crowding, Magnetoencephalography, semantic processing

## Influence on Spatial Resolution by Dual Task and Illuminance Level

**Toru Sugiura^1^ and Hiroyuki Shinoda^2^**

^1^Graduate School of Information Science and Engineering, Ritsumeikan University

^2^College of Information Science and Engineering, Ritsumeikan University

## 

Eyewitness testimony in a crime scene is treated as an important evidence in a trial. Accuracy of eyewitness testimony has been studied from various perspectives. Most of the studies took account of the influence of environments of sighting. For instance, color response was shown to be inconsistent because color recognition was affected by illuminance, stimulus size and observation duration. In actual sighting situations, however, witnesses happen to see an accident or an event in the midst of execution of other irrelevant tasks such as driving. In other words, witnesses may not completely concentrated on sighting. In the present study, experiments were conducted to examine how spatial resolution changes due to dual task. A participant discriminated the orientation of a Landolt ring presented at random times while tracking a moving target using a mouse. The illuminance of an experimental booth was set at various levels from photopic to scotopic visions. The luminance of the Landolt ring, the moving target, the mouse cursor and the background were modulated on a display according to the illuminance level. The result showed a degradation in the orientation discrimination due to dual task. The performance degradation was larger at mesopic illuminance levels than other illuminance levels.

**Grant:** none

## 

**Keywords:** eyewitness testimony, psychophysics, visual acuity

## A Novel Kind of Suppression in Human Visual Cortex: Orientation-specific Surround Suppression in Polar Space

**Juhyoung Ryu and Sang-Hun Lee**

Department of Brain and Cognitive Sciences, Seoul National University

## 

Active vision—adaptive sampling of visual information for an impending task—creates intricate regularities in image statistics, which can be utilized as prior knowledges for efficient visual processing. We note one such prior in active vision of visual search for objects: circular arrays of concentrically oriented contours become prevalent as a meaningful object (e.g., human face) enters the fovea. As one way of exploiting this prior, we hypothesize that human visual system modulates the excitation-vs-inhibition (EI) balance between receptive-field center and surround in a manner specific to the orientation defined in polar space, such that concentric contours induce salient population neural representations. We (i) acquired BOLD responses from V1 to ‘concentrically’ versus ‘radially’ oriented bars in ring- or wedge- shape apertures that drifted over space along the polar axis, (ii) developed several V1 models that differ in the way of implementing the orientation-specific EI balance, and (iii) examined which model best explains the observed BOLD time series. The best model supported our hypothesis by exhibiting pronounced surround suppression for concentric unit along the radial axis, implying that human visual system promotes salient neural representations of visual objects at its earliest stage by augmenting its suppression on distractive features in the surround.

**Grant:** The Brain Research Programs and Basic Research Laboratory Program through the National Research Foundation of Korea (NRF- 2015M3C7A1031969; NRF-2017M3C7A1047860; NRF-2018R1A4A1025891)

## 

**Keywords:** Object vision/fMRI

## Correlations among Geometrical Illusions Dissociate Length Illusions from Size Illusions

**Takahiro Suzuki^1^, Daisuke Matsuyoshi^1^, Katsumi Watanabe^1,2^**

^1^Faculty of Science and Engineering, Waseda University

^2^Art & Design, UNSW Sydney

## 

The majority of studies of geometrical illusions have individually investigated mechanisms underlying each illusion (but see Axelrod et al., 2017; Grzeczkowski et al., 2017). It would be important to understand common factors in various kinds of visual illusions rather than just focusing on a single illusion. The present study examined the correlation between five visual illusions (Muller-Lyer, Ebbinghaus, Delboeuf, vertical-horizontal, and contrast illusions) by using correlation analysis and principal component analysis (PCA). Seventy nine Japanese participants reported the magnitudes of the 5 illusions were measured by using the adjustment method. We found a significant positive correlation between Ebbinghaus and Delboeuf illusions. The PCA showed that the first two principal components (PC) accounted for 57% of the variance. The Delboeuf and Ebbinghaus illusions have positive loadings on the first PC, whereas the Muller–Lyer and vertical-horizontal illusions have negative loadings on the second PC. The first and second PCs can be interpreted as being related to size perception and length perception, respectively. Although it should also be noted that the remaining 43% of the variance remains to be explained, these results suggest that the two illusions of size share common underlying mechanisms and the two illusion of length share other common mechanisms.

**Grant:** none

## 

**Keywords:** visual illusions, geometrical illusions, correlations, psychophysics

## Experiments for Measurement of Peripheral Contrast Sensitivity Function

**Shiori Ito^1^, Suguru Saito^2^ and Keiji Uchikawa^3^**

^1^Tokyo Institute of Technology

^2^Tokyo Institute of Technology

^3^Kanagawa Institute of Technology

## 

For a wide view display such as HMD, considering peripheral visual perception characteristics is useful for accelerating rendering speed for computer graphics images, designing UI and reducing the image data transfer from a host device to a display device. Contrast sensitivity function, CSF, which is one of the visual perception characteristics, is the perceivable threshold for the contrast of visual stimulus. We present our experimental environment and method to measure peripheral color CSFs that have not been measured in previous studies. The measurements are performed in the range from the fovea to peripheral up to 90 degrees in steps of 7 degrees. The stimuli are sinusoidal spatially varying Gaussian enveloped gratings, which are achromatic, red-green and blue-yellow chromatic, presented upon a gray background. The diameter of the stimulus is always constant at 10 degrees. All experiments are performed photopic and monocularly. Staircase-driven two-alternative forced-choice paradigm to measure thresholds is adopted. The distortion of the distribution of the accuracy rate is reduced using the bootstrap method, fitted to a sigmoid curve, and a value at which the 75% accuracy rate is used as a threshold.

**Grant:** none

## 

**Keywords:** contrast sensitivity function, peripheral vision, wide view image application, spatial frequency

## The Visual Cognition of Inverted Navon Figure: A Study by Psychophysical Method

**Takashi Murakami**

Ritsumeikan University

## 

We investigated the influence of inverted features of Navon figures of different sizes. According as the visual angle of Navon figure increases, the local feature of it is more focused. Using the task that the participants was asked which local or global feature in each figure was perceived more clearly, we could determine the threshold size at which local feature was more focused than the global one. We used a factorial within-subjects design of the orientation of global feature (upright/inverted) and the orientation of local feature (upright/inverted), calculated the threshold size in each conditions and compared with them.

**Grant:** none

## 

**Keywords:** visual cognition, navon figure, psychophysics

## Dissecting the Origins of Perceptual Biases during Delayed Orientation Estimation

**Dong-gyu Yoo, Sungje Kim, Jungwon Ryu and Sang-Hun Lee**

Department of Brain and Cognitive Sciences, Seoul National University

## 

Visual events occur stochastically in nature. The imprecision in our sensory apparatus adds further noises to sensory measurements of those visual events. Thus, a rational agent is expected to resort to any prior knowledge about world states available at given moments in space and time and utilize that knowledge to infer the world state that has engendered particular sensory measurements. Indeed, people are well known to exploit spatial and temporal coincidence statistics in the static environment (long-term prior) for visual perception. Recent work reports that human perception also reflects rather short-term prior knowledge about recent and nearby events. We reason that these long-term and short-term priors both contribute to visual perception. However, despite many separate lines of studies on these two types of priors respectively, concurrent effects of the long-term and short-term priors on visual perception have been rarely studied. To address this issue, we conducted a delayed orientation estimation experiment. We found that subjects’ target estimates were systematically biased as a function of ‘current target orientation’, ‘previous target orientation’, and ‘current non-target orientation’. These results confirm that both the long-term and short-term priors contribute to visual perception in a scene and require their concurrent effects to be investigated.

**Grant:** The Brain Research Programs and Basic Research Laboratory Program through the National Research Foundation of Korea (NRF- 2015M3C7A1031969; NRF-2017M3C7A1047860; NRF-2018R1A4A1025891)

## 

**Keywords:** Delayed Estimation, Perceptual Biases

## Color-Shape Associations in Kids and Parent-Kid Pairs

**Na Chen and Katsumi Watanabe**

Waseda University

## 

Neurotypical Japanese people systematically associate shapes with colors (e.g., circle-red, triangle-yellow, and square-blue), which might be explained by common semantic dimensions (e.g., warm/cold). However, the development of color-shape associations has been yet unknown. In the present study, we examined color-shape associations in Japanese kids (N = 60, 24 male, Mage = 8.4, SD = 1.8) and their parents (N = 60, 18 male, Mage = 42, SD = 6.4) using an online questionnaire survey during a lab event. Results showed that kids could systematically associate certain shapes with particular colors (e.g., circle-red, triangle-yellow, and square-blue/green), consistent with the pattern observed in their parents’ group (e.g., circle-red and triangle-yellow), and other adults in a previous study (e.g., circle-red, triangle-yellow, and square-blue/green; Chen et al., 2016). Further analysis on the agreement of those color-shape associations within each parent-kid pair showed that there was little agreement on the color choices for shapes. Those results suggested that kids at the age around eight might have establish some color-shape associations, consistent with adults, and the parenting experience might have little influence on those color-shape associations.

**Grant:** none

## 

**Keywords:** experimental psychology

## Visual Distractor Modulates Neural Representation of Objects’ Roughness Held in Visual Working Memory

**Munendo Fujimichi^1,2^, Hiroyuki Tsuda^1^, Hiroki Yamamoto^1^ and Jun Saiki^1^**

^1^Graduate School of Human and Environmental Studies, Kyoto University

^2^Japan Society for the Promotion of Science

## 

We previously examined where objects’ roughness is maintained in the human brain. In the prior study, the use of blank delay allowed participants to concentrate on holding objects’ roughness. However, the fact that we must process visual information while maintaining memory information in everyday life casts some doubts whether experiments with the blank delay reflect the nature of visual working memory for roughness. In the current study, we conducted an fMRI experiment using a delayed roughness discrimination task with visual distractor. We also conducted localizer runs to identify brain regions processing objects, color, faces, and scenes; and phase-encoded retinotopy measurements to define retinotopic visual areas. The memorized sample’s roughness was decoded from activity patterns during the delay in which a distractor was presented in an unpredictable fashion. We found that the ventral visual cortex could decode the roughness when the distractor was presented, while the secondary somatosensory cortex could decode it when no distractor was presented. Furthermore, similar to the secondary somatosensory cortex, intraparietal sulcus supports maintaining roughness only when there is no distractor in the current study. These results suggest that visual distractor affects neural representation of objects’ roughness held in visual working memory.

**Grant:** Japan Society for the Promotion of Science

## 

**Keywords:** visual working memory, fMRI, roughness

## Reverse Correlation AnalysIs of Visual Evoked Potentials for Natural Texture Statistics

**Taiki Orima and Isamu Motoyoshi**

Department of Life Sciences, the University of Tokyo

## 

Recent evidence shows that primate early visual cortex computes the statistical structure of natural images. To understand the temporal dynamics of neural encoding of such statistical information, we applied reverse correlation analysis to visual evoked potentials (VEPs) for image statistics present in natural textures. We recorded EEG signals from 14 observers viewing 166 natural texture images presented foveally in random order for 500 ms with a 750 ms blank (24 repetition each). We computed the correlation coefficients between occipital VEP amplitudes (O1+O2) and image statistics believed to be encoded in V1 and V2 (e.g., Freeman & Simoncelli, 2011). The analysis revealed that correlations with low-level moment statistics (SD and kurtosis) rose up at different latencies for lower (∼120 ms) and higher (∼170 ms) spatial frequency bands. Correlations with higher-order statistics (cross-band energy correlations) were evident over latencies ranging from 150 to 300 ms. We found no correlation to image features at a specific spatial location (e.g., fovea). Similar patterns of results were obtained for textures synthesized with the Portilla-Simoncelli algorithm. These results indicate that early visual cortex encodes with systematically different temporal dynamics for different classes and spatial scales of image statistics.

**Grant:** Supported by Commissioned Research of NICT (1940101), and by JSPS KAKENHI JP15H05916 and JP18H04935.

## 

**Keywords:** natural image statistics, spatial vision

## Effects of Surface Optics on the Perceived Shape of Elliptical Objects

**Masakazu Ohara^1^, Juno Kim^2^, Kowa Koida^1,3^**

^1^Department of Computer Science and Engineering, Toyohashi University of Technology

^2^School of Optometry and Vision Science, University of New South Wales

^3^Electronics-Inspired Interdisciplinary Research Institute, Toyohashi University of Technology

## 

Pictorial cues such as shading are the basis of our three-dimensional (3D) shape perception. However, natural objects have different forms of surface optics (e.g., specular and diffuse reflectance, as well as refractivity and translucency) that may potentially complicate the inference of 3D shape. How do we untangle complex image structure to experience the shape and surface quality of 3D objects? We examined the effects of surface optics on shape perception of virtual objects rendered with varying surface optics (matte, specular, refractive or combined specular and refractive). We also varied the surface’s relief (smooth or bumpy) and convexity along the viewing axis. Rendering was performed in the two natural environments. Observers performed a shape matching task to estimate the convexity of these objects when presented either statically or dynamically oscillating horizontally in movie sequences. We found that purely refractive objects without specular reflection were perceived flatter than specular and matte objects for all convexity levels. These effects were consistently observed for objects of different sizes and even for statically presented images. Image pixel and sub-band analysis revealed that narrow band (not lower band) power could account for perceived shape.

**Grant:** Leading Graduate School Program R03 of MEXT

## 

**Keywords:** material perception, depth perception, psychophysics, motion

## Effects of the Manipulation of Neural Activities in the Gloss Selective Region on the Gloss Discrimination Behavior in The Macaque Monkey

**Akiko Nishio^1^, Mika Baba^2^, Takeaki Shimokawa^3^ and Hidehiko Komatsu^2^**

^1^National Institute for Physiological Sciences

^2^Brain Science Institute, Tamagawa University

Advanced Telecommunications Research Institute International (ATR)

## 

Previously, we have reported that there exist neurons that selectively responded to specific gloss (gloss selective neuron) and that these gloss selective neurons were concentrated in a restricted region extending 2–3 mm in the lower bank of the superior temporal sulcus in the central part of the inferior temporal (IT) cortex of the monkey (gloss selective region) (Nishio et al., 2012). In the present study, to examine the causal relationships between the activities of the gloss selective neurons and gloss perception, we manipulated neural activities by applying electrical microstimulation or injecting a small amount of muscimol (GABA-A agonist) within the gloss selective region or its surrounding region while monkeys were performing a gloss discrimination task. We found that microstimulation induced bias toward higher gloss judgment at some sites within and at the vicinity of the gloss selective region. For muscimol injection, the gloss discrimination performance was degraded after the first injection of muscimol into this region. These results suggest that gloss selective neurons in IT cortex have causal relationships with gloss perception.

**Grant:** KAKENHI (16K21587), KAKENHI (15H05916)

**Keywords:** gloss perception, muscimol injection, microstimulation, macaque

## Effects of Optical Parameters on Perceptual Transparency

**Mika Baba^1^, Kei Kanari^1^, Kei Iwasaki^2^ and Hidehiko Komatsu^1^**

^1^Brain Science Institute, Tamagawa University

^2^Wakayama University

## 

Transparency or translucency are important characteristics for visually estimating material properties of objects. It is widely acknowledged that the way lights transmit and scatter inside an object affects the perceptual transparency, although only few studies have systematically examined the relationship between physical parameters of optical transmission/scattering and perceived transparency or translucency. Recently, it has been reported that parameters related to phase function of light scattering affect perceptual translucency (Gkioulekas et al., 2013). In the present study, we investigated how other parameters related to light transmission/scattering (absorption coefficient and scattering coefficient) affect the perceived transparency. We asked subjects to evaluate the degree of transparency of computer graphics images of objects having different combinations of extinction coefficient (sum of absorption and scattering coefficients) and albedo (ratio between absorption and scattering coefficients), generated by Mitsuba renderer. We found that perceived transparency mainly depended on extinction coefficient. However, albedo also had significant effect on perceived transparency and the effect depended on the complexity of object shapes.

**Grant:** KAKENHI 15H05916

## 

**Keywords:** psychophysics, perceptual transparency, shitsukan

## Perceived Glossiness Based on Low-Luminance Specular Components Can Be Increased by Enhancing Luminance Edge Contrasts

**Hiroaki Kiyokawa^1^, Tomonori Tashiro^1^, Yasuki Yamauchi^1^ and Takehiro Nagai^2^**

^1^Yamagata University

^2^Tokyo Institute of Technology

## 

It has been reported that our visual system utilizes low-luminance specular components for glossiness perception even on object surfaces without specular highlights (Fleming et al., 2003; Kim et al., 2012). Previously, we found that amounts of luminance edges are well correlated with perceived glossiness on some object images whose highlight dependency (HD) of glossiness perception was weak. Here, we tested whether manipulation of luminance edge contrasts affects perceived glossiness obtained from low-luminance specular components. We manipulated luminance edge contrasts using a Laplacian filter on six object images used in previous our experiment (three object images with high HDs and other three images with low HDs). Then, perceived glossiness was measured on these stimuli in a glossiness rating experiment. In the results, perceived glossiness was significantly enhanced by increasing the edge contrast on the low HD images, while it was not strongly enhanced by edge contrast manipulation on the high HD samples. These results suggest that our visual system also utilizes luminance edges for glossiness perception as a cue, though there should be some constraints in object image characteristics for luminance edges to act as a cue for glossiness perception.

**Grant:** none

## 

**Keywords:** Material perception, Glossiness perception, Psychophysics

## Perceptual Dynamics for Diffuse-Specular Spatial Congruence in Gloss Perception

**Ryotaro Maki^1^, Tomonori Tashiro^1^, Yasuki Yamauchi^1^ and Takehiro Nagai^2^**

^1^Yamagata University

^2^Tokyo Institute of Technology

## 

Spatial congruence between specular and diffuse reflection components is crucial for glossiness perception. Here, we investigated if modulation of glossiness perception due to the specular/diffuse congruence accompanied with recognition of the congruence by comparing perceptual dynamics of glossiness perception and congruence detection. The stimulus was a pair of computer-graphics images, which were briefly presented at the left and right on an LCD monitor. In one of the images, specular and diffuse components were spatially congruent, while in the other, the specular components were rotated relative to the diffuse components. Participants performed two tasks: glossiness task and congruence task. In glossiness task, they judged which image was glossier. In congruence task, they judged which image had spatially congruent specular/ diffuse components. For these two tasks, the effects of stimulus duration on the accuracy were investigated. The results showed that the accuracy was better for the glossiness task than for the congruence task, and that the accuracy differences became larger as the stimulus duration got shorter. These results suggest that the spatial incongruence of the specular/diffuse components affects perceived glossiness without recognition of it, that is, the incongruence was automatically detected for glossiness perception in the visual system.

**Grant:** none

## 

**Keywords:** material perception, perceptual dynamics, psychophysics

## Relationship Between Image Statistics and Psychophysical Dynamics of Perception of Various Surface Qualities

**Ryoto Seno^1^, Tomonori Tashiro^1^, Yasuki Yamauchi^1^ and Takehiro Nagai^2^**

^1^Yamagata University

^2^Tokyo Institute of Technology

## 

Humans can perceive different kinds of surface qualities on object surfaces at a glance. In this study, we examined how lower-order image statistics relate to surface quality perception on briefly presented object images. The stimulus was an image pair selected from 131 object images, which was briefly (for 33.4–150.2 ms) presented to observers. They responded which of two images gave stronger impression regarding either glossiness, transparency, warmness, or heaviness. In addition, we optimized a linear support vector machine (SVM) based on lower-order image statistics to predict observer’s responses. The SVM performance was used as an index, which describes relationship between image statistics and human perception. In the results, the difference in perception accuracies between the surface qualities enlarged as the stimulus durations got shorter. In addition, the surface quality that can be perceived stably even for short presentation duration, such as glossiness and transparency, had closer relationship with lower-order image statistics than the other qualities. These results suggest that our instantaneous surface quality perception may depend on lower-order image features.

**Grant:** none

## 

**Keywords:** material perception, perceptial dynamics, psychophysics

## Estimation of Difference in Unified Material-Appearance Yielded by Color and Gloss of a Surface

**Kenji Kagitani^1^, Takehiro Nagai^2^, Tomohisa Matsumoto^3^ and Keiji Uchikawa^3^**

^1^Ricoh Co., Ltd

^2^Tokyo Institute of Technology

^3^Kanagawa Institute of Technology

## 

It has been studied how a material appearance of a surface is quantified using image feature values when it varies in a single attribute, such as gloss, color or texture. However, in our real word, surfaces vary not in a single attribute but in several attributes, which are unified into a material appearance. In this study, we aimed at quantifying this “unified material-appearance”. We measured differences in material-appearance of surfaces, using CG-faces and plastic corrugated sheets as stimuli. We used colors and glosses in several steps as material attributes, and made all combinations of these colors and glosses. In experiments two stimuli of different combinations were presented side by side on a display. Observers estimated the magnitude of difference in unified material-appearance of the stimuli with 2 or 4 scales. Our prediction model used up-to-third order image statistics (mean, standard deviation and skewness) in a color space, and showed high correlations with the estimations. It consisted of the same explanatory variables with very close standardized partial regression coefficients for both type of stimuli. These results indicate that our model is not restricted to specific objects but could be possibly used for general objects.

**Grant:** This work was supported by JSPS KAKENHI Grant Number JP17H01809.

## 

**Keywords:** color, material perception

## Influence of the HDR Environment on Shitsukan Perception

**Gaku Watanabe^1,2^, Ichiro Kuriki^1,2^, Yasuhiro Hatori^1,2^, Chia-huei Tseng^1,2^, Satoshi Shioiri^1,2^**

^1^Research Institute of Electrical Communication, Tohoku University

^2^Graduate School of Information Sciences, Tohoku University

## 

High dynamic range (HDR) display is a technology to present high and low luminance (over 1 : 10,000 in min-to-max ratio) simultaneously. Recently, HDR displays like organic electroluminescent display (OLED) becomes more accessible, and its luminance contrast is significantly higher than standard dynamic range (SDR) displays like liquid crystal displays (LCDs). If human visual sensitivity changed with the dynamic range, apparent lightness contrast would be different between SDR and HDR environments, which could affect texture perception of material surfaces. However, this was not intensively studied. We first examined the lightness perception of various luminance patches under various surround conditions of luminance dynamic range, by using an OLED display in an otherwise dark room. We found that the lightness perception is significantly affected in darker part by the luminance dynamic range of surround. Next, we presented material sample images (fabric, wood, stone, and leather) on the SDR (1 : 1,000) and HDR (1 : 100,000) surrounds, and asked participants to choose one in which the clues for material perception are more clearly recognizable. The choice ratio for samples in the HDR background was significantly higher than the chance level. This is probably because the perceived contrast, as our lightness matching experiment suggested, was higher in HDR environment.

**Grant:** none

## 

**Keywords:** high dynamic range, lightness perception, material perception, psychophysics

## Metal- and Gloss-Perceptions and Object Impression Measured by Semantic Differential Method on Gold-, Silver- and Copper-Gradation-Colored CG Objects

**Keizo Shinomori and Takumi Inoue**

School of Information, Kochi University of Technology

## 

Recently, various studies on occurrence conditions of the metal- and gloss-perceptions have been conducted in views of material perception. However, the relationship between the metal- and gloss-perceptions and object impression which can be measured by semantic differential method has not been investigated yet. In this research, the magnitude of these perceptions and the object impression were measured on CG objects when parameters of surface reflection properties were changed. Colors of objects were gold, silver and copper and additionally, we created unrealized object images that had gradational color changes between two colors of them. Both of metal- and gloss-perception scores increased in objects of larger specular reflection value and lower surface roughness value. We employed the principal component (PC) analysis to scores of the semantic differential evaluation. Only the first PC score had the significant correlation (p<0.001) with scores of metal- and gloss-perceptions, while the second and fourth PC scores had significant relationship (p<0.05) with colors of objects. The usage of unrealized objects (in gradation color) did not show any special effect; the object impression tended to be determined by primary visual information from the object surface rather than the logical understanding or recognition of the unrealized objects.

**Grant:** JSPS KAKENHI Grant Number 18H03323

## 

**Keywords:** metal perception, gloss perception, object impression, semantic differential method

## Illusory Enhancement of Brightness Contrast of Sinusoidal Grating by a Salient Spot

**Soyogu Matsushita^1^, Sakiko Kikunaga^2^, Junya Aoyama^2^ and Tsuyoshi Nomura^2^**

^1^Osaka Shoin Women’s University

^2^Pias Group Central R&D Laboratory

## 

Perceived brightness is calculated from not only the target stimulus but also from various visual elements in the visual field. In this study, we report a novel phenomenon in which a salient spot illusory increases the perceived contrast of brightness of the adjacent area. The stimulus images were sinusoidal profiled grayscale gratings with/without a black patch. In the results, the dark area of the grating with a black patch was perceived as darker and the bright area was perceived as brighter than those without the patch. These results indicate that a salient spot in certain conditions enhances the apparent brightness contrast rather than masking it.

**Grant:** none

## 

**Keywords:** brightness, contrast, illusion

## Color Adaptation to Temporal Color Modulations along Complicated Loci in the Chromaticity-Luminance Plane

**Kana Kakuta^1^, Tomonori Tashiro^1^, Yasuki Yamauchi^1^ and Takehiro Nagai^2^**

^1^Yamagata University

^2^Tokyo Institute of Technology

## 

Color contrast adaptation to temporally color-modulated stimuli, in which chromaticity and luminance are one-dimensionally correlated, is well known. However, considering complex perceptual phenomena such as color constancy based on physical features of specular highlight colors, there is a possibility that color contrast adaptation also occurs to stimuli with more complicated relations between chromaticity and luminance. In this study, we examined if there are color mechanisms that adapt to temporal color modulations along complicated loci in the chromaticity-luminance plane. The adaptation stimulus was a uniformly colored circle. Its color temporally changed along the “〈“or ”〉” shaped locus in the chromaticity (L–M)-luminance plane. After the observer adapted to this stimulus, achromatic points were measured for several luminance levels. In the results, the achromatic point shifted toward the same direction as the adapted chromaticity at each luminance level; namely, after adapted to the “<” stimulus, the achromatic points also formed a “<” shape, and vice versa. In addition, these achromatic points were well predicted by the higher-order color representation model with multiple channels. These results suggest that the visual system can adapt to different chromaticity independently at low/high luminance levels.

**Grant:** none

## 

**Keywords:** contrast adaptation, color vision, psychophysics

## Sensitivity to Different Levels of Supra-Threshold Color Difference

**Yuken Ito^1^, Tomoharu Sato^2^, Tomonori Tashiro^1^, Yasuki Yamauchi^1^ and Takehiro Nagai^3^**

^1^Yamagata University

^2^Ichinoseki College

^3^Tokyo Institute of Technology

## 

Our previous study demonstrated that color discrimination and supra-threshold color difference perception exhibited different sensitivity transition patterns along stimulus chromaticity (Sato et al., 2016). In this study, we examined whether sensitivity properties of supra-threshold color difference perception change with the stimulus color difference. In the experiments, the stimuli consisted of three squares with different chromaticity on the LM axis. The left and center squares, and right and center squares were defined as pairs, respectively. There were several conditions about physical color differences between the pairs. The observers selected which pair exhibited larger perceptual color difference. The sensitivities for supra-threshold color differences on all the stimulus chromaticity were estimated with the Maximum Likelihood Difference Scaling (MLDS) method from the observer’s responses for each color difference condition. In the results, for the smallest color difference there was only a sensitivity peak on the L–M axis near the achromatic point, while for larger color differences several sensitivity peaks were clearly apparent. These results suggest that the color mechanisms involved in supra-threshold color difference perception may change with the color difference to be judged.

**Grant:** none

## 

**Keywords:** supra-threshold color differences, psychophysics, color vision

## Magnitude of Yellow-Blue Color Aftereffect Varied Depending on Luminance of Test Stimuli

**Yuta Watanabe^1^, Kowa Koida^1,2^**

^1^Department of Computer Science and Engineering, Toyohashi University of Technology

^2^Electronics-Inspired Interdisciplinary Research Institute, Toyohashi University of Technology

## 

Color and luminance are thought to be processed independently at early stages of visual processing, however the interaction between them are poorly understood. Adaptation technique has been widely used to reveal the coding strategies in the visual system. How does color aftereffect differ when the luminance of test stimuli changed? We measured the aftereffect by using five level of luminance of test stimuli. Adaptation stimuli were circular patches colored either blue-yellow direction or L–M cone direction on gray background. After adapting to color, observers were asked to null the appearance of color by modulating chromaticity of test stimuli. We found color aftereffect induced by blue-yellow stimuli enhanced when test stimulus was darker than adapting stimuli. In contrast, those luminance dependency of color aftereffects were not observed for L–M cone color adapter. These luminance dependencies were similarly observed across three types of test stimuli in which uniform screen, circular patches same to the adapter, and textured pattern. These data indicate that integration of color and luminance signals is different between two cardinal axes.

**Grant:** none

## 

**Keywords:** color vision, psychophysics

## Brightness Perception of Face in Different Type of Skin Color

**Suguru Tanaka and Yoko Mizokami**

Department of Imaging Sciences, Graduate School of Engineering, Chiba University

## 

Yoshikawa et al. (2012) showed that a face with reddish skin appeared brighter than that with yellowish skin, even though the average lightness of both faces was the same. In their experiment, the face and the skin color was the average of Japanese females, and the observers were Japanese. However, the characteristics of skin color depend on ethnicities, environments and other factors. In this study, we investigated whether brightness perception was similar in the skin color of different ethnicities. The average skin colors of Caucasian, Thai, and African were reproduced on a Japanese female face. We prepared reference face stimuli with five hue angles including yellower and redder than the original hue for each ethnicity. A matching stimulus with the average skin color changing only lightness was created for each ethnicity. Observers adjusted the brightness of the matching stimulus to match the brightness of the reference stimulus. We also tested stimuli with different lightness conditions to examine the influence of hue and lightness on the brightness perception. As a result, reddish skin tended to appear brighter and yellowish skin appeared darker in any skin color. However, this effect decreased as the lightness of the faces decreased.

**Grant:** JSPS KAKENHI JP18H04183

## 

**Keywords:** skin, face, brightness, color

## Influence of Skin Color to The Brightness Perception of Facial Skin—Comparison of Japanese, Thai and Chinese

**Yuanyuan He, Taiga Mikami, Suguru Tanaka and Yoko Mizokami**

Chiba University

## 

It was shown that reddish skin appeared brighter than yellowish skin when both had the same lightness (Yoshikawa et al., 2012). However, this effect was confirmed only for Japanese observers, and it is not clear how the brightness perception of facial skin influenced by the diversity of skin colors and observers. Here, we investigate the influence of skin color to the brightness perception of facial skin for Japanese and Thai and Chinese observers. We used a young Japanese female face as an original face. We prepared test faces with four skin color types that were the average skin colors of Japanese, Thai, Caucasian, and African. A test face image (one of five images with constant lightness and different hue angles) and a scale face image (skin color of each skin color types and different lightness levels) were presented side by side on a tablet display. Observers adjusted the brightness of a scale face to match that of a test face. As a result, Japanese showed a trend consistent with the previous study, whereas Thai showed an opposite trend and Chinese did not show the influence of hue, suggesting diversity in the brightness perception of facial skin.

**Grant:** JSPS KAKENHI JP16H01663 and JP18H04183

## 

**Keywords:** Color perception, Brightness perception, Facial skin, Psychophysics

## The Development of Handy Measurements for Visual Dynamic Range

**Satoshi Nakadomari^1,2,3^, Eiji Suzuki^4^, Hiroshi Horiguchi^3^, Takashi Fujikado^5^, Muneto Tatsumoto^6^, Takeo Fukuchi^7^ and Yasuo Kurimoto^2^**

^1^Next Vision Public-interest Co

^2^Kobe City Eye Hospital

^3^The Jikei University School of Medicine

^4^Tokai Optical Co., Ltd

^5^Osaka University

^6^Dokkyo Medical University

^7^Niigata University

## 

We developed handy measurements to estimate the abnormality of visual dynamic range (DR) of the Human eye. Here we defined DR as the range that a subject could promptly distinguish gray gradation. Twenty-three healthy controls and 31 photophobic patients with eye diseases were participated. A thick bar whose color was gradually changing spatially from white to black was displayed on a tablet PC. A subject tapped a border between grayish white/black and perfect white/black with three conditions of background brightness. In controls, a range of gray gradation was 77.7±9.7%, 76.8±10.0% and 77.7%±9.2% in dark, middle and bright condition, respectively. A gray center point shifted at 1.6±2.3% from bright to dark background in controls. On the other hand, in patients, the gray range was 67.0±19.5%, 64.5±20.0% and 65.0±20.3%, respectively. The gray center shifted at 3.7±5.8% in patients. The range of gray gradation area was consistent with three different background both in controls and patients. The range of patients was significantly smaller than controls and the gray center in patients shifted more than controls, which might result from a disorder of automatic gain control system in patients.

**Grant:** none

## 

**Keywords:** contrast sensitivity, eye disease, psychophysics, handy measurements

## Gender Recognition Biased by Facial Beauty, Likability, and Attractiveness

**Kanako Mitsuhiro and Akiyoshi Kitaoka**

Ritsumeikan University

## 

It has been found that faces that emphasize feminine traits are evaluated more attractive than the originals whereas those that emphasize masculine traits are not. If gender differences in faces affect evaluations of facial attractiveness, it is suggested that facial attractiveness might affect gender judgement. In this study, we examined the relationships between three different evaluations (beauty, likability, and attractiveness) and gender judgement. Participants rated the beauty, likability, and attractiveness of 36 illustrated, neutral faces with 6-point scale, and subsequently they judged the gender of each face. As a result, faces that were evaluated more highly tended to be judged as female in any of three evaluations. This finding suggests a possibility that aesthetic evaluation of faces might be used as a clue for gender recognition of faces.

**Grant:** none

## 

**Keywords:** face, attractiveness, gender recognition, illustrated face

## Hand-Written Chinese Calligraphy Activated the Mirror Neuron System

**Wei-Li Tu, Shwu-Lih Huang, Guang-Yi Lai and Yun-An Yu**

National Chengchi University

## 

As Umilta, Berchio, Sestito, Freedberg, and Gallese (2012) found that mu rhythm suppression was evoked by the observation of original artworks by Lucio Fontana, the result might suggest that even the static artworks could arise the cortical motor activation while there were some motor actions implied. Following this view, our research assumed that the hand-written words might play the same role while people appreciating the Chinese calligraphy artworks. We used four kinds of typefaces as the stimuli, including two hand-written styles (regular script and running hand) and two printed fonts (Ming and boldface). EEG data was recorded as the Chinese participants viewing every single-word stimuli one by one. Our result demonstrated that the hand-written words led to higher cortical activation of mirror neuron system (MNS) as reflected by mu rhythm suppression in C3 C4 channel compared to the printed words. And the running hand, which is more dynamic, induced more mu rhythm suppression in contrast to the regular script. The result supported that the implied motor information of artworks might initiate the beholders’ MNS and reflected in EEG data.

**Grant:** none

## 

**Keywords:** Neuroaesthetics, Vision science, Art

## The Geometric, Not Photometric Structure of Synthetic Images Determines Discrimination Sensitivity, Perceived Similarity and Visual Preferences

**Leena Y Duyen Nguyen, Zoey Isherwood and Branka Spehar**

School of Psychology, University of New South Wales

## 

Despite the apparent variety and diversity of natural scenes, their statistical structure is non-random and highly ordered. Photometric (intensity based) analyses of natural scenes show that a majority of signal amplitude is distributed at low spatial frequencies, with amplitude dropping off towards high spatial frequencies: known as a 1/f α linear function whose slope for natural scenes averages ∼1.25. Previous work has demonstrated that discrimination sensitivity and preferences peak in response to stimuli with natural α. However, recent work has suggested that both neural response and visual preference are better accounted for by geometric (contour based) image properties across a wide range of synthetic images. This study investigates discrimination sensitivity, perceived similarity and visual preference across three types of synthetic images: grayscale (GS); black and white thresholded (TH) and edges only (ED). While the photometric properties vary substantially across different image types, their geometric properties remain stable. Measured discrimination thresholds and visual preference both peaked at natural α and were very stable across image type. Additionally, Multidimensional Scaling analysis of perceived similarity between different images types supports these conclusions. This suggests that geometric image properties predominantly drive the observed sensitivity and preference across a diverse set of synthetic images.

**Grant:** none

## 

**Keywords:** Natural scene statistics, Psychophysics, Aesthetics, Similarity

## Emotion Modulates Multiple Colors for a Short Duration

**Noriyo Shibata^1^ and Ko Sakai^2^**

^1^Graduate school of Systems and information Engineering, University of Tsukuba

^2^Department of computer science, University of Tsukuba

## 

The relationship between color and emotion has been investigated for a long time but not clarified in detail. We examined whether emotional states (happy, angry, and neutral) modulate color perception, where the emotion was controlled by showing affective face images. Participants were asked to choose one out of three color test patches that appeared most similar to the reference. Fifteen reference colors were evenly selected in Lab color space with L fixed, and the test colors were 6 or 12 degree away from the reference. The results showed that there was no difference in the discrimination accuracy across emotion, but there were significant differences in the directions of the chosen colors (bias). We observed that happy and angry significantly extended the perceptual range of red and shifted green-blue, but neutral in the opposite directions. The results indicate that happy and angry showed the same tendency, and that the emotion affected discontinuous multiple colors. We also examined the effective duration of the modulation by performing a delayed-match-to-sample test. The observed bias disappeared within the duration of 2s, which might correspond to the time course of emotion.

**Grant:** none

## 

**Keywords:** psychophysics, emotion, color

## Is Trypophobia Affected by Size Illusion?

**Kanichi Fukumoto and Kenji Yokoi**

National Defense Academy of Japan

## 

Aversion to clusters of holes or warts is known as trypophobia. In our previous study, we manipulated various spatial properties, such as frequency, size, viewing distance, and so on, to clarify the crucial factor for trypophobia. The results revealed that trypophobic discomfort was depended on the size of stimuli, not on the frequency that has been thought to be the important feature of trypophobic images. Concerning the size of stimuli, however, the subjective rating suggested that the apparent size should be effective, while the pupillary response indicated that the retinal size might affect discomfort. To clarify the relationship between the size of images and trypophobia furthermore, we adopted the Ebbinghaus illusion. The images containing holes or warts were presented at the center with surrounding inducers. The distance and the size of inducers were manipulated to change the apparent size of the central image while keeping the retinal size constant. Participants were asked to rate both discomfort and arousal against the images. Their pupillary responses were also recorded. The relationship among the apparent size, the retinal size, and trypophobia was discussed.

**Grant:** none

## 

**Keywords:** Trypophobia, Discomfort, Pupil, Illusion

## Identifying Cortical Area for Processing of Emotional Facial Expressions in ADHD Children Measured by Near-Infrared Spectroscopy

**Megumi Kobayashi^1^, Masako Nagashima^2^, Tatsuya Tokuda^3^, Takahiro Ikeda^2^, Yukifumi Monden^2,4^, So Kanazawa^5^, Masami K Yamaguchi^6^, Ryoichi Sakuta^7^, Takanori Yamagata^2^ and Ippeita Dan^3^**

^1^Institute for Developmental Research, Aichi Developmental Disability Center

^2^Department of Pediatrics, Jichi Medical University

^3^Applied Cognitive Neuroscience Laboratory, Chuo University

^4^Department of Pediatrics, International University of Health and Welfare

^5^Department of Psychology, Japan Women’s University

^6^Department of Psychology, Chuo University

^7^Child Development and Psychosomatic Medicine Center, Dokkyo Medical University Saitama Medical Center

## 

Previous studies reported that children with ADHD had impairment in negative emotional facial expressions, especially angry, but not in positive facial expressions (e.g., Pelc et al., 2006), and that they also showed atypical brain activity to angry expressions (Ichikawa et al., 2014). However, little is known about neural basis of recognition of facial expressions in ADHD children. We used near-infrared spectroscopy to measure nineteen ADHD children’s hemodynamic responses in the bilateral temporal-occipital areas during presentation of happy and angry facial expressions before and after methylphenidate (MPH) or placebo administration. Because MPH is considered to increase synaptic transmission and consequently improve cerebral processing and cognitive performance (e.g., Monden et al., 2012), we predicted that the cortical areas involved in recognition of angry showed increased hemodynamic responses by MPH administration. We found that for happy expressions, ADHD children showed increased hemodynamic responses in the right inferior occipital gyrus (IOG) regardless of medication. In contrast, for angry expressions, the left IOG significantly activated by MPH administration, but not by placebo administration. Our results suggest that (1) ADHD children’s processing of facial expressions would rely on information of physical forms of faces, (2) MPH administration facilitate processing of physical form information of angry expressions.

**Grant:** This work was supported in part by JST-RISTEX to ID, and the Grant-in-Aid for Scientific Research from the Japan Society for Promotion of Science (19K14492) to MK

## 

**Keywords:** facial expression, attention-deficit/hyperactivity disorder, brain, NIRS

## The Effect of Mood State on Visual Search Times under VR Environment

**Ikuhisa Mitsugami^1^, Miyu Sakai^1^, Rina Yonami^1^, Jun’ichi Murakami^1^ and Noriko Yamagishi^2^**

^1^Hiroshima City University

^2^Ritsumeikan University

## 

It was reported that an individual’s perceived happiness level affects their ability of visual search (Maekawa et al., 2018). In their work, however, the visual search task was performed on a small smartphone display. It is unclear whether this tendency is effective even under more general conditions, like in our daily lives. This work expanded the small search area to all circumferences (canvas size = 22.6 × 360 deg) by introducing Virtual Reality techniques to investigate whether the tendency is effective under such conditions. We employed a pop-out and serial visual search paradigm, and implemented a VR application that allowed search times and self-rated levels of happiness to be recorded. Each participant performed a session three times a day, for two weeks. From collected data of 23 participants, we confirmed that we were able to replicate the classic visual search findings, whereby pop-out search times remained largely unaffected by the number of distractors whereas serial search times increased with increasing number of distractors. While pop-out search times were unaffected by happiness level, serial search times with the maximum numbers of distractors (n = 240) were significantly faster for high happiness levels than low happiness levels (p<0.05).

**Grant:** none

## 

**Keywords:** visual search, VR, emotion, experience sampling

## Convolutional Neural Networks for humanlike Image Assessment

**Hassan Matout^1,2^, Hao Wang^2^, Yasuhiro Hatori^2,3^, Yoshiyuki Sato^3,4^, Kazuya Matsubara^5^, Yuji Wada^6^, Chia-huei Tseng^2,3^, Ichiro Kuriki^2,3^, Satoshi Shioiri^2,3,4^**

^1^Department of Computer Science, TU Dortmund University

^2^Graduate School of Information Science, Tohoku University

^3^Research Institute of Electrical Communication, Tohoku University

^4^Advanced Institute for Yotta Informatics; Tohoku University

^5^BKC Research Organization of Social Sciences, Ritsumeikan University

^6^College of Gastronomy Management, Ritsumeikan University

## 

One serious problems in an information-intensive society is to face too much information to handle, which affects working efficiency as well as cost for storing and processing. Obviously, prioritizing and deleting data is crucial, so we investigated the usage of Convolution Neural Networks as decision support system for high level human judgments such as preference of images. For this purpose, we used a lunchbox dataset, containing 760 images and subjective judgments by 2120 subjects (Wada & Matsubara, 2018). Each image of the dataset was rated for 6 qualities (“want to eat”, “made for young/old”, etc.) on a discrete scale (1 to 6). To compensate the small size of the dataset, we fine-tuned several layers of a trained CNN for object recognition (AlexNet). The network was tested with one quarter of the dataset after training with three quarters. Results have mean absolute error of 0.34 on average, suggesting that high-level human judgments could be estimated with dataset size of less than 1000 with a considerable accuracy. Differences in prediction rate are also seen among different judgments. Subjective qualities (such as gender) yield larger errors (0.48) compared to objective qualities (0.28). In conclusion, CNNs are a decent approach for estimating human judgments.

**Grant:** none

## 

**Keywords:** image assessment, human preference, convolutional neural networks

## Attitudes to Vision Science and Phenomenology: A Survey

**David Rose**

University of Surrey

## 

There has been much interest recently in the definition of illusions and their implications for the relationship between perception and reality. Several symposia have been organized (at ECVP in 2015, 2017 and 2019), special issues of journals have been published, a series of editorials in Perception, chapters in a major compendium, and an extensive discussion took place on CVNet in early 2016. For example Koenderink (Perception 43, 1–6, 2014) has claimed that scientists believe in “a silly delusion”—that there exists an objective reality, which exists even when not observed. Also, we are mistaken if we believe it is the aim of the visual system to construct, to the best of its ability, a veridical representation or model of that external reality. Our common understanding of illusions as deviations from the perception of physical reality is thus challenged. Informal discussions with my fellow researchers have shown that many of them agree with Koenderink, while many others do not. At this poster I will collect data on the relative popularities of these two views, to try to find the roots of this divergence. Is one view held by a majority? Does the difference correlate with whether researchers have been trained in ‘continental’ phenomenology or ‘oriental’ philosophy, as opposed to ‘anglophone’ or ‘scientific’ worldviews?

**Grant:** none

## 

**Keywords:** philosophy of perception, illusions

## Enhanced Perceptual Sensitivity for Negative Facial Expressions in Trait Anxiety Individuals

**Li-Chuan Hsu^1,2^, Yi-Min Tien^3^, Chia-Yao Lin^1,2^ and Ya-Ting Wu^4^**

^1^School of Medicine, China Medical University

^2^Graduate Institute of Biomedical Sciences

^3^Department of Psychology, Chung Shan Medical University

^4^College of Public Health, China Medical University

## 

Individuals with high trait anxiety often demonstrate attentional bias when they meet threatening stimulus. Furthermore, these people also show higher sensitivity of recognizing fearful faces than low trait anxiety ones. We aim to examine whether high trait anxiety people would display higher sensitivity in perceiving other types of facial expressions, such as angry faces. Trait anxiety was assessed for participants by using the State-Trait Anxiety Inventory- Trait Scale. In Experiment 1, we manipulated emotion intensity of four facial expressions (happiness, fear, sadness, anger). Participants had to detect any emotionality of the presented faces. In Experiment 2, by blending two facial expressions with reciprocal proportions, three sets of morphed faces were created: the happy face gradually morphs and blends with one of the negative faces (fear, sadness, or anger). For example, a face consists 20% happy and 80% fearful expression. Participants were asked to discriminate the facial expression as positive or negative. The results revealed the group with high trait anxiety expressed significantly higher sensitivity to sad, angry and fearful faces compared to the group with low trait anxiety. It suggests that an individual with trait anxiety showed negative bias for facial expressions.

**Grant:** MOST 106-2629-H-040-001 & MOST 107-2410-H-039-002

## 

**Keywords:** facial expression, emotion, psychophysics

## Differential Effects of Color on Fear and Disgust

**Hiromi Sato^1^, Shiori Muranaka^2^, Wakana Hata^2^ and Isamu Motoyoshi^1^**

^1^Department of Life Sciences, The University of Tokyo

^2^Department of Integrated Sciences, The University of Tokyo

## 

Natural scenes evoke a variety of affective response in humans. In particular, negative emotions such as fear and disgust are known to prompt immediate avoidance from threat or danger in the environment. This leads us to a notion that, independently from the recognition of objects and scenes, some negative emotions can be directly summoned by simple image features rapidly encoded in the visual cortex (e.g., Motoyoshi & Mori, 2017). The present study examined how manipulation of simple image features affects the emotional valence of natural scenes and reports that color (and luminance) have asymmetric effects on fear and disgust. In separate blocks, observers rated the pleasantness-vs.-disgust and the happiness-vs.-fear of 200 natural scenes, their achromatic version, and their RGB-inverted version. We found that (1) removal of color information strongly reduces disgust, but not fear, and (2) RGB-inversion reduces disgust while increasing fear. The results further demonstrate the impact of image features on emotion and suggest distinct visual pathways (e.g., Magno/Parvo streams) involved in neural processing of fear and disgust.

**Grant:** Supported by the Commissioned Research of NICT, and by JSPS KAKENHI JP15H05916 and 18H04186.

## 

**Keywords:** psychophysics, emotional valence, color

## The Difference in Emotions Aroused by Self- and Automatically-Controlled Facial Expressions

**Kana Ohashi^1^ and Takao Sato^2^**

^1^Department of Psychology, Ritsumeikan University, ^2^College of Comprehensive Psychology, Ritsumeikan University

## 

In self-controlled condition, observer made emotional expressions, smiling or sad faces, and the real-time video image was presented on a display. In automatic-condition, the expressions were automatically generated by a computer by deforming the neutral face of the observer. The observer observed one of these stimuli for 90 seconds while conducting unrelated distracter tasks, poking a circle presented at a random position within the stimulus field. After the observation period, observers’ emotional status after the observation was measured by a questionnaire (IPANAT). Observers were also asked whether they noticed the expression was manipulated in the automatic condition. It was found that positive impression increases and negative impression decreases for the self-generated condition relative to the automatic condition regardless the type of expressions used. In addition, the score for helplessness was higher when observers noticed the manipulation of expression in the automatic condition. These results indicate that, when observers observe their own face, the difference in who generates the expression (observers themselves or computer), affects the emotional status of observers. In addition, the results suggest the sense of agency is an important factor for arousing emotions.

**Grant:** none

## 

**Keywords:** emotion, facial expression, sense of agency

## Salience: A Comparative Study of the Experience of Visual Art in the Laboratory and the Art Museum

**Vicente Estrada-Gonzalez^1^, Branka Spehar^1^, Michael Garbutt^2^ and Scott East^2^**

^1^School of Psychology, University of New South Wales

^2^Art & Design Faculty, University of New South Wales

## 

Since experimental aesthetics have mostly focused on fine arts, visual salience has become a relevant concept to understand how individuals experience aesthetics. Salience modulates gaze behaviour by attracting eye fixations towards conspicuous elements of the visual field. This characteristic is mainly determined by low-level visual properties such as colour and spatial frequency content. Nevertheless, it has also been demonstrated that other factors, such as the context in which artworks are exhibited, can significantly influence salience computation and, in doing so, eye gaze is modulated. Additionally, context has been demonstrated to affect aesthetic experience as well. For that reason, the aim of this study is to demonstrate whether 1) people look at artworks differently depending on the context they are presented (museum or lab). Secondly, 2) if peoplés art experience is correlated to specific eye movements (e.g. Fixation Duration, Number of Fixations) and finally, 3) to explore if salience is modulated by the context. We conducted a study where artworks were presented in two different conditions (in-museum and on-screen), while participants’ eye movements were recorded by an eye-tracker. The preliminary results show that some components of the gazing behaviour (i.e., Average Fixation Duration) are different when participants consume art in the museum than when works of art are shown on a screen.

**Grant:** none

## 

**Keywords:** empirical aesthetics

## Emotional Facial Detection in the Foveal and Peripheral Vision

**Wan-Ting Huang and Pi-Chun Huang**

Department of Psychology, National Cheng Kung University (NCKU)

## 

The ability to detect emotional facial expressions is an important aspect of social cognition. Processing facial expressions in central vision has been extensively studied, but processing it in peripheral vision has rarely been studied. This study compared the emotional facial detection ability of the central and peripheral visual field. Three basic facial expressions, including happy, angry, and sad, were morphed from a neutral expression to nine different intensities (10% to 90%), respectively. The faces were presented in either fovea or the peripheral visual field (8 degrees in the left and right visual field), and participants were asked to decide whether the presented face showed an emotion. The subjective judgement and the response time were recorded. Our results showed that participants required a similar level of morphing to detect emotions when the target was presented in the fovea. A happy facial expression required a higher morphed level, but the other two facial expressions required a lower morphed level for the participant to decide if the face expressed an emotion. The response times were shortest for angry and sad faces, and longest for happy faces. Our results suggests that facial expression processing is different in the foveal and peripheral vision.

**Grant:** This work was supported by Minister of Science and Technology in Taiwan (MOST 104-2628-H-006-001-MY3) to PCH.

## 

**Keywords:** facial expression, morphed task, peripheral vision

## Symmetry or Beauty at First Sight: Neural Responses to Human Face Symmetry and Attractiveness

**Chih-Chia Hsing and Pi-Chun Huang**

Department of Psychology, National Cheng Kung University

## 

Previous studies have shown a positive correlation between facial symmetry and facial attractiveness. However, few studies have investigated how the brain processes interactions between them. This study adopts 36 pairs of photographs containing asymmetrical human faces and their symmetrical versions as our stimuli. Functional magnetic resonance imaging (fMRI) was conducted while the subjects performed the symmetry rating task and the attractiveness rating task. Behavioral results showed that the symmetrical versions of the stimuli received higher ratings in both tasks. A comparison of fMRI data between symmetrical and asymmetrical stimuli showed activation at the right superior parietal gyrus in the symmetry task, whereas no activation was found during the attractiveness task. Another analysis was conducted according to the difference in Ratings between the two versions of the same face. The analysis revealed greater activity at the reward- and emotion-related brain areas while assessing attractiveness, whereas activity in the right inferior occipital gyrus and the right anterior cingulate gyrus increased in the symmetry task. Our results were in line with previous studies. Nevertheless, while earlier research describes the assessment of facial attractiveness as automated, our study demonstrated that evaluation of facial symmetry is not an automated process.

**Grant:** This work was supported by Minister of Science and Technology in Taiwan (MOST 104-2628-H-006-001-MY3) to PCH.

## 

**Keywords:** fMRI, facial attractiveness, facial symmetry

## Trial Order Effect in Size Preference 2AFC Judgment Task About Natural Scene Moving Images

**Masamitsu Harasawa^1,2^, Yasuhito Sawahata^1^, Yamato Miyashita^1^, Kazuteru Komine^1^ and Satoshi Shioiri^2^**

^1^Japan Broadcasting Corporation

^2^Tohoku University

## 

We measured the preferred size of natural scene moving images (5 secs) by method of constant stimuli where participants reported their preference of size by 2AFC judgment, enlarge / shrink, on 100 moving images with 7 steps (25–100% to original) of physical size. 255 participants performed 700 trials separated into 6 sessions consisted of 116 or 117 trials where presentation order of stimuli was randomized for each participant. To investigate the effect of trial order, we calculated the 50% response ratio threshold as a preferred size for each trial number from 1st to 700th by gathering 255 trials from all participants. The preferred size showed shrinking with progress of the experiment and recovers in few minutes of breaks between the sessions. On average, the preferred size shrunk 9.7% for a whole experiment and 7.1% for a session, respectively. While these trends seem to be derived from some adaptation to stimulus exposure, it is unclear why the adaptation appeared as shrinking instead of enlarging. In addition, 2AFC responses showed negative correlations between adjacent trials. This effect lasted for six trials and suggested that participants tried to avoid repeating the same responses.

**Grant:** none

## 

**Keywords:** size preference, aesthetic judgment, trial order effect, psychophysics

## Impaired Emotion Processing in Voice and Content Is Associated with Alexithymia, the Inability to Name and Describe Feelings

**Yutong Lin^1,2^, Xuanchang Lin^1,2^, Hong Xu^1,2^, Kalon Sou^1,2^, Fun Lau^1,2^**

^1^Nanyang Technological University

^2^Raffles Institution

## 

Alexithymia, the difficulty in interpreting and verbalizing one’s own and others’ feelings, has received heightening attention due to its potential correlation with various clinical illnesses and social dysfunctioning. Our study specifically examines the integration of emotional information from voice and speech content and if there exists any correlation with alexithymic traits. Vocal stimulus used in the experiment comprises of validated and randomized recordings that can be either congruent or incongruent in terms of the emotions conveyed through voices and contents. Two blocks of experiments were carried out for 24 participants to rate the emotion within either voice or sentence content independent of the other. Participants with varied alexithymia levels as calculated by the Toronto Alexithymia Scale (TAS 20) display differed abilities to identify content or voice emotions with a different combination of voice and content emotions. The results suggest that those with higher alexithymic level have greater difficulty in dissociating emotional information in speech, especially from the emotional content when voices are varied, and the effect is the most pronounced when they are exposed to a material with a sad voice. Therefore, the existing approach to mitigate the effect of alexithymia may need to be further revised and enhanced.

**Grant:** none

## 

**Keywords:** Alexithymia, Emotion processing, Voice, Content

## Effect of Task-Irrelevant Ugly-Beauty Faces on Involuntary Attention: ERP Study and Individual Differences

**Eriko Matsumoto^1^, Tomoya Kawashima^2^ and Tomoyuki Naito^3^**

^1^Kobe University

^2^NICT (national institute of information and communications technology)

^3^Osaka University

## 

The aim of this study is to examine how ugly or beauty faces capture attention even they were completely task-irrelevant. Many studies have reported that human face attracts attention powerfully. Especially, attractive faces or threat-related faces capture more attention. Several neuroimaging studies have reported the emotional brain network for ugly stimuli perception, however, little is known the difference of the effects on involuntary attention between beauty and ugly. We employed the letter detection task to measure the distractibility of attention by peripheral images (beauty face, ugly face, and control stimuli) with recording the distractor time-locked ERPs. In this task, participants were asked to detect the target letter with ignoring peripheral stimuli. We used artificial ugly/beautiful faces as distractors created by using a psychophysical reverse-correlation technique (Naito et al., 2017). The results showed that N2pc peak amplitude was modulated by the face presentation compared to control images but there were no significant difference between face conditions. LPP amplitude was significantly reduced when ugly face was presented. Negative correlation was observed between LPP amplitude and the correct detection rate with ugly face distractor. The results suggest that ugly faces could have higher distractibility feature than other faces.

**Grant:** none

## 

**Keywords:** attention, face, ERP

## Oral Session 4-1 (August 1, 2019): Cognition

## Perceived Dominance from Face Images Depends on Interaction Situations: Examinations of Consistency Across Japanese and Taiwanese and the Own-Race Bias

**Yoshiyuki Ueda^1^, Bo-Cheng Huang^2^, Su-Ling Yeh^2^ and Sakiko Yoshikawa^1^**

^1^Kokoro Research Center, Kyoto University

^2^Department of Psychology, National Taiwan University

## 

Facial features and expressions can be used as important nonverbal cues to infer other’s personality traits and status such as perceived dominance. Most previous studies used a single face presentation to investigate this issue; however, in everyday life our communication is not limited to one-on-one situations. For example, Ueda and Yoshikawa (2018) showed that while faces with angry/disgusted expressions were perceived as more dominant in one-on-one situations, those showing happy expressions were perceived as more dominant in two-person interaction scenes instead. In this study, using Caucasian and East Asian faces, Japanese and Taiwanese participants judged which person was more dominant in two-person interaction scenes to examine the possibility of idiosyncrasy of Japanese participants and the own-race bias of face perception. The results showed that i) both Japanese and Taiwanese judged that people showing happy expressions were dominant in two-person interaction scenes regardless of image races, suggesting that concealing negative expressions against disgusted persons make themselves more dominant, and ii) people showing happy expressions were perceived as more dominant for Japanese than Taiwanese. These findings indicate both commonality and difference in the signal of facial expressions for perceived dominance.

**Grant:** none

## 

**Keywords:** facial expression, face recognition, dominance, own-race bias

## A Novel Neural Mechanism for Autistic Vision Through Rose Coloured Spectacles

**David Philip Crewther^1^, Laila Hugrass^2^ and Eveline Mu^1^**

^1^Centre for Human Psychopharmacology, Swinburne University of Technology

^2^School of Psychology and Public Health, La Trobe University

## 

Autism shows consistent visual abnormalities, particularly in magnocellular processing. Also, saccadic suppression—loss of visual contrast sensitivity associated with a rapid eye movement appears to be selective for low spatial frequencies (attributed to magnocellular selection) only in individuals with high autistic tendency. Primate studies suggest such suppression arises in superior colliculus (SC) to pulvinar (PUL) inhibition, also that early PUL inputs to cortical area MT gradually withdraw, replaced by inputs from area V1. Thus, we propose that the withdrawal process in autism is not as complete as in typical development. Altered balance of LGN vs PUL inputs to cortex could be achieved through inhibition of the Type IV M cells which are suppressed by red light and which project to LGN but not SC. We tested this hypothesis by looking at the effects on ERP P100 and N170 responses to facial emotion in those high (n = 22) and low (n = 21) in autistic tendency using red and green backgrounds of equal luminance. For the low AQ group, red surrounds reduced the effect of fear on P100 amplitude. For the high AQ group, red surrounds enhanced low spatial frequency fearful P100 amplitudes, implying a disinhibition of amygdala connected attentional input.

**Grant:** none

## 

**Keywords:** EEG, psychophysics, autistic vision, facial emotion

## Perceptual Learning along the “Weaker” Principal Meridian Improves Contrast Sensitivity Function and Visual Acuity in Patients with Astigmatism

**Li Gu^1^, Jinrong Li^1^, Jing Zhong^1^, Zhipeng Chen^1^, Zhong-Lin Lu^2^ and Yuan Jin^1^**

^1^State Key Laboratory of Ophthalmology, Guangdong Provincial Key Lab of Ophthalmology and Visual Science, Zhongshan Ophthalmic Center, Sun Yat-sen University

^2^Department of Psychology, The Ohio State University

## 

Astigmatism before visual development results in abnormal visual development due to principal meridional variations in visual processing. Ten subjects with with-the-rule astigmatism (13.90±1.73 years) participated in baseline assessments, which consisted of visual acuity and CSFs measured with both vertical and horizontal sinewave gratings. They were then trained in a luminance grating orientation identification task (±5°) around the vertical direction at their individual cutoff SF, whichever had relatively poorer CSF. Post-training assessments were the same as the baseline. Results from the baseline tests showed that the cut-off SF on the horizontal meridian was lower than that on the vertical meridian (t9 = 1.94, p = 0.084), demonstrating differential effects of astigmatism on visual processing in different meridians. Additionally, training in the weaker meridian near cut-off SF led to significant improvements in contrast sensitivity at the trained SF measured with vertical gratings (4.50 dB or 67.96%; t9 = 2.81, p = 0.020). No significant improvement was found in contrast sensitivity at the trained SF measured with horizontal gratings. Moreover, the training improved visual acuity of the trained eye by 3.70 dB (or 53.17%). These findings demonstrate effects of astigmatism on visual processing and provide empirical evidence for perceptual learning as a potential treatment for astigmatism.

**Grant:** none

## 

**Keywords:** psychophysics, astigmatism, perceptual learning

## Short-Term Source Amnesia Does Not Persist in Auditory Modality

**Ping Zhu, Mengjiao Xu, Yingtao Fu, Jiahan Yu, Mowei Shen and Hui Chen**

Department of Psychology and Behavioral Sciences, Zhejiang University

## 

Failing to remember the source of retrievable information is known as source amnesia. This phenomenon has been extensively investigated in long-term memory but rarely in short-term or working memory, as we share the intuition that source information of an item that we have encountered momently before is always available. However, a recent study (Chen, Carlson, & Wyble, 2018) challenged this common sense by showing a striking phenomenon of source amnesia for simple visual stimuli in the context of working memory when participants did not expect to report source information. The current study sought to explore the boundaries of short-term source amnesia by testing whether it persists in complex and meaningful stimuli in the visual modality (Experiment 1), cross-visual-and-auditory modalities (Experiments 2a & 2b), and within-auditory modality (Experiment 3). The results revealed that short-term source amnesia was a stable phenomenon in the visual modality, whereas it was absent in the cross-visual-and-auditory or within-auditory modalities, regardless of reporting expectation. This indicated differences between working memory representations of auditory stimuli and that of visual stimuli, namely, the former was automatically bound to the corresponding original sources, while the latter was stored independently of its source information.

**Grant:** National Natural Science Foundation of China (No.31771201), and National Science Foundation for Distinguished Young Scholars of Zhejiang Province, China (No. LR19C090002).

## 

**Keywords:** working memory, source memory

## Memory-Driven Capture Is at the Level of Features Not Objects

**Edyta Sasin and Daryl Fougnie**

New York University of Abu Dhabi

## 

Previous findings have shown that items in memory capture attention (memory-driven capture). Research has also shown that people are able to selectively maintain a subset of the features previously encoded into working memory. Here, we examined whether memory-driven capture operates at the level of the object or at the level of an individual feature. After remembering the color and orientation of a triangle, participants were instructed, via cue, whether the color, the orientation, or both features had to be remembered for a later continuous report. To measure memory-driven capture we asked participants to execute a subsequent search task and we compared performance in displays that did and did not contain memory-matching feature. The results showed that color attracted attention only when it was indicated as task relevant by the cue. The attentional capture by color was reduced when both color and orientation had to be held in memory and it was eliminated when color was indicated as no longer relevant. There was no evidence of capture by orientation. Taken together, our results suggest that both memory selection and memory-driven attentional capture operate on individual features, supporting the notion that features are stored separately rather than in an integrated form.

**Grant:** none

## 

**Keywords:** memory-driven capture, working memory, attentional selection

## The Storage of Dynamic Relations in Visual Working Memory

**Jing Chen, Jifan Zhou and Mowei Shen**

Department of Psychology and Behavioral Sciences, Zhejiang University

## 

The ability of briefly holding and manipulating object relations provides a foundation stone for interacting with the complex environment around us. As the first step to understand the storage mechanism of object relations in visual working memory (VWM), we measured the capacity of holding dynamic relations of objects, by using a modified change-detection paradigm with moving objects as memory items. Participants were required to memorize the object relations showed by the dynamic display in which several pairs of objects moved under each other’s constraint (e.g., two moving balls connected by a spring). After holding a while, participants reported whether the relation of probe objects was identical to one of the memorized relations. The results showed that participants were able to hold approximately 2 relations, whether the relation was familiar (e.g., piston motion) or unfamiliar (e.g., synchronous vibration). When the complexity of relations increased (i.e., an object simultaneously related to more objects) the memory accuracy decreased. These findings suggested that the VWM capacity of holding dynamic relations depended on the complexity of relation. For the display containing simple relations, in which each object related to only one object, the capacity is 2; while it was even less for more complex relations.

**Grant:** The National Natural Science Foundation of China (No. 31871096, 31600881)

## 

**Keywords:** visual working memory, object relation, dynamic relation, memory capacity

## Differential Neural Activation to Explicit and Implicit Moral Processing

**Dan Tao and Yue Leng**

Southeast University

## 

Explicit moral judgement was often accompanied by active attention and explicit response to moral information in previous studies. However, implicit moral information could also affect other cognitive processes that have no relation with moral judgment. The present study aimed to investigate the differences of neural temporal dynamics between the implicit and explicit harm/care moral processing. Participants were enrolled and the event-related potential (ERP) responses were recorded while viewing 192 images indicating moral behaviors based on harm/care with high/low arousal. Half participants were instructed whether pictures represented moral or immoral behavior (explicit task) while the other half were instructed whether pictures involved kids or animals, which served as distraction stimulate (implicit task). The results showed that distraction stimulate was more disturbed under the high-arousal moral condition(indexed by accuracy and reaction time); For both implicit and explicit task, moral information could cause early attention of the subject (indexed by the N1, P1, and N2); Attention of moral rightness only affect the early period in explicit task, while the implicit task was influenced in the whole phase (indexed by P300, Parietal-occipital LSW); The difference between two tasks may introduced by the regulatory capacity of inhibitory control to moral cues (indexed by Frontal LSW).

**Grant:** none

## 

**Keywords:** implicit moral processing, explicit moral processing, behavioral method, event-related potential

## ‘Sociality’ Matters in Emotion Perception of Visual Narratives

**Jinyoung Kim, Yeonwha Kim and Sang-Hun Lee**

Department of Brain and Cognitive Sciences, Seoul National University

## 

Visual perception is typically conceptualized as inferring external world states from visual sensory measurements, whereby people are assumed to utilize prior knowledge about the physical environment to compensate for the imprecision of sensory measurements. By the same token, visual perception can also be directed toward internal emotional states when people navigate and act in their social environment, whereby certain prior knowledge about the social environment are expected to assist people to read nuanced emotions out of ambiguous visual narratives unfolding in various social contexts. We reasoned that such prior knowledge required for emotion perception is likely to vary from individual to individual depending on their personality traits, particularly those associated with ‘sociality’ – the extent to which individuals engage in social activities or transactions. To explore this idea, we characterized the personality traits of human observers with a multitude of psychometric questionnaires and assessed their emotion perception by asking them to assign affective states to a wide range of visual narratives (short excerpts from movies). Using multivariate analyses, we discovered a tight linkage between personality traits and emotion perception: individuals characterized with strong sociality traits tend to assign more intensified affective states to visual narratives, compared to those with weak sociality traits.

**Grant:** The Brain Research Programs and Basic Research Laboratory Program through the National Research Foundation of Korea (NRF2015M3C7A1031969; NRF-2017M3C7A1047860; NRF-2018R1A4A1025891)

## 

**Keywords:** Visual cognition, High-level visual perception, Psychophysics, Canonical correlation analysis

